# Association Between Plasma Metabolomic Profile and Machine Learning‐Based Brain Age

**DOI:** 10.1111/acel.70208

**Published:** 2025-09-01

**Authors:** Yang Li, Jiao Wang, Yuyang Miao, Michelle M. Dunk, Yan Liu, Zhongze Fang, Qiang Zhang, Weili Xu

**Affiliations:** ^1^ Aging Research Center, Department of Geriatrics Tianjin Medical University General Hospital, Tianjin Geriatrics Institute Tianjin China; ^2^ Department of Toxicology and Health Inspection and Quarantine School of Public Health, Tianjin Medical University Tianjin China; ^3^ National Clinical Research Center for Geriatrics West China Hospital, Sichuan University Chengdu China; ^4^ Aging Research Center, Department of Neurobiology, Care Sciences and Society Karolinska Institute Stockholm Sweden; ^5^ Department of Health Care, the Second Medical Center Chinese PLA General Hospital Beijing China

**Keywords:** brain age, machine learning, magnetic resonance imaging, metabolites, UK biobank

## Abstract

Metabolomics has been associated with cognitive decline and dementia, but the relationship between metabolites and brain aging remains unclear. We aimed to investigate the associations of metabolomics with brain age assessed by neuroimaging and to explore whether these relationships vary according to apolipoprotein E (*APOE*) ε4. This study included 17,770 chronic brain disorder‐free participants aged 40–69 years from UK Biobank who underwent neuroimaging scans an average of 9 years after baseline. A total of 249 plasma metabolites were measured using nuclear magnetic resonance spectroscopy at baseline. Brain age was estimated using LASSO regression and 1079 brain MRI phenotypes and brain age gap (BAG; i.e., brain age minus chronological age) was calculated. Data were analyzed using linear regression. We identified 64 and 77 metabolites associated with brain age and BAG, respectively, of which 55 overlapped. Lipids (including cholesterol, cholesteryl esters, free cholesterol, phospholipids, and total lipids) in S/M‐HDL, as well as phospholipids and triglycerides as a percentage of total lipids in different‐density lipoproteins, were associated with larger BAG. The percentages of cholesterol, cholesteryl esters, and free cholesterol to total lipids in VLDL, LDL, and HDL of different particle sizes were associated with smaller BAG. The associations of LA/FA, omega‐6/FA, SFA/FA, and phospholipids to total lipids in L‐HDL with brain age were consistent across *APOE* ε4 carriers and non‐carriers (all *p* for interaction > 0.05). Plasma metabolites show remarkably widespread associations with brain aging regardless of *APOE* ε4 genetic risk. Metabolic profiles could serve as an early indicator of accelerated brain aging.

## Graphical abstract

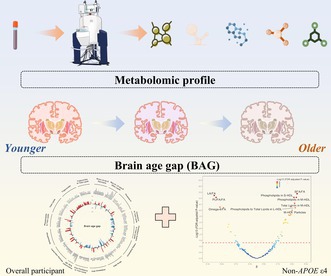

**Abbreviations**
Apo: apolipoprotein
BAG: brain age gap
BCAAs: branched‐chain amino acids
BMI: body mass index
CI: confidence interval
DHA: docosahexaenoic acid
FA: fatty acid
FDR: False discovery rate
HDL: high‐density lipoprotein
HDL‐AD: average diameter for HDL particles
IDL: intermediate‐density lipoprotein
LA: linoleic acid
LASSO: Least Absolute Shrinkage and Selection Operator
LDL: low‐density lipoprotein
LDL‐AD: average diameter for LDL particles
MRI: magnetic resonance imaging
MUFA: monounsaturated fatty acid
NMR: nuclear magnetic resonance
PUFA: polyunsaturated fatty acid
SFA: saturated fatty acid
TDI: Townsend Deprivation Index
TG: triglycerides
VLDL: very low‐density lipoprotein
VLDL‐AD: average diameter for VLDL particles

## 1 Introduction

Metabolomics has been used to identify novel biomarkers for the diagnosis and monitoring of chronic diseases (Griffin et al. 2011; Hasin et al. 2017). In addition, metabolomics can provide valuable insights into the pathophysiology of age‐related diseases by sensitively capturing metabolic alterations even at the pre‐clinical stage (Tynkkynen et al. 2018). In brain magnetic resonance imaging (MRI) studies, blood metabolites, including lipid subclasses, fatty acids, amino acids, and inflammatory biomarkers, have been linked to regional brain volumes, white matter microstructure, cortical thickness, and microstructural lesions (Harshfield and Markus 2023; Sliz et al. 2022, 2020, 2021).

In recent years, machine learning has been utilized to model brain age from an aggregation of multimodal brain MRI phenotypes. By integrating multiple MRI measures, brain age captures subtle structural changes and intricate connections among various brain regions, thus providing a comprehensive picture of the individual's brain aging process (Bzdok 2017; Cole 2020). Brain age gap (BAG), the difference between predicted brain age and chronological age, further reveals whether an individual's brain age deviates from the norm and is recognized as a significant biomarker of neurological health and aging (Baecker et al. 2021).

To date, three landmark studies have explored the association of metabolic signatures with MRI‐derived brain aging measures (Gordon et al. 2024; Kim et al. 2023; Tian et al. 2023). Gordon et al. (2024) and Kim et al. (2023) established brain age using T1‐weighted structural MRI data without considering phenotypes from other MRI modalities. Tian et al. (2023) made significant strides by developing multiorgan aging clocks including the brain age using the multimodal neuroimaging phenotypes and the metabolic system integrating blood biomarkers associated with lipids and glucose metabolites, and then suggested metabolic perturbation and accelerated brain aging. However, this study assessed the health of the metabolic system with limited metabolites and did not explore a wider range of metabolites as well as assess individual metabolites in relation to brain age. Thus, we still need to further explore the association of broad metabolites with brain age/BAG.

The apolipoprotein E (*APOE*) ε4 allele—the greatest genetic risk factor for Alzheimer's dementia—has been associated with brain health (such as brain microstructure, gray matter, and hippocampal volume) (Malek‐Ahmadi et al. 2023). Beyond its established links to brain health, emerging epidemiological evidence reveals that *APOE* ε4 carriers exhibit distinct plasma metabolomic profiles characterized by altered lipid metabolism (e.g., lower high‐density lipoprotein cholesterol, elevated triglyceride‐rich lipoproteins) and neuroinflammation, which may accelerate brain aging independently of amyloid *β* deposition (Montagne et al. 2021; Nakamura et al. 2023). Critically, *APOE* ε4 carriers exhibit metabolic shifts decades before clinical dementia onset, positioning these profiles as potential early indicators of neurobiological decline. However, there is a lack of direct evidence on whether metabolites alter the risk of *APOE*‐associated brain aging.

To fill these knowledge gaps, we conducted a comprehensive examination of the association between circulating metabolites and brain age in over 30,000 middle‐aged and older adults, utilizing detailed neuroimaging data from the UK Biobank covering 6 different MRI modalities. Specifically, we aimed to (1) assess the associations of metabolites with brain age and BAG, and (2) explore the role of *APOE* ε4 carrier status in these associations.

## 2 Methods

### 2.1 Study Design and Participants

The UK Biobank recruited 502,412 participants aged 37–73 years between 2006 and 2010 from 22 assessment centers. The UK Biobank study received ethical approval from the North West Multi‐Centre Research Ethics Committee (Ref 11/NW/0382) and all registered participants provided informed and written consent. This research was conducted using the UK Biobank resource (Application Number 67048).

Following baseline assessment, a subsample of 42,806 participants consented to undergo MRI assessment from 2014 to 2020. Following the exclusion of participants with incomplete brain MRI (*n* = 8510), we used the remaining individuals with complete brain MRI data (*n* = 34,296) for subsequent analysis. We then further excluded those with ICD‐10 diagnosed somatic disease (UK Biobank‐field ID 41270), self‐reported long‐term illness, disability, or frailty (field ID 2188), or self‐reported fair or poor health (field ID 2178), yielding a sample of 4355 healthy participants. These participants were randomly allocated in a 4:1 ratio to a training set (*n* = 3484, for training the brain age model) and a validation set (*n* = 871, for validating model performance). The remaining 29,941 participants were included in the testing set.

**FIGURE 1:**
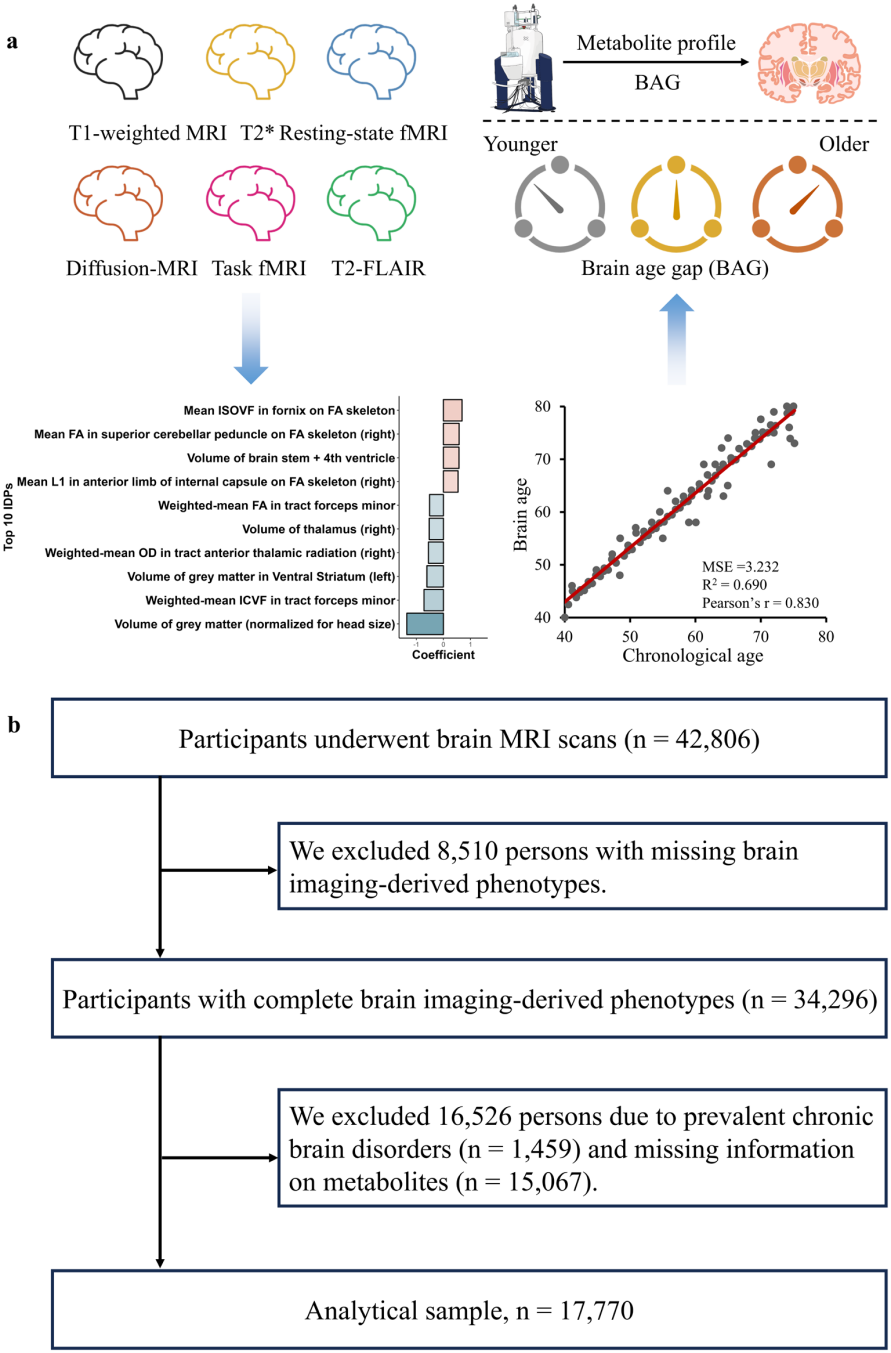
(a) Study overview. Six MRI modalities used to estimate brain age, namely T1‐weighted MRI, T2*, Resting‐state fMRI, Diffusion‐MRI, Task fMRI, and T2‐FLAIR. Top 10 IDPs for LASSO modeling; (b) Flowchart of participants included in the study.

After further excluding 16,526 participants with chronic brain diseases (*n* = 1459; detailed code is available in Table S1) and missing information on metabolites (*n* = 15,067), a total of 17,770 participants were included in the final study sample (Figure 1). The study population included 17,770 participants, among whom 51.95% were female, with a mean (SD) age of 54.73 (7.48) years. Participant characteristics are detailed in Table 1.

**TABLE 1:** Baseline characteristics of the study population (*n* = 17,770).

Characteristics	Total participants
Age (year)	54.73 ± 7.48
Female	9232 (51.95)
Race‐White	16,490 (92.80)
TDI	−2.69 (−3.94, −0.69)
Education‐College	8180 (46.03)
BMI (kg/m^2^)	26.57 ± 4.17
Smoking status	
Never	10,853 (61.07)
Former	5793 (32.60)
Current	1088 (6.13)
Alcohol drinking status	
Never	440 (2.48)
Former	353 (1.99)
Current	16,971 (95.50)
Regular physical activity	12,797 (72.01)
High social connection	13,685 (77.01)
Hypertension	7902 (44.47)
Diabetes	462 (2.60)
Heart disease	641 (3.61)
Medication	
Beta‐blockers	669 (3.76)
Calcium blockers	593 (3.34)
Lipid‐lowering	1789 (10.07)
*APOE* ε4 carrier	4155 (23.38)
Brain age (year)	63.82 ± 9.07
Brain age gap (year)	0.35 ± 5.16

*Note:* Data are presented as mean ± standard deviations, median (interquartile range), or *n* (%). Missing data: Education = 56; Race = 46; TDI = 16; Smoking status = 36; Alcohol drinking status = 6; BMI = 15; Physical activity = 573; Social connection = 43; *APOE* ε4 carrier status = 2670.

Abbreviations: *APOE* ε4, apolipoprotein E epsilon; BMI, body mass index; TDI, Townsend Deprivation Index.

### 2.2 Measurement of Metabolomics

Ethylenediaminetetraacetic acid (EDTA) plasma samples were collected in a non‐fasting state, following the UK Biobank protocol. However, the sampling time was 4 h after the last meal, so the effect on metabolite concentrations was minor. Metabolites in EDTA plasma samples collected at baseline were quantified using a high‐throughput nuclear magnetic resonance (NMR)‐based metabolic biomarker analysis platform developed by Nightingale Health Ltd. Detailed information on metabolite processing can be found online (https://biobank.ctsu.ox.ac.uk/ukb/ukb/docs/nmrm_companion_doc). In this study, we included 249 metabolic biomarkers, including 168 absolute levels and 81 derived ratios. The comprehensive biomarker profile included cholesterol, fatty acids, and low‐molecular‐weight metabolites, such as ketone bodies, amino acids, and glycolysis‐related metabolites. All metabolites were Z‐transformed before analysis.

### 2.3 MRI Data Acquisition and Pre‐Processing

Brain MRI scans were performed between 2014 and 2020 at 4 MRI assessment centers using standard Siemens Skyra 3 T scanners running VD13A. Details on the UK Biobank brain MRI data protocol and processing pipeline are available on the UK Biobank website in the brain scan protocol and brain imaging documentation (biobank.ndph.ox.ac.uk/crystal/ukb/docs/brain_mri.pdf). Summary measurements of imaging‐derived phenotypes were generated with an image‐processing pipeline developed and run on behalf of the UK Biobank, using publicly available image processing tools FSL (the FMRIB Software Library, version 5.0.10, http://fsl.fmrib.ox.ac.uk/fsl) and FreeSurfer (version 6.0). A total of 1079 neuroimaging phenotypes were generated from six modalities, including T1‐weighted (*n* = 165), T2‐FLAIR (*n* = 1; total volume of white‐matter hyperintensities), SWI (*n* = 14), diffusion‐MRI (*n* = 675), task fMRI (*n* = 14), and resting‐state fMRI (*n* = 210) (Table S2).

### 2.4 Assessment of Brain Age and BAG

A total of 1079 MRI phenotypes were first Z‐transformed, followed by training of nine machine learning models to calculate brain age in the training dataset. These models included least absolute shrinkage and selection operator (LASSO) regression, eXtreme gradient boosting, and support vector regression, each combined with one of three feature selection approaches: no feature selection, FeatureWiz, or recursive feature elimination with cross‐validation. To identify the best hyperparameter, Bayesian optimization was employed across 100 epochs (Tables S3 and S4). After optimizing the hyperparameters, each model was tested on the validation set for performance comparison. Finally, the LASSO model without feature selection performed best (with mean absolute error = 3.232, *R* ^2^ = 0.690, and Pearson's *r* = 0.830) (Table S5) and was therefore selected for predicting brain age across the entire population. Among the 1079 imaging‐derived phenotypes (IDPs), 285 were found to have a significant contribution to the brain age estimation, and their details are provided in Table S6.

In addition, we corrected for the age bias commonly observed in brain age prediction, that is corrected brain age = (original brain age‐*β*)/α, where the coefficients *α* and *β* are the slope and intercept, respectively, of the linear regression model for estimating brain age from chronological age in the training set (brain age _training set_ = *α* * chronological age _training set_ ± *β*). BAG was calculated by subtracting chronological age from corrected brain age. A positive BAG indicates accelerated brain age, and a negative BAG reflects a younger, healthier brain than expected based on chronological age. This correction adjusts for residual age correlations but does not alter the model's intrinsic performance metrics, which were finalized before correction.

### 2.5 Assessment of Covariates

Upon study entry, participants underwent a comprehensive physical examination and clinical evaluation. In addition, information on demographic characteristics, socioeconomic status, and lifestyle factors was collected via a computerized touchscreen questionnaire.

Education was categorized as college or non‐college. Race was dichotomized as white or non‐white (including Asian, Black, and mixed background). Townsend Deprivation Index (TDI), which combined information on housing, employment, and car availability, was calculated based on census and postcode before participant recruitment, reflecting the subject's socioeconomic status. Smoking and alcohol drinking status were both categorized as never, former, or current. Body mass index (BMI) was calculated as weight (kg) divided by height (m^2^). Regular physical activity was defined as ≥ 150 min of moderate‐intensity activity per week, or ≥ 75 min of vigorous‐intensity activity per week or its equivalent. Social connection was evaluated based on responses to the question “How often do you visit friends or have them visit you?” and classified as high (“almost daily”, “2–4 times a week”, and “about once a week”) and low levels (“about once a month”, “once every few months”, “never or almost never”, and “no friends/family outside household”).

Hypertension was defined as systolic blood pressure ≥ 140 mmHg, diastolic blood pressure ≥ 90 mmHg, use of antihypertensive drugs, or a history of hypertension. Heart disease including coronary artery disease, congestive heart failure, and atrial fibrillation was assessed based on self‐reported and registered medical history. Diabetes was defined as hemoglobin A1c ≥ 6.5%, fasting plasma glucose ≥ 126 mg/dL, a history of diabetes, or the use of glucose‐lowering medications.

*APOE* was genotyped from blood samples, and a detailed genotyping process can be found at http://www.ukbiobank.ac.uk/scientists‐3/genetic‐data. Single nucleotide polymorphism data for rs429358 and rs7412 were used to determine the *APOE* genotype. *APOE* genotypes were classified as ε2 carriers (genotypes ε2ε2), ε3 homozygotes (ε3ε3), ε4 heterozygotes (ε3ε4), or ε4 homozygotes (ε4ε4). The *APOE* gene was genotyped and dichotomized as carriers versus non‐carriers of the ε4 allele.

### 2.6 Statistical Analysis

Baseline characteristics of study participants were described as mean (SD) and median (25th–75th percentiles) for continuous variables and number (%) for qualitative variables. All statistical analyses were performed using R (version 4.1.0). With two‐tailed testing, a *p‐*value < 0.05 was considered statistically significant.

Linear regression models were used to estimate *β* coefficients and 95% confidence intervals (CIs) for the association of metabolites with brain age and BAG. The basic models were adjusted for age, sex, and education. Multivariable‐adjusted models were further adjusted for race, socioeconomic status, body mass index, smoking status, alcohol drinking status, physical activity, social contact, hypertension, diabetes, heart disease, *β*‐blockers, calcium blockers, lipid‐lowering medications, and *APOE* ε4 carrier status. Stratified analyses were performed to explore the role of *APOE* ε4 in the association of metabolites with brain age and BAG. Statistical interaction was examined by creating an indicator variable with the cross‐product of two variables. Benjamini‐Hochberg for false discovery rate (FDR) was used to correct for the *p*‐value.

In sensitivity analysis, we repeated the analyses after (1) using chained‐equation multiple imputation to impute the missing values or covariates (including race, education, TDI, BMI, smoking status, alcohol drinking status, physical activity, social connection, and *APOE* ε4 carrier status) and (2) using BAG calculated based on brain age estimated from other candidate machine learning models.

## 3 Results

### 3.1 Association of Individual Metabolites With Brain Age and BAG

**FIGURE 2:**
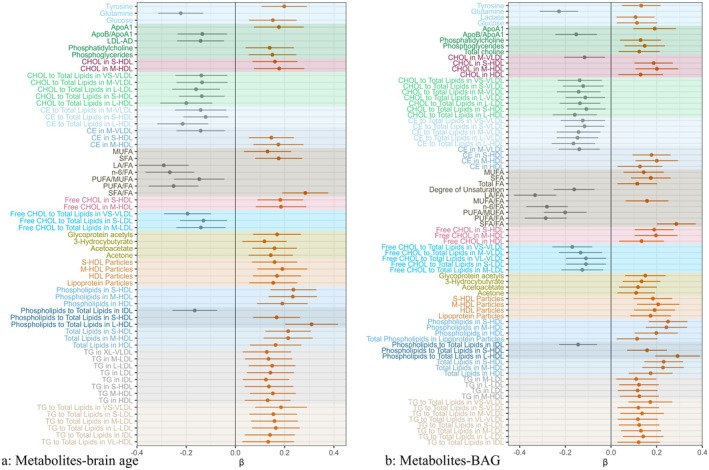
Benjamini‐Hochberg for false discovery rate‐corrected metabolites significantly associated with (a) brain age and (b) brain age gap. Orange point and line: Metabolite associated with brain age/BAG with β and 95% confidence interval (CI) of > 0; gray point and line: Metabolite associated with brain age/BAG with β and 95% CI of < 0. All models adjusted for age, sex, education, race, socioeconomic status, body mass index, smoking status, alcohol drinking status, physical activity, social connection, hypertension, diabetes, heart disease, beta‐blockers, calcium blockers, lipid‐lowering, and *APOE* ε4 status. Apo, apolipoprotein; CE, cholesteryl esters; CHOL, cholesterol; FA, fatty acid; HDL, high‐density lipoprotein; IDL, intermediate‐density lipoprotein; L, large; LA, linoleic acid; LDL, low‐density lipoprotein; LDL‐AD, average diameter for LDL particles; M, medium; MUFA, monounsaturated fatty acid; n‐6, omega‐6 fatty acid; PUFA, polyunsaturated fatty acid; S, small; SFA, saturated fatty acid; TG, triglycerides; VL, very large; VLDL, very low‐density lipoprotein; XL, extremely large.

The association of individual metabolites with brain age and BAG was assessed with the use of linear regression models. In the multi‐variable models, 64 (25.70%) and 77 (30.92%) metabolites were associated with brain age and BAG, respectively (Tables S7 and S8), and the overlap of significant metabolites between brain age and BAG was 55 (22.09%) (Figure 2 and Figure S1).

Cholesterol, cholesteryl esters, free cholesterol, phospholipids, and total lipids in S/M‐HDL (e.g., brain age: *β*_CHOL in S‐HDL_ = 0.160, 95% confidence interval [CI] = 0.069–0.252; BAG: *β*_CHOL in S‐HDL_ = 0.186, 95% CI = 0.103–0.270), as well as phospholipids and triglycerides as a percentage of total lipids in different density lipoproteins, were associated with older brain age and larger BAG (e.g., *β* and 95% CI of TG/Total lipids in S‐LDL, 0.154 [0.057, 0.250] for brain age; 0.123 [0.035, 0.212] for BAG). Meanwhile, tyrosine, glucose, ApoA1, phosphatidylcholine, phosphoglycerides, monounsaturated fatty acid (MUFA), SFA, SFA/FA, glycoprotein acetyls, and three ketone bodies (e.g., brain age: *β*_Acetoacetate_ = 0.159, 95% CI = 0.068–0.249; BAG: *β*_Acetoacetate_ = 0.118, 95% CI = 0.035–0.201) also were related to older brain age and larger BAG. However, glutamine, ApoB/ApoA1, certain fatty acid ratios (e.g., *β* and 95% CI of LA/FA, −0.291 [−0.392, −0.190] for brain age; *β* and 95% CI of −0.333 [−0.425, −0.240] for BAG), and the percentages of cholesterol, cholesteryl esters, and free cholesterol to total lipids in VLDL, LDL, and HDL of different particle sizes were associated with younger brain age and smaller BAG.

### 3.2 Role of *APOE* ε4 Status

Among *APOE* ε4 non‐carriers, 43 and 45 metabolites were associated with brain age and BAG, respectively. In contrast, only four metabolites were related to BAG among *APOE* ε4 carriers (Tables S9–S12).

**FIGURE 3:**
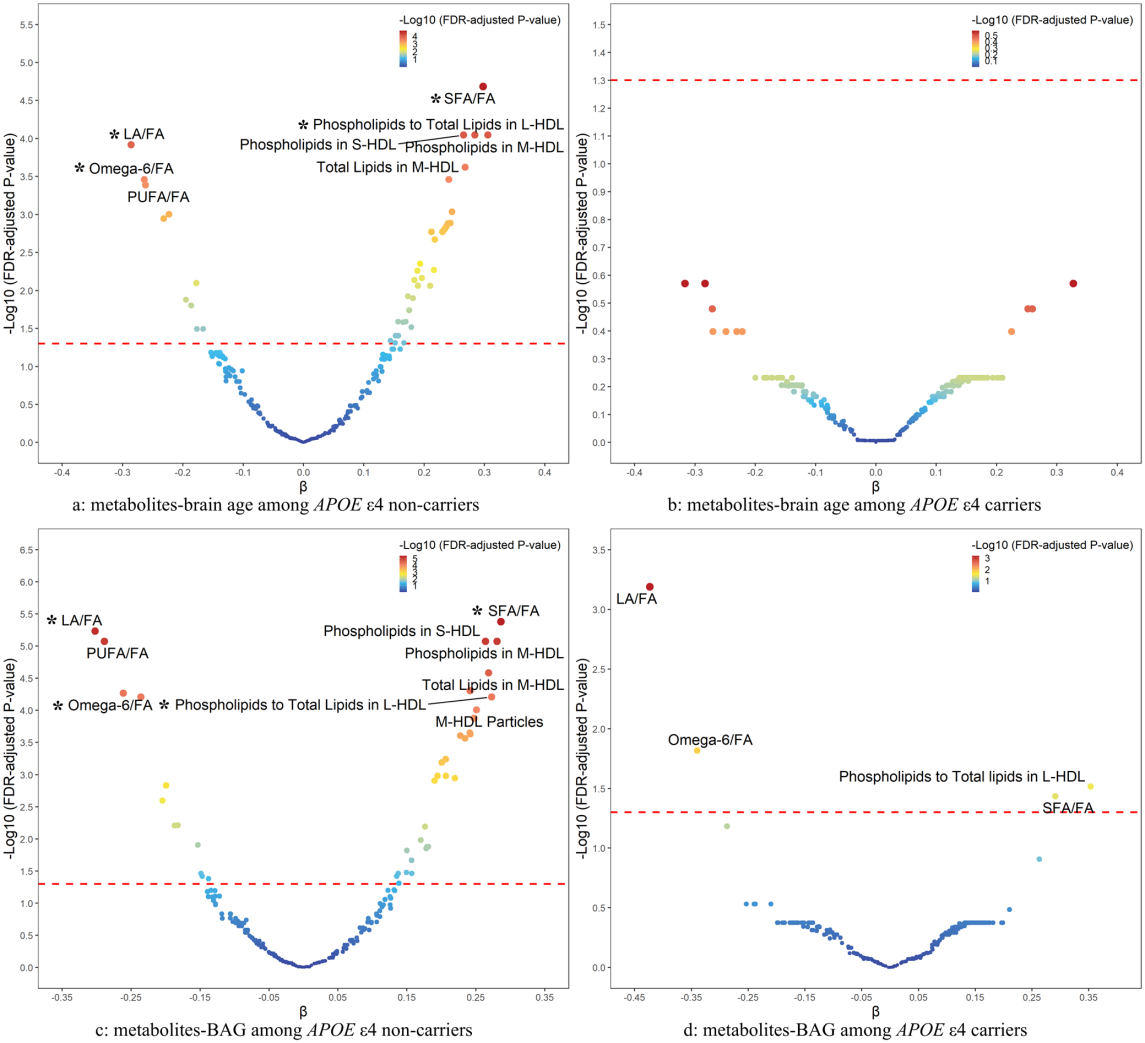
Association of metabolites with brain age and BAG stratified by *APOE* ε4 carrier status. (a/b) *APOE* ε4 non‐carriers/carriers and brain age; (c/d) *APOE* ε4 non‐carriers/carriers and BAG. All models adjusted for age, sex, education, race, socioeconomic status, body mass index, smoking status, alcohol drinking status, physical activity, social connection, hypertension, diabetes, heart disease, beta‐blockers, calcium blockers, and lipid‐lowering. FA, fatty acid; HDL, high‐density lipoprotein; LA, linoleic acid; PUFA, polyunsaturated fatty acid; SFA, saturated fatty acid. * indicates no significant interaction effect of LA/FA, omega‐6/FA, SFA/FA, and phospholipids to total lipids in L‐HDL with *APOE* ε4 on brain age/BAG (*P*‐interaction > 0.05)

Among *APOE* ε4 non‐carriers, 8 identical and all absolute effect values > 0.25 metabolites were related to both brain age and BAG (Figure 3a,c). Interestingly, 4 metabolites associated with BAG among *APOE* ε4 carriers (i.e., LA/FA, omega‐6/FA, SFA/FA, and phospholipids to total lipids in L‐HDL) were similarly identified among non‐*APOE* ε4 carriers (Figure 3d). SFA/FA and phospholipids to total lipids in L‐HDL had positive effects on brain aging, whereas LA/FA and omega‐6/FA exerted negative effects. There were no statistical interactions of LA/FA, omega‐6/FA, SFA/FA, and phospholipids to total lipids in L‐HDL with *APOE* ε4 in relation to brain age or BAG in the multi‐variable adjusted models (all *p*‐interactions > 0.05). However, we observed significant associations of DHA (*p* = 0.031), DHA/FA (*p* = 0.012), omega‐3/FA (*p* = 0.019), and omega‐6/omega‐3 (*p* = 0.023) with *APOE* ε4 on BAG before multiple testing correction, while these associations were not significant after FDR adjustment (Table S13).

### 3.3 Sensitivity Analysis

The associations of metabolites with brain age and BAG were not greatly altered when we repeated the analyses after (1) performing multiple imputations to account for missing values of covariates (including race, education, TDI, BMI, smoking status, alcohol drinking status, physical activity, and social connection; Tables S14 and S15), (2) using brain age and BAG calculated from 8 other machine learning models (Table S16).

## 4 Discussion

In this large neuroimaging study with multimodal MRI measures, we constructed a brain age prediction model based on LASSO regression and found that (1) 64 and 77 metabolites were associated with brain age and BAG respectively, including lipoprotein subclasses, fatty acids, glucose, inflammatory biomarkers, ketone bodies, and tyrosine; and (2) regardless of *APOE* ε4 carrier status, the significant relationship of fatty acids and lipoprotein subclasses with brain age remained.

### 4.1 Comparison With Previous Research and Interpretation of Our Findings

Previous studies have identified higher specific lipid components (such as phospholipids and cholesteryl esters) in S/M‐HDL to be associated with neuroimaging signatures (white matter hyperintensities, mean diffusivity, higher mean diffusivity, and fractional anisotropy) (Harshfield and Markus 2023; Sun et al. 2023) and clinical endpoints (dementia and stroke) (de Leeuw et al. 2021; Huang et al. 2023; van der Lee et al. 2018), which is consistent with our findings. However, these studies predominantly relied on unidimensional assessments of cerebral integrity, potentially overlooking the multidimensional nature of brain health. Current understanding of metabolomic correlates of brain age remains nascent, with only three prior studies systematically evaluating this relationship (Gordon et al. 2024; Kim et al. 2023). Among these, two research‐derived brain age estimates solely from T1‐weighted structural MRI, inherently limit their capacity to capture the multiscale aging process spanning microstructural and functional decline. Tian et al. used multimodal neuroimaging to estimate brain age, linking BAG to specific lipid and glucose metabolites. Our work builds on this by estimating brain age/BAG from multiple neuroimaging phenotypes across six MRI modalities and incorporating 249 plasma metabolites. We found that multiple lipids in S/M‐HDL are associated with larger brain age and BAG. Additionally, this study identified cholesterol‐like substances in VLDL, LDL, and HDL as a percentage of lipids that were likewise related to brain age and BAG. This phenomenon may be caused by differences in the function and ability of various lipoprotein subclasses to promote cholesterol‐like efflux (Navab et al. 2005; Rader and Hovingh 2014).

The present study observed that higher MUFA, SFA, total FA, MUFA/FA, and SFA/FA as well as lower LA, n‐6/FA, PUFA/FA, degree of unsaturation, and PUFA/MUFA are associated with higher brain age, consistent with previous studies on dementia risk and brain structure (e.g., mean diffusivity, white matter hyperintensities, and fractional anisotropy). While mechanistic pathways cannot be directly inferred from observational data, evidence from preclinical and clinical studies contextualizes these findings. PUFA modulates neurogenesis, neuroplasticity, and neurotransmitter metabolism to exert properties that reduce neurodegeneration and maintain cognitive function in aging (Daiello et al. 2015). Conversely, elevated SFA levels may promote neuroinflammation through ceramide accumulation (Gupta et al. 2012). In preclinical dementia studies, neuroinflammation leads to a reduction in inhibitory activity by impairing inhibitory GABAergic signaling (Crowley et al. 2016), thereby triggering hyperexcitability (Maestú et al. 2021). Current research on the association of MUFA with brain health is limited and inconsistent. The majority of studies link higher MUFA based on dietary assessments to better cognitive performance and lower risk of dementia (Zhang et al. 2020). However, some studies observe positive associations of MUFA in the metabolic profile with the risk of aging‐related disease (Lian and Vardhanabhuti 2024), which is in line with our finding that MUFA is linked to higher brain age/BAG. Notably, animal studies have found that MUFA disrupts insulin homeostasis via its toxic effects on *β* cells (Plötz et al. 2017; Zhang et al. 2005) and in turn is associated with brain aging (Chen et al. 2022).

This study found an association between higher glucose levels and larger brain age and BAG. Substantial evidence suggests that higher glucose is strongly associated with abnormal brain functional connectivity, WMH, brain atrophy, and cortical thinning (Chen et al. 2014; de Leeuw et al. 2021; Qiang et al. 2024; Wennberg et al. 2016). Higher glucose accelerates the aging of the brain through several mechanisms, including glucose neurotoxicity (Sommerfield et al. 2004), insulin resistance (Candeias et al. 2012), inflammation (Schmidt et al. 1999), and cardiovascular events (Kalmijn et al. 2000). Additionally, the study identified a novel NMR‐derived biomarker of systemic inflammation, namely glycoprotein acetyls (GlycA), which is associated with higher brain age and BAG. The association of GlycA with WMH and poorer cognitive performance is in line with our findings. Beyond glucose and inflammatory markers, ketone bodies exhibit significant associations with energy metabolism. Dysregulated energy metabolism is a modifier or even proximate cause of brain aging (Kapogiannis 2015). Our findings that ketone bodies are associated with higher brain age are consistent with other research that links elevated ketone bodies to decreased hippocampal volume and an increased risk of dementia (de Leeuw et al. 2021; Huang et al. 2023). However, several studies have also found that ketone bodies are related to improved cognitive performance and a lower risk of dementia (Reger et al. 2004; Shippy et al. 2020). This apparent paradox may arise from context‐dependent effects: ketone bodies are an effective alternative energy substrate for the brain under physiological conditions (e.g., fasting), which can strengthen mitochondrial function and reduce inflammatory mediators (Rusek et al. 2019). Conversely, persistently elevated circulating ketone bodies in non‐fasting states (e.g., insulin resistance) may indicate metabolic dysregulation, thereby contributing to neurovascular damage (Meneilly and Tessier 2016). The role of ketone bodies in the mechanisms of brain aging is limited, and further studies are needed to clarify the molecular mechanisms involved.

Kim et al. (2023) reported significant associations of glucose and lipid metabolites with BAG derived from T1‐weighted structural imaging information. Building on this, we developed a more comprehensive brain age model by integrating multimodal IDPs encompassing brain structure, function, and connectivity. While our model exhibits a higher MAE than that reported by Kim et al. the integration of multimodal information enhances our ability to gain deeper insights into the complexity of brain aging, thereby extending and advancing the findings of Kim et al.

### 4.2 Role of *APOE* ε4

The role of *APOE* ε4 as a key genetic risk factor in brain age has been extensively studied. *APOE* ε4 has been associated with poor microstructure and functional networks in the brain (Malek‐Ahmadi et al. 2023; Turney et al. 2020), and a higher risk of dementia (Graff‐Radford et al. 2021). The *APOE* ε4 allele also affects the level of metabolites in the body, including inflammation, oxidative stress, small molecules related to glucose metabolism, and lipid homeostasis (Ahmad et al. 2024; Liu et al. 2013; Reiman et al. 2004). The higher brain age observed in *APOE* ε4 carriers (BAG: 0.48 ± 5.42 vs. 0.25 ± 5.10 years in non‐carriers, *p* < 0.005) likely reflects both amyloid‐dependent and amyloid‐independent pathways (Frick et al. 2024). *APOE* ε4 disrupts astrocytic lipid metabolism and mitochondrial function, driving neuroinflammation and synaptic loss even in A*β*‐negative models (Bell et al. 2012). For instance, *APOE* ε4 impairs cholesterol transport across the blood–brain barrier, exacerbating oxidative stress independent of the amyloid pathway (Montagne et al. 2021). We found that fatty acid and lipoprotein subclasses were associated with brain health irrespective of *APOE* ε4 status, with no significant interaction between these metabolites and *APOE* on brain age. This discrepancy may reflect a dual mechanism of gene‐metabolism interaction. Among *APOE* ε4 carriers, the dominant role of *APOE* ε4 driven pathological cascades (e.g., amyloid aggregation) overshadowed metabolic contributions in carriers. Conversely, in non‐carriers, peripheral metabolic dysregulation (e.g., lipid proportion imbalance) may directly exacerbate neuroinflammation and mitochondrial dysfunction, thereby accelerating brain aging. These findings underscore the need for genotype‐stratified interventions targeting metabolic pathways in brain aging.

### 4.3 Strength and Limitations

Strengths of this study include exploring for the first time the associations of various metabolites with predicted brain age using data from multiple imaging modalities in a large‐scale community‐based population with a comprehensive data collection procedure. Nevertheless, some limitations should be acknowledged. Firstly, participants in the UK Biobank cohort are healthier than the general population (Fry et al. 2017). Additionally, our study's analytical sample consisted of participants who underwent brain MRI scans and were free of chronic brain diseases, which is a relatively healthier subset of the overall UK Biobank population. This may result in an overestimation or underestimation of the extent of the associations between certain metabolites and brain health. Second, the approach used in this study to calculate brain age vis multi‐modal IDPs information may reduce model performance compared to methods relying on intuitive imaging information. Third, plasma from the UK Biobank was extracted in a non‐fasting state but an average of 4 h from the last meal (Julkunen et al. 2023), suggesting minimal bias in our findings. However, future studies with fasting samples could further validate our results. Fourth, while the metabolomics platform captured a wide range of lipids and lipid subspecies, it provided limited coverage of low‐molecular‐weight metabolites. Fifth, although our findings suggest the potential effects of metabolites on brain aging, causality cannot be inferred due to the observational design. Future work using repeat brain imaging data now available in the UK Biobank will allow longitudinal assessment of brain aging trajectories and their metabolic correlates. Moreover, while our study provides important associations, we did not perform Mendelian randomization (MR) analyses to examine potential causal links. Although we acknowledge that MR is a powerful tool for causal inference, the current availability of large‐scale GWAS summary statistics for brain age phenotypes is limited. Future studies leveraging well‐powered GWAS data for both brain aging and metabolomic traits are needed to establish causality more robustly. Finally, caution must be taken when generalizing our findings to other ethnic groups because the majority of participants in the UK Biobank were white. Emerging evidence indicates cardiometabolic risks (e.g., obesity, hypertension) disproportionately accelerate brain aging in Global South populations due to compounded health disparities. Future multi‐ethnic studies should validate findings and optimize precision prevention strategies.

## 5 Conclusion

In conclusion, we employed a novel study method to identify multiple metabolomic biomarkers—namely lipoprotein subclasses, fatty acids, glucose, inflammatory biomarkers, ketone bodies, and amino acids—that were significantly associated with brain age. Furthermore, we observed that the association of fatty acids and lipoprotein subclasses with brain age was consistent across *APOE* ε4 carriers' status. The metabolites identified in this study may complement future hypotheses explaining the intricate relationships of lipid homeostasis and energy metabolism with brain health.

## Author Contributions

**Yang Li:** formal analysis, visualization, writing – original draft. **Jiao Wang:** conceptualization, data curation, writing – review and editing, funding acquisition. **Yuyang Miao:** writing – review and editing. **Michelle M. Dunk:** writing – review and editing. **Yan Liu:** writing – review and editing. **Zhongze Fang:** writing – review and editing, funding acquisition. **Qiang Zhang:** writing – review and editing. **Weili Xu:** conceptualization, funding acquisition. All authors reviewed the manuscript.

## Conflicts of Interest

The authors declare no conflicts of interest.

## Supporting information


**Table S1:** Self‐reported health variable codes used for exclusion criteria on initial population.
**Table S2:** Missing number for brain imaging‐derived phenotypes.
**Table S3:** Coefficients for 285 IDPs that significantly contribute to brain age estimation in the LASSO regression without feature selection.
**Table S4:** Hyperparameter spaces of 9 models in Bayesian optimization.
**Table S5:** The chosen hyperparameters of 9 models.
**Table S6:** Model evaluation of 9 models in the validation set and testing set.
**Table S7:** *β* coefficients and 95% confidence intervals (CIs) for the association between metabolites and brain age: results from linear regression models.
**Table S8:** *β* coefficients and 95% confidence intervals (CIs) for the association between metabolites and brain age gap (BAG): results from linear regression models.
**Table S9:** *β* coefficients and 95% confidence intervals (CIs) for the association between metabolites and brain age among non‐*APOE* ε4 carriers: results from linear regression models.
**Table S10:** *β* coefficients and 95% confidence intervals (CIs) for the association between metabolites and brain age gap (BAG) among non‐*APOE* ε4 carriers: results from linear regression models.
**Table S11:** *β* coefficients and 95% confidence intervals (CIs) for the association between metabolites and brain age among *APOE* ε4 carriers: results from linear regression models.
**Table S12:** *β* coefficients and 95% confidence intervals (CIs) for the association between metabolites and brain age gap (BAG) among *APOE* ε4 carriers: results from linear regression models.
**Table S13:**. The interaction effect of metabolites with *APOE* ε4 status on brain age gap (BAG).
**Table S14:** *β* coefficients and 95% confidence intervals (CIs) for the association between metabolites and brain age after multiple imputation of covariates: results from linear regression models.
**Table S15:** *β* coefficients and 95% confidence intervals (CIs) for the association between metabolites and brain age gap (BAG) after multiple imputation of covariates: results from linear regression models.
**Table S16:** *β* coefficients and 95% confidence intervals (CIs) for the association between metabolites and brain age gap (BAG) within eight other candidate machine learning models: results from linear regression models.
**Figure S1:** 249 individual metabolites in relation to (a) brain age and (b) brain age gap.

## Data Availability

Data from this study are available through the UK Biobank (https://www.ukbiobank.ac.uk/).

## References

[acel70208-bib-0001] Ahmad, S. , W. Yang , A. Orellana , et al. 2024. “Association of Oxidative Stress and Inflammatory Metabolites With Alzheimer's Disease Cerebrospinal Fluid Biomarkers in Mild Cognitive Impairment.” Alzheimer's Research & Therapy 16, no. 1: 171. 10.1186/s13195-024-01542-4. PMC11287840 39080778

[acel70208-bib-0002] Baecker, L. , R. Garcia‐Dias , S. Vieira , C. Scarpazza , and A. Mechelli . 2021. “Machine Learning for Brain Age Prediction: Introduction to Methods and Clinical Applications.” eBioMedicine 72: 103600. 10.1016/j.ebiom.2021.103600. 34614461 PMC8498228

[acel70208-bib-0003] Bell, R. D. , E. A. Winkler , I. Singh , et al. 2012. “Apolipoprotein E Controls Cerebrovascular Integrity via Cyclophilin A.” Nature 485, no. 7399: 512–516. 10.1038/nature11087. 22622580 PMC4047116

[acel70208-bib-0004] Bzdok, D. 2017. “Classical Statistics and Statistical Learning in Imaging Neuroscience.” Frontiers in Neuroscience 11: 543. 10.3389/fnins.2017.00543. 29056896 PMC5635056

[acel70208-bib-0005] Candeias, E. , A. I. Duarte , C. Carvalho , et al. 2012. “The Impairment of Insulin Signaling in Alzheimer's Disease.” IUBMB Life 64, no. 12: 951–957. 10.1002/iub.1098. 23129399

[acel70208-bib-0006] Chen, W. , W. Cai , B. Hoover , and C. R. Kahn . 2022. “Insulin Action in the Brain: Cell Types, Circuits, and Diseases.” Trends in Neurosciences 45, no. 5: 384–400. 10.1016/j.tins.2022.03.001. 35361499 PMC9035105

[acel70208-bib-0007] Chen, Y. C. , Y. Jiao , Y. Cui , et al. 2014. “Aberrant Brain Functional Connectivity Related to Insulin Resistance in Type 2 Diabetes: A Resting‐State fMRI Study.” Diabetes Care 37, no. 6: 1689–1696. 10.2337/dc13-2127. 24658392

[acel70208-bib-0008] Cole, J. H. 2020. “Multimodality Neuroimaging Brain‐Age in UK Biobank: Relationship to Biomedical, Lifestyle, and Cognitive Factors.” Neurobiology of Aging 92: 34–42. 10.1016/j.neurobiolaging.2020.03.014. 32380363 PMC7280786

[acel70208-bib-0009] Crowley, T. , J. F. Cryan , E. J. Downer , and O. F. O'Leary . 2016. “Inhibiting Neuroinflammation: The Role and Therapeutic Potential of GABA in Neuro‐Immune Interactions.” Brain, Behavior, and Immunity 54: 260–277. 10.1016/j.bbi.2016.02.001. 26851553

[acel70208-bib-0010] Daiello, L. A. , A. Gongvatana , S. Dunsiger , R. A. Cohen , and B. R. Ott . 2015. “Association of Fish Oil Supplement Use With Preservation of Brain Volume and Cognitive Function.” Alzheimer's & Dementia 11, no. 2: 226–235. 10.1016/j.jalz.2014.02.005. PMC4829435 24954371

[acel70208-bib-0011] de Leeuw, F. A. , H. Karamujić‐Čomić , B. M. Tijms , et al. 2021. “Circulating Metabolites Are Associated With Brain Atrophy and White Matter Hyperintensities.” Alzheimer's & Dementia 17, no. 2: 205–214. 10.1002/alz.12180. PMC7984157 32886448

[acel70208-bib-0012] Frick, E. A. , V. Emilsson , T. Jonmundsson , et al. 2024. “Serum Proteomics Reveal APOE‐ε4‐Dependent and APOE‐ε4‐Independent Protein Signatures in Alzheimer's Disease.” Nature Aging 4, no. 10: 1446–1464. 10.1038/s43587-024-00693-1. 39169269 PMC11485263

[acel70208-bib-0013] Fry, A. , T. J. Littlejohns , C. Sudlow , et al. 2017. “Comparison of Sociodemographic and Health‐Related Characteristics of UK Biobank Participants With Those of the General Population.” American Journal of Epidemiology 186, no. 9: 1026–1034. 10.1093/aje/kwx246. 28641372 PMC5860371

[acel70208-bib-0014] Gordon, S. , J. S. Lee , T. M. Scott , et al. 2024. “Metabolites and MRI‐Derived Markers of AD/ADRD Risk in a Puerto Rican Cohort.” Research Square 13: 3941791/v1. 10.21203/rs.3.rs-3941791/v1.

[acel70208-bib-0015] Graff‐Radford, J. , K. X. X. Yong , L. G. Apostolova , et al. 2021. “New Insights Into Atypical Alzheimer's Disease in the Era of Biomarkers.” Lancet Neurology 20, no. 3: 222–234. 10.1016/s1474-4422(20)30440-3. 33609479 PMC8056394

[acel70208-bib-0016] Griffin, J. L. , H. Atherton , J. Shockcor , and L. Atzori . 2011. “Metabolomics as a Tool for Cardiac Research.” Nature Reviews. Cardiology 8, no. 11: 630–643. 10.1038/nrcardio.2011.138. 21931361

[acel70208-bib-0017] Gupta, S. , A. G. Knight , S. Gupta , J. N. Keller , and A. J. Bruce‐Keller . 2012. “Saturated Long‐Chain Fatty Acids Activate Inflammatory Signaling in Astrocytes.” Journal of Neurochemistry 120, no. 6: 1060–1071. 10.1111/j.1471-4159.2012.07660.x. 22248073 PMC3296820

[acel70208-bib-0018] Harshfield, E. L. , and H. S. Markus . 2023. “Association of Baseline Metabolomic Profiles With Incident Stroke and Dementia and With Imaging Markers of Cerebral Small Vessel Disease.” Neurology 101, no. 5: e489–e501. 10.1212/wnl.0000000000207458. 37290969 PMC10401678

[acel70208-bib-0019] Hasin, Y. , M. Seldin , and A. Lusis . 2017. “Multi‐Omics Approaches to Disease.” Genome Biology 18, no. 1: 83. 10.1186/s13059-017-1215-1. 28476144 PMC5418815

[acel70208-bib-0020] Huang, S. Y. , Y. R. Zhang , L. Yang , et al. 2023. “Circulating Metabolites and Risk of Incident Dementia: A Prospective Cohort Study.” Journal of Neurochemistry 167, no. 5: 668–679. 10.1111/jnc.15997. 37908051

[acel70208-bib-0021] Julkunen, H. , A. Cichońska , M. Tiainen , et al. 2023. “Atlas of Plasma NMR Biomarkers for Health and Disease in 118,461 Individuals From the UK Biobank.” Nature Communications 14, no. 1: 604. 10.1038/s41467-023-36231-7. PMC9898515 36737450

[acel70208-bib-0022] Kalmijn, S. , D. Foley , L. White , et al. 2000. “Metabolic Cardiovascular Syndrome and Risk of Dementia in Japanese‐American Elderly Men. The Honolulu‐Asia Aging Study.” Arteriosclerosis, Thrombosis, and Vascular Biology 20, no. 10: 2255–2260. 10.1161/01.atv.20.10.2255. 11031212

[acel70208-bib-0023] Kapogiannis, D. 2015. “Energy Metabolism and the Brain: A Bidirectional Relationship.” Ageing Research Reviews 20: 35–36. 10.1016/j.arr.2015.02.002. 25728594 PMC5456290

[acel70208-bib-0024] Kim, J. , J. Lee , K. Nam , and S. Lee . 2023. “Investigation of Genetic Variants and Causal Biomarkers Associated With Brain Aging.” Scientific Reports 13, no. 1: 1526. 10.1038/s41598-023-27903-x. 36707530 PMC9883521

[acel70208-bib-0025] Lian, J. , and V. Vardhanabhuti . 2024. “Metabolic Biomarkers Using Nuclear Magnetic Resonance Metabolomics Assay for the Prediction of Aging‐Related Disease Risk and Mortality: A Prospective, Longitudinal, Observational, Cohort Study Based on the UK Biobank.” Geroscience 46, no. 2: 1515–1526. 10.1007/s11357-023-00918-y. 37648937 PMC10828466

[acel70208-bib-0026] Liu, C. C. , C. C. Liu , T. Kanekiyo , H. Xu , and G. Bu . 2013. “Apolipoprotein E and Alzheimer Disease: Risk, Mechanisms and Therapy.” Nature Reviews. Neurology 9, no. 2: 106–118. 10.1038/nrneurol.2012.263. 23296339 PMC3726719

[acel70208-bib-0027] Maestú, F. , W. de Haan , M. A. Busche , and J. DeFelipe . 2021. “Neuronal Excitation/Inhibition Imbalance: Core Element of a Translational Perspective on Alzheimer Pathophysiology.” Ageing Research Reviews 69: 101372. 10.1016/j.arr.2021.101372. 34029743

[acel70208-bib-0028] Malek‐Ahmadi, M. , Y. Su , V. Ghisays , et al. 2023. “Plasma NfL Is Associated With the APOE ε4 Allele, Brain Imaging Measurements of Neurodegeneration, and Lower Recall Memory Scores in Cognitively Unimpaired Late‐Middle‐Aged and Older Adults.” Alzheimer's Research & Therapy 15, no. 1: 74. 10.1186/s13195-023-01221-w. PMC10084600 37038190

[acel70208-bib-0029] Meneilly, G. S. , and D. M. Tessier . 2016. “Diabetes, Dementia and Hypoglycemia.” Canadian Journal of Diabetes 40, no. 1: 73–76. 10.1016/j.jcjd.2015.09.006. 26778684

[acel70208-bib-0030] Montagne, A. , A. M. Nikolakopoulou , M. T. Huuskonen , et al. 2021. “APOE4 Accelerates Advanced‐Stage Vascular and Neurodegenerative Disorder in Old Alzheimer's Mice via Cyclophilin A Independently of Amyloid‐β.” Nature Aging 1, no. 6: 506–520. 10.1038/s43587-021-00073-z. 35291561 PMC8920485

[acel70208-bib-0031] Nakamura, T. , T. Kawarabayashi , T. Ueda , et al. 2023. “Plasma ApoE4 Levels Are Lower Than ApoE2 and ApoE3 Levels, and not Associated With Plasma Aβ40/42 Ratio as a Biomarker of Amyloid‐β Amyloidosis in Alzheimer's Disease.” Journal of Alzheimer's Disease 93, no. 1: 333–348. 10.3233/jad-220996. 36970894

[acel70208-bib-0032] Navab, M. , G. M. Ananthramaiah , S. T. Reddy , et al. 2005. “The Double Jeopardy of HDL.” Annals of Medicine 37, no. 3: 173–178. 10.1080/07853890510007322. 16019715

[acel70208-bib-0033] Plötz, T. , B. Krümmel , A. Laporte , et al. 2017. “The Monounsaturated Fatty Acid Oleate Is the Major Physiological Toxic Free Fatty Acid for Human Beta Cells.” Nutrition & Diabetes 7, no. 12: 305. 10.1038/s41387-017-0005-x. 29269872 PMC5865546

[acel70208-bib-0034] Qiang, Y. X. , J. You , X. Y. He , et al. 2024. “Plasma Metabolic Profiles Predict Future Dementia and Dementia Subtypes: A Prospective Analysis of 274,160 Participants.” Alzheimer's Research & Therapy 16, no. 1: 16. 10.1186/s13195-023-01379-3. PMC10802055 38254212

[acel70208-bib-0035] Rader, D. J. , and G. K. Hovingh . 2014. “HDL and Cardiovascular Disease.” Lancet 384, no. 9943: 618–625. 10.1016/s0140-6736(14)61217-4. 25131981

[acel70208-bib-0036] Reger, M. A. , S. T. Henderson , C. Hale , et al. 2004. “Effects of Beta‐Hydroxybutyrate on Cognition in Memory‐Impaired Adults.” Neurobiology of Aging 25, no. 3: 311–314. 10.1016/s0197-4580(03)00087-3. 15123336

[acel70208-bib-0037] Reiman, E. M. , K. Chen , G. E. Alexander , et al. 2004. “Functional Brain Abnormalities in Young Adults at Genetic Risk for Late‐Onset Alzheimer's Dementia.” Proceedings of the National Academy of Sciences of the United States of America 101, no. 1: 284–289. 10.1073/pnas.2635903100. 14688411 PMC314177

[acel70208-bib-0038] Rusek, M. , R. Pluta , M. Ułamek‐Kozioł , and S. J. Czuczwar . 2019. “Ketogenic Diet in Alzheimer's Disease.” International Journal of Molecular Sciences 20, no. 16: 3892. 10.3390/ijms20163892. 31405021 PMC6720297

[acel70208-bib-0039] Schmidt, M. I. , B. B. Duncan , A. R. Sharrett , et al. 1999. “Markers of Inflammation and Prediction of Diabetes Mellitus in Adults (Atherosclerosis Risk in Communities Study): A Cohort Study.” Lancet 353, no. 9165: 1649–1652. 10.1016/s0140-6736(99)01046-6. 10335783

[acel70208-bib-0040] Shippy, D. C. , C. Wilhelm , P. A. Viharkumar , T. J. Raife , and T. K. Ulland . 2020. “β‐Hydroxybutyrate Inhibits Inflammasome Activation to Attenuate Alzheimer's Disease Pathology.” Journal of Neuroinflammation 17, no. 1: 280. 10.1186/s12974-020-01948-5. 32958021 PMC7507727

[acel70208-bib-0041] Sliz, E. , J. Shin , S. Ahmad , et al. 2022. “Circulating Metabolome and White Matter Hyperintensities in Women and Men.” Circulation 145, no. 14: 1040–1052. 10.1161/circulationaha.121.056892. 35050683 PMC9645366

[acel70208-bib-0042] Sliz, E. , J. Shin , C. Syme , et al. 2020. “Thickness of the Cerebral Cortex Shows Positive Association With Blood Levels of Triacylglycerols Carrying 18‐Carbon Fatty Acids.” Communications Biology 3, no. 1: 456. 10.1038/s42003-020-01189-5. 32820227 PMC7441395

[acel70208-bib-0043] Sliz, E. , J. Shin , C. Syme , et al. 2021. “A Variant Near DHCR24 Associates With Microstructural Properties of White Matter and Peripheral Lipid Metabolism in Adolescents.” Molecular Psychiatry 26, no. 8: 3795–3805. 10.1038/s41380-019-0640-9. 31900429 PMC7332371

[acel70208-bib-0044] Sommerfield, A. J. , I. J. Deary , and B. M. Frier . 2004. “Acute Hyperglycemia Alters Mood State and Impairs Cognitive Performance in People With Type 2 Diabetes.” Diabetes Care 27, no. 10: 2335–2340. 10.2337/diacare.27.10.2335. 15451897

[acel70208-bib-0045] Sun, Y. , Y. Guo , H. Q. Li , et al. 2023. “Associations of Circulating Metabolites With Cerebral White Matter Hyperintensities.” Journal of Neurochemistry 166, no. 2: 414–423. 10.1111/jnc.15845. 37222503

[acel70208-bib-0046] Tian, Y. E. , V. Cropley , A. B. Maier , N. T. Lautenschlager , M. Breakspear , and A. Zalesky . 2023. “Heterogeneous Aging Across Multiple Organ Systems and Prediction of Chronic Disease and Mortality.” Nature Medicine 29, no. 5: 1221–1231. 10.1038/s41591-023-02296-6. 37024597

[acel70208-bib-0047] Turney, I. C. , A. G. Chesebro , M. A. Rentería , et al. 2020. “APOE ε4 and Resting‐State Functional Connectivity in Racially/Ethnically Diverse Older Adults.” Alzheimer's & Dementia: Diagnosis, Assessment & Disease Monitoring 12, no. 1: e12094. 10.1002/dad2.12094. PMC7508460 32999914

[acel70208-bib-0048] Tynkkynen, J. , V. Chouraki , S. J. van der Lee , et al. 2018. “Association of Branched‐Chain Amino Acids and Other Circulating Metabolites With Risk of Incident Dementia and Alzheimer's Disease: A Prospective Study in Eight Cohorts.” Alzheimer's & Dementia 14, no. 6: 723–733. 10.1016/j.jalz.2018.01.003. PMC6082422 29519576

[acel70208-bib-0049] van der Lee, S. J. , C. E. Teunissen , R. Pool , et al. 2018. “Circulating Metabolites and General Cognitive Ability and Dementia: Evidence From 11 Cohort Studies.” Alzheimer's & Dementia 14, no. 6: 707–722. 10.1016/j.jalz.2017.11.012. 29316447

[acel70208-bib-0050] Wennberg, A. M. , A. P. Spira , C. Pettigrew , et al. 2016. “Blood Glucose Levels and Cortical Thinning in Cognitively Normal, Middle‐Aged Adults.” Journal of the Neurological Sciences 365: 89–95. 10.1016/j.jns.2016.04.017. 27206882 PMC4876973

[acel70208-bib-0051] Zhang, T. , X. Han , X. Zhang , Z. Chen , Y. Mi , and X. Gou . 2020. “Dietary Fatty Acid Factors in Alzheimer's Disease: A Review.” Journal of Alzheimer's Disease 78, no. 3: 887–904. 10.3233/jad-200558. 33074226

[acel70208-bib-0052] Zhang, Y. , M. Xiao , G. Niu , and H. Tan . 2005. “Mechanisms of Oleic Acid Deterioration in Insulin Secretion: Role in the Pathogenesis of Type 2 Diabetes.” Life Sciences 77, no. 17: 2071–2081. 10.1016/j.lfs.2004.12.047. 15935394

