# Supplementary material for: Association Between Plasma Metabolomic Profile and Machine Learning‐Based Brain Age
Source: Aging Cell. 2025 Sep 1;24(10):e70208. doi: 10.1111/acel.70208 (PMC12507404; doi:10.1111/acel.70208)
Supplement: Supplementary file 1 — Table S1: Self‐reported health variable codes used for exclusion criteria on initial population. Table S2: Missing number for brain imaging‐derived phenotypes. Table S3: Coefficients for 285 IDPs that significantly contribute to brain age estimation in the LASSO regression without feature selection. Table S4: Hyperparameter spaces of 9 models in Bayesian optimization. Table S5: The chosen hyperparameters of 9 models. Table S6: Model evaluation of 9 models in the validation set and testing set. Table S7: β coefficients and 95% confidence intervals (CIs) for the association between metabolites and brain age: results from linear regression models. Table S8: β coefficients and 95% confidence intervals (CIs) for the association between metabolites and brain age gap (BAG): results from linear regression models. Table S9: β coefficients and 95% confidence intervals (CIs) for the association between metabolites and brain age among non‐APOE ε4 carriers: results from linear regression models. Table S10: β coefficients and 95% confidence intervals (CIs) for the association between metabolites and brain age gap (BAG) among non‐APOE ε4 carriers: results from linear regression models. Table S11: β coefficients and 95% confidence intervals (CIs) for the association between metabolites and brain age among APOE ε4 carriers: results from linear regression models. Table S12: β coefficients and 95% confidence intervals (CIs) for the association between metabolites and brain age gap (BAG) among APOE ε4 carriers: results from linear regression models. Table S13:. The interaction effect of metabolites with APOE ε4 status on brain age gap (BAG). Table S14: β coefficients and 95% confidence intervals (CIs) for the association between metabolites and brain age after multiple imputation of covariates: results from linear regression models. Table S15: β coefficients and 95% confidence intervals (CIs) for the association between metabolites and brain age gap (BAG) after multiple imputation of c [file ACEL-24-e70208-s001.docx]

**Association between plasma metabolomic profile and machine learning-based brain age**

**Table S1.** Self-reported health variable codes used for exclusion criteria on initial population

**Table S2.** Missing number for brain imaging-derived phenotypes

**Table S3.** Coefficients for 285 IDPs that significantly contribute to brain age estimation in the LASSO regression without feature selection

**Table S4**. Hyperparameter spaces of 9 models in Bayesian optimization.

**Table S5.** The chosen hyperparameters of 9 models.

**Table S6.** Model evaluation of 9 models in the validation set and testing set.

**Table S7.** β coefficients and 95% confidence intervals (CIs) for the association between metabolites and brain age: results from linear regression models

**Table S8.** β coefficients and 95% confidence intervals (CIs) for the association between metabolites and brain age gap (BAG): results from linear regression models

**Table S9.** β coefficients and 95% confidence intervals (CIs) for the association between metabolites and brain age among non-*APOE* ε4 carriers: results from linear regression models

**Table S10.** β coefficients and 95% confidence intervals (CIs) for the association between metabolites and brain age gap (BAG) among non-*APOE* ε4 carriers: results from linear regression models

**Table S11.** β coefficients and 95% confidence intervals (CIs) for the association between metabolites and brain age among *APOE* ε4 carriers: results from linear regression models

**Table S12.** β coefficients and 95% confidence intervals (CIs) for the association between metabolites and brain age gap (BAG) among *APOE* ε4 carriers: results from linear regression models

**Table S13.** The interaction effect of metabolites with *APOE* ε4 status on brain age gap (BAG)

**Table S14.** β coefficients and 95% confidence intervals (CIs) for the association between metabolites and brain age after multiple imputation of covariates: results from linear regression models

**Table S15.** β coefficients and 95% confidence intervals (CIs) for the association between metabolites and brain age gap (BAG) after multiple imputation of covariates: results from linear regression models

**Table S16.** β coefficients and 95% confidence intervals (CIs) for the association between metabolites and brain age gap (BAG) within eight other candidate machine learning models: results from linear regression models

**Figure S1.** 249 individual metabolites in relation to (a) brain age and (b) brain age gap.

| **Table S1.** Self-reported health variable codes used for exclusion criteria on initial population | |
| --- | --- |
| **Self-reported illness** | **Code (Field ID 20002 and 20003)** |
| Dementia or Alzheimer’s disease | 1263 |
| Parkinson’s disease | 1262 |
| Chronic degenerative neurological | 1258 |
| Guillain-Barré syndrome | 1256 |
| Multiple Sclerosis | 1261 |
| Other demyelinating disease | 1397 |
| Stroke or ischemic stroke | 1081 |
| Brain cancer | 1032 |
| Brain hemorrhage | 1491 |
| Brain/intracranial abscess | 1245 |
| Cerebral aneurysm | 1425 |
| Cerebral palsy | 1433 |
| Encephalitis | 1246 |
| Epilepsy | 1264 |
| Head injury | 1266 |
| Infections of the nervous system | 1244 |
| Ischemic stroke | 1583 |
| Meningeal cancer | 1031 |
| Meningioma (benign) | 1659 |
| Meningitis | 1247 |
| Motor Neuron Disease | 1259 |
| Neurological injury/trauma | 1240 |
| Spina bifida | 1524 |
| Subdural hematoma | 1083 |
| Subarachnoid hemorrhage | 1086 |
| Transient ischemic attack | 1082 |

**Table S2.** Missing number for brain imaging-derived phenotypes.

| **Phenotype** | **Field ID** | **Modality** | **Missing** |
| --- | --- | --- | --- |
| Volumetric scaling from T1 head image to standard space | 25000 | T1-weighted MRI | 3 |
| Volume of peripheral cortical grey matter (normalized for head size) | 25001 | T1-weighted MRI | 3 |
| Volume of peripheral cortical grey matter | 25002 | T1-weighted MRI | 3 |
| Volume of ventricular cerebrospinal fluid (normalized for head size) | 25003 | T1-weighted MRI | 3 |
| Volume of ventricular cerebrospinal fluid | 25004 | T1-weighted MRI | 3 |
| Volume of grey matter (normalized for head size) | 25005 | T1-weighted MRI | 3 |
| Volume of grey matter | 25006 | T1-weighted MRI | 3 |
| Volume of white matter (normalized for head size) | 25007 | T1-weighted MRI | 3 |
| Volume of white matter | 25008 | T1-weighted MRI | 3 |
| Volume of brain, grey+white matter (normalized for head size) | 25009 | T1-weighted MRI | 3 |
| Volume of brain, grey+white matter | 25010 | T1-weighted MRI | 3 |
| Volume of thalamus (left) | 25011 | T1-weighted MRI | 20 |
| Volume of thalamus (right) | 25012 | T1-weighted MRI | 20 |
| Volume of caudate (left) | 25013 | T1-weighted MRI | 20 |
| Volume of caudate (right) | 25014 | T1-weighted MRI | 20 |
| Volume of putamen (left) | 25015 | T1-weighted MRI | 20 |
| Volume of putamen (right) | 25016 | T1-weighted MRI | 20 |
| Volume of pallidum (left) | 25017 | T1-weighted MRI | 20 |
| Volume of pallidum (right) | 25018 | T1-weighted MRI | 20 |
| Volume of hippocampus (left) | 25019 | T1-weighted MRI | 20 |
| Volume of hippocampus (right) | 25020 | T1-weighted MRI | 20 |
| Volume of amygdala (left) | 25021 | T1-weighted MRI | 20 |
| Volume of amygdala (right) | 25022 | T1-weighted MRI | 20 |
| Volume of accumbens (left) | 25023 | T1-weighted MRI | 20 |
| Volume of accumbens (right) | 25024 | T1-weighted MRI | 20 |
| Volume of brain stem + 4th ventricle | 25025 | T1-weighted MRI | 20 |
| Volume of grey matter in Frontal Pole (left) | 25782 | T1-weighted MRI | 8 |
| Volume of grey matter in Frontal Pole (right) | 25783 | T1-weighted MRI | 8 |
| Volume of grey matter in Insular Cortex (left) | 25784 | T1-weighted MRI | 8 |
| Volume of grey matter in Insular Cortex (right) | 25785 | T1-weighted MRI | 8 |
| Volume of grey matter in Superior Frontal Gyrus (left) | 25786 | T1-weighted MRI | 8 |
| Volume of grey matter in Superior Frontal Gyrus (right) | 25787 | T1-weighted MRI | 8 |
| Volume of grey matter in Middle Frontal Gyrus (left) | 25788 | T1-weighted MRI | 8 |
| Volume of grey matter in Middle Frontal Gyrus (right) | 25789 | T1-weighted MRI | 8 |
| Volume of grey matter in Inferior Frontal Gyrus, pars triangularis (left) | 25790 | T1-weighted MRI | 8 |
| Volume of grey matter in Inferior Frontal Gyrus, pars triangularis (right) | 25791 | T1-weighted MRI | 8 |
| Volume of grey matter in Inferior Frontal Gyrus, pars opercularis (left) | 25792 | T1-weighted MRI | 8 |
| Volume of grey matter in Inferior Frontal Gyrus, pars opercularis (right) | 25793 | T1-weighted MRI | 8 |
| Volume of grey matter in Precentral Gyrus (left) | 25794 | T1-weighted MRI | 8 |
| Volume of grey matter in Precentral Gyrus (right) | 25795 | T1-weighted MRI | 8 |
| Volume of grey matter in Temporal Pole (left) | 25796 | T1-weighted MRI | 8 |
| Volume of grey matter in Temporal Pole (right) | 25797 | T1-weighted MRI | 8 |
| Volume of grey matter in Superior Temporal Gyrus, anterior division (left) | 25798 | T1-weighted MRI | 8 |
| Volume of grey matter in Superior Temporal Gyrus, anterior division (right) | 25799 | T1-weighted MRI | 8 |
| Volume of grey matter in Superior Temporal Gyrus, posterior division (left) | 25800 | T1-weighted MRI | 8 |
| Volume of grey matter in Superior Temporal Gyrus, posterior division (right) | 25801 | T1-weighted MRI | 8 |
| Volume of grey matter in Middle Temporal Gyrus, anterior division (left) | 25802 | T1-weighted MRI | 8 |
| Volume of grey matter in Middle Temporal Gyrus, anterior division (right) | 25803 | T1-weighted MRI | 8 |
| Volume of grey matter in Middle Temporal Gyrus, posterior division (left) | 25804 | T1-weighted MRI | 8 |
| Volume of grey matter in Middle Temporal Gyrus, posterior division (right) | 25805 | T1-weighted MRI | 8 |
| Volume of grey matter in Middle Temporal Gyrus, temporooccipital part (left) | 25806 | T1-weighted MRI | 8 |
| Volume of grey matter in Middle Temporal Gyrus, temporooccipital part (right) | 25807 | T1-weighted MRI | 8 |
| Volume of grey matter in Inferior Temporal Gyrus, anterior division (left) | 25808 | T1-weighted MRI | 8 |
| Volume of grey matter in Inferior Temporal Gyrus, anterior division (right) | 25809 | T1-weighted MRI | 8 |
| Volume of grey matter in Inferior Temporal Gyrus, posterior division (left) | 25810 | T1-weighted MRI | 8 |
| Volume of grey matter in Inferior Temporal Gyrus, posterior division (right) | 25811 | T1-weighted MRI | 8 |
| Volume of grey matter in Inferior Temporal Gyrus, temporooccipital part (left) | 25812 | T1-weighted MRI | 8 |
| Volume of grey matter in Inferior Temporal Gyrus, temporooccipital part (right) | 25813 | T1-weighted MRI | 8 |
| Volume of grey matter in Postcentral Gyrus (left) | 25814 | T1-weighted MRI | 8 |
| Volume of grey matter in Postcentral Gyrus (right) | 25815 | T1-weighted MRI | 8 |
| Volume of grey matter in Superior Parietal Lobule (left) | 25816 | T1-weighted MRI | 8 |
| Volume of grey matter in Superior Parietal Lobule (right) | 25817 | T1-weighted MRI | 8 |
| Volume of grey matter in Supramarginal Gyrus, anterior division (left) | 25818 | T1-weighted MRI | 8 |
| Volume of grey matter in Supramarginal Gyrus, anterior division (right) | 25819 | T1-weighted MRI | 8 |
| Volume of grey matter in Supramarginal Gyrus, posterior division (left) | 25820 | T1-weighted MRI | 8 |
| Volume of grey matter in Supramarginal Gyrus, posterior division (right) | 25821 | T1-weighted MRI | 8 |
| Volume of grey matter in Angular Gyrus (left) | 25822 | T1-weighted MRI | 8 |
| Volume of grey matter in Angular Gyrus (right) | 25823 | T1-weighted MRI | 8 |
| Volume of grey matter in Lateral Occipital Cortex, superior division (left) | 25824 | T1-weighted MRI | 8 |
| Volume of grey matter in Lateral Occipital Cortex, superior division (right) | 25825 | T1-weighted MRI | 8 |
| Volume of grey matter in Lateral Occipital Cortex, inferior division (left) | 25826 | T1-weighted MRI | 8 |
| Volume of grey matter in Lateral Occipital Cortex, inferior division (right) | 25827 | T1-weighted MRI | 8 |
| Volume of grey matter in Intracalcarine Cortex (left) | 25828 | T1-weighted MRI | 8 |
| Volume of grey matter in Intracalcarine Cortex (right) | 25829 | T1-weighted MRI | 8 |
| Volume of grey matter in Frontal Medial Cortex (left) | 25830 | T1-weighted MRI | 8 |
| Volume of grey matter in Frontal Medial Cortex (right) | 25831 | T1-weighted MRI | 8 |
| Volume of grey matter in Juxtapositional Lobule Cortex (formerly Supplementary Motor Cortex) (left) | 25832 | T1-weighted MRI | 8 |
| Volume of grey matter in Juxtapositional Lobule Cortex (formerly Supplementary Motor Cortex) (right) | 25833 | T1-weighted MRI | 8 |
| Volume of grey matter in Subcallosal Cortex (left) | 25834 | T1-weighted MRI | 8 |
| Volume of grey matter in Subcallosal Cortex (right) | 25835 | T1-weighted MRI | 8 |
| Volume of grey matter in Paracingulate Gyrus (left) | 25836 | T1-weighted MRI | 8 |
| Volume of grey matter in Paracingulate Gyrus (right) | 25837 | T1-weighted MRI | 8 |
| Volume of grey matter in Cingulate Gyrus, anterior division (left) | 25838 | T1-weighted MRI | 8 |
| Volume of grey matter in Cingulate Gyrus, anterior division (right) | 25839 | T1-weighted MRI | 8 |
| Volume of grey matter in Cingulate Gyrus, posterior division (left) | 25840 | T1-weighted MRI | 8 |
| Volume of grey matter in Cingulate Gyrus, posterior division (right) | 25841 | T1-weighted MRI | 8 |
| Volume of grey matter in Precuneous Cortex (left) | 25842 | T1-weighted MRI | 8 |
| Volume of grey matter in Precuneous Cortex (right) | 25843 | T1-weighted MRI | 8 |
| Volume of grey matter in Cuneal Cortex (left) | 25844 | T1-weighted MRI | 8 |
| Volume of grey matter in Cuneal Cortex (right) | 25845 | T1-weighted MRI | 8 |
| Volume of grey matter in Frontal Orbital Cortex (left) | 25846 | T1-weighted MRI | 8 |
| Volume of grey matter in Frontal Orbital Cortex (right) | 25847 | T1-weighted MRI | 8 |
| Volume of grey matter in Parahippocampal Gyrus, anterior division (left) | 25848 | T1-weighted MRI | 8 |
| Volume of grey matter in Parahippocampal Gyrus, anterior division (right) | 25849 | T1-weighted MRI | 8 |
| Volume of grey matter in Parahippocampal Gyrus, posterior division (left) | 25850 | T1-weighted MRI | 8 |
| Volume of grey matter in Parahippocampal Gyrus, posterior division (right) | 25851 | T1-weighted MRI | 8 |
| Volume of grey matter in Lingual Gyrus (left) | 25852 | T1-weighted MRI | 8 |
| Volume of grey matter in Lingual Gyrus (right) | 25853 | T1-weighted MRI | 8 |
| Volume of grey matter in Temporal Fusiform Cortex, anterior division (left) | 25854 | T1-weighted MRI | 8 |
| Volume of grey matter in Temporal Fusiform Cortex, anterior division (right) | 25855 | T1-weighted MRI | 8 |
| Volume of grey matter in Temporal Fusiform Cortex, posterior division (left) | 25856 | T1-weighted MRI | 8 |
| Volume of grey matter in Temporal Fusiform Cortex, posterior division (right) | 25857 | T1-weighted MRI | 8 |
| Volume of grey matter in Temporal Occipital Fusiform Cortex (left) | 25858 | T1-weighted MRI | 8 |
| Volume of grey matter in Temporal Occipital Fusiform Cortex (right) | 25859 | T1-weighted MRI | 8 |
| Volume of grey matter in Occipital Fusiform Gyrus (left) | 25860 | T1-weighted MRI | 8 |
| Volume of grey matter in Occipital Fusiform Gyrus (right) | 25861 | T1-weighted MRI | 8 |
| Volume of grey matter in Frontal Operculum Cortex (left) | 25862 | T1-weighted MRI | 8 |
| Volume of grey matter in Frontal Operculum Cortex (right) | 25863 | T1-weighted MRI | 8 |
| Volume of grey matter in Central Opercular Cortex (left) | 25864 | T1-weighted MRI | 8 |
| Volume of grey matter in Central Opercular Cortex (right) | 25865 | T1-weighted MRI | 8 |
| Volume of grey matter in Parietal Operculum Cortex (left) | 25866 | T1-weighted MRI | 8 |
| Volume of grey matter in Parietal Operculum Cortex (right) | 25867 | T1-weighted MRI | 8 |
| Volume of grey matter in Planum Polare (left) | 25868 | T1-weighted MRI | 8 |
| Volume of grey matter in Planum Polare (right) | 25869 | T1-weighted MRI | 8 |
| Volume of grey matter in Heschl's Gyrus (includes H1 and H2) (left) | 25870 | T1-weighted MRI | 8 |
| Volume of grey matter in Heschl's Gyrus (includes H1 and H2) (right) | 25871 | T1-weighted MRI | 8 |
| Volume of grey matter in Planum Temporale (left) | 25872 | T1-weighted MRI | 8 |
| Volume of grey matter in Planum Temporale (right) | 25873 | T1-weighted MRI | 8 |
| Volume of grey matter in Supracalcarine Cortex (left) | 25874 | T1-weighted MRI | 8 |
| Volume of grey matter in Supracalcarine Cortex (right) | 25875 | T1-weighted MRI | 8 |
| Volume of grey matter in Occipital Pole (left) | 25876 | T1-weighted MRI | 8 |
| Volume of grey matter in Occipital Pole (right) | 25877 | T1-weighted MRI | 8 |
| Volume of grey matter in Thalamus (left) | 25878 | T1-weighted MRI | 8 |
| Volume of grey matter in Thalamus (right) | 25879 | T1-weighted MRI | 8 |
| Volume of grey matter in Caudate (left) | 25880 | T1-weighted MRI | 8 |
| Volume of grey matter in Caudate (right) | 25881 | T1-weighted MRI | 8 |
| Volume of grey matter in Putamen (left) | 25882 | T1-weighted MRI | 8 |
| Volume of grey matter in Putamen (right) | 25883 | T1-weighted MRI | 8 |
| Volume of grey matter in Pallidum (left) | 25884 | T1-weighted MRI | 8 |
| Volume of grey matter in Pallidum (right) | 25885 | T1-weighted MRI | 8 |
| Volume of grey matter in Hippocampus (left) | 25886 | T1-weighted MRI | 8 |
| Volume of grey matter in Hippocampus (right) | 25887 | T1-weighted MRI | 8 |
| Volume of grey matter in Amygdala (left) | 25888 | T1-weighted MRI | 8 |
| Volume of grey matter in Amygdala (right) | 25889 | T1-weighted MRI | 8 |
| Volume of grey matter in Ventral Striatum (left) | 25890 | T1-weighted MRI | 8 |
| Volume of grey matter in Ventral Striatum (right) | 25891 | T1-weighted MRI | 8 |
| Volume of grey matter in Brain-Stem | 25892 | T1-weighted MRI | 8 |
| Volume of grey matter in I-IV Cerebellum (left) | 25893 | T1-weighted MRI | 8 |
| Volume of grey matter in I-IV Cerebellum (right) | 25894 | T1-weighted MRI | 8 |
| Volume of grey matter in V Cerebellum (left) | 25895 | T1-weighted MRI | 8 |
| Volume of grey matter in V Cerebellum (right) | 25896 | T1-weighted MRI | 8 |
| Volume of grey matter in VI Cerebellum (left) | 25897 | T1-weighted MRI | 8 |
| Volume of grey matter in VI Cerebellum (vermis) | 25898 | T1-weighted MRI | 8 |
| Volume of grey matter in VI Cerebellum (right) | 25899 | T1-weighted MRI | 8 |
| Volume of grey matter in Crus I Cerebellum (left) | 25900 | T1-weighted MRI | 8 |
| Volume of grey matter in Crus I Cerebellum (vermis) | 25901 | T1-weighted MRI | 8 |
| Volume of grey matter in Crus I Cerebellum (right) | 25902 | T1-weighted MRI | 8 |
| Volume of grey matter in Crus II Cerebellum (left) | 25903 | T1-weighted MRI | 8 |
| Volume of grey matter in Crus II Cerebellum (vermis) | 25904 | T1-weighted MRI | 8 |
| Volume of grey matter in Crus II Cerebellum (right) | 25905 | T1-weighted MRI | 8 |
| Volume of grey matter in VIIb Cerebellum (left) | 25906 | T1-weighted MRI | 8 |
| Volume of grey matter in VIIb Cerebellum (vermis) | 25907 | T1-weighted MRI | 8 |
| Volume of grey matter in VIIb Cerebellum (right) | 25908 | T1-weighted MRI | 8 |
| Volume of grey matter in VIIIa Cerebellum (left) | 25909 | T1-weighted MRI | 8 |
| Volume of grey matter in VIIIa Cerebellum (vermis) | 25910 | T1-weighted MRI | 8 |
| Volume of grey matter in VIIIa Cerebellum (right) | 25911 | T1-weighted MRI | 8 |
| Volume of grey matter in VIIIb Cerebellum (left) | 25912 | T1-weighted MRI | 8 |
| Volume of grey matter in VIIIb Cerebellum (vermis) | 25913 | T1-weighted MRI | 8 |
| Volume of grey matter in VIIIb Cerebellum (right) | 25914 | T1-weighted MRI | 8 |
| Volume of grey matter in IX Cerebellum (left) | 25915 | T1-weighted MRI | 8 |
| Volume of grey matter in IX Cerebellum (vermis) | 25916 | T1-weighted MRI | 8 |
| Volume of grey matter in IX Cerebellum (right) | 25917 | T1-weighted MRI | 8 |
| Volume of grey matter in X Cerebellum (left) | 25918 | T1-weighted MRI | 8 |
| Volume of grey matter in X Cerebellum (vermis) | 25919 | T1-weighted MRI | 8 |
| Volume of grey matter in X Cerebellum (right) | 25920 | T1-weighted MRI | 8 |
| Total volume of white matter hyperintensities (from T1 and T2_FLAIR images) | 25781 | T2-FLAIR | 1372 |
| Median T2star in thalamus (left) | 25026 | T2∗ | 4065 |
| Median T2star in thalamus (right) | 25027 | T2∗ | 4065 |
| Median T2star in caudate (left) | 25028 | T2∗ | 4065 |
| Median T2star in caudate (right) | 25029 | T2∗ | 4065 |
| Median T2star in putamen (left) | 25030 | T2∗ | 4065 |
| Median T2star in putamen (right) | 25031 | T2∗ | 4065 |
| Median T2star in pallidum (left) | 25032 | T2∗ | 4065 |
| Median T2star in pallidum (right) | 25033 | T2∗ | 4065 |
| Median T2star in hippocampus (left) | 25034 | T2∗ | 4065 |
| Median T2star in hippocampus (right) | 25035 | T2∗ | 4065 |
| Median T2star in amygdala (left) | 25036 | T2∗ | 4065 |
| Median T2star in amygdala (right) | 25037 | T2∗ | 4065 |
| Median T2star in accumbens (left) | 25038 | T2∗ | 4065 |
| Median T2star in accumbens (right) | 25039 | T2∗ | 4065 |
| Mean FA in middle cerebellar peduncle on FA skeleton | 25056 | diffusion-MRI | 2289 |
| Mean FA in pontine crossing tract on FA skeleton | 25057 | diffusion-MRI | 2289 |
| Mean FA in genu of corpus callosum on FA skeleton | 25058 | diffusion-MRI | 2289 |
| Mean FA in body of corpus callosum on FA skeleton | 25059 | diffusion-MRI | 2289 |
| Mean FA in splenium of corpus callosum on FA skeleton | 25060 | diffusion-MRI | 2289 |
| Mean FA in fornix on FA skeleton | 25061 | diffusion-MRI | 2289 |
| Mean FA in corticospinal tract on FA skeleton (right) | 25062 | diffusion-MRI | 2289 |
| Mean FA in corticospinal tract on FA skeleton (left) | 25063 | diffusion-MRI | 2289 |
| Mean FA in medial lemniscus on FA skeleton (right) | 25064 | diffusion-MRI | 2289 |
| Mean FA in medial lemniscus on FA skeleton (left) | 25065 | diffusion-MRI | 2289 |
| Mean FA in inferior cerebellar peduncle on FA skeleton (right) | 25066 | diffusion-MRI | 2289 |
| Mean FA in inferior cerebellar peduncle on FA skeleton (left) | 25067 | diffusion-MRI | 2289 |
| Mean FA in superior cerebellar peduncle on FA skeleton (right) | 25068 | diffusion-MRI | 2289 |
| Mean FA in superior cerebellar peduncle on FA skeleton (left) | 25069 | diffusion-MRI | 2289 |
| Mean FA in cerebral peduncle on FA skeleton (right) | 25070 | diffusion-MRI | 2289 |
| Mean FA in cerebral peduncle on FA skeleton (left) | 25071 | diffusion-MRI | 2289 |
| Mean FA in anterior limb of internal capsule on FA skeleton (right) | 25072 | diffusion-MRI | 2289 |
| Mean FA in anterior limb of internal capsule on FA skeleton (left) | 25073 | diffusion-MRI | 2289 |
| Mean FA in posterior limb of internal capsule on FA skeleton (right) | 25074 | diffusion-MRI | 2289 |
| Mean FA in posterior limb of internal capsule on FA skeleton (left) | 25075 | diffusion-MRI | 2289 |
| Mean FA in retrolenticular part of internal capsule on FA skeleton (right) | 25076 | diffusion-MRI | 2289 |
| Mean FA in retrolenticular part of internal capsule on FA skeleton (left) | 25077 | diffusion-MRI | 2289 |
| Mean FA in anterior corona radiata on FA skeleton (right) | 25078 | diffusion-MRI | 2289 |
| Mean FA in anterior corona radiata on FA skeleton (left) | 25079 | diffusion-MRI | 2289 |
| Mean FA in superior corona radiata on FA skeleton (right) | 25080 | diffusion-MRI | 2289 |
| Mean FA in superior corona radiata on FA skeleton (left) | 25081 | diffusion-MRI | 2289 |
| Mean FA in posterior corona radiata on FA skeleton (right) | 25082 | diffusion-MRI | 2289 |
| Mean FA in posterior corona radiata on FA skeleton (left) | 25083 | diffusion-MRI | 2289 |
| Mean FA in posterior thalamic radiation on FA skeleton (right) | 25084 | diffusion-MRI | 2289 |
| Mean FA in posterior thalamic radiation on FA skeleton (left) | 25085 | diffusion-MRI | 2289 |
| Mean FA in sagittal stratum on FA skeleton (right) | 25086 | diffusion-MRI | 2289 |
| Mean FA in sagittal stratum on FA skeleton (left) | 25087 | diffusion-MRI | 2289 |
| Mean FA in external capsule on FA skeleton (right) | 25088 | diffusion-MRI | 2289 |
| Mean FA in external capsule on FA skeleton (left) | 25089 | diffusion-MRI | 2289 |
| Mean FA in cingulum cingulate gyrus on FA skeleton (right) | 25090 | diffusion-MRI | 2289 |
| Mean FA in cingulum cingulate gyrus on FA skeleton (left) | 25091 | diffusion-MRI | 2289 |
| Mean FA in cingulum hippocampus on FA skeleton (right) | 25092 | diffusion-MRI | 2289 |
| Mean FA in cingulum hippocampus on FA skeleton (left) | 25093 | diffusion-MRI | 2289 |
| Mean FA in fornix cres+stria terminalis on FA skeleton (right) | 25094 | diffusion-MRI | 2289 |
| Mean FA in fornix cres+stria terminalis on FA skeleton (left) | 25095 | diffusion-MRI | 2289 |
| Mean FA in superior longitudinal fasciculus on FA skeleton (right) | 25096 | diffusion-MRI | 2289 |
| Mean FA in superior longitudinal fasciculus on FA skeleton (left) | 25097 | diffusion-MRI | 2289 |
| Mean FA in superior fronto-occipital fasciculus on FA skeleton (right) | 25098 | diffusion-MRI | 2289 |
| Mean FA in superior fronto-occipital fasciculus on FA skeleton (left) | 25099 | diffusion-MRI | 2289 |
| Mean FA in uncinate fasciculus on FA skeleton (right) | 25100 | diffusion-MRI | 2289 |
| Mean FA in uncinate fasciculus on FA skeleton (left) | 25101 | diffusion-MRI | 2289 |
| Mean FA in tapetum on FA skeleton (right) | 25102 | diffusion-MRI | 2289 |
| Mean FA in tapetum on FA skeleton (left) | 25103 | diffusion-MRI | 2289 |
| Mean MD in middle cerebellar peduncle on FA skeleton | 25104 | diffusion-MRI | 2289 |
| Mean MD in pontine crossing tract on FA skeleton | 25105 | diffusion-MRI | 2289 |
| Mean MD in genu of corpus callosum on FA skeleton | 25106 | diffusion-MRI | 2289 |
| Mean MD in body of corpus callosum on FA skeleton | 25107 | diffusion-MRI | 2289 |
| Mean MD in splenium of corpus callosum on FA skeleton | 25108 | diffusion-MRI | 2289 |
| Mean MD in fornix on FA skeleton | 25109 | diffusion-MRI | 2289 |
| Mean MD in corticospinal tract on FA skeleton (right) | 25110 | diffusion-MRI | 2289 |
| Mean MD in corticospinal tract on FA skeleton (left) | 25111 | diffusion-MRI | 2289 |
| Mean MD in medial lemniscus on FA skeleton (right) | 25112 | diffusion-MRI | 2289 |
| Mean MD in medial lemniscus on FA skeleton (left) | 25113 | diffusion-MRI | 2289 |
| Mean MD in inferior cerebellar peduncle on FA skeleton (right) | 25114 | diffusion-MRI | 2289 |
| Mean MD in inferior cerebellar peduncle on FA skeleton (left) | 25115 | diffusion-MRI | 2289 |
| Mean MD in superior cerebellar peduncle on FA skeleton (right) | 25116 | diffusion-MRI | 2289 |
| Mean MD in superior cerebellar peduncle on FA skeleton (left) | 25117 | diffusion-MRI | 2289 |
| Mean MD in cerebral peduncle on FA skeleton (right) | 25118 | diffusion-MRI | 2289 |
| Mean MD in cerebral peduncle on FA skeleton (left) | 25119 | diffusion-MRI | 2289 |
| Mean MD in anterior limb of internal capsule on FA skeleton (right) | 25120 | diffusion-MRI | 2289 |
| Mean MD in anterior limb of internal capsule on FA skeleton (left) | 25121 | diffusion-MRI | 2289 |
| Mean MD in posterior limb of internal capsule on FA skeleton (right) | 25122 | diffusion-MRI | 2289 |
| Mean MD in posterior limb of internal capsule on FA skeleton (left) | 25123 | diffusion-MRI | 2289 |
| Mean MD in retrolenticular part of internal capsule on FA skeleton (right) | 25124 | diffusion-MRI | 2289 |
| Mean MD in retrolenticular part of internal capsule on FA skeleton (left) | 25125 | diffusion-MRI | 2289 |
| Mean MD in anterior corona radiata on FA skeleton (right) | 25126 | diffusion-MRI | 2289 |
| Mean MD in anterior corona radiata on FA skeleton (left) | 25127 | diffusion-MRI | 2289 |
| Mean MD in superior corona radiata on FA skeleton (right) | 25128 | diffusion-MRI | 2289 |
| Mean MD in superior corona radiata on FA skeleton (left) | 25129 | diffusion-MRI | 2289 |
| Mean MD in posterior corona radiata on FA skeleton (right) | 25130 | diffusion-MRI | 2289 |
| Mean MD in posterior corona radiata on FA skeleton (left) | 25131 | diffusion-MRI | 2289 |
| Mean MD in posterior thalamic radiation on FA skeleton (right) | 25132 | diffusion-MRI | 2289 |
| Mean MD in posterior thalamic radiation on FA skeleton (left) | 25133 | diffusion-MRI | 2289 |
| Mean MD in sagittal stratum on FA skeleton (right) | 25134 | diffusion-MRI | 2289 |
| Mean MD in sagittal stratum on FA skeleton (left) | 25135 | diffusion-MRI | 2289 |
| Mean MD in external capsule on FA skeleton (right) | 25136 | diffusion-MRI | 2289 |
| Mean MD in external capsule on FA skeleton (left) | 25137 | diffusion-MRI | 2289 |
| Mean MD in cingulum cingulate gyrus on FA skeleton (right) | 25138 | diffusion-MRI | 2289 |
| Mean MD in cingulum cingulate gyrus on FA skeleton (left) | 25139 | diffusion-MRI | 2289 |
| Mean MD in cingulum hippocampus on FA skeleton (right) | 25140 | diffusion-MRI | 2289 |
| Mean MD in cingulum hippocampus on FA skeleton (left) | 25141 | diffusion-MRI | 2289 |
| Mean MD in fornix cres+stria terminalis on FA skeleton (right) | 25142 | diffusion-MRI | 2289 |
| Mean MD in fornix cres+stria terminalis on FA skeleton (left) | 25143 | diffusion-MRI | 2289 |
| Mean MD in superior longitudinal fasciculus on FA skeleton (right) | 25144 | diffusion-MRI | 2289 |
| Mean MD in superior longitudinal fasciculus on FA skeleton (left) | 25145 | diffusion-MRI | 2289 |
| Mean MD in superior fronto-occipital fasciculus on FA skeleton (right) | 25146 | diffusion-MRI | 2289 |
| Mean MD in superior fronto-occipital fasciculus on FA skeleton (left) | 25147 | diffusion-MRI | 2289 |
| Mean MD in uncinate fasciculus on FA skeleton (right) | 25148 | diffusion-MRI | 2289 |
| Mean MD in uncinate fasciculus on FA skeleton (left) | 25149 | diffusion-MRI | 2289 |
| Mean MD in tapetum on FA skeleton (right) | 25150 | diffusion-MRI | 2289 |
| Mean MD in tapetum on FA skeleton (left) | 25151 | diffusion-MRI | 2289 |
| Mean MO in middle cerebellar peduncle on FA skeleton | 25152 | diffusion-MRI | 2289 |
| Mean MO in pontine crossing tract on FA skeleton | 25153 | diffusion-MRI | 2289 |
| Mean MO in genu of corpus callosum on FA skeleton | 25154 | diffusion-MRI | 2289 |
| Mean MO in body of corpus callosum on FA skeleton | 25155 | diffusion-MRI | 2289 |
| Mean MO in splenium of corpus callosum on FA skeleton | 25156 | diffusion-MRI | 2289 |
| Mean MO in fornix on FA skeleton | 25157 | diffusion-MRI | 2289 |
| Mean MO in corticospinal tract on FA skeleton (right) | 25158 | diffusion-MRI | 2289 |
| Mean MO in corticospinal tract on FA skeleton (left) | 25159 | diffusion-MRI | 2289 |
| Mean MO in medial lemniscus on FA skeleton (right) | 25160 | diffusion-MRI | 2289 |
| Mean MO in medial lemniscus on FA skeleton (left) | 25161 | diffusion-MRI | 2289 |
| Mean MO in inferior cerebellar peduncle on FA skeleton (right) | 25162 | diffusion-MRI | 2289 |
| Mean MO in inferior cerebellar peduncle on FA skeleton (left) | 25163 | diffusion-MRI | 2289 |
| Mean MO in superior cerebellar peduncle on FA skeleton (right) | 25164 | diffusion-MRI | 2289 |
| Mean MO in superior cerebellar peduncle on FA skeleton (left) | 25165 | diffusion-MRI | 2289 |
| Mean MO in cerebral peduncle on FA skeleton (right) | 25166 | diffusion-MRI | 2289 |
| Mean MO in cerebral peduncle on FA skeleton (left) | 25167 | diffusion-MRI | 2289 |
| Mean MO in anterior limb of internal capsule on FA skeleton (right) | 25168 | diffusion-MRI | 2289 |
| Mean MO in anterior limb of internal capsule on FA skeleton (left) | 25169 | diffusion-MRI | 2289 |
| Mean MO in posterior limb of internal capsule on FA skeleton (right) | 25170 | diffusion-MRI | 2289 |
| Mean MO in posterior limb of internal capsule on FA skeleton (left) | 25171 | diffusion-MRI | 2289 |
| Mean MO in retrolenticular part of internal capsule on FA skeleton (right) | 25172 | diffusion-MRI | 2289 |
| Mean MO in retrolenticular part of internal capsule on FA skeleton (left) | 25173 | diffusion-MRI | 2289 |
| Mean MO in anterior corona radiata on FA skeleton (right) | 25174 | diffusion-MRI | 2289 |
| Mean MO in anterior corona radiata on FA skeleton (left) | 25175 | diffusion-MRI | 2289 |
| Mean MO in superior corona radiata on FA skeleton (right) | 25176 | diffusion-MRI | 2289 |
| Mean MO in superior corona radiata on FA skeleton (left) | 25177 | diffusion-MRI | 2289 |
| Mean MO in posterior corona radiata on FA skeleton (right) | 25178 | diffusion-MRI | 2289 |
| Mean MO in posterior corona radiata on FA skeleton (left) | 25179 | diffusion-MRI | 2289 |
| Mean MO in posterior thalamic radiation on FA skeleton (right) | 25180 | diffusion-MRI | 2289 |
| Mean MO in posterior thalamic radiation on FA skeleton (left) | 25181 | diffusion-MRI | 2289 |
| Mean MO in sagittal stratum on FA skeleton (right) | 25182 | diffusion-MRI | 2289 |
| Mean MO in sagittal stratum on FA skeleton (left) | 25183 | diffusion-MRI | 2289 |
| Mean MO in external capsule on FA skeleton (right) | 25184 | diffusion-MRI | 2289 |
| Mean MO in external capsule on FA skeleton (left) | 25185 | diffusion-MRI | 2289 |
| Mean MO in cingulum cingulate gyrus on FA skeleton (right) | 25186 | diffusion-MRI | 2289 |
| Mean MO in cingulum cingulate gyrus on FA skeleton (left) | 25187 | diffusion-MRI | 2289 |
| Mean MO in cingulum hippocampus on FA skeleton (right) | 25188 | diffusion-MRI | 2289 |
| Mean MO in cingulum hippocampus on FA skeleton (left) | 25189 | diffusion-MRI | 2289 |
| Mean MO in fornix cres+stria terminalis on FA skeleton (right) | 25190 | diffusion-MRI | 2289 |
| Mean MO in fornix cres+stria terminalis on FA skeleton (left) | 25191 | diffusion-MRI | 2289 |
| Mean MO in superior longitudinal fasciculus on FA skeleton (right) | 25192 | diffusion-MRI | 2289 |
| Mean MO in superior longitudinal fasciculus on FA skeleton (left) | 25193 | diffusion-MRI | 2289 |
| Mean MO in superior fronto-occipital fasciculus on FA skeleton (right) | 25194 | diffusion-MRI | 2289 |
| Mean MO in superior fronto-occipital fasciculus on FA skeleton (left) | 25195 | diffusion-MRI | 2289 |
| Mean MO in uncinate fasciculus on FA skeleton (right) | 25196 | diffusion-MRI | 2289 |
| Mean MO in uncinate fasciculus on FA skeleton (left) | 25197 | diffusion-MRI | 2289 |
| Mean MO in tapetum on FA skeleton (right) | 25198 | diffusion-MRI | 2289 |
| Mean MO in tapetum on FA skeleton (left) | 25199 | diffusion-MRI | 2289 |
| Mean L1 in middle cerebellar peduncle on FA skeleton | 25200 | diffusion-MRI | 2289 |
| Mean L1 in pontine crossing tract on FA skeleton | 25201 | diffusion-MRI | 2289 |
| Mean L1 in genu of corpus callosum on FA skeleton | 25202 | diffusion-MRI | 2289 |
| Mean L1 in body of corpus callosum on FA skeleton | 25203 | diffusion-MRI | 2289 |
| Mean L1 in splenium of corpus callosum on FA skeleton | 25204 | diffusion-MRI | 2289 |
| Mean L1 in fornix on FA skeleton | 25205 | diffusion-MRI | 2289 |
| Mean L1 in corticospinal tract on FA skeleton (right) | 25206 | diffusion-MRI | 2289 |
| Mean L1 in corticospinal tract on FA skeleton (left) | 25207 | diffusion-MRI | 2289 |
| Mean L1 in medial lemniscus on FA skeleton (right) | 25208 | diffusion-MRI | 2289 |
| Mean L1 in medial lemniscus on FA skeleton (left) | 25209 | diffusion-MRI | 2289 |
| Mean L1 in inferior cerebellar peduncle on FA skeleton (right) | 25210 | diffusion-MRI | 2289 |
| Mean L1 in inferior cerebellar peduncle on FA skeleton (left) | 25211 | diffusion-MRI | 2289 |
| Mean L1 in superior cerebellar peduncle on FA skeleton (right) | 25212 | diffusion-MRI | 2289 |
| Mean L1 in superior cerebellar peduncle on FA skeleton (left) | 25213 | diffusion-MRI | 2289 |
| Mean L1 in cerebral peduncle on FA skeleton (right) | 25214 | diffusion-MRI | 2289 |
| Mean L1 in cerebral peduncle on FA skeleton (left) | 25215 | diffusion-MRI | 2289 |
| Mean L1 in anterior limb of internal capsule on FA skeleton (right) | 25216 | diffusion-MRI | 2289 |
| Mean L1 in anterior limb of internal capsule on FA skeleton (left) | 25217 | diffusion-MRI | 2289 |
| Mean L1 in posterior limb of internal capsule on FA skeleton (right) | 25218 | diffusion-MRI | 2289 |
| Mean L1 in posterior limb of internal capsule on FA skeleton (left) | 25219 | diffusion-MRI | 2289 |
| Mean L1 in retrolenticular part of internal capsule on FA skeleton (right) | 25220 | diffusion-MRI | 2289 |
| Mean L1 in retrolenticular part of internal capsule on FA skeleton (left) | 25221 | diffusion-MRI | 2289 |
| Mean L1 in anterior corona radiata on FA skeleton (right) | 25222 | diffusion-MRI | 2289 |
| Mean L1 in anterior corona radiata on FA skeleton (left) | 25223 | diffusion-MRI | 2289 |
| Mean L1 in superior corona radiata on FA skeleton (right) | 25224 | diffusion-MRI | 2289 |
| Mean L1 in superior corona radiata on FA skeleton (left) | 25225 | diffusion-MRI | 2289 |
| Mean L1 in posterior corona radiata on FA skeleton (right) | 25226 | diffusion-MRI | 2289 |
| Mean L1 in posterior corona radiata on FA skeleton (left) | 25227 | diffusion-MRI | 2289 |
| Mean L1 in posterior thalamic radiation on FA skeleton (right) | 25228 | diffusion-MRI | 2289 |
| Mean L1 in posterior thalamic radiation on FA skeleton (left) | 25229 | diffusion-MRI | 2289 |
| Mean L1 in sagittal stratum on FA skeleton (right) | 25230 | diffusion-MRI | 2289 |
| Mean L1 in sagittal stratum on FA skeleton (left) | 25231 | diffusion-MRI | 2289 |
| Mean L1 in external capsule on FA skeleton (right) | 25232 | diffusion-MRI | 2289 |
| Mean L1 in external capsule on FA skeleton (left) | 25233 | diffusion-MRI | 2289 |
| Mean L1 in cingulum cingulate gyrus on FA skeleton (right) | 25234 | diffusion-MRI | 2289 |
| Mean L1 in cingulum cingulate gyrus on FA skeleton (left) | 25235 | diffusion-MRI | 2289 |
| Mean L1 in cingulum hippocampus on FA skeleton (right) | 25236 | diffusion-MRI | 2289 |
| Mean L1 in cingulum hippocampus on FA skeleton (left) | 25237 | diffusion-MRI | 2289 |
| Mean L1 in fornix cres+stria terminalis on FA skeleton (right) | 25238 | diffusion-MRI | 2289 |
| Mean L1 in fornix cres+stria terminalis on FA skeleton (left) | 25239 | diffusion-MRI | 2289 |
| Mean L1 in superior longitudinal fasciculus on FA skeleton (right) | 25240 | diffusion-MRI | 2289 |
| Mean L1 in superior longitudinal fasciculus on FA skeleton (left) | 25241 | diffusion-MRI | 2289 |
| Mean L1 in superior fronto-occipital fasciculus on FA skeleton (right) | 25242 | diffusion-MRI | 2289 |
| Mean L1 in superior fronto-occipital fasciculus on FA skeleton (left) | 25243 | diffusion-MRI | 2289 |
| Mean L1 in uncinate fasciculus on FA skeleton (right) | 25244 | diffusion-MRI | 2289 |
| Mean L1 in uncinate fasciculus on FA skeleton (left) | 25245 | diffusion-MRI | 2289 |
| Mean L1 in tapetum on FA skeleton (right) | 25246 | diffusion-MRI | 2289 |
| Mean L1 in tapetum on FA skeleton (left) | 25247 | diffusion-MRI | 2289 |
| Mean L2 in middle cerebellar peduncle on FA skeleton | 25248 | diffusion-MRI | 2289 |
| Mean L2 in pontine crossing tract on FA skeleton | 25249 | diffusion-MRI | 2289 |
| Mean L2 in genu of corpus callosum on FA skeleton | 25250 | diffusion-MRI | 2289 |
| Mean L2 in body of corpus callosum on FA skeleton | 25251 | diffusion-MRI | 2289 |
| Mean L2 in splenium of corpus callosum on FA skeleton | 25252 | diffusion-MRI | 2289 |
| Mean L2 in fornix on FA skeleton | 25253 | diffusion-MRI | 2289 |
| Mean L2 in corticospinal tract on FA skeleton (right) | 25254 | diffusion-MRI | 2289 |
| Mean L2 in corticospinal tract on FA skeleton (left) | 25255 | diffusion-MRI | 2289 |
| Mean L2 in medial lemniscus on FA skeleton (right) | 25256 | diffusion-MRI | 2289 |
| Mean L2 in medial lemniscus on FA skeleton (left) | 25257 | diffusion-MRI | 2289 |
| Mean L2 in inferior cerebellar peduncle on FA skeleton (right) | 25258 | diffusion-MRI | 2289 |
| Mean L2 in inferior cerebellar peduncle on FA skeleton (left) | 25259 | diffusion-MRI | 2289 |
| Mean L2 in superior cerebellar peduncle on FA skeleton (right) | 25260 | diffusion-MRI | 2289 |
| Mean L2 in superior cerebellar peduncle on FA skeleton (left) | 25261 | diffusion-MRI | 2289 |
| Mean L2 in cerebral peduncle on FA skeleton (right) | 25262 | diffusion-MRI | 2289 |
| Mean L2 in cerebral peduncle on FA skeleton (left) | 25263 | diffusion-MRI | 2289 |
| Mean L2 in anterior limb of internal capsule on FA skeleton (right) | 25264 | diffusion-MRI | 2289 |
| Mean L2 in anterior limb of internal capsule on FA skeleton (left) | 25265 | diffusion-MRI | 2289 |
| Mean L2 in posterior limb of internal capsule on FA skeleton (right) | 25266 | diffusion-MRI | 2289 |
| Mean L2 in posterior limb of internal capsule on FA skeleton (left) | 25267 | diffusion-MRI | 2289 |
| Mean L2 in retrolenticular part of internal capsule on FA skeleton (right) | 25268 | diffusion-MRI | 2289 |
| Mean L2 in retrolenticular part of internal capsule on FA skeleton (left) | 25269 | diffusion-MRI | 2289 |
| Mean L2 in anterior corona radiata on FA skeleton (right) | 25270 | diffusion-MRI | 2289 |
| Mean L2 in anterior corona radiata on FA skeleton (left) | 25271 | diffusion-MRI | 2289 |
| Mean L2 in superior corona radiata on FA skeleton (right) | 25272 | diffusion-MRI | 2289 |
| Mean L2 in superior corona radiata on FA skeleton (left) | 25273 | diffusion-MRI | 2289 |
| Mean L2 in posterior corona radiata on FA skeleton (right) | 25274 | diffusion-MRI | 2289 |
| Mean L2 in posterior corona radiata on FA skeleton (left) | 25275 | diffusion-MRI | 2289 |
| Mean L2 in posterior thalamic radiation on FA skeleton (right) | 25276 | diffusion-MRI | 2289 |
| Mean L2 in posterior thalamic radiation on FA skeleton (left) | 25277 | diffusion-MRI | 2289 |
| Mean L2 in sagittal stratum on FA skeleton (right) | 25278 | diffusion-MRI | 2289 |
| Mean L2 in sagittal stratum on FA skeleton (left) | 25279 | diffusion-MRI | 2289 |
| Mean L2 in external capsule on FA skeleton (right) | 25280 | diffusion-MRI | 2289 |
| Mean L2 in external capsule on FA skeleton (left) | 25281 | diffusion-MRI | 2289 |
| Mean L2 in cingulum cingulate gyrus on FA skeleton (right) | 25282 | diffusion-MRI | 2289 |
| Mean L2 in cingulum cingulate gyrus on FA skeleton (left) | 25283 | diffusion-MRI | 2289 |
| Mean L2 in cingulum hippocampus on FA skeleton (right) | 25284 | diffusion-MRI | 2289 |
| Mean L2 in cingulum hippocampus on FA skeleton (left) | 25285 | diffusion-MRI | 2289 |
| Mean L2 in fornix cres+stria terminalis on FA skeleton (right) | 25286 | diffusion-MRI | 2289 |
| Mean L2 in fornix cres+stria terminalis on FA skeleton (left) | 25287 | diffusion-MRI | 2289 |
| Mean L2 in superior longitudinal fasciculus on FA skeleton (right) | 25288 | diffusion-MRI | 2289 |
| Mean L2 in superior longitudinal fasciculus on FA skeleton (left) | 25289 | diffusion-MRI | 2289 |
| Mean L2 in superior fronto-occipital fasciculus on FA skeleton (right) | 25290 | diffusion-MRI | 2289 |
| Mean L2 in superior fronto-occipital fasciculus on FA skeleton (left) | 25291 | diffusion-MRI | 2289 |
| Mean L2 in uncinate fasciculus on FA skeleton (right) | 25292 | diffusion-MRI | 2289 |
| Mean L2 in uncinate fasciculus on FA skeleton (left) | 25293 | diffusion-MRI | 2289 |
| Mean L2 in tapetum on FA skeleton (right) | 25294 | diffusion-MRI | 2289 |
| Mean L2 in tapetum on FA skeleton (left) | 25295 | diffusion-MRI | 2289 |
| Mean L3 in middle cerebellar peduncle on FA skeleton | 25296 | diffusion-MRI | 2289 |
| Mean L3 in pontine crossing tract on FA skeleton | 25297 | diffusion-MRI | 2289 |
| Mean L3 in genu of corpus callosum on FA skeleton | 25298 | diffusion-MRI | 2289 |
| Mean L3 in body of corpus callosum on FA skeleton | 25299 | diffusion-MRI | 2289 |
| Mean L3 in splenium of corpus callosum on FA skeleton | 25300 | diffusion-MRI | 2289 |
| Mean L3 in fornix on FA skeleton | 25301 | diffusion-MRI | 2289 |
| Mean L3 in corticospinal tract on FA skeleton (right) | 25302 | diffusion-MRI | 2289 |
| Mean L3 in corticospinal tract on FA skeleton (left) | 25303 | diffusion-MRI | 2289 |
| Mean L3 in medial lemniscus on FA skeleton (right) | 25304 | diffusion-MRI | 2289 |
| Mean L3 in medial lemniscus on FA skeleton (left) | 25305 | diffusion-MRI | 2289 |
| Mean L3 in inferior cerebellar peduncle on FA skeleton (right) | 25306 | diffusion-MRI | 2289 |
| Mean L3 in inferior cerebellar peduncle on FA skeleton (left) | 25307 | diffusion-MRI | 2289 |
| Mean L3 in superior cerebellar peduncle on FA skeleton (right) | 25308 | diffusion-MRI | 2289 |
| Mean L3 in superior cerebellar peduncle on FA skeleton (left) | 25309 | diffusion-MRI | 2289 |
| Mean L3 in cerebral peduncle on FA skeleton (right) | 25310 | diffusion-MRI | 2289 |
| Mean L3 in cerebral peduncle on FA skeleton (left) | 25311 | diffusion-MRI | 2289 |
| Mean L3 in anterior limb of internal capsule on FA skeleton (right) | 25312 | diffusion-MRI | 2289 |
| Mean L3 in anterior limb of internal capsule on FA skeleton (left) | 25313 | diffusion-MRI | 2289 |
| Mean L3 in posterior limb of internal capsule on FA skeleton (right) | 25314 | diffusion-MRI | 2289 |
| Mean L3 in posterior limb of internal capsule on FA skeleton (left) | 25315 | diffusion-MRI | 2289 |
| Mean L3 in retrolenticular part of internal capsule on FA skeleton (right) | 25316 | diffusion-MRI | 2289 |
| Mean L3 in retrolenticular part of internal capsule on FA skeleton (left) | 25317 | diffusion-MRI | 2289 |
| Mean L3 in anterior corona radiata on FA skeleton (right) | 25318 | diffusion-MRI | 2289 |
| Mean L3 in anterior corona radiata on FA skeleton (left) | 25319 | diffusion-MRI | 2289 |
| Mean L3 in superior corona radiata on FA skeleton (right) | 25320 | diffusion-MRI | 2289 |
| Mean L3 in superior corona radiata on FA skeleton (left) | 25321 | diffusion-MRI | 2289 |
| Mean L3 in posterior corona radiata on FA skeleton (right) | 25322 | diffusion-MRI | 2289 |
| Mean L3 in posterior corona radiata on FA skeleton (left) | 25323 | diffusion-MRI | 2289 |
| Mean L3 in posterior thalamic radiation on FA skeleton (right) | 25324 | diffusion-MRI | 2289 |
| Mean L3 in posterior thalamic radiation on FA skeleton (left) | 25325 | diffusion-MRI | 2289 |
| Mean L3 in sagittal stratum on FA skeleton (right) | 25326 | diffusion-MRI | 2289 |
| Mean L3 in sagittal stratum on FA skeleton (left) | 25327 | diffusion-MRI | 2289 |
| Mean L3 in external capsule on FA skeleton (right) | 25328 | diffusion-MRI | 2289 |
| Mean L3 in external capsule on FA skeleton (left) | 25329 | diffusion-MRI | 2289 |
| Mean L3 in cingulum cingulate gyrus on FA skeleton (right) | 25330 | diffusion-MRI | 2289 |
| Mean L3 in cingulum cingulate gyrus on FA skeleton (left) | 25331 | diffusion-MRI | 2289 |
| Mean L3 in cingulum hippocampus on FA skeleton (right) | 25332 | diffusion-MRI | 2289 |
| Mean L3 in cingulum hippocampus on FA skeleton (left) | 25333 | diffusion-MRI | 2289 |
| Mean L3 in fornix cres+stria terminalis on FA skeleton (right) | 25334 | diffusion-MRI | 2289 |
| Mean L3 in fornix cres+stria terminalis on FA skeleton (left) | 25335 | diffusion-MRI | 2289 |
| Mean L3 in superior longitudinal fasciculus on FA skeleton (right) | 25336 | diffusion-MRI | 2289 |
| Mean L3 in superior longitudinal fasciculus on FA skeleton (left) | 25337 | diffusion-MRI | 2289 |
| Mean L3 in superior fronto-occipital fasciculus on FA skeleton (right) | 25338 | diffusion-MRI | 2289 |
| Mean L3 in superior fronto-occipital fasciculus on FA skeleton (left) | 25339 | diffusion-MRI | 2289 |
| Mean L3 in uncinate fasciculus on FA skeleton (right) | 25340 | diffusion-MRI | 2289 |
| Mean L3 in uncinate fasciculus on FA skeleton (left) | 25341 | diffusion-MRI | 2289 |
| Mean L3 in tapetum on FA skeleton (right) | 25342 | diffusion-MRI | 2289 |
| Mean L3 in tapetum on FA skeleton (left) | 25343 | diffusion-MRI | 2289 |
| Mean ICVF in middle cerebellar peduncle on FA skeleton | 25344 | diffusion-MRI | 2291 |
| Mean ICVF in pontine crossing tract on FA skeleton | 25345 | diffusion-MRI | 2291 |
| Mean ICVF in genu of corpus callosum on FA skeleton | 25346 | diffusion-MRI | 2291 |
| Mean ICVF in body of corpus callosum on FA skeleton | 25347 | diffusion-MRI | 2291 |
| Mean ICVF in splenium of corpus callosum on FA skeleton | 25348 | diffusion-MRI | 2291 |
| Mean ICVF in fornix on FA skeleton | 25349 | diffusion-MRI | 2291 |
| Mean ICVF in corticospinal tract on FA skeleton (right) | 25350 | diffusion-MRI | 2291 |
| Mean ICVF in corticospinal tract on FA skeleton (left) | 25351 | diffusion-MRI | 2291 |
| Mean ICVF in medial lemniscus on FA skeleton (right) | 25352 | diffusion-MRI | 2291 |
| Mean ICVF in medial lemniscus on FA skeleton (left) | 25353 | diffusion-MRI | 2291 |
| Mean ICVF in inferior cerebellar peduncle on FA skeleton (right) | 25354 | diffusion-MRI | 2291 |
| Mean ICVF in inferior cerebellar peduncle on FA skeleton (left) | 25355 | diffusion-MRI | 2291 |
| Mean ICVF in superior cerebellar peduncle on FA skeleton (right) | 25356 | diffusion-MRI | 2291 |
| Mean ICVF in superior cerebellar peduncle on FA skeleton (left) | 25357 | diffusion-MRI | 2291 |
| Mean ICVF in cerebral peduncle on FA skeleton (right) | 25358 | diffusion-MRI | 2291 |
| Mean ICVF in cerebral peduncle on FA skeleton (left) | 25359 | diffusion-MRI | 2291 |
| Mean ICVF in anterior limb of internal capsule on FA skeleton (right) | 25360 | diffusion-MRI | 2291 |
| Mean ICVF in anterior limb of internal capsule on FA skeleton (left) | 25361 | diffusion-MRI | 2291 |
| Mean ICVF in posterior limb of internal capsule on FA skeleton (right) | 25362 | diffusion-MRI | 2291 |
| Mean ICVF in posterior limb of internal capsule on FA skeleton (left) | 25363 | diffusion-MRI | 2291 |
| Mean ICVF in retrolenticular part of internal capsule on FA skeleton (right) | 25364 | diffusion-MRI | 2291 |
| Mean ICVF in retrolenticular part of internal capsule on FA skeleton (left) | 25365 | diffusion-MRI | 2291 |
| Mean ICVF in anterior corona radiata on FA skeleton (right) | 25366 | diffusion-MRI | 2291 |
| Mean ICVF in anterior corona radiata on FA skeleton (left) | 25367 | diffusion-MRI | 2291 |
| Mean ICVF in superior corona radiata on FA skeleton (right) | 25368 | diffusion-MRI | 2291 |
| Mean ICVF in superior corona radiata on FA skeleton (left) | 25369 | diffusion-MRI | 2291 |
| Mean ICVF in posterior corona radiata on FA skeleton (right) | 25370 | diffusion-MRI | 2291 |
| Mean ICVF in posterior corona radiata on FA skeleton (left) | 25371 | diffusion-MRI | 2291 |
| Mean ICVF in posterior thalamic radiation on FA skeleton (right) | 25372 | diffusion-MRI | 2291 |
| Mean ICVF in posterior thalamic radiation on FA skeleton (left) | 25373 | diffusion-MRI | 2291 |
| Mean ICVF in sagittal stratum on FA skeleton (right) | 25374 | diffusion-MRI | 2291 |
| Mean ICVF in sagittal stratum on FA skeleton (left) | 25375 | diffusion-MRI | 2291 |
| Mean ICVF in external capsule on FA skeleton (right) | 25376 | diffusion-MRI | 2291 |
| Mean ICVF in external capsule on FA skeleton (left) | 25377 | diffusion-MRI | 2291 |
| Mean ICVF in cingulum cingulate gyrus on FA skeleton (right) | 25378 | diffusion-MRI | 2291 |
| Mean ICVF in cingulum cingulate gyrus on FA skeleton (left) | 25379 | diffusion-MRI | 2291 |
| Mean ICVF in cingulum hippocampus on FA skeleton (right) | 25380 | diffusion-MRI | 2291 |
| Mean ICVF in cingulum hippocampus on FA skeleton (left) | 25381 | diffusion-MRI | 2291 |
| Mean ICVF in fornix cres+stria terminalis on FA skeleton (right) | 25382 | diffusion-MRI | 2291 |
| Mean ICVF in fornix cres+stria terminalis on FA skeleton (left) | 25383 | diffusion-MRI | 2291 |
| Mean ICVF in superior longitudinal fasciculus on FA skeleton (right) | 25384 | diffusion-MRI | 2291 |
| Mean ICVF in superior longitudinal fasciculus on FA skeleton (left) | 25385 | diffusion-MRI | 2291 |
| Mean ICVF in superior fronto-occipital fasciculus on FA skeleton (right) | 25386 | diffusion-MRI | 2291 |
| Mean ICVF in superior fronto-occipital fasciculus on FA skeleton (left) | 25387 | diffusion-MRI | 2291 |
| Mean ICVF in uncinate fasciculus on FA skeleton (right) | 25388 | diffusion-MRI | 2291 |
| Mean ICVF in uncinate fasciculus on FA skeleton (left) | 25389 | diffusion-MRI | 2291 |
| Mean ICVF in tapetum on FA skeleton (right) | 25390 | diffusion-MRI | 2291 |
| Mean ICVF in tapetum on FA skeleton (left) | 25391 | diffusion-MRI | 2291 |
| Mean OD in middle cerebellar peduncle on FA skeleton | 25392 | diffusion-MRI | 2291 |
| Mean OD in pontine crossing tract on FA skeleton | 25393 | diffusion-MRI | 2291 |
| Mean OD in genu of corpus callosum on FA skeleton | 25394 | diffusion-MRI | 2291 |
| Mean OD in body of corpus callosum on FA skeleton | 25395 | diffusion-MRI | 2291 |
| Mean OD in splenium of corpus callosum on FA skeleton | 25396 | diffusion-MRI | 2291 |
| Mean OD in fornix on FA skeleton | 25397 | diffusion-MRI | 2291 |
| Mean OD in corticospinal tract on FA skeleton (right) | 25398 | diffusion-MRI | 2291 |
| Mean OD in corticospinal tract on FA skeleton (left) | 25399 | diffusion-MRI | 2291 |
| Mean OD in medial lemniscus on FA skeleton (right) | 25400 | diffusion-MRI | 2291 |
| Mean OD in medial lemniscus on FA skeleton (left) | 25401 | diffusion-MRI | 2291 |
| Mean OD in inferior cerebellar peduncle on FA skeleton (right) | 25402 | diffusion-MRI | 2291 |
| Mean OD in inferior cerebellar peduncle on FA skeleton (left) | 25403 | diffusion-MRI | 2291 |
| Mean OD in superior cerebellar peduncle on FA skeleton (right) | 25404 | diffusion-MRI | 2291 |
| Mean OD in superior cerebellar peduncle on FA skeleton (left) | 25405 | diffusion-MRI | 2291 |
| Mean OD in cerebral peduncle on FA skeleton (right) | 25406 | diffusion-MRI | 2291 |
| Mean OD in cerebral peduncle on FA skeleton (left) | 25407 | diffusion-MRI | 2291 |
| Mean OD in anterior limb of internal capsule on FA skeleton (right) | 25408 | diffusion-MRI | 2291 |
| Mean OD in anterior limb of internal capsule on FA skeleton (left) | 25409 | diffusion-MRI | 2291 |
| Mean OD in posterior limb of internal capsule on FA skeleton (right) | 25410 | diffusion-MRI | 2291 |
| Mean OD in posterior limb of internal capsule on FA skeleton (left) | 25411 | diffusion-MRI | 2291 |
| Mean OD in retrolenticular part of internal capsule on FA skeleton (right) | 25412 | diffusion-MRI | 2291 |
| Mean OD in retrolenticular part of internal capsule on FA skeleton (left) | 25413 | diffusion-MRI | 2291 |
| Mean OD in anterior corona radiata on FA skeleton (right) | 25414 | diffusion-MRI | 2291 |
| Mean OD in anterior corona radiata on FA skeleton (left) | 25415 | diffusion-MRI | 2291 |
| Mean OD in superior corona radiata on FA skeleton (right) | 25416 | diffusion-MRI | 2291 |
| Mean OD in superior corona radiata on FA skeleton (left) | 25417 | diffusion-MRI | 2291 |
| Mean OD in posterior corona radiata on FA skeleton (right) | 25418 | diffusion-MRI | 2291 |
| Mean OD in posterior corona radiata on FA skeleton (left) | 25419 | diffusion-MRI | 2291 |
| Mean OD in posterior thalamic radiation on FA skeleton (right) | 25420 | diffusion-MRI | 2291 |
| Mean OD in posterior thalamic radiation on FA skeleton (left) | 25421 | diffusion-MRI | 2291 |
| Mean OD in sagittal stratum on FA skeleton (right) | 25422 | diffusion-MRI | 2291 |
| Mean OD in sagittal stratum on FA skeleton (left) | 25423 | diffusion-MRI | 2291 |
| Mean OD in external capsule on FA skeleton (right) | 25424 | diffusion-MRI | 2291 |
| Mean OD in external capsule on FA skeleton (left) | 25425 | diffusion-MRI | 2291 |
| Mean OD in cingulum cingulate gyrus on FA skeleton (right) | 25426 | diffusion-MRI | 2291 |
| Mean OD in cingulum cingulate gyrus on FA skeleton (left) | 25427 | diffusion-MRI | 2291 |
| Mean OD in cingulum hippocampus on FA skeleton (right) | 25428 | diffusion-MRI | 2291 |
| Mean OD in cingulum hippocampus on FA skeleton (left) | 25429 | diffusion-MRI | 2291 |
| Mean OD in fornix cres+stria terminalis on FA skeleton (right) | 25430 | diffusion-MRI | 2291 |
| Mean OD in fornix cres+stria terminalis on FA skeleton (left) | 25431 | diffusion-MRI | 2291 |
| Mean OD in superior longitudinal fasciculus on FA skeleton (right) | 25432 | diffusion-MRI | 2291 |
| Mean OD in superior longitudinal fasciculus on FA skeleton (left) | 25433 | diffusion-MRI | 2291 |
| Mean OD in superior fronto-occipital fasciculus on FA skeleton (right) | 25434 | diffusion-MRI | 2291 |
| Mean OD in superior fronto-occipital fasciculus on FA skeleton (left) | 25435 | diffusion-MRI | 2291 |
| Mean OD in uncinate fasciculus on FA skeleton (right) | 25436 | diffusion-MRI | 2291 |
| Mean OD in uncinate fasciculus on FA skeleton (left) | 25437 | diffusion-MRI | 2291 |
| Mean OD in tapetum on FA skeleton (right) | 25438 | diffusion-MRI | 2291 |
| Mean OD in tapetum on FA skeleton (left) | 25439 | diffusion-MRI | 2291 |
| Mean ISOVF in middle cerebellar peduncle on FA skeleton | 25440 | diffusion-MRI | 2291 |
| Mean ISOVF in pontine crossing tract on FA skeleton | 25441 | diffusion-MRI | 2291 |
| Mean ISOVF in genu of corpus callosum on FA skeleton | 25442 | diffusion-MRI | 2291 |
| Mean ISOVF in body of corpus callosum on FA skeleton | 25443 | diffusion-MRI | 2291 |
| Mean ISOVF in splenium of corpus callosum on FA skeleton | 25444 | diffusion-MRI | 2291 |
| Mean ISOVF in fornix on FA skeleton | 25445 | diffusion-MRI | 2291 |
| Mean ISOVF in corticospinal tract on FA skeleton (right) | 25446 | diffusion-MRI | 2291 |
| Mean ISOVF in corticospinal tract on FA skeleton (left) | 25447 | diffusion-MRI | 2291 |
| Mean ISOVF in medial lemniscus on FA skeleton (right) | 25448 | diffusion-MRI | 2291 |
| Mean ISOVF in medial lemniscus on FA skeleton (left) | 25449 | diffusion-MRI | 2291 |
| Mean ISOVF in inferior cerebellar peduncle on FA skeleton (right) | 25450 | diffusion-MRI | 2291 |
| Mean ISOVF in inferior cerebellar peduncle on FA skeleton (left) | 25451 | diffusion-MRI | 2291 |
| Mean ISOVF in superior cerebellar peduncle on FA skeleton (right) | 25452 | diffusion-MRI | 2291 |
| Mean ISOVF in superior cerebellar peduncle on FA skeleton (left) | 25453 | diffusion-MRI | 2291 |
| Mean ISOVF in cerebral peduncle on FA skeleton (right) | 25454 | diffusion-MRI | 2291 |
| Mean ISOVF in cerebral peduncle on FA skeleton (left) | 25455 | diffusion-MRI | 2291 |
| Mean ISOVF in anterior limb of internal capsule on FA skeleton (right) | 25456 | diffusion-MRI | 2291 |
| Mean ISOVF in anterior limb of internal capsule on FA skeleton (left) | 25457 | diffusion-MRI | 2291 |
| Mean ISOVF in posterior limb of internal capsule on FA skeleton (right) | 25458 | diffusion-MRI | 2291 |
| Mean ISOVF in posterior limb of internal capsule on FA skeleton (left) | 25459 | diffusion-MRI | 2291 |
| Mean ISOVF in retrolenticular part of internal capsule on FA skeleton (right) | 25460 | diffusion-MRI | 2291 |
| Mean ISOVF in retrolenticular part of internal capsule on FA skeleton (left) | 25461 | diffusion-MRI | 2291 |
| Mean ISOVF in anterior corona radiata on FA skeleton (right) | 25462 | diffusion-MRI | 2291 |
| Mean ISOVF in anterior corona radiata on FA skeleton (left) | 25463 | diffusion-MRI | 2291 |
| Mean ISOVF in superior corona radiata on FA skeleton (right) | 25464 | diffusion-MRI | 2291 |
| Mean ISOVF in superior corona radiata on FA skeleton (left) | 25465 | diffusion-MRI | 2291 |
| Mean ISOVF in posterior corona radiata on FA skeleton (right) | 25466 | diffusion-MRI | 2291 |
| Mean ISOVF in posterior corona radiata on FA skeleton (left) | 25467 | diffusion-MRI | 2291 |
| Mean ISOVF in posterior thalamic radiation on FA skeleton (right) | 25468 | diffusion-MRI | 2291 |
| Mean ISOVF in posterior thalamic radiation on FA skeleton (left) | 25469 | diffusion-MRI | 2291 |
| Mean ISOVF in sagittal stratum on FA skeleton (right) | 25470 | diffusion-MRI | 2291 |
| Mean ISOVF in sagittal stratum on FA skeleton (left) | 25471 | diffusion-MRI | 2291 |
| Mean ISOVF in external capsule on FA skeleton (right) | 25472 | diffusion-MRI | 2291 |
| Mean ISOVF in external capsule on FA skeleton (left) | 25473 | diffusion-MRI | 2291 |
| Mean ISOVF in cingulum cingulate gyrus on FA skeleton (right) | 25474 | diffusion-MRI | 2291 |
| Mean ISOVF in cingulum cingulate gyrus on FA skeleton (left) | 25475 | diffusion-MRI | 2291 |
| Mean ISOVF in cingulum hippocampus on FA skeleton (right) | 25476 | diffusion-MRI | 2291 |
| Mean ISOVF in cingulum hippocampus on FA skeleton (left) | 25477 | diffusion-MRI | 2291 |
| Mean ISOVF in fornix cres+stria terminalis on FA skeleton (right) | 25478 | diffusion-MRI | 2291 |
| Mean ISOVF in fornix cres+stria terminalis on FA skeleton (left) | 25479 | diffusion-MRI | 2291 |
| Mean ISOVF in superior longitudinal fasciculus on FA skeleton (right) | 25480 | diffusion-MRI | 2291 |
| Mean ISOVF in superior longitudinal fasciculus on FA skeleton (left) | 25481 | diffusion-MRI | 2291 |
| Mean ISOVF in superior fronto-occipital fasciculus on FA skeleton (right) | 25482 | diffusion-MRI | 2291 |
| Mean ISOVF in superior fronto-occipital fasciculus on FA skeleton (left) | 25483 | diffusion-MRI | 2291 |
| Mean ISOVF in uncinate fasciculus on FA skeleton (right) | 25484 | diffusion-MRI | 2291 |
| Mean ISOVF in uncinate fasciculus on FA skeleton (left) | 25485 | diffusion-MRI | 2291 |
| Mean ISOVF in tapetum on FA skeleton (right) | 25486 | diffusion-MRI | 2291 |
| Mean ISOVF in tapetum on FA skeleton (left) | 25487 | diffusion-MRI | 2291 |
| Weighted-mean FA in tract acoustic radiation (left) | 25488 | diffusion-MRI | 2291 |
| Weighted-mean FA in tract acoustic radiation (right) | 25489 | diffusion-MRI | 2291 |
| Weighted-mean FA in tract anterior thalamic radiation (left) | 25490 | diffusion-MRI | 2291 |
| Weighted-mean FA in tract anterior thalamic radiation (right) | 25491 | diffusion-MRI | 2291 |
| Weighted-mean FA in tract cingulate gyrus part of cingulum (left) | 25492 | diffusion-MRI | 2291 |
| Weighted-mean FA in tract cingulate gyrus part of cingulum (right) | 25493 | diffusion-MRI | 2291 |
| Weighted-mean FA in tract parahippocampal part of cingulum (left) | 25494 | diffusion-MRI | 2291 |
| Weighted-mean FA in tract parahippocampal part of cingulum (right) | 25495 | diffusion-MRI | 2291 |
| Weighted-mean FA in tract corticospinal tract (left) | 25496 | diffusion-MRI | 2291 |
| Weighted-mean FA in tract corticospinal tract (right) | 25497 | diffusion-MRI | 2291 |
| Weighted-mean FA in tract forceps major | 25498 | diffusion-MRI | 2291 |
| Weighted-mean FA in tract forceps minor | 25499 | diffusion-MRI | 2291 |
| Weighted-mean FA in tract inferior fronto-occipital fasciculus (left) | 25500 | diffusion-MRI | 2291 |
| Weighted-mean FA in tract inferior fronto-occipital fasciculus (right) | 25501 | diffusion-MRI | 2291 |
| Weighted-mean FA in tract inferior longitudinal fasciculus (left) | 25502 | diffusion-MRI | 2291 |
| Weighted-mean FA in tract inferior longitudinal fasciculus (right) | 25503 | diffusion-MRI | 2291 |
| Weighted-mean FA in tract middle cerebellar peduncle | 25504 | diffusion-MRI | 2291 |
| Weighted-mean FA in tract medial lemniscus (left) | 25505 | diffusion-MRI | 2291 |
| Weighted-mean FA in tract medial lemniscus (right) | 25506 | diffusion-MRI | 2291 |
| Weighted-mean FA in tract posterior thalamic radiation (left) | 25507 | diffusion-MRI | 2291 |
| Weighted-mean FA in tract posterior thalamic radiation (right) | 25508 | diffusion-MRI | 2291 |
| Weighted-mean FA in tract superior longitudinal fasciculus (left) | 25509 | diffusion-MRI | 2291 |
| Weighted-mean FA in tract superior longitudinal fasciculus (right) | 25510 | diffusion-MRI | 2291 |
| Weighted-mean FA in tract superior thalamic radiation (left) | 25511 | diffusion-MRI | 2291 |
| Weighted-mean FA in tract superior thalamic radiation (right) | 25512 | diffusion-MRI | 2291 |
| Weighted-mean FA in tract uncinate fasciculus (left) | 25513 | diffusion-MRI | 2291 |
| Weighted-mean FA in tract uncinate fasciculus (right) | 25514 | diffusion-MRI | 2291 |
| Weighted-mean MD in tract acoustic radiation (left) | 25515 | diffusion-MRI | 2291 |
| Weighted-mean MD in tract acoustic radiation (right) | 25516 | diffusion-MRI | 2291 |
| Weighted-mean MD in tract anterior thalamic radiation (left) | 25517 | diffusion-MRI | 2291 |
| Weighted-mean MD in tract anterior thalamic radiation (right) | 25518 | diffusion-MRI | 2291 |
| Weighted-mean MD in tract cingulate gyrus part of cingulum (left) | 25519 | diffusion-MRI | 2291 |
| Weighted-mean MD in tract cingulate gyrus part of cingulum (right) | 25520 | diffusion-MRI | 2291 |
| Weighted-mean MD in tract parahippocampal part of cingulum (left) | 25521 | diffusion-MRI | 2291 |
| Weighted-mean MD in tract parahippocampal part of cingulum (right) | 25522 | diffusion-MRI | 2291 |
| Weighted-mean MD in tract corticospinal tract (left) | 25523 | diffusion-MRI | 2291 |
| Weighted-mean MD in tract corticospinal tract (right) | 25524 | diffusion-MRI | 2291 |
| Weighted-mean MD in tract forceps major | 25525 | diffusion-MRI | 2291 |
| Weighted-mean MD in tract forceps minor | 25526 | diffusion-MRI | 2291 |
| Weighted-mean MD in tract inferior fronto-occipital fasciculus (left) | 25527 | diffusion-MRI | 2291 |
| Weighted-mean MD in tract inferior fronto-occipital fasciculus (right) | 25528 | diffusion-MRI | 2291 |
| Weighted-mean MD in tract inferior longitudinal fasciculus (left) | 25529 | diffusion-MRI | 2291 |
| Weighted-mean MD in tract inferior longitudinal fasciculus (right) | 25530 | diffusion-MRI | 2291 |
| Weighted-mean MD in tract middle cerebellar peduncle | 25531 | diffusion-MRI | 2291 |
| Weighted-mean MD in tract medial lemniscus (left) | 25532 | diffusion-MRI | 2291 |
| Weighted-mean MD in tract medial lemniscus (right) | 25533 | diffusion-MRI | 2291 |
| Weighted-mean MD in tract posterior thalamic radiation (left) | 25534 | diffusion-MRI | 2291 |
| Weighted-mean MD in tract posterior thalamic radiation (right) | 25535 | diffusion-MRI | 2291 |
| Weighted-mean MD in tract superior longitudinal fasciculus (left) | 25536 | diffusion-MRI | 2291 |
| Weighted-mean MD in tract superior longitudinal fasciculus (right) | 25537 | diffusion-MRI | 2291 |
| Weighted-mean MD in tract superior thalamic radiation (left) | 25538 | diffusion-MRI | 2291 |
| Weighted-mean MD in tract superior thalamic radiation (right) | 25539 | diffusion-MRI | 2291 |
| Weighted-mean MD in tract uncinate fasciculus (left) | 25540 | diffusion-MRI | 2291 |
| Weighted-mean MD in tract uncinate fasciculus (right) | 25541 | diffusion-MRI | 2291 |
| Weighted-mean MO in tract acoustic radiation (left) | 25542 | diffusion-MRI | 2291 |
| Weighted-mean MO in tract acoustic radiation (right) | 25543 | diffusion-MRI | 2291 |
| Weighted-mean MO in tract anterior thalamic radiation (left) | 25544 | diffusion-MRI | 2291 |
| Weighted-mean MO in tract anterior thalamic radiation (right) | 25545 | diffusion-MRI | 2291 |
| Weighted-mean MO in tract cingulate gyrus part of cingulum (left) | 25546 | diffusion-MRI | 2291 |
| Weighted-mean MO in tract cingulate gyrus part of cingulum (right) | 25547 | diffusion-MRI | 2291 |
| Weighted-mean MO in tract parahippocampal part of cingulum (left) | 25548 | diffusion-MRI | 2291 |
| Weighted-mean MO in tract parahippocampal part of cingulum (right) | 25549 | diffusion-MRI | 2291 |
| Weighted-mean MO in tract corticospinal tract (left) | 25550 | diffusion-MRI | 2291 |
| Weighted-mean MO in tract corticospinal tract (right) | 25551 | diffusion-MRI | 2291 |
| Weighted-mean MO in tract forceps major | 25552 | diffusion-MRI | 2291 |
| Weighted-mean MO in tract forceps minor | 25553 | diffusion-MRI | 2291 |
| Weighted-mean MO in tract inferior fronto-occipital fasciculus (left) | 25554 | diffusion-MRI | 2291 |
| Weighted-mean MO in tract inferior fronto-occipital fasciculus (right) | 25555 | diffusion-MRI | 2291 |
| Weighted-mean MO in tract inferior longitudinal fasciculus (left) | 25556 | diffusion-MRI | 2291 |
| Weighted-mean MO in tract inferior longitudinal fasciculus (right) | 25557 | diffusion-MRI | 2291 |
| Weighted-mean MO in tract middle cerebellar peduncle | 25558 | diffusion-MRI | 2291 |
| Weighted-mean MO in tract medial lemniscus (left) | 25559 | diffusion-MRI | 2291 |
| Weighted-mean MO in tract medial lemniscus (right) | 25560 | diffusion-MRI | 2291 |
| Weighted-mean MO in tract posterior thalamic radiation (left) | 25561 | diffusion-MRI | 2291 |
| Weighted-mean MO in tract posterior thalamic radiation (right) | 25562 | diffusion-MRI | 2291 |
| Weighted-mean MO in tract superior longitudinal fasciculus (left) | 25563 | diffusion-MRI | 2291 |
| Weighted-mean MO in tract superior longitudinal fasciculus (right) | 25564 | diffusion-MRI | 2291 |
| Weighted-mean MO in tract superior thalamic radiation (left) | 25565 | diffusion-MRI | 2291 |
| Weighted-mean MO in tract superior thalamic radiation (right) | 25566 | diffusion-MRI | 2291 |
| Weighted-mean MO in tract uncinate fasciculus (left) | 25567 | diffusion-MRI | 2291 |
| Weighted-mean MO in tract uncinate fasciculus (right) | 25568 | diffusion-MRI | 2291 |
| Weighted-mean L1 in tract acoustic radiation (left) | 25569 | diffusion-MRI | 2291 |
| Weighted-mean L1 in tract acoustic radiation (right) | 25570 | diffusion-MRI | 2291 |
| Weighted-mean L1 in tract anterior thalamic radiation (left) | 25571 | diffusion-MRI | 2291 |
| Weighted-mean L1 in tract anterior thalamic radiation (right) | 25572 | diffusion-MRI | 2291 |
| Weighted-mean L1 in tract cingulate gyrus part of cingulum (left) | 25573 | diffusion-MRI | 2291 |
| Weighted-mean L1 in tract cingulate gyrus part of cingulum (right) | 25574 | diffusion-MRI | 2291 |
| Weighted-mean L1 in tract parahippocampal part of cingulum (left) | 25575 | diffusion-MRI | 2291 |
| Weighted-mean L1 in tract parahippocampal part of cingulum (right) | 25576 | diffusion-MRI | 2291 |
| Weighted-mean L1 in tract corticospinal tract (left) | 25577 | diffusion-MRI | 2291 |
| Weighted-mean L1 in tract corticospinal tract (right) | 25578 | diffusion-MRI | 2291 |
| Weighted-mean L1 in tract forceps major | 25579 | diffusion-MRI | 2291 |
| Weighted-mean L1 in tract forceps minor | 25580 | diffusion-MRI | 2291 |
| Weighted-mean L1 in tract inferior fronto-occipital fasciculus (left) | 25581 | diffusion-MRI | 2291 |
| Weighted-mean L1 in tract inferior fronto-occipital fasciculus (right) | 25582 | diffusion-MRI | 2291 |
| Weighted-mean L1 in tract inferior longitudinal fasciculus (left) | 25583 | diffusion-MRI | 2291 |
| Weighted-mean L1 in tract inferior longitudinal fasciculus (right) | 25584 | diffusion-MRI | 2291 |
| Weighted-mean L1 in tract middle cerebellar peduncle | 25585 | diffusion-MRI | 2291 |
| Weighted-mean L1 in tract medial lemniscus (left) | 25586 | diffusion-MRI | 2291 |
| Weighted-mean L1 in tract medial lemniscus (right) | 25587 | diffusion-MRI | 2291 |
| Weighted-mean L1 in tract posterior thalamic radiation (left) | 25588 | diffusion-MRI | 2291 |
| Weighted-mean L1 in tract posterior thalamic radiation (right) | 25589 | diffusion-MRI | 2291 |
| Weighted-mean L1 in tract superior longitudinal fasciculus (left) | 25590 | diffusion-MRI | 2291 |
| Weighted-mean L1 in tract superior longitudinal fasciculus (right) | 25591 | diffusion-MRI | 2291 |
| Weighted-mean L1 in tract superior thalamic radiation (left) | 25592 | diffusion-MRI | 2291 |
| Weighted-mean L1 in tract superior thalamic radiation (right) | 25593 | diffusion-MRI | 2291 |
| Weighted-mean L1 in tract uncinate fasciculus (left) | 25594 | diffusion-MRI | 2291 |
| Weighted-mean L1 in tract uncinate fasciculus (right) | 25595 | diffusion-MRI | 2291 |
| Weighted-mean L2 in tract acoustic radiation (left) | 25596 | diffusion-MRI | 2291 |
| Weighted-mean L2 in tract acoustic radiation (right) | 25597 | diffusion-MRI | 2291 |
| Weighted-mean L2 in tract anterior thalamic radiation (left) | 25598 | diffusion-MRI | 2291 |
| Weighted-mean L2 in tract anterior thalamic radiation (right) | 25599 | diffusion-MRI | 2291 |
| Weighted-mean L2 in tract cingulate gyrus part of cingulum (left) | 25600 | diffusion-MRI | 2291 |
| Weighted-mean L2 in tract cingulate gyrus part of cingulum (right) | 25601 | diffusion-MRI | 2291 |
| Weighted-mean L2 in tract parahippocampal part of cingulum (left) | 25602 | diffusion-MRI | 2291 |
| Weighted-mean L2 in tract parahippocampal part of cingulum (right) | 25603 | diffusion-MRI | 2291 |
| Weighted-mean L2 in tract corticospinal tract (left) | 25604 | diffusion-MRI | 2291 |
| Weighted-mean L2 in tract corticospinal tract (right) | 25605 | diffusion-MRI | 2291 |
| Weighted-mean L2 in tract forceps major | 25606 | diffusion-MRI | 2291 |
| Weighted-mean L2 in tract forceps minor | 25607 | diffusion-MRI | 2291 |
| Weighted-mean L2 in tract inferior fronto-occipital fasciculus (left) | 25608 | diffusion-MRI | 2291 |
| Weighted-mean L2 in tract inferior fronto-occipital fasciculus (right) | 25609 | diffusion-MRI | 2291 |
| Weighted-mean L2 in tract inferior longitudinal fasciculus (left) | 25610 | diffusion-MRI | 2291 |
| Weighted-mean L2 in tract inferior longitudinal fasciculus (right) | 25611 | diffusion-MRI | 2291 |
| Weighted-mean L2 in tract middle cerebellar peduncle | 25612 | diffusion-MRI | 2291 |
| Weighted-mean L2 in tract medial lemniscus (left) | 25613 | diffusion-MRI | 2291 |
| Weighted-mean L2 in tract medial lemniscus (right) | 25614 | diffusion-MRI | 2291 |
| Weighted-mean L2 in tract posterior thalamic radiation (left) | 25615 | diffusion-MRI | 2291 |
| Weighted-mean L2 in tract posterior thalamic radiation (right) | 25616 | diffusion-MRI | 2291 |
| Weighted-mean L2 in tract superior longitudinal fasciculus (left) | 25617 | diffusion-MRI | 2291 |
| Weighted-mean L2 in tract superior longitudinal fasciculus (right) | 25618 | diffusion-MRI | 2291 |
| Weighted-mean L2 in tract superior thalamic radiation (left) | 25619 | diffusion-MRI | 2291 |
| Weighted-mean L2 in tract superior thalamic radiation (right) | 25620 | diffusion-MRI | 2291 |
| Weighted-mean L2 in tract uncinate fasciculus (left) | 25621 | diffusion-MRI | 2291 |
| Weighted-mean L2 in tract uncinate fasciculus (right) | 25622 | diffusion-MRI | 2291 |
| Weighted-mean L3 in tract acoustic radiation (left) | 25623 | diffusion-MRI | 2291 |
| Weighted-mean L3 in tract acoustic radiation (right) | 25624 | diffusion-MRI | 2291 |
| Weighted-mean L3 in tract anterior thalamic radiation (left) | 25625 | diffusion-MRI | 2291 |
| Weighted-mean L3 in tract anterior thalamic radiation (right) | 25626 | diffusion-MRI | 2291 |
| Weighted-mean L3 in tract cingulate gyrus part of cingulum (left) | 25627 | diffusion-MRI | 2291 |
| Weighted-mean L3 in tract cingulate gyrus part of cingulum (right) | 25628 | diffusion-MRI | 2291 |
| Weighted-mean L3 in tract parahippocampal part of cingulum (left) | 25629 | diffusion-MRI | 2291 |
| Weighted-mean L3 in tract parahippocampal part of cingulum (right) | 25630 | diffusion-MRI | 2291 |
| Weighted-mean L3 in tract corticospinal tract (left) | 25631 | diffusion-MRI | 2291 |
| Weighted-mean L3 in tract corticospinal tract (right) | 25632 | diffusion-MRI | 2291 |
| Weighted-mean L3 in tract forceps major | 25633 | diffusion-MRI | 2291 |
| Weighted-mean L3 in tract forceps minor | 25634 | diffusion-MRI | 2291 |
| Weighted-mean L3 in tract inferior fronto-occipital fasciculus (left) | 25635 | diffusion-MRI | 2291 |
| Weighted-mean L3 in tract inferior fronto-occipital fasciculus (right) | 25636 | diffusion-MRI | 2291 |
| Weighted-mean L3 in tract inferior longitudinal fasciculus (left) | 25637 | diffusion-MRI | 2291 |
| Weighted-mean L3 in tract inferior longitudinal fasciculus (right) | 25638 | diffusion-MRI | 2291 |
| Weighted-mean L3 in tract middle cerebellar peduncle | 25639 | diffusion-MRI | 2291 |
| Weighted-mean L3 in tract medial lemniscus (left) | 25640 | diffusion-MRI | 2291 |
| Weighted-mean L3 in tract medial lemniscus (right) | 25641 | diffusion-MRI | 2291 |
| Weighted-mean L3 in tract posterior thalamic radiation (left) | 25642 | diffusion-MRI | 2291 |
| Weighted-mean L3 in tract posterior thalamic radiation (right) | 25643 | diffusion-MRI | 2291 |
| Weighted-mean L3 in tract superior longitudinal fasciculus (left) | 25644 | diffusion-MRI | 2291 |
| Weighted-mean L3 in tract superior longitudinal fasciculus (right) | 25645 | diffusion-MRI | 2291 |
| Weighted-mean L3 in tract superior thalamic radiation (left) | 25646 | diffusion-MRI | 2291 |
| Weighted-mean L3 in tract superior thalamic radiation (right) | 25647 | diffusion-MRI | 2291 |
| Weighted-mean L3 in tract uncinate fasciculus (left) | 25648 | diffusion-MRI | 2291 |
| Weighted-mean L3 in tract uncinate fasciculus (right) | 25649 | diffusion-MRI | 2291 |
| Weighted-mean ICVF in tract acoustic radiation (left) | 25650 | diffusion-MRI | 2292 |
| Weighted-mean ICVF in tract acoustic radiation (right) | 25651 | diffusion-MRI | 2292 |
| Weighted-mean ICVF in tract anterior thalamic radiation (left) | 25652 | diffusion-MRI | 2292 |
| Weighted-mean ICVF in tract anterior thalamic radiation (right) | 25653 | diffusion-MRI | 2292 |
| Weighted-mean ICVF in tract cingulate gyrus part of cingulum (left) | 25654 | diffusion-MRI | 2292 |
| Weighted-mean ICVF in tract cingulate gyrus part of cingulum (right) | 25655 | diffusion-MRI | 2292 |
| Weighted-mean ICVF in tract parahippocampal part of cingulum (left) | 25656 | diffusion-MRI | 2292 |
| Weighted-mean ICVF in tract parahippocampal part of cingulum (right) | 25657 | diffusion-MRI | 2292 |
| Weighted-mean ICVF in tract corticospinal tract (left) | 25658 | diffusion-MRI | 2292 |
| Weighted-mean ICVF in tract corticospinal tract (right) | 25659 | diffusion-MRI | 2292 |
| Weighted-mean ICVF in tract forceps major | 25660 | diffusion-MRI | 2292 |
| Weighted-mean ICVF in tract forceps minor | 25661 | diffusion-MRI | 2292 |
| Weighted-mean ICVF in tract inferior fronto-occipital fasciculus (left) | 25662 | diffusion-MRI | 2292 |
| Weighted-mean ICVF in tract inferior fronto-occipital fasciculus (right) | 25663 | diffusion-MRI | 2292 |
| Weighted-mean ICVF in tract inferior longitudinal fasciculus (left) | 25664 | diffusion-MRI | 2292 |
| Weighted-mean ICVF in tract inferior longitudinal fasciculus (right) | 25665 | diffusion-MRI | 2292 |
| Weighted-mean ICVF in tract middle cerebellar peduncle | 25666 | diffusion-MRI | 2292 |
| Weighted-mean ICVF in tract medial lemniscus (left) | 25667 | diffusion-MRI | 2292 |
| Weighted-mean ICVF in tract medial lemniscus (right) | 25668 | diffusion-MRI | 2292 |
| Weighted-mean ICVF in tract posterior thalamic radiation (left) | 25669 | diffusion-MRI | 2292 |
| Weighted-mean ICVF in tract posterior thalamic radiation (right) | 25670 | diffusion-MRI | 2292 |
| Weighted-mean ICVF in tract superior longitudinal fasciculus (left) | 25671 | diffusion-MRI | 2292 |
| Weighted-mean ICVF in tract superior longitudinal fasciculus (right) | 25672 | diffusion-MRI | 2292 |
| Weighted-mean ICVF in tract superior thalamic radiation (left) | 25673 | diffusion-MRI | 2292 |
| Weighted-mean ICVF in tract superior thalamic radiation (right) | 25674 | diffusion-MRI | 2292 |
| Weighted-mean ICVF in tract uncinate fasciculus (left) | 25675 | diffusion-MRI | 2292 |
| Weighted-mean ICVF in tract uncinate fasciculus (right) | 25676 | diffusion-MRI | 2292 |
| Weighted-mean OD in tract acoustic radiation (left) | 25677 | diffusion-MRI | 2292 |
| Weighted-mean OD in tract acoustic radiation (right) | 25678 | diffusion-MRI | 2292 |
| Weighted-mean OD in tract anterior thalamic radiation (left) | 25679 | diffusion-MRI | 2292 |
| Weighted-mean OD in tract anterior thalamic radiation (right) | 25680 | diffusion-MRI | 2292 |
| Weighted-mean OD in tract cingulate gyrus part of cingulum (left) | 25681 | diffusion-MRI | 2292 |
| Weighted-mean OD in tract cingulate gyrus part of cingulum (right) | 25682 | diffusion-MRI | 2292 |
| Weighted-mean OD in tract parahippocampal part of cingulum (left) | 25683 | diffusion-MRI | 2292 |
| Weighted-mean OD in tract parahippocampal part of cingulum (right) | 25684 | diffusion-MRI | 2292 |
| Weighted-mean OD in tract corticospinal tract (left) | 25685 | diffusion-MRI | 2292 |
| Weighted-mean OD in tract corticospinal tract (right) | 25686 | diffusion-MRI | 2292 |
| Weighted-mean OD in tract forceps major | 25687 | diffusion-MRI | 2292 |
| Weighted-mean OD in tract forceps minor | 25688 | diffusion-MRI | 2292 |
| Weighted-mean OD in tract inferior fronto-occipital fasciculus (left) | 25689 | diffusion-MRI | 2292 |
| Weighted-mean OD in tract inferior fronto-occipital fasciculus (right) | 25690 | diffusion-MRI | 2292 |
| Weighted-mean OD in tract inferior longitudinal fasciculus (left) | 25691 | diffusion-MRI | 2292 |
| Weighted-mean OD in tract inferior longitudinal fasciculus (right) | 25692 | diffusion-MRI | 2292 |
| Weighted-mean OD in tract middle cerebellar peduncle | 25693 | diffusion-MRI | 2292 |
| Weighted-mean OD in tract medial lemniscus (left) | 25694 | diffusion-MRI | 2292 |
| Weighted-mean OD in tract medial lemniscus (right) | 25695 | diffusion-MRI | 2292 |
| Weighted-mean OD in tract posterior thalamic radiation (left) | 25696 | diffusion-MRI | 2292 |
| Weighted-mean OD in tract posterior thalamic radiation (right) | 25697 | diffusion-MRI | 2292 |
| Weighted-mean OD in tract superior longitudinal fasciculus (left) | 25698 | diffusion-MRI | 2292 |
| Weighted-mean OD in tract superior longitudinal fasciculus (right) | 25699 | diffusion-MRI | 2292 |
| Weighted-mean OD in tract superior thalamic radiation (left) | 25700 | diffusion-MRI | 2292 |
| Weighted-mean OD in tract superior thalamic radiation (right) | 25701 | diffusion-MRI | 2292 |
| Weighted-mean OD in tract uncinate fasciculus (left) | 25702 | diffusion-MRI | 2292 |
| Weighted-mean OD in tract uncinate fasciculus (right) | 25703 | diffusion-MRI | 2292 |
| Weighted-mean ISOVF in tract acoustic radiation (left) | 25704 | diffusion-MRI | 2292 |
| Weighted-mean ISOVF in tract acoustic radiation (right) | 25705 | diffusion-MRI | 2292 |
| Weighted-mean ISOVF in tract anterior thalamic radiation (left) | 25706 | diffusion-MRI | 2292 |
| Weighted-mean ISOVF in tract anterior thalamic radiation (right) | 25707 | diffusion-MRI | 2292 |
| Weighted-mean ISOVF in tract cingulate gyrus part of cingulum (left) | 25708 | diffusion-MRI | 2292 |
| Weighted-mean ISOVF in tract cingulate gyrus part of cingulum (right) | 25709 | diffusion-MRI | 2292 |
| Weighted-mean ISOVF in tract parahippocampal part of cingulum (left) | 25710 | diffusion-MRI | 2292 |
| Weighted-mean ISOVF in tract parahippocampal part of cingulum (right) | 25711 | diffusion-MRI | 2292 |
| Weighted-mean ISOVF in tract corticospinal tract (left) | 25712 | diffusion-MRI | 2292 |
| Weighted-mean ISOVF in tract corticospinal tract (right) | 25713 | diffusion-MRI | 2292 |
| Weighted-mean ISOVF in tract forceps major | 25714 | diffusion-MRI | 2292 |
| Weighted-mean ISOVF in tract forceps minor | 25715 | diffusion-MRI | 2292 |
| Weighted-mean ISOVF in tract inferior fronto-occipital fasciculus (left) | 25716 | diffusion-MRI | 2292 |
| Weighted-mean ISOVF in tract inferior fronto-occipital fasciculus (right) | 25717 | diffusion-MRI | 2292 |
| Weighted-mean ISOVF in tract inferior longitudinal fasciculus (left) | 25718 | diffusion-MRI | 2292 |
| Weighted-mean ISOVF in tract inferior longitudinal fasciculus (right) | 25719 | diffusion-MRI | 2292 |
| Weighted-mean ISOVF in tract middle cerebellar peduncle | 25720 | diffusion-MRI | 2292 |
| Weighted-mean ISOVF in tract medial lemniscus (left) | 25721 | diffusion-MRI | 2292 |
| Weighted-mean ISOVF in tract medial lemniscus (right) | 25722 | diffusion-MRI | 2292 |
| Weighted-mean ISOVF in tract posterior thalamic radiation (left) | 25723 | diffusion-MRI | 2292 |
| Weighted-mean ISOVF in tract posterior thalamic radiation (right) | 25724 | diffusion-MRI | 2292 |
| Weighted-mean ISOVF in tract superior longitudinal fasciculus (left) | 25725 | diffusion-MRI | 2292 |
| Weighted-mean ISOVF in tract superior longitudinal fasciculus (right) | 25726 | diffusion-MRI | 2292 |
| Weighted-mean ISOVF in tract superior thalamic radiation (left) | 25727 | diffusion-MRI | 2292 |
| Weighted-mean ISOVF in tract superior thalamic radiation (right) | 25728 | diffusion-MRI | 2292 |
| Weighted-mean ISOVF in tract uncinate fasciculus (left) | 25729 | diffusion-MRI | 2292 |
| Weighted-mean ISOVF in tract uncinate fasciculus (right) | 25730 | diffusion-MRI | 2292 |
| rfMRI partial correlation matrix, dimension 25 | 25752* | resting-state fMRI | 1888 |
| Median BOLD effect (in group-defined mask) for shapes activation | 25040 | task fMRI | 7373 |
| Median z-statistic (in group-defined mask) for shapes activation | 25042 | task fMRI | 7373 |
| Median BOLD effect (in group-defined mask) for faces activation | 25044 | task fMRI | 7373 |
| Median z-statistic (in group-defined mask) for faces activation | 25046 | task fMRI | 7373 |
| Median BOLD effect (in group-defined mask) for faces-shapes contrast | 25048 | task fMRI | 7373 |
| Median BOLD effect (in group-defined amygdala activation mask) for faces-shapes contrast | 25052 | task fMRI | 7373 |
| Median z-statistic (in group-defined amygdala activation mask) for faces-shapes contrast | 25054 | task fMRI | 7373 |
| 90th percentile of BOLD effect (in group-defined mask) for shapes activation | 25761 | task fMRI | 7373 |
| 90th percentile of z-statistic (in group-defined mask) for shapes activation | 25762 | task fMRI | 7373 |
| 90th percentile of BOLD effect (in group-defined mask) for faces activation | 25763 | task fMRI | 7373 |
| 90th percentile of z-statistic (in group-defined mask) for faces activation | 25764 | task fMRI | 7373 |
| 90th percentile of BOLD effect (in group-defined mask) for faces-shapes contrast | 25765 | task fMRI | 7373 |
| 90th percentile of BOLD effect (in group-defined amygdala activation mask) for faces-shapes contrast | 25767 | task fMRI | 7373 |
| 90th percentile of z-statistic (in group-defined amygdala activation mask) for faces-shapes contrast | 25768 | task fMRI | 7373 |

**Table S3.** Hyperparameter spaces of 9 models in Bayesian optimization

| **Model** | **Feature Selector** | **Hyperparameter** | **Range** |
| --- | --- | --- | --- |
| XGBoost | NA | alpha | 0.001 to 1000 |
| XGBoost | NA | gamma | 0.001 to 1000 |
| XGBoost | NA | learning_rate | 0.01 to 0.3 |
| XGBoost | NA | max_depth | 2 to 6 |
| XGBoost | NA | n_estimators | 200 to 800 |
| XGBoost | NA | reg_lambda | 0.001 to 1000 |
| XGBoost | NA | subsample | 0.6 to 1 |
| LASSO | NA | alpha | 0.07 to 1 |
| SVR | NA | C | 1 to 1000 |
| XGBoost | FeatureWiz | alpha | 0.001 to 1000 |
| XGBoost | FeatureWiz | gamma | 0.001 to 1000 |
| XGBoost | FeatureWiz | learning_rate | 0.01 to 0.3 |
| XGBoost | FeatureWiz | max_depth | 2 to 6 |
| XGBoost | FeatureWiz | n_estimators | 200 to 800 |
| XGBoost | FeatureWiz | reg_lambda | 0.001 to 1000 |
| XGBoost | FeatureWiz | subsample | 0.6 to 1 |
| LASSO | FeatureWiz | alpha | 0.07 to 1 |
| SVR | FeatureWiz | C | 1 to 1000 |
| XGBoost | RFECV | alpha | 0.001 to 1000 |
| XGBoost | RFECV | gamma | 0.001 to 1000 |
| XGBoost | RFECV | learning_rate | 0.01 to 0.3 |
| XGBoost | RFECV | max_depth | 2 to 6 |
| XGBoost | RFECV | n_estimators | 200 to 800 |
| XGBoost | RFECV | reg_lambda | 0.001 to 1000 |
| XGBoost | RFECV | subsample | 0.6 to 1 |
| LASSO | RFECV | alpha | 0.07 to 1 |
| SVR | RFECV | C | 1 to 1000 |

Abbreviation: LASSO, least absolute shrinkage and selection operator; MAE, mean absolute error; NA, not available; RFECV, recursive feature elimination cross-validation; R^2^, R-squared; SVR, support vector regression; XGBoost, eXtreme Gradient Boosting.

**Table S4.** The chosen hyperparameters of 9 models

| **Model** | **Feature Selector** | **Hyperparameter** | **Range** |
| --- | --- | --- | --- |
| XGBoost | NA | alpha | 0.001 |
| XGBoost | NA | gamma | 0.010548939 |
| XGBoost | NA | learning_rate | 0.086711115 |
| XGBoost | NA | max_depth | 3 |
| XGBoost | NA | n_estimators | 536 |
| XGBoost | NA | reg_lambda | 118.9444064 |
| XGBoost | NA | subsample | 0.779595775 |
| LASSO | NA | alpha | 0.07 |
| SVR | NA | C | 19 |
| XGBoost | FeatureWiz | alpha | 24.66941176 |
| XGBoost | FeatureWiz | gamma | 2.833421611 |
| XGBoost | FeatureWiz | learning_rate | 0.196324798 |
| XGBoost | FeatureWiz | max_depth | 2 |
| XGBoost | FeatureWiz | n_estimators | 790 |
| XGBoost | FeatureWiz | reg_lambda | 1000 |
| XGBoost | FeatureWiz | subsample | 0.654883315 |
| LASSO | FeatureWiz | alpha | 0.07 |
| SVR | FeatureWiz | C | 26 |
| XGBoost | RFECV | alpha | 0.539132035 |
| XGBoost | RFECV | gamma | 0.001 |
| XGBoost | RFECV | learning_rate | 0.067385078 |
| XGBoost | RFECV | max_depth | 2 |
| XGBoost | RFECV | n_estimators | 732 |
| XGBoost | RFECV | reg_lambda | 6.84397712 |
| XGBoost | RFECV | subsample | 0.632891091 |
| LASSO | RFECV | alpha | 0.07 |
| SVR | RFECV | C | 5 |

Abbreviation: LASSO, least absolute shrinkage and selection operator; MAE, mean absolute error; NA, not available; RFECV, recursive feature elimination cross-validation; R^2^, R-squared; SVR, support vector regression; XGBoost, eXtreme Gradient Boosting.

**Table S5.** Model evaluation of 9 models in the validation set and testing set

| **Model** | **Feature Selector** | **MAE** | **R^2^** | **Pearson’s r** | **MSE** | **Explained Variance Score** |
| --- | --- | --- | --- | --- | --- | --- |
| Validation set | | | | | | |
| XGBoost | NA | 3.411 | 0.650 | 0.806 | 18.300 | 0.651 |
| LASSO | NA | 3.232 | 0.690 | 0.830 | 16.225 | 0.692 |
| SVR | NA | 3.235 | 0.680 | 0.825 | 16.731 | 0.681 |
| XGBoost | FeatureWiz | 3.285 | 0.676 | 0.822 | 16.967 | 0.677 |
| LASSO | FeatureWiz | 3.301 | 0.677 | 0.823 | 16.903 | 0.679 |
| SVR | FeatureWiz | 3.283 | 0.677 | 0.823 | 16.898 | 0.677 |
| XGBoost | RFECV | 3.415 | 0.650 | 0.806 | 18.322 | 0.651 |
| LASSO | RFECV | 3.348 | 0.666 | 0.816 | 17.472 | 0.668 |
| SVR | RFECV | 3.412 | 0.645 | 0.803 | 18.539 | 0.648 |
| Testing set |  |  |  |  |  |  |
| XGBoost | NA | 3.552 | 0.662 | 0.814 | 19.859 | 0.664 |
| LASSO | NA | 3.416 | 0.675 | 0.822 | 19.118 | 0.676 |
| SVR | NA | 3.455 | 0.680 | 0.825 | 18.811 | 0.682 |
| XGBoost | FeatureWiz | 3.526 | 0.667 | 0.817 | 19.577 | 0.669 |
| LASSO | FeatureWiz | 3.482 | 0.666 | 0.816 | 19.619 | 0.668 |
| SVR | FeatureWiz | 3.491 | 0.674 | 0.821 | 19.178 | 0.676 |
| XGBoost | RFECV | 3.545 | 0.663 | 0.814 | 19.820 | 0.665 |
| LASSO | RFECV | 3.525 | 0.657 | 0.814 | 20.155 | 0.659 |
| SVR | RFECV | 3.548 | 0.663 | 0.814 | 19.834 | 0.664 |

Abbreviation: LASSO, least absolute shrinkage and selection operator; MAE, mean absolute error; MSE, mean square error; NA, not available; RFECV, recursive feature elimination cross-validation; R^2^, R-squared; SVR, support vector regression; XGBoost, eXtreme Gradient Boosting.

| **Table S6.** Coefficients for 285 IDPs that significantly contribute to brain age estimation in the LASSO regression without feature selection | |  |
| --- | --- | --- |
| **Phenotype^1^** | **Coefficient** |  |
| Volume of grey matter (normalized for head size) | -1.3516 |  |
| Weighted-mean ICVF in tract forceps minor | -0.7169 |  |
| Mean ISOVF in fornix on FA skeleton | 0.6877 |  |
| Volume of grey matter in Ventral Striatum (left) | -0.6099 |  |
| Mean FA in superior cerebellar peduncle on FA skeleton (right) | 0.5779 |  |
| Volume of brain stem + 4th ventricle | 0.5745 |  |
| Weighted-mean OD in tract anterior thalamic radiation (right) | -0.5529 |  |
| Mean L1 in anterior limb of internal capsule on FA skeleton (right) | 0.5410 |  |
| Volume of thalamus (right) | -0.5232 |  |
| Weighted-mean FA in tract forceps minor | -0.5113 |  |
| Mean FA in cerebral peduncle on FA skeleton (left) | -0.4964 |  |
| Volume of grey matter in Putamen (left) | 0.4557 |  |
| Mean L1 in middle cerebellar peduncle on FA skeleton | -0.4240 |  |
| Volume of grey matter in Insular Cortex (left) | 0.4107 |  |
| Volume of grey matter in VI Cerebellum (left) | -0.4004 |  |
| Volume of putamen (left) | -0.3795 |  |
| Median T2star in putamen (left) | -0.3773 |  |
| Volume of grey matter in IX Cerebellum (left) | 0.3740 |  |
| Mean L1 in anterior limb of internal capsule on FA skeleton (left) | 0.3556 |  |
| Mean MO in fornix cres+stria terminalis on FA skeleton (left) | -0.3367 |  |
| Weighted-mean OD in tract posterior thalamic radiation (right) | -0.3219 |  |
| Mean ICVF in superior longitudinal fasciculus on FA skeleton (right) | 0.3088 |  |
| Mean MO in fornix on FA skeleton | 0.3075 |  |
| Weighted-mean ISOVF in tract uncinate fasciculus (left) | 0.2999 |  |
| Volume of grey matter in Frontal Operculum Cortex (right) | -0.2969 |  |
| Mean L3 in posterior thalamic radiation on FA skeleton (right) | 0.2762 |  |
| Volume of thalamus (left) | -0.2757 |  |
| Mean L2 in fornix cres+stria terminalis on FA skeleton (left) | 0.2661 |  |
| Mean OD in posterior limb of internal capsule on FA skeleton (right) | 0.2650 |  |
| Mean FA in body of corpus callosum on FA skeleton | 0.2633 |  |
| Weighted-mean MO in tract acoustic radiation (left) | -0.2515 |  |
| Volume of grey matter in Heschl's Gyrus (includes H1 and H2) (right) | -0.2482 |  |
| Mean L1 in genu of corpus callosum on FA skeleton | 0.2473 |  |
| Mean L2 in splenium of corpus callosum on FA skeleton | -0.2430 |  |
| Weighted-mean ISOVF in tract forceps minor | -0.2387 |  |
| Volume of putamen (right) | -0.2383 |  |
| Mean OD in anterior limb of internal capsule on FA skeleton (right) | 0.2364 |  |
| Weighted-mean ISOVF in tract superior thalamic radiation (right) | 0.2271 |  |
| Volume of grey matter in Lateral Occipital Cortex, inferior division (left) | 0.2193 |  |
| Mean ICVF in body of corpus callosum on FA skeleton | 0.2128 |  |
| rfMRI partial correlation matrix, dimension 252 (element 180) | 0.2090 |  |
| Mean ICVF in tapetum on FA skeleton (right) ^2^ | -0.2087 |  |
| Median z-statistic (in group-defined amygdala activation mask) for faces-shapes contrast | -0.2082 |  |
| Mean OD in pontine crossing tract on FA skeleton | 0.2078 |  |
| Mean L3 in retrolenticular part of internal capsule on FA skeleton (left) | -0.2076 |  |
| Weighted-mean ISOVF in tract superior longitudinal fasciculus (right) | 0.2068 |  |
| Mean ISOVF in superior corona radiata on FA skeleton (right) | 0.1990 |  |
| rfMRI partial correlation matrix, dimension 25 (element 127) | 0.1965 |  |
| Mean L1 in sagittal stratum on FA skeleton (right) | -0.1930 |  |
| Mean ICVF in fornix cres+stria terminalis on FA skeleton (right) | -0.1921 |  |
| Weighted-mean L3 in tract medial lemniscus (right) | -0.1885 |  |
| rfMRI partial correlation matrix, dimension 25 (element 44) | -0.1877 |  |
| Volume of grey matter in Inferior Temporal Gyrus, temporooccipital part (left) | 0.1851 |  |
| Mean ICVF in retrolenticular part of internal capsule on FA skeleton (right) | 0.1833 |  |
| Mean FA in superior cerebellar peduncle on FA skeleton (left) | 0.1828 |  |
| rfMRI partial correlation matrix, dimension 25 (element 172) | 0.1826 |  |
| Weighted-mean L1 in tract parahippocampal part of cingulum (left) | -0.1823 |  |
| Weighted-mean ICVF in tract superior longitudinal fasciculus (right) | 0.1801 |  |
| Mean OD in superior cerebellar peduncle on FA skeleton (left) | -0.1790 |  |
| Volume of grey matter in Frontal Orbital Cortex (left) | -0.1696 |  |
| Volume of grey matter in X Cerebellum (left) | -0.1652 |  |
| Volume of grey matter in Paracingulate Gyrus (right) | -0.1651 |  |
| Weighted-mean L1 in tract corticospinal tract (left) | -0.1642 |  |
| Mean ICVF in medial lemniscus on FA skeleton (left) | 0.1599 |  |
| rfMRI partial correlation matrix, dimension 25 (element 22) | 0.1593 |  |
| Volume of grey matter in Hippocampus (left) | -0.1592 |  |
| Weighted-mean FA in tract middle cerebellar peduncle | -0.1569 |  |
| Mean ICVF in corticospinal tract on FA skeleton (right) | 0.1564 |  |
| 90th percentile of BOLD effect (in group-defined mask) for faces-shapes contrast | -0.1537 |  |
| Volume of grey matter in Crus II Cerebellum (vermis) | 0.1511 |  |
| rfMRI partial correlation matrix, dimension 25 (element 96) | 0.1498 |  |
| Weighted-mean ICVF in tract middle cerebellar peduncle | -0.1496 |  |
| Weighted-mean OD in tract anterior thalamic radiation (left) | -0.1485 |  |
| Mean L1 in superior corona radiata on FA skeleton (left) | 0.1481 |  |
| rfMRI partial correlation matrix, dimension 25 (element 151) | -0.1473 |  |
| rfMRI partial correlation matrix, dimension 25 (element 131) | -0.1440 |  |
| Mean L1 in superior longitudinal fasciculus on FA skeleton (left) | -0.1422 |  |
| Mean ISOVF in cingulum cingulate gyrus on FA skeleton (right) | -0.1402 |  |
| rfMRI partial correlation matrix, dimension 25 (element 89) | 0.1397 |  |
| Mean ICVF in corticospinal tract on FA skeleton (left) | 0.1388 |  |
| Volume of grey matter in Frontal Operculum Cortex (left) | -0.1377 |  |
| rfMRI partial correlation matrix, dimension 25 (element 46) | -0.1336 |  |
| Volume of grey matter in X Cerebellum (right) | -0.1322 |  |
| Mean FA in fornix on FA skeleton | -0.1321 |  |
| Median T2star in thalamus (left) | 0.1312 |  |
| Mean FA in corticospinal tract on FA skeleton (right) | 0.1299 |  |
| Weighted-mean MO in tract acoustic radiation (right) | -0.1284 |  |
| Volume of grey matter in Lingual Gyrus (right) | -0.1282 |  |
| Mean L1 in cerebral peduncle on FA skeleton (right) | -0.1270 |  |
| rfMRI partial correlation matrix, dimension 25 (element 146) | 0.1266 |  |
| rfMRI partial correlation matrix, dimension 25 (element 135) | -0.1256 |  |
| Weighted-mean L1 in tract corticospinal tract (right) | -0.1253 |  |
| Volume of grey matter in Angular Gyrus (left) | 0.1231 |  |
| Mean L2 in cingulum cingulate gyrus on FA skeleton (right) | -0.1207 |  |
| Volume of grey matter in Supramarginal Gyrus, anterior division (right) | 0.1195 |  |
| Volume of grey matter in Crus I Cerebellum (vermis) | -0.1187 |  |
| Volume of grey matter in Planum Polare (left) | -0.1185 |  |
| Volume of grey matter in Pallidum (left) | 0.1175 |  |
| Volume of grey matter in Planum Polare (right) | -0.1166 |  |
| Weighted-mean MO in tract forceps major | -0.1160 |  |
| Mean ICVF in posterior corona radiata on FA skeleton (left) | 0.1153 |  |
| Weighted-mean ICVF in tract anterior thalamic radiation (right) | -0.1150 |  |
| Volume of grey matter in Frontal Orbital Cortex (right) | -0.1143 |  |
| rfMRI partial correlation matrix, dimension 25 (element 159) | 0.1122 |  |
| rfMRI partial correlation matrix, dimension 25 (element 161) | -0.1119 |  |
| Weighted-mean OD in tract superior longitudinal fasciculus (left) | 0.1116 |  |
| Weighted-mean L2 in tract posterior thalamic radiation (right) | 0.1114 |  |
| rfMRI partial correlation matrix, dimension 25 (element 49) | -0.1111 |  |
| Weighted-mean ICVF in tract corticospinal tract (right) | 0.1107 |  |
| rfMRI partial correlation matrix, dimension 25 (element 202) | 0.1087 |  |
| Weighted-mean L1 in tract acoustic radiation (left) | -0.1083 |  |
| Mean MD in cingulum cingulate gyrus on FA skeleton (right) | -0.1082 |  |
| Volume of grey matter in Frontal Medial Cortex (right) | -0.1079 |  |
| Mean L1 in superior corona radiata on FA skeleton (right) | 0.1078 |  |
| rfMRI partial correlation matrix, dimension 25 (element 124) | -0.1065 |  |
| Mean FA in middle cerebellar peduncle on FA skeleton | -0.1061 |  |
| Volume of grey matter in Occipital Fusiform Gyrus (left) | 0.1054 |  |
| 90th percentile of z-statistic (in group-defined amygdala activation mask) for faces-shapes contrast | -0.1052 |  |
| rfMRI partial correlation matrix, dimension 25 (element 58) | 0.1025 |  |
| Mean ISOVF in inferior cerebellar peduncle on FA skeleton (left) | -0.1009 |  |
| Mean MO in medial lemniscus on FA skeleton (right) | 0.1004 |  |
| Mean FA in fornix cres+stria terminalis on FA skeleton (right) | -0.0995 |  |
| Volume of grey matter in Thalamus (right) | 0.0991 |  |
| Mean ICVF in posterior thalamic radiation on FA skeleton (left) | -0.0989 |  |
| Weighted-mean OD in tract posterior thalamic radiation (left) | -0.0978 |  |
| rfMRI partial correlation matrix, dimension 25 (element 66) | -0.0954 |  |
| rfMRI partial correlation matrix, dimension 25 (element 60) | -0.0949 |  |
| Mean OD in superior cerebellar peduncle on FA skeleton (right) | -0.0947 |  |
| Mean MO in medial lemniscus on FA skeleton (left) | 0.0945 |  |
| rfMRI partial correlation matrix, dimension 25 (element 155) | 0.0934 |  |
| rfMRI partial correlation matrix, dimension 25 (element 144) | -0.0924 |  |
| Weighted-mean MO in tract cingulate gyrus part of cingulum (left) | -0.0910 |  |
| Volume of grey matter in Inferior Temporal Gyrus, posterior division (right) | -0.0895 |  |
| rfMRI partial correlation matrix, dimension 25 (element 156) | 0.0885 |  |
| Weighted-mean MD in tract corticospinal tract (right) | -0.0885 |  |
| rfMRI partial correlation matrix, dimension 25 (element 130) | -0.0881 |  |
| Volume of grey matter in Crus II Cerebellum (right) | -0.0856 |  |
| Volume of grey matter in VIIIb Cerebellum (vermis) | 0.0850 |  |
| Median T2star in thalamus (right) | 0.0843 |  |
| Volume of grey matter in Supramarginal Gyrus, posterior division (right) | -0.0840 |  |
| rfMRI partial correlation matrix, dimension 25 (element 103) | -0.0840 |  |
| Mean MD in uncinate fasciculus on FA skeleton (right) | 0.0835 |  |
| Mean ICVF in superior fronto-occipital fasciculus on FA skeleton (right) | -0.0832 |  |
| rfMRI partial correlation matrix, dimension 25 (element 204) | 0.0819 |  |
| Weighted-mean ISOVF in tract middle cerebellar peduncle | -0.0816 |  |
| Mean MO in fornix cres+stria terminalis on FA skeleton (right) | -0.0807 |  |
| Mean L1 in superior fronto-occipital fasciculus on FA skeleton (left) | 0.0799 |  |
| Mean MO in external capsule on FA skeleton (right) | -0.0797 |  |
| Mean OD in fornix on FA skeleton | -0.0796 |  |
| rfMRI partial correlation matrix, dimension 25 (element 25) | -0.0790 |  |
| rfMRI partial correlation matrix, dimension 25 (element 121) | -0.0781 |  |
| Weighted-mean ISOVF in tract parahippocampal part of cingulum (right) | 0.0780 |  |
| rfMRI partial correlation matrix, dimension 25 (element 97) | 0.0779 |  |
| rfMRI partial correlation matrix, dimension 25 (element 125) | 0.0772 |  |
| Mean ICVF in inferior cerebellar peduncle on FA skeleton (left) | -0.0749 |  |
| Volume of grey matter in Planum Temporale (left) | -0.0745 |  |
| Mean FA in inferior cerebellar peduncle on FA skeleton (left) | -0.0728 |  |
| Weighted-mean MO in tract posterior thalamic radiation (left) | -0.0724 |  |
| Mean OD in posterior thalamic radiation on FA skeleton (right) | 0.0715 |  |
| rfMRI partial correlation matrix, dimension 25 (element 84) | -0.0707 |  |
| rfMRI partial correlation matrix, dimension 25 (element 55) | 0.0682 |  |
| rfMRI partial correlation matrix, dimension 25 (element 142) | -0.0678 |  |
| 90th percentile of BOLD effect (in group-defined amygdala activation mask) for faces-shapes contrast | 0.0666 |  |
| Mean OD in posterior limb of internal capsule on FA skeleton (left) | 0.0662 |  |
| Mean FA in anterior limb of internal capsule on FA skeleton (left) | 0.0661 |  |
| rfMRI partial correlation matrix, dimension 25 (element 101) | 0.0657 |  |
| rfMRI partial correlation matrix, dimension 25 (element 31) | 0.0647 |  |
| rfMRI partial correlation matrix, dimension 25 (element 205) | 0.0644 |  |
| rfMRI partial correlation matrix, dimension 25 (element 35) | -0.0634 |  |
| Weighted-mean L1 in tract superior thalamic radiation (right) | 0.0622 |  |
| Mean MD in retrolenticular part of internal capsule on FA skeleton (right) | -0.0617 |  |
| Weighted-mean MO in tract medial lemniscus (left) | -0.0614 |  |
| Mean OD in external capsule on FA skeleton (right) | 0.0612 |  |
| Mean ISOVF in posterior limb of internal capsule on FA skeleton (left) | -0.0589 |  |
| Median T2star in accumbens (left) | -0.0570 |  |
| Weighted-mean ISOVF in tract anterior thalamic radiation (left) | -0.0569 |  |
| rfMRI partial correlation matrix, dimension 25 (element 169) | 0.0560 |  |
| Mean MO in inferior cerebellar peduncle on FA skeleton (right) | -0.0556 |  |
| rfMRI partial correlation matrix, dimension 25 (element 92) | -0.0552 |  |
| Mean OD in cingulum cingulate gyrus on FA skeleton (left) | -0.0534 |  |
| rfMRI partial correlation matrix, dimension 25 (element 94) | 0.0528 |  |
| Mean OD in uncinate fasciculus on FA skeleton (right) | 0.0502 |  |
| Volume of grey matter in Cingulate Gyrus, anterior division (left) | -0.0490 |  |
| Weighted-mean OD in tract uncinate fasciculus (right) | -0.0490 |  |
| Weighted-mean OD in tract parahippocampal part of cingulum (right) | 0.0468 |  |
| Mean OD in anterior corona radiata on FA skeleton (right) | -0.0465 |  |
| Weighted-mean ISOVF in tract posterior thalamic radiation (right) | 0.0450 |  |
| rfMRI partial correlation matrix, dimension 25 (element 21) | -0.0448 |  |
| rfMRI partial correlation matrix, dimension 25 (element 194) | 0.0446 |  |
| Volume of grey matter in VI Cerebellum (right) | -0.0435 |  |
| rfMRI partial correlation matrix, dimension 25 (element 128) | 0.0433 |  |
| Mean ISOVF in cingulum hippocampus on FA skeleton (left) | 0.0425 |  |
| Mean OD in corticospinal tract on FA skeleton (left) | -0.0416 |  |
| rfMRI partial correlation matrix, dimension 25 (element 143) | 0.0412 |  |
| Mean MD in cingulum hippocampus on FA skeleton (right) | -0.0410 |  |
| rfMRI partial correlation matrix, dimension 25 (element 39) | -0.0407 |  |
| Volume of amygdala (left) | 0.0403 |  |
| rfMRI partial correlation matrix, dimension 25 (element 51) | -0.0399 |  |
| Mean L2 in posterior corona radiata on FA skeleton (left) | -0.0396 |  |
| rfMRI partial correlation matrix, dimension 25 (element 210) | 0.0390 |  |
| rfMRI partial correlation matrix, dimension 25 (element 95) | -0.0380 |  |
| Mean L2 in fornix cres+stria terminalis on FA skeleton (right) | 0.0377 |  |
| rfMRI partial correlation matrix, dimension 25 (element 50) | -0.0375 |  |
| rfMRI partial correlation matrix, dimension 25 (element 15) | 0.0372 |  |
| Weighted-mean MO in tract superior longitudinal fasciculus (right) | -0.0366 |  |
| Weighted-mean MD in tract superior thalamic radiation (left) | 0.0364 |  |
| Mean OD in cerebral peduncle on FA skeleton (right) | 0.0360 |  |
| Mean ISOVF in anterior limb of internal capsule on FA skeleton (right) | 0.0348 |  |
| Volume of grey matter in Subcallosal Cortex (right) | 0.0343 |  |
| Volume of grey matter in VIIb Cerebellum (vermis) | -0.0337 |  |
| Volume of grey matter in Occipital Pole (left) | -0.0329 |  |
| rfMRI partial correlation matrix, dimension 25 (element 189) | 0.0328 |  |
| Median T2star in pallidum (right) | 0.0326 |  |
| rfMRI partial correlation matrix, dimension 25 (element 183) | 0.0325 |  |
| Mean MD in cingulum cingulate gyrus on FA skeleton (left) | -0.0322 |  |
| Mean L1 in pontine crossing tract on FA skeleton | -0.0321 |  |
| rfMRI partial correlation matrix, dimension 25 (element 140) | -0.0305 |  |
| rfMRI partial correlation matrix, dimension 25 (element 75) | 0.0302 |  |
| rfMRI partial correlation matrix, dimension 25 (element 5) | 0.0301 |  |
| Volume of grey matter in Brain-Stem | -0.0301 |  |
| rfMRI partial correlation matrix, dimension 25 (element 176) | 0.0294 |  |
| rfMRI partial correlation matrix, dimension 25 (element 85) | -0.0294 |  |
| Mean L3 in posterior limb of internal capsule on FA skeleton (left) | -0.0290 |  |
| rfMRI partial correlation matrix, dimension 25 (element 82) | -0.0284 |  |
| rfMRI partial correlation matrix, dimension 25 (element 123) | -0.0283 |  |
| 90th percentile of BOLD effect (in group-defined mask) for shapes activation | 0.0279 |  |
| Volume of grey matter in Lingual Gyrus (left) | -0.0278 |  |
| rfMRI partial correlation matrix, dimension 25 (element 26) | 0.0269 |  |
| Mean ICVF in superior cerebellar peduncle on FA skeleton (right) | 0.0266 |  |
| Weighted-mean OD in tract inferior longitudinal fasciculus (right) | 0.0264 |  |
| rfMRI partial correlation matrix, dimension 25 (element 141) | 0.0259 |  |
| rfMRI partial correlation matrix, dimension 25 (element 106) | -0.0257 |  |
| rfMRI partial correlation matrix, dimension 25 (element 16) | 0.0245 |  |
| rfMRI partial correlation matrix, dimension 25 (element 112) | 0.0243 |  |
| Volume of grey matter in I-IV Cerebellum (right) | -0.0236 |  |
| rfMRI partial correlation matrix, dimension 25 (element 116) | -0.0235 |  |
| Weighted-mean ISOVF in tract anterior thalamic radiation (right) | -0.0232 |  |
| Median BOLD effect (in group-defined mask) for faces activation | 0.0229 |  |
| rfMRI partial correlation matrix, dimension 25 (element 64) | -0.0224 |  |
| Volume of grey matter in IX Cerebellum (right) | 0.0214 |  |
| Volume of grey matter in Temporal Fusiform Cortex, posterior division (left) | -0.0212 |  |
| rfMRI partial correlation matrix, dimension 25 (element 196) | -0.0201 |  |
| Weighted-mean MO in tract posterior thalamic radiation (right) | -0.0199 |  |
| rfMRI partial correlation matrix, dimension 25 (element 29) | 0.0190 |  |
| rfMRI partial correlation matrix, dimension 25 (element 71) | 0.0189 |  |
| Mean L2 in posterior limb of internal capsule on FA skeleton (right) | 0.0187 |  |
| rfMRI partial correlation matrix, dimension 25 (element 56) | -0.0180 |  |
| rfMRI partial correlation matrix, dimension 25 (element 17) | -0.0175 |  |
| Volume of grey matter in Inferior Temporal Gyrus, anterior division (right) | -0.0168 |  |
| Volume of grey matter in Juxtapositional Lobule Cortex (formerly Supplementary Motor Cortex) (left) | -0.0155 |  |
| Mean L2 in sagittal stratum on FA skeleton (left) | -0.0150 |  |
| rfMRI partial correlation matrix, dimension 25 (element 164) | 0.0149 |  |
| rfMRI partial correlation matrix, dimension 25 (element 133) | 0.0144 |  |
| Volume of grey matter in Lateral Occipital Cortex, superior division (right) | -0.0140 |  |
| rfMRI partial correlation matrix, dimension 25 (element 107) | 0.0136 |  |
| Volume of grey matter in Cuneal Cortex (right) | 0.0131 |  |
| Mean L1 in cerebral peduncle on FA skeleton (left) | -0.0126 |  |
| Volume of grey matter in Superior Temporal Gyrus, posterior division (left) | -0.0125 |  |
| Volume of grey matter in Supramarginal Gyrus, anterior division (left) | 0.0123 |  |
| rfMRI partial correlation matrix, dimension 25 (element 113) | -0.0123 |  |
| Mean L2 in retrolenticular part of internal capsule on FA skeleton (right) | -0.0121 |  |
| rfMRI partial correlation matrix, dimension 25 (element 80) | 0.0118 |  |
| Mean OD in retrolenticular part of internal capsule on FA skeleton (left) | 0.0109 |  |
| Mean MO in cerebral peduncle on FA skeleton (left) | -0.0108 |  |
| Volume of grey matter in Superior Parietal Lobule (left) | -0.0106 |  |
| rfMRI partial correlation matrix, dimension 25 (element 98) | 0.0094 |  |
| Weighted-mean OD in tract cingulate gyrus part of cingulum (left) | 0.0089 |  |
| Mean L1 in uncinate fasciculus on FA skeleton (right) | 0.0084 |  |
| rfMRI partial correlation matrix, dimension 25 (element 7) | -0.0081 |  |
| Weighted-mean OD in tract uncinate fasciculus (left) | 0.0079 |  |
| rfMRI partial correlation matrix, dimension 25 (element 197) | -0.0077 |  |
| Median T2star in pallidum (left) | 0.0067 |  |
| rfMRI partial correlation matrix, dimension 25 (element 190) | 0.0066 |  |
| rfMRI partial correlation matrix, dimension 25 (element 206) | 0.0063 |  |
| Weighted-mean MO in tract forceps minor | -0.0062 |  |
| Volume of grey matter in Frontal Pole (left) | -0.0060 |  |
| Mean L3 in posterior thalamic radiation on FA skeleton (left) | 0.0059 |  |
| rfMRI partial correlation matrix, dimension 25 (element 136) | -0.0057 |  |
| rfMRI partial correlation matrix, dimension 25 (element 179) | 0.0053 |  |
| Mean MO in cingulum hippocampus on FA skeleton (right) | 0.0044 |  |
| Mean OD in superior fronto-occipital fasciculus on FA skeleton (left) | -0.0034 |  |
| Weighted-mean OD in tract acoustic radiation (left) | 0.0023 |  |
| rfMRI partial correlation matrix, dimension 25 (element 186) | 0.0019 |  |
| Volume of grey matter in VI Cerebellum (vermis) | -0.0013 |  |
| rfMRI partial correlation matrix, dimension 25 (element 87) | -0.0008 |  |
| ^1^ The phenotypes with non-zero coefficient values have been selectively listed.  ^2^ A partial correlation matrix with 25 dimensionalities (field ID: 25752), which had been converted into vectors with 210 elements. | |  |
|  |  |  |

**Table S7.** β coefficients and 95% confidence intervals (CIs) for the association between metabolites and brain age: results from linear regression models

| **Metabolites** | **Basic-model ^†^** | |  |  |  | **Multi-model ^‡^** | |  |  |  |
| --- | --- | --- | --- | --- | --- | --- | --- | --- | --- | --- |
|  | β | Lower | Upper | *P*-value | FDR-q | β | Lower | Upper | *P*-value | FDR-q |
| **Amino acids & Glycolysis** | |  |  |  |  |  |  |  |  |  |
| Valine | 0.141 | 0.056 | 0.226 | 0.001 | 0.002 | 0.070 | -0.026 | 0.165 | 0.153 | 0.321 |
| Leucine | 0.118 | 0.032 | 0.204 | 0.007 | 0.012 | 0.063 | -0.032 | 0.158 | 0.192 | 0.383 |
| Isoleucine | 0.071 | -0.013 | 0.155 | 0.099 | 0.126 | 0.022 | -0.070 | 0.115 | 0.638 | 0.819 |
| Phenylalanine | 0.113 | 0.031 | 0.196 | 0.007 | 0.012 | 0.074 | -0.017 | 0.164 | 0.111 | 0.251 |
| Tyrosine | 0.242 | 0.159 | 0.325 | <0.001 | <0.001 | 0.198 | 0.106 | 0.290 | <0.001 | 0.001 |
| Alanine | 0.085 | 0.001 | 0.168 | 0.046 | 0.062 | 0.021 | -0.070 | 0.112 | 0.650 | 0.822 |
| Glutamine | -0.298 | -0.381 | -0.215 | <0.001 | <0.001 | -0.222 | -0.314 | -0.130 | <0.001 | <0.001 |
| Glycine | -0.079 | -0.167 | 0.009 | 0.079 | 0.103 | -0.008 | -0.105 | 0.089 | 0.874 | 0.970 |
| Histidine | -0.073 | -0.157 | 0.010 | 0.084 | 0.108 | -0.037 | -0.127 | 0.053 | 0.418 | 0.648 |
| BCAAs | 0.124 | 0.039 | 0.210 | 0.004 | 0.007 | 0.060 | -0.035 | 0.155 | 0.216 | 0.419 |
| Lactate | 0.055 | -0.028 | 0.138 | 0.193 | 0.229 | -0.019 | -0.109 | 0.072 | 0.686 | 0.849 |
| Pyruvate | 0.003 | -0.080 | 0.086 | 0.944 | 0.948 | -0.044 | -0.134 | 0.046 | 0.340 | 0.565 |
| Glucose | 0.311 | 0.228 | 0.394 | <0.001 | <0.001 | 0.152 | 0.055 | 0.249 | 0.002 | 0.013 |
| Citrate | -0.129 | -0.213 | -0.044 | 0.003 | 0.005 | -0.067 | -0.160 | 0.025 | 0.153 | 0.321 |
| **Apo-LP, LP size & Other lipids** | |  |  |  |  |  |  |  |  |  |
| ApoA1 | 0.111 | 0.020 | 0.201 | 0.016 | 0.024 | 0.177 | 0.075 | 0.278 | 0.001 | 0.006 |
| ApoB | -0.109 | -0.192 | -0.026 | 0.010 | 0.016 | -0.050 | -0.148 | 0.048 | 0.318 | 0.542 |
| ApoB/ApoA1 | -0.145 | -0.230 | -0.060 | 0.001 | 0.002 | -0.135 | -0.235 | -0.035 | 0.008 | 0.036 |
| HDL-AD | -0.197 | -0.291 | -0.102 | <0.001 | <0.001 | 0.002 | -0.110 | 0.113 | 0.978 | 0.991 |
| LDL-AD | -0.205 | -0.291 | -0.120 | <0.001 | <0.001 | -0.141 | -0.237 | -0.046 | 0.004 | 0.020 |
| VLDL-AD | 0.207 | 0.117 | 0.297 | <0.001 | <0.001 | 0.047 | -0.057 | 0.152 | 0.375 | 0.595 |
| Phosphatidylcholine | 0.096 | 0.008 | 0.184 | 0.033 | 0.045 | 0.139 | 0.041 | 0.237 | 0.005 | 0.028 |
| Phosphoglycerides | 0.128 | 0.041 | 0.215 | 0.004 | 0.007 | 0.150 | 0.053 | 0.247 | 0.002 | 0.014 |
| Sphingomyeline | -0.071 | -0.159 | 0.016 | 0.111 | 0.139 | 0.048 | -0.053 | 0.149 | 0.350 | 0.578 |
| Total choline | 0.069 | -0.019 | 0.156 | 0.124 | 0.154 | 0.122 | 0.024 | 0.221 | 0.015 | 0.054 |
| TG/Phosphoglycerides | 0.206 | 0.116 | 0.295 | <0.001 | <0.001 | 0.039 | -0.066 | 0.145 | 0.465 | 0.688 |
| **Cholesterol** |  |  |  |  |  |  |  |  |  |  |
| XS-VLDL | -0.122 | -0.207 | -0.037 | 0.005 | 0.008 | -0.031 | -0.130 | 0.069 | 0.549 | 0.755 |
| S-VLDL | -0.065 | -0.148 | 0.018 | 0.127 | 0.156 | -0.057 | -0.154 | 0.039 | 0.244 | 0.459 |
| M-VLDL | -0.194 | -0.276 | -0.111 | <0.001 | <0.001 | -0.115 | -0.213 | -0.018 | 0.020 | 0.063 |
| L-VLDL | 0.067 | -0.019 | 0.152 | 0.125 | 0.154 | -0.010 | -0.109 | 0.089 | 0.846 | 0.954 |
| VL-VLDL | 0.089 | 0.003 | 0.174 | 0.042 | 0.057 | 0.010 | -0.090 | 0.110 | 0.846 | 0.954 |
| XL-VLDL | 0.208 | 0.123 | 0.294 | <0.001 | <0.001 | 0.097 | -0.002 | 0.196 | 0.055 | 0.138 |
| VLDL | -0.033 | -0.117 | 0.050 | 0.432 | 0.472 | -0.034 | -0.131 | 0.064 | 0.499 | 0.702 |
| S-LDL | -0.084 | -0.167 | -0.001 | 0.047 | 0.063 | -0.027 | -0.124 | 0.071 | 0.591 | 0.787 |
| M-LDL | -0.044 | -0.127 | 0.039 | 0.302 | 0.351 | 0.001 | -0.095 | 0.098 | 0.981 | 0.991 |
| L-LDL | -0.123 | -0.206 | -0.040 | 0.004 | 0.006 | -0.024 | -0.121 | 0.073 | 0.629 | 0.819 |
| Clinical LDL | -0.126 | -0.209 | -0.043 | 0.003 | 0.005 | -0.029 | -0.127 | 0.068 | 0.554 | 0.758 |
| LDL | -0.099 | -0.182 | -0.016 | 0.019 | 0.027 | -0.018 | -0.114 | 0.079 | 0.720 | 0.871 |
| IDL | -0.142 | -0.227 | -0.057 | 0.001 | 0.002 | <0.001 | -0.101 | 0.101 | 0.999 | 0.999 |
| S-HDL | 0.199 | 0.116 | 0.282 | <0.001 | <0.001 | 0.160 | 0.069 | 0.252 | 0.001 | 0.006 |
| M-HDL | 0.109 | 0.019 | 0.199 | 0.018 | 0.026 | 0.178 | 0.076 | 0.280 | 0.001 | 0.006 |
| L-HDL | -0.198 | -0.293 | -0.104 | <0.001 | <0.001 | 0.003 | -0.109 | 0.114 | 0.961 | 0.991 |
| XL-HDL | -0.278 | -0.370 | -0.186 | <0.001 | <0.001 | -0.059 | -0.168 | 0.051 | 0.293 | 0.518 |
| HDL | -0.040 | -0.133 | 0.052 | 0.393 | 0.433 | 0.110 | 0.003 | 0.217 | 0.044 | 0.115 |
| Total | -0.102 | -0.187 | -0.017 | 0.018 | 0.026 | 0.015 | -0.084 | 0.114 | 0.761 | 0.899 |
| Remnant | -0.089 | -0.172 | -0.006 | 0.036 | 0.049 | -0.020 | -0.119 | 0.078 | 0.685 | 0.849 |
| Total-HDLC | -0.096 | -0.179 | -0.013 | 0.023 | 0.033 | -0.019 | -0.117 | 0.078 | 0.697 | 0.855 |
| **Cholesterol to Total lipids** | |  |  |  |  |  |  |  |  |  |
| VS-VLDL | -0.370 | -0.457 | -0.284 | <0.001 | <0.001 | -0.139 | -0.244 | -0.033 | 0.010 | 0.041 |
| S-VLDL | -0.279 | -0.363 | -0.195 | <0.001 | <0.001 | -0.120 | -0.218 | -0.023 | 0.016 | 0.056 |
| M-VLDL | -0.351 | -0.438 | -0.264 | <0.001 | <0.001 | -0.136 | -0.240 | -0.032 | 0.011 | 0.043 |
| L-VLDL | -0.181 | -0.265 | -0.097 | <0.001 | <0.001 | -0.078 | -0.173 | 0.016 | 0.103 | 0.236 |
| XL-VLDL | -0.244 | -0.329 | -0.158 | <0.001 | <0.001 | -0.088 | -0.186 | 0.010 | 0.079 | 0.185 |
| XXL-VLDL | -0.051 | -0.134 | 0.032 | 0.227 | 0.266 | -0.006 | -0.096 | 0.085 | 0.904 | 0.970 |
| S-LDL | -0.154 | -0.237 | -0.072 | <0.001 | 0.001 | -0.034 | -0.127 | 0.059 | 0.477 | 0.689 |
| M-LDL | -0.276 | -0.359 | -0.193 | <0.001 | <0.001 | -0.116 | -0.211 | -0.021 | 0.017 | 0.057 |
| L-LDL | -0.361 | -0.444 | -0.277 | <0.001 | <0.001 | -0.160 | -0.257 | -0.063 | 0.001 | 0.009 |
| IDL | -0.258 | -0.342 | -0.174 | <0.001 | <0.001 | -0.046 | -0.145 | 0.053 | 0.361 | 0.583 |
| S-HDL | -0.283 | -0.365 | -0.200 | <0.001 | <0.001 | -0.137 | -0.229 | -0.044 | 0.004 | 0.021 |
| M-HDL | -0.248 | -0.334 | -0.161 | <0.001 | <0.001 | -0.077 | -0.178 | 0.024 | 0.133 | 0.290 |
| L-HDL | -0.426 | -0.513 | -0.338 | <0.001 | <0.001 | -0.200 | -0.306 | -0.094 | <0.001 | 0.004 |
| XL-HDL | 0.042 | -0.046 | 0.130 | 0.355 | 0.396 | 0.005 | -0.094 | 0.103 | 0.927 | 0.974 |
| **Cholesteryl esters to Total lipids** | |  |  |  |  |  |  |  |  |  |
| VS-VLDL | -0.361 | -0.448 | -0.274 | <0.001 | <0.001 | -0.121 | -0.227 | -0.015 | 0.025 | 0.075 |
| S-VLDL | -0.222 | -0.305 | -0.139 | <0.001 | <0.001 | -0.105 | -0.200 | -0.011 | 0.029 | 0.082 |
| M-VLDL | -0.357 | -0.445 | -0.270 | <0.001 | <0.001 | -0.141 | -0.246 | -0.037 | 0.008 | 0.035 |
| L-VLDL | -0.267 | -0.353 | -0.181 | <0.001 | <0.001 | -0.119 | -0.218 | -0.020 | 0.018 | 0.060 |
| XL-VLDL | -0.235 | -0.321 | -0.149 | <0.001 | <0.001 | -0.085 | -0.182 | 0.012 | 0.088 | 0.204 |
| XXL-VLDL | -0.065 | -0.148 | 0.017 | 0.121 | 0.152 | -0.021 | -0.111 | 0.069 | 0.645 | 0.819 |
| S-LDL | 0.132 | 0.048 | 0.215 | 0.002 | 0.004 | 0.085 | -0.009 | 0.179 | 0.078 | 0.184 |
| M-LDL | 0.094 | 0.009 | 0.178 | 0.030 | 0.041 | 0.034 | -0.061 | 0.130 | 0.481 | 0.689 |
| L-LDL | -0.155 | -0.238 | -0.073 | <0.001 | 0.001 | -0.103 | -0.193 | -0.013 | 0.025 | 0.075 |
| IDL | -0.170 | -0.254 | -0.086 | <0.001 | <0.001 | <0.001 | -0.097 | 0.097 | 0.995 | 0.999 |
| S-HDL | -0.225 | -0.308 | -0.142 | <0.001 | <0.001 | -0.121 | -0.212 | -0.029 | 0.010 | 0.041 |
| M-HDL | -0.241 | -0.326 | -0.157 | <0.001 | <0.001 | -0.099 | -0.196 | -0.001 | 0.048 | 0.120 |
| L-HDL | -0.424 | -0.510 | -0.338 | <0.001 | <0.001 | -0.214 | -0.318 | -0.110 | <0.001 | 0.001 |
| XL-HDL | -0.127 | -0.210 | -0.044 | 0.003 | 0.005 | -0.017 | -0.109 | 0.075 | 0.718 | 0.871 |
| **Cholestryl esters** |  |  |  |  |  |  |  |  |  |  |
| VS-VLDL | -0.146 | -0.231 | -0.061 | 0.001 | 0.002 | -0.035 | -0.136 | 0.066 | 0.499 | 0.702 |
| S-VLDL | -0.042 | -0.126 | 0.041 | 0.320 | 0.365 | -0.050 | -0.146 | 0.047 | 0.316 | 0.542 |
| M-VLDL | -0.251 | -0.334 | -0.168 | <0.001 | <0.001 | -0.141 | -0.239 | -0.043 | 0.005 | 0.026 |
| L-VLDL | <0.001 | -0.085 | 0.084 | 0.993 | 0.993 | -0.049 | -0.147 | 0.049 | 0.325 | 0.551 |
| VL-VLDL | 0.033 | -0.052 | 0.118 | 0.447 | 0.486 | -0.025 | -0.125 | 0.074 | 0.616 | 0.816 |
| XL-VLDL | 0.188 | 0.102 | 0.273 | <0.001 | <0.001 | 0.083 | -0.016 | 0.182 | 0.100 | 0.230 |
| VLDL | -0.078 | -0.161 | 0.005 | 0.064 | 0.085 | -0.054 | -0.152 | 0.043 | 0.276 | 0.505 |
| S-LDL | -0.043 | -0.126 | 0.040 | 0.308 | 0.356 | -0.007 | -0.104 | 0.091 | 0.891 | 0.970 |
| M-LDL | -0.012 | -0.095 | 0.072 | 0.782 | 0.808 | 0.015 | -0.082 | 0.112 | 0.766 | 0.899 |
| L-LDL | -0.108 | -0.191 | -0.025 | 0.011 | 0.017 | -0.021 | -0.117 | 0.076 | 0.672 | 0.840 |
| LDL | -0.077 | -0.160 | 0.006 | 0.069 | 0.090 | -0.010 | -0.106 | 0.086 | 0.837 | 0.952 |
| IDL | -0.131 | -0.216 | -0.046 | 0.003 | 0.005 | 0.006 | -0.095 | 0.108 | 0.902 | 0.970 |
| S-HDL | 0.185 | 0.102 | 0.268 | <0.001 | <0.001 | 0.146 | 0.054 | 0.237 | 0.002 | 0.012 |
| M-HDL | 0.106 | 0.016 | 0.197 | 0.020 | 0.029 | 0.174 | 0.073 | 0.276 | 0.001 | 0.007 |
| L-HDL | -0.208 | -0.302 | -0.114 | <0.001 | <0.001 | -0.006 | -0.117 | 0.106 | 0.922 | 0.973 |
| XL-HDL | -0.277 | -0.370 | -0.184 | <0.001 | <0.001 | -0.057 | -0.167 | 0.053 | 0.312 | 0.539 |
| HDL | -0.048 | -0.141 | 0.044 | 0.309 | 0.356 | 0.103 | -0.004 | 0.209 | 0.060 | 0.148 |
| Total | -0.099 | -0.184 | -0.014 | 0.022 | 0.032 | 0.024 | -0.075 | 0.123 | 0.636 | 0.819 |
| **Fatty acids** |  |  |  |  |  |  |  |  |  |  |
| DHA | -0.044 | -0.130 | 0.042 | 0.313 | 0.359 | 0.037 | -0.057 | 0.131 | 0.442 | 0.667 |
| LA | -0.102 | -0.186 | -0.019 | 0.016 | 0.024 | -0.039 | -0.134 | 0.056 | 0.419 | 0.648 |
| MUFA | 0.249 | 0.166 | 0.332 | <0.001 | <0.001 | 0.130 | 0.035 | 0.226 | 0.008 | 0.035 |
| n-3 | 0.042 | -0.043 | 0.128 | 0.329 | 0.373 | 0.056 | -0.037 | 0.148 | 0.236 | 0.453 |
| n-6 | -0.041 | -0.125 | 0.043 | 0.337 | 0.380 | -0.001 | -0.096 | 0.094 | 0.983 | 0.991 |
| PUFA | -0.024 | -0.108 | 0.061 | 0.584 | 0.614 | 0.015 | -0.079 | 0.110 | 0.749 | 0.892 |
| SFA | 0.238 | 0.155 | 0.321 | <0.001 | <0.001 | 0.176 | 0.081 | 0.270 | <0.001 | 0.004 |
| Total | 0.171 | 0.087 | 0.254 | <0.001 | <0.001 | 0.117 | 0.023 | 0.211 | 0.015 | 0.056 |
| Unsaturation | -0.276 | -0.362 | -0.190 | <0.001 | <0.001 | -0.119 | -0.216 | -0.021 | 0.017 | 0.057 |
| DHA/FA | -0.124 | -0.209 | -0.039 | 0.004 | 0.007 | -0.005 | -0.100 | 0.090 | 0.913 | 0.973 |
| LA/FA | -0.467 | -0.551 | -0.382 | <0.001 | <0.001 | -0.291 | -0.392 | -0.190 | <0.001 | <0.001 |
| MUFA/FA | 0.329 | 0.242 | 0.415 | <0.001 | <0.001 | 0.108 | 0.005 | 0.210 | 0.040 | 0.106 |
| n-3/FA | -0.026 | -0.111 | 0.059 | 0.546 | 0.579 | 0.026 | -0.067 | 0.118 | 0.586 | 0.787 |
| n-6/n-3 | -0.026 | -0.110 | 0.058 | 0.545 | 0.579 | -0.001 | -0.094 | 0.091 | 0.978 | 0.991 |
| n-6/FA | -0.423 | -0.508 | -0.338 | <0.001 | <0.001 | -0.267 | -0.366 | -0.167 | <0.001 | <0.001 |
| PUFA/MUFA | -0.354 | -0.440 | -0.268 | <0.001 | <0.001 | -0.147 | -0.249 | -0.045 | 0.005 | 0.026 |
| PUFA/FA | -0.422 | -0.507 | -0.337 | <0.001 | <0.001 | -0.251 | -0.352 | -0.151 | <0.001 | <0.001 |
| SFA/FA | 0.348 | 0.265 | 0.430 | <0.001 | <0.001 | 0.284 | 0.191 | 0.377 | <0.001 | <0.001 |
| **Free cholesterol** |  |  |  |  |  |  |  |  |  |  |
| VS-VLDL | -0.062 | -0.146 | 0.022 | 0.151 | 0.182 | -0.020 | -0.117 | 0.078 | 0.688 | 0.849 |
| S-VLDL | -0.103 | -0.186 | -0.020 | 0.015 | 0.023 | -0.070 | -0.167 | 0.027 | 0.155 | 0.321 |
| M-VLDL | -0.109 | -0.192 | -0.026 | 0.010 | 0.015 | -0.076 | -0.173 | 0.021 | 0.124 | 0.278 |
| L-VLDL | 0.131 | 0.045 | 0.217 | 0.003 | 0.005 | 0.029 | -0.071 | 0.130 | 0.567 | 0.772 |
| VL-VLDL | 0.144 | 0.059 | 0.230 | 0.001 | 0.002 | 0.046 | -0.054 | 0.146 | 0.363 | 0.583 |
| XL-VLDL | 0.230 | 0.145 | 0.316 | <0.001 | <0.001 | 0.111 | 0.013 | 0.209 | 0.027 | 0.079 |
| VLDL | 0.029 | -0.055 | 0.113 | 0.503 | 0.537 | -0.005 | -0.103 | 0.093 | 0.919 | 0.973 |
| S-LDL | -0.182 | -0.265 | -0.100 | <0.001 | <0.001 | -0.075 | -0.171 | 0.021 | 0.128 | 0.284 |
| M-LDL | -0.127 | -0.210 | -0.044 | 0.003 | 0.005 | -0.035 | -0.131 | 0.061 | 0.472 | 0.689 |
| L-LDL | -0.163 | -0.246 | -0.079 | <0.001 | <0.001 | -0.032 | -0.130 | 0.066 | 0.525 | 0.730 |
| LDL | -0.156 | -0.239 | -0.073 | <0.001 | <0.001 | -0.038 | -0.135 | 0.060 | 0.448 | 0.671 |
| IDL | -0.168 | -0.253 | -0.083 | <0.001 | <0.001 | -0.018 | -0.119 | 0.084 | 0.732 | 0.881 |
| S-HDL | 0.214 | 0.130 | 0.298 | <0.001 | <0.001 | 0.182 | 0.089 | 0.275 | <0.001 | 0.002 |
| M-HDL | 0.115 | 0.024 | 0.207 | 0.013 | 0.020 | 0.185 | 0.083 | 0.287 | <0.001 | 0.005 |
| L-HDL | -0.163 | -0.257 | -0.068 | 0.001 | 0.002 | 0.031 | -0.079 | 0.142 | 0.578 | 0.782 |
| XL-HDL | -0.266 | -0.356 | -0.176 | <0.001 | <0.001 | -0.062 | -0.167 | 0.044 | 0.253 | 0.467 |
| HDL | -0.011 | -0.105 | 0.082 | 0.811 | 0.831 | 0.130 | 0.024 | 0.237 | 0.017 | 0.057 |
| Total | -0.107 | -0.191 | -0.023 | 0.013 | 0.019 | -0.006 | -0.105 | 0.092 | 0.899 | 0.970 |
| **Free cholesterol to Total lipids** | |  |  |  |  |  |  |  |  |  |
| VS-VLDL | -0.339 | -0.421 | -0.256 | <0.001 | <0.001 | -0.194 | -0.290 | -0.099 | <0.001 | 0.001 |
| S-VLDL | -0.316 | -0.401 | -0.230 | <0.001 | <0.001 | -0.121 | -0.222 | -0.020 | 0.019 | 0.060 |
| M-VLDL | -0.318 | -0.404 | -0.232 | <0.001 | <0.001 | -0.112 | -0.215 | -0.009 | 0.032 | 0.090 |
| L-VLDL | 0.065 | -0.019 | 0.148 | 0.128 | 0.157 | 0.023 | -0.070 | 0.115 | 0.632 | 0.819 |
| XL-VLDL | -0.205 | -0.289 | -0.120 | <0.001 | <0.001 | -0.069 | -0.165 | 0.027 | 0.158 | 0.324 |
| XXL-VLDL | -0.006 | -0.089 | 0.077 | 0.888 | 0.895 | 0.031 | -0.062 | 0.123 | 0.515 | 0.720 |
| S-LDL | -0.303 | -0.387 | -0.219 | <0.001 | <0.001 | -0.130 | -0.225 | -0.035 | 0.008 | 0.035 |
| M-LDL | -0.335 | -0.421 | -0.250 | <0.001 | <0.001 | -0.140 | -0.240 | -0.041 | 0.006 | 0.029 |
| L-LDL | -0.345 | -0.431 | -0.259 | <0.001 | <0.001 | -0.108 | -0.210 | -0.006 | 0.039 | 0.104 |
| IDL | -0.290 | -0.373 | -0.207 | <0.001 | <0.001 | -0.105 | -0.199 | -0.011 | 0.028 | 0.080 |
| S-HDL | -0.230 | -0.317 | -0.143 | <0.001 | <0.001 | -0.051 | -0.151 | 0.048 | 0.309 | 0.538 |
| M-HDL | -0.143 | -0.236 | -0.050 | 0.003 | 0.005 | 0.071 | -0.038 | 0.179 | 0.201 | 0.395 |
| L-HDL | -0.226 | -0.315 | -0.138 | <0.001 | <0.001 | -0.020 | -0.126 | 0.085 | 0.703 | 0.859 |
| XL-HDL | 0.212 | 0.119 | 0.305 | <0.001 | <0.001 | 0.030 | -0.079 | 0.140 | 0.589 | 0.787 |
| **KB, FB & Inflammation** | |  |  |  |  |  |  |  |  |  |
| Glycoprotein acetyls | 0.309 | 0.225 | 0.392 | <0.001 | <0.001 | 0.169 | 0.072 | 0.266 | 0.001 | 0.006 |
| 3-Hydrocybutyrate | 0.108 | 0.025 | 0.191 | 0.011 | 0.017 | 0.118 | 0.028 | 0.208 | 0.010 | 0.042 |
| Acetate | -0.126 | -0.209 | -0.043 | 0.003 | 0.005 | -0.090 | -0.176 | -0.003 | 0.042 | 0.111 |
| Acetoacetate | 0.159 | 0.076 | 0.242 | <0.001 | <0.001 | 0.159 | 0.068 | 0.249 | 0.001 | 0.006 |
| Acetone | 0.109 | 0.026 | 0.192 | 0.010 | 0.015 | 0.143 | 0.053 | 0.234 | 0.002 | 0.012 |
| Albumin | 0.008 | -0.075 | 0.090 | 0.859 | 0.873 | 0.033 | -0.058 | 0.125 | 0.478 | 0.689 |
| Creatinine | -0.038 | -0.139 | 0.062 | 0.455 | 0.492 | -0.134 | -0.245 | -0.024 | 0.017 | 0.058 |
| **Lipoprotein particles** | |  |  |  |  |  |  |  |  |  |
| VS-VLDL | -0.030 | -0.113 | 0.054 | 0.486 | 0.521 | 0.001 | -0.096 | 0.098 | 0.979 | 0.991 |
| S-VLDL | 0.042 | -0.042 | 0.127 | 0.326 | 0.370 | -0.015 | -0.112 | 0.081 | 0.754 | 0.894 |
| M-VLDL | -0.054 | -0.137 | 0.030 | 0.206 | 0.243 | -0.052 | -0.149 | 0.045 | 0.292 | 0.518 |
| L-VLDL | 0.147 | 0.060 | 0.233 | 0.001 | 0.002 | 0.042 | -0.059 | 0.142 | 0.416 | 0.648 |
| VL-VLDL | 0.194 | 0.107 | 0.280 | <0.001 | <0.001 | 0.075 | -0.025 | 0.176 | 0.142 | 0.306 |
| XL-VLDL | 0.244 | 0.159 | 0.330 | <0.001 | <0.001 | 0.123 | 0.024 | 0.222 | 0.015 | 0.054 |
| VLDL | 0.023 | -0.061 | 0.106 | 0.598 | 0.625 | -0.006 | -0.103 | 0.091 | 0.900 | 0.970 |
| S-LDL | -0.095 | -0.179 | -0.012 | 0.024 | 0.034 | -0.040 | -0.138 | 0.057 | 0.418 | 0.648 |
| M-LDL | -0.060 | -0.143 | 0.023 | 0.155 | 0.187 | -0.011 | -0.108 | 0.087 | 0.830 | 0.952 |
| L-LDL | -0.153 | -0.236 | -0.070 | <0.001 | 0.001 | -0.089 | -0.186 | 0.008 | 0.073 | 0.175 |
| LDL | -0.125 | -0.207 | -0.042 | 0.003 | 0.006 | -0.064 | -0.161 | 0.033 | 0.197 | 0.390 |
| IDL | -0.108 | -0.191 | -0.025 | 0.011 | 0.017 | -0.011 | -0.111 | 0.088 | 0.822 | 0.948 |
| S-HDL | 0.212 | 0.129 | 0.295 | <0.001 | <0.001 | 0.159 | 0.067 | 0.251 | 0.001 | 0.006 |
| M-HDL | 0.143 | 0.053 | 0.233 | 0.002 | 0.004 | 0.190 | 0.090 | 0.291 | <0.001 | 0.004 |
| L-HDL | -0.177 | -0.272 | -0.083 | <0.001 | 0.001 | 0.016 | -0.095 | 0.127 | 0.778 | 0.905 |
| XL-HDL | -0.253 | -0.345 | -0.160 | <0.001 | <0.001 | -0.044 | -0.153 | 0.064 | 0.422 | 0.648 |
| HDL | 0.123 | 0.035 | 0.211 | 0.006 | 0.010 | 0.169 | 0.071 | 0.266 | 0.001 | 0.006 |
| Total | 0.099 | 0.011 | 0.186 | 0.027 | 0.037 | 0.153 | 0.056 | 0.251 | 0.002 | 0.013 |
| **Phospholipids** |  |  |  |  |  |  |  |  |  |  |
| VS-VLDL | -0.006 | -0.090 | 0.078 | 0.885 | 0.895 | -0.004 | -0.100 | 0.093 | 0.943 | 0.982 |
| S-VLDL | -0.040 | -0.123 | 0.044 | 0.349 | 0.392 | -0.044 | -0.141 | 0.052 | 0.370 | 0.591 |
| M-VLDL | -0.061 | -0.144 | 0.022 | 0.151 | 0.182 | -0.053 | -0.150 | 0.044 | 0.286 | 0.518 |
| L-VLDL | 0.153 | 0.067 | 0.239 | 0.001 | 0.001 | 0.040 | -0.061 | 0.140 | 0.439 | 0.666 |
| VL-VLDL | 0.169 | 0.083 | 0.255 | <0.001 | <0.001 | 0.062 | -0.039 | 0.162 | 0.230 | 0.444 |
| XL-VLDL | 0.245 | 0.160 | 0.331 | <0.001 | <0.001 | 0.120 | 0.021 | 0.219 | 0.017 | 0.057 |
| VLDL | 0.071 | -0.013 | 0.156 | 0.096 | 0.123 | 0.014 | -0.084 | 0.113 | 0.772 | 0.903 |
| S-LDL | -0.111 | -0.194 | -0.028 | 0.009 | 0.014 | -0.051 | -0.149 | 0.046 | 0.299 | 0.525 |
| M-LDL | -0.033 | -0.116 | 0.050 | 0.432 | 0.472 | 0.004 | -0.092 | 0.101 | 0.931 | 0.975 |
| L-LDL | -0.100 | -0.183 | -0.017 | 0.018 | 0.026 | -0.007 | -0.104 | 0.091 | 0.896 | 0.970 |
| LDL | -0.083 | -0.166 | <0.001 | 0.049 | 0.066 | -0.009 | -0.106 | 0.088 | 0.851 | 0.954 |
| IDL | -0.144 | -0.229 | -0.059 | 0.001 | 0.002 | -0.025 | -0.126 | 0.076 | 0.627 | 0.819 |
| S-HDL | 0.336 | 0.253 | 0.419 | <0.001 | <0.001 | 0.235 | 0.142 | 0.328 | <0.001 | <0.001 |
| M-HDL | 0.243 | 0.154 | 0.332 | <0.001 | <0.001 | 0.233 | 0.135 | 0.332 | <0.001 | <0.001 |
| L-HDL | -0.089 | -0.184 | 0.005 | 0.064 | 0.085 | 0.074 | -0.036 | 0.184 | 0.187 | 0.375 |
| XL-HDL | -0.235 | -0.328 | -0.142 | <0.001 | <0.001 | -0.038 | -0.147 | 0.071 | 0.491 | 0.699 |
| HDL | 0.127 | 0.035 | 0.219 | 0.007 | 0.011 | 0.191 | 0.087 | 0.294 | <0.001 | 0.004 |
| Total | 0.058 | -0.029 | 0.144 | 0.191 | 0.227 | 0.117 | 0.019 | 0.215 | 0.019 | 0.060 |
| **Phospholipids to Total lipids** | |  |  |  |  |  |  |  |  |  |
| VS-VLDL | 0.064 | -0.020 | 0.148 | 0.138 | 0.168 | -0.109 | -0.203 | -0.014 | 0.024 | 0.072 |
| S-VLDL | -0.307 | -0.392 | -0.222 | <0.001 | <0.001 | -0.119 | -0.219 | -0.019 | 0.019 | 0.060 |
| M-VLDL | -0.256 | -0.341 | -0.171 | <0.001 | <0.001 | -0.072 | -0.174 | 0.030 | 0.166 | 0.339 |
| L-VLDL | 0.144 | 0.058 | 0.229 | 0.001 | 0.002 | 0.016 | -0.080 | 0.112 | 0.748 | 0.892 |
| VL-VLDL | -0.036 | -0.120 | 0.047 | 0.392 | 0.433 | -0.055 | -0.147 | 0.037 | 0.245 | 0.459 |
| XL-VLDL | 0.118 | 0.035 | 0.202 | 0.006 | 0.009 | 0.021 | -0.071 | 0.113 | 0.658 | 0.827 |
| S-LDL | -0.143 | -0.227 | -0.060 | 0.001 | 0.002 | -0.100 | -0.194 | -0.007 | 0.035 | 0.096 |
| M-LDL | -0.069 | -0.152 | 0.014 | 0.103 | 0.131 | -0.055 | -0.146 | 0.036 | 0.238 | 0.453 |
| L-LDL | -0.016 | -0.099 | 0.068 | 0.714 | 0.741 | -0.002 | -0.093 | 0.089 | 0.964 | 0.991 |
| IDL | -0.119 | -0.202 | -0.037 | 0.005 | 0.008 | -0.165 | -0.256 | -0.075 | <0.001 | 0.005 |
| S-HDL | 0.242 | 0.156 | 0.329 | <0.001 | <0.001 | 0.167 | 0.072 | 0.262 | 0.001 | 0.006 |
| M-HDL | 0.298 | 0.211 | 0.384 | <0.001 | <0.001 | 0.104 | 0.002 | 0.206 | 0.046 | 0.118 |
| L-HDL | 0.536 | 0.450 | 0.621 | <0.001 | <0.001 | 0.309 | 0.202 | 0.416 | <0.001 | <0.001 |
| XL-HDL | -0.207 | -0.297 | -0.117 | <0.001 | <0.001 | -0.070 | -0.174 | 0.034 | 0.186 | 0.375 |
| **Total lipids** |  |  |  |  |  |  |  |  |  |  |
| VS-VLDL | -0.016 | -0.100 | 0.068 | 0.714 | 0.741 | 0.008 | -0.089 | 0.104 | 0.879 | 0.970 |
| S-VLDL | 0.039 | -0.045 | 0.124 | 0.359 | 0.399 | -0.012 | -0.108 | 0.085 | 0.814 | 0.943 |
| M-VLDL | -0.010 | -0.094 | 0.074 | 0.819 | 0.835 | -0.035 | -0.132 | 0.062 | 0.481 | 0.689 |
| L-VLDL | 0.135 | 0.049 | 0.221 | 0.002 | 0.004 | 0.031 | -0.069 | 0.131 | 0.544 | 0.753 |
| VL-VLDL | 0.192 | 0.106 | 0.278 | <0.001 | <0.001 | 0.077 | -0.024 | 0.177 | 0.134 | 0.290 |
| XL-VLDL | 0.236 | 0.151 | 0.322 | <0.001 | <0.001 | 0.122 | 0.023 | 0.220 | 0.016 | 0.056 |
| VLDL | 0.112 | 0.027 | 0.196 | 0.010 | 0.015 | 0.036 | -0.063 | 0.134 | 0.475 | 0.689 |
| S-LDL | -0.071 | -0.154 | 0.012 | 0.094 | 0.120 | -0.023 | -0.121 | 0.074 | 0.637 | 0.819 |
| M-LDL | -0.025 | -0.108 | 0.058 | 0.556 | 0.586 | 0.010 | -0.086 | 0.107 | 0.834 | 0.952 |
| L-LDL | -0.097 | -0.181 | -0.014 | 0.022 | 0.031 | -0.009 | -0.106 | 0.088 | 0.861 | 0.961 |
| LDL | -0.075 | -0.158 | 0.007 | 0.074 | 0.097 | -0.005 | -0.102 | 0.092 | 0.918 | 0.973 |
| IDL | -0.116 | -0.201 | -0.031 | 0.007 | 0.012 | 0.008 | -0.093 | 0.108 | 0.884 | 0.970 |
| S-HDL | 0.305 | 0.222 | 0.388 | <0.001 | <0.001 | 0.214 | 0.122 | 0.307 | <0.001 | <0.001 |
| M-HDL | 0.196 | 0.106 | 0.285 | <0.001 | <0.001 | 0.214 | 0.114 | 0.314 | <0.001 | 0.001 |
| L-HDL | -0.141 | -0.236 | -0.046 | 0.003 | 0.006 | 0.041 | -0.069 | 0.152 | 0.463 | 0.688 |
| XL-HDL | -0.250 | -0.343 | -0.157 | <0.001 | <0.001 | -0.044 | -0.154 | 0.065 | 0.425 | 0.650 |
| HDL | 0.066 | -0.027 | 0.158 | 0.164 | 0.196 | 0.163 | 0.058 | 0.268 | 0.002 | 0.014 |
| Total | 0.030 | -0.053 | 0.114 | 0.476 | 0.513 | 0.071 | -0.025 | 0.168 | 0.145 | 0.308 |
| **Triglycerides** |  |  |  |  |  |  |  |  |  |  |
| VS-VLDL | 0.213 | 0.129 | 0.296 | <0.001 | <0.001 | 0.096 | 0.002 | 0.191 | 0.046 | 0.118 |
| S-VLDL | 0.164 | 0.079 | 0.249 | <0.001 | <0.001 | 0.045 | -0.052 | 0.142 | 0.362 | 0.583 |
| M-VLDL | 0.121 | 0.035 | 0.206 | 0.006 | 0.009 | 0.023 | -0.075 | 0.121 | 0.645 | 0.819 |
| L-VLDL | 0.157 | 0.071 | 0.244 | <0.001 | 0.001 | 0.047 | -0.053 | 0.146 | 0.356 | 0.583 |
| XL-VLDL | 0.231 | 0.145 | 0.317 | <0.001 | <0.001 | 0.103 | 0.003 | 0.204 | 0.044 | 0.115 |
| XXL-VLDL | 0.239 | 0.154 | 0.325 | <0.001 | <0.001 | 0.128 | 0.029 | 0.226 | 0.011 | 0.044 |
| VLDL | 0.194 | 0.108 | 0.280 | <0.001 | <0.001 | 0.077 | -0.022 | 0.176 | 0.129 | 0.284 |
| S-LDL | 0.217 | 0.132 | 0.301 | <0.001 | <0.001 | 0.109 | 0.012 | 0.205 | 0.028 | 0.080 |
| M-LDL | 0.242 | 0.158 | 0.326 | <0.001 | <0.001 | 0.135 | 0.039 | 0.230 | 0.006 | 0.029 |
| L-LDL | 0.249 | 0.165 | 0.332 | <0.001 | <0.001 | 0.149 | 0.054 | 0.244 | 0.002 | 0.013 |
| LDL | 0.246 | 0.162 | 0.329 | <0.001 | <0.001 | 0.142 | 0.047 | 0.237 | 0.003 | 0.020 |
| IDL | 0.223 | 0.139 | 0.306 | <0.001 | <0.001 | 0.124 | 0.030 | 0.218 | 0.010 | 0.041 |
| S-HDL | 0.296 | 0.211 | 0.382 | <0.001 | <0.001 | 0.136 | 0.037 | 0.235 | 0.007 | 0.033 |
| M-HDL | 0.279 | 0.196 | 0.363 | <0.001 | <0.001 | 0.152 | 0.059 | 0.245 | 0.001 | 0.010 |
| L-HDL | 0.104 | 0.019 | 0.190 | 0.017 | 0.024 | 0.087 | -0.006 | 0.179 | 0.067 | 0.162 |
| XL-HDL | 0.077 | -0.006 | 0.161 | 0.069 | 0.090 | 0.054 | -0.038 | 0.145 | 0.251 | 0.467 |
| HDL | 0.247 | 0.164 | 0.330 | <0.001 | <0.001 | 0.131 | 0.038 | 0.224 | 0.006 | 0.029 |
| Total | 0.210 | 0.125 | 0.295 | <0.001 | <0.001 | 0.092 | -0.006 | 0.191 | 0.066 | 0.160 |
| **Triglycerides to Total lipids** | |  |  |  |  |  |  |  |  |  |
| VS-VLDL | 0.400 | 0.314 | 0.486 | <0.001 | <0.001 | 0.185 | 0.080 | 0.290 | 0.001 | 0.006 |
| S-VLDL | 0.293 | 0.209 | 0.377 | <0.001 | <0.001 | 0.123 | 0.024 | 0.221 | 0.014 | 0.054 |
| M-VLDL | 0.336 | 0.250 | 0.423 | <0.001 | <0.001 | 0.124 | 0.021 | 0.228 | 0.019 | 0.060 |
| L-VLDL | 0.051 | -0.031 | 0.134 | 0.223 | 0.261 | 0.045 | -0.046 | 0.136 | 0.334 | 0.561 |
| XL-VLDL | 0.246 | 0.162 | 0.331 | <0.001 | <0.001 | 0.105 | 0.009 | 0.202 | 0.032 | 0.089 |
| XXL-VLDL | 0.011 | -0.072 | 0.094 | 0.795 | 0.818 | -0.001 | -0.091 | 0.089 | 0.981 | 0.991 |
| S-LDL | 0.333 | 0.249 | 0.417 | <0.001 | <0.001 | 0.154 | 0.057 | 0.250 | 0.002 | 0.012 |
| M-LDL | 0.335 | 0.252 | 0.418 | <0.001 | <0.001 | 0.159 | 0.063 | 0.254 | 0.001 | 0.009 |
| L-LDL | 0.372 | 0.289 | 0.456 | <0.001 | <0.001 | 0.164 | 0.067 | 0.261 | 0.001 | 0.007 |
| IDL | 0.366 | 0.282 | 0.451 | <0.001 | <0.001 | 0.140 | 0.039 | 0.242 | 0.007 | 0.032 |
| S-HDL | 0.205 | 0.118 | 0.291 | <0.001 | <0.001 | 0.049 | -0.052 | 0.149 | 0.340 | 0.565 |
| M-HDL | 0.195 | 0.109 | 0.280 | <0.001 | <0.001 | 0.053 | -0.045 | 0.152 | 0.291 | 0.518 |
| L-HDL | 0.210 | 0.123 | 0.297 | <0.001 | <0.001 | 0.055 | -0.046 | 0.156 | 0.289 | 0.518 |
| XL-HDL | 0.317 | 0.231 | 0.404 | <0.001 | <0.001 | 0.133 | 0.028 | 0.238 | 0.013 | 0.050 |

† Model adjusted for age, sex, and education.
‡ Model adjusted for age, sex, education, race, socioeconomic status, body mass index, smoking status, alcohol drinking status, physical activity, social connection, hypertension, diabetes, heart disease, beta-blockers, calcium blockers, lipid-lowering, and *APOE* ε4.
Abbreviation: Apo, apolipoprotein; LDL-AD, average diameter for LDL particles; CHOL, cholesterol; CE, cholesteryl esters; TG, triglycerides; S, small; M, medium; L, large; VL, very large; XL, extremely large; VLDL, very low-density lipoprotein; LDL; low-density lipoprotein; IDL, intermediate-density lipoprotein; HDL, high-density lipoprotein; LA, linoleic acid; MUFA, monounsaturated fatty acid; n-6, omega-6 fatty acid; PUFA, polyunsaturated fatty acid; SFA, saturated fatty acid; FA, fatty acid.

**Table S8.** β coefficients and 95% confidence intervals (CIs) for the association between metabolites and brain age gap (BAG): results from linear regression models

| **Metabolites** | **Basic-model^†^** | |  |  |  | **Multi-model^‡^** | |  |  |  |
| --- | --- | --- | --- | --- | --- | --- | --- | --- | --- | --- |
|  | **β** | **Lower** | **Upper** | ***P*-value** | **FDR-q** | **β** | **Lower** | **Upper** | ***P*-value** | **FDR-q** |
| **Amino acids & Glycolysis** | |  |  |  |  |  |  |  |  |  |
| Valine | 0.141 | 0.056 | 0.226 | 0.001 | 0.002 | 0.070 | -0.026 | 0.165 | 0.153 | 0.321 |
| Leucine | 0.118 | 0.032 | 0.204 | 0.007 | 0.012 | 0.063 | -0.032 | 0.158 | 0.192 | 0.383 |
| Isoleucine | 0.071 | -0.013 | 0.155 | 0.099 | 0.126 | 0.022 | -0.070 | 0.115 | 0.638 | 0.819 |
| Phenylalanine | 0.113 | 0.031 | 0.196 | 0.007 | 0.012 | 0.074 | -0.017 | 0.164 | 0.111 | 0.251 |
| Tyrosine | 0.242 | 0.159 | 0.325 | <0.001 | <0.001 | 0.198 | 0.106 | 0.290 | <0.001 | 0.001 |
| Alanine | 0.085 | 0.001 | 0.168 | 0.046 | 0.062 | 0.021 | -0.070 | 0.112 | 0.650 | 0.822 |
| Glutamine | -0.298 | -0.381 | -0.215 | <0.001 | <0.001 | -0.222 | -0.314 | -0.130 | <0.001 | <0.001 |
| Glycine | -0.079 | -0.167 | 0.009 | 0.079 | 0.103 | -0.008 | -0.105 | 0.089 | 0.874 | 0.970 |
| Histidine | -0.073 | -0.157 | 0.010 | 0.084 | 0.108 | -0.037 | -0.127 | 0.053 | 0.418 | 0.648 |
| BCAAs | 0.124 | 0.039 | 0.210 | 0.004 | 0.007 | 0.060 | -0.035 | 0.155 | 0.216 | 0.419 |
| Lactate | 0.055 | -0.028 | 0.138 | 0.193 | 0.229 | -0.019 | -0.109 | 0.072 | 0.686 | 0.849 |
| Pyruvate | 0.003 | -0.080 | 0.086 | 0.944 | 0.948 | -0.044 | -0.134 | 0.046 | 0.340 | 0.565 |
| Glucose | 0.311 | 0.228 | 0.394 | <0.001 | <0.001 | 0.152 | 0.055 | 0.249 | 0.002 | 0.013 |
| Citrate | -0.129 | -0.213 | -0.044 | 0.003 | 0.005 | -0.067 | -0.160 | 0.025 | 0.153 | 0.321 |
| **Apo-LP, LP size & Other lipids** | |  |  |  |  |  |  |  |  |  |
| ApoA1 | 0.111 | 0.020 | 0.201 | 0.016 | 0.024 | 0.177 | 0.075 | 0.278 | 0.001 | 0.006 |
| ApoB | -0.109 | -0.192 | -0.026 | 0.010 | 0.016 | -0.050 | -0.148 | 0.048 | 0.318 | 0.542 |
| ApoB/ApoA1 | -0.145 | -0.230 | -0.060 | 0.001 | 0.002 | -0.135 | -0.235 | -0.035 | 0.008 | 0.036 |
| HDL-AD | -0.197 | -0.291 | -0.102 | <0.001 | <0.001 | 0.002 | -0.110 | 0.113 | 0.978 | 0.991 |
| LDL-AD | -0.205 | -0.291 | -0.120 | <0.001 | <0.001 | -0.141 | -0.237 | -0.046 | 0.004 | 0.020 |
| VLDL-AD | 0.207 | 0.117 | 0.297 | <0.001 | <0.001 | 0.047 | -0.057 | 0.152 | 0.375 | 0.595 |
| Phosphatidylcholine | 0.096 | 0.008 | 0.184 | 0.033 | 0.045 | 0.139 | 0.041 | 0.237 | 0.005 | 0.028 |
| Phosphoglycerides | 0.128 | 0.041 | 0.215 | 0.004 | 0.007 | 0.150 | 0.053 | 0.247 | 0.002 | 0.014 |
| Sphingomyeline | -0.071 | -0.159 | 0.016 | 0.111 | 0.139 | 0.048 | -0.053 | 0.149 | 0.350 | 0.578 |
| Total choline | 0.069 | -0.019 | 0.156 | 0.124 | 0.154 | 0.122 | 0.024 | 0.221 | 0.015 | 0.054 |
| TG/Phosphoglycerides | 0.206 | 0.116 | 0.295 | <0.001 | <0.001 | 0.039 | -0.066 | 0.145 | 0.465 | 0.688 |
| **Cholesterol** |  |  |  |  |  |  |  |  |  |  |
| XS-VLDL | -0.122 | -0.207 | -0.037 | 0.005 | 0.008 | -0.031 | -0.130 | 0.069 | 0.549 | 0.755 |
| S-VLDL | -0.065 | -0.148 | 0.018 | 0.127 | 0.156 | -0.057 | -0.154 | 0.039 | 0.244 | 0.459 |
| M-VLDL | -0.194 | -0.276 | -0.111 | <0.001 | <0.001 | -0.115 | -0.213 | -0.018 | 0.020 | 0.063 |
| L-VLDL | 0.067 | -0.019 | 0.152 | 0.125 | 0.154 | -0.010 | -0.109 | 0.089 | 0.846 | 0.954 |
| VL-VLDL | 0.089 | 0.003 | 0.174 | 0.042 | 0.057 | 0.010 | -0.090 | 0.110 | 0.846 | 0.954 |
| XL-VLDL | 0.208 | 0.123 | 0.294 | <0.001 | <0.001 | 0.097 | -0.002 | 0.196 | 0.055 | 0.138 |
| VLDL | -0.033 | -0.117 | 0.050 | 0.432 | 0.472 | -0.034 | -0.131 | 0.064 | 0.499 | 0.702 |
| S-LDL | -0.084 | -0.167 | -0.001 | 0.047 | 0.063 | -0.027 | -0.124 | 0.071 | 0.591 | 0.787 |
| M-LDL | -0.044 | -0.127 | 0.039 | 0.302 | 0.351 | 0.001 | -0.095 | 0.098 | 0.981 | 0.991 |
| L-LDL | -0.123 | -0.206 | -0.040 | 0.004 | 0.006 | -0.024 | -0.121 | 0.073 | 0.629 | 0.819 |
| Clinical LDL | -0.126 | -0.209 | -0.043 | 0.003 | 0.005 | -0.029 | -0.127 | 0.068 | 0.554 | 0.758 |
| LDL | -0.099 | -0.182 | -0.016 | 0.019 | 0.027 | -0.018 | -0.114 | 0.079 | 0.720 | 0.871 |
| IDL | -0.142 | -0.227 | -0.057 | 0.001 | 0.002 | <0.001 | -0.101 | 0.101 | 0.999 | 0.999 |
| S-HDL | 0.199 | 0.116 | 0.282 | <0.001 | <0.001 | 0.160 | 0.069 | 0.252 | 0.001 | 0.006 |
| M-HDL | 0.109 | 0.019 | 0.199 | 0.018 | 0.026 | 0.178 | 0.076 | 0.280 | 0.001 | 0.006 |
| L-HDL | -0.198 | -0.293 | -0.104 | <0.001 | <0.001 | 0.003 | -0.109 | 0.114 | 0.961 | 0.991 |
| XL-HDL | -0.278 | -0.370 | -0.186 | <0.001 | <0.001 | -0.059 | -0.168 | 0.051 | 0.293 | 0.518 |
| HDL | -0.040 | -0.133 | 0.052 | 0.393 | 0.433 | 0.110 | 0.003 | 0.217 | 0.044 | 0.115 |
| Total | -0.102 | -0.187 | -0.017 | 0.018 | 0.026 | 0.015 | -0.084 | 0.114 | 0.761 | 0.899 |
| Remnant | -0.089 | -0.172 | -0.006 | 0.036 | 0.049 | -0.020 | -0.119 | 0.078 | 0.685 | 0.849 |
| Total-HDLC | -0.096 | -0.179 | -0.013 | 0.023 | 0.033 | -0.019 | -0.117 | 0.078 | 0.697 | 0.855 |
| **Cholesterol to Total lipids** | |  |  |  |  |  |  |  |  |  |
| VS-VLDL | -0.370 | -0.457 | -0.284 | <0.001 | <0.001 | -0.139 | -0.244 | -0.033 | 0.010 | 0.041 |
| S-VLDL | -0.279 | -0.363 | -0.195 | <0.001 | <0.001 | -0.120 | -0.218 | -0.023 | 0.016 | 0.056 |
| M-VLDL | -0.351 | -0.438 | -0.264 | <0.001 | <0.001 | -0.136 | -0.240 | -0.032 | 0.011 | 0.043 |
| L-VLDL | -0.181 | -0.265 | -0.097 | <0.001 | <0.001 | -0.078 | -0.173 | 0.016 | 0.103 | 0.236 |
| XL-VLDL | -0.244 | -0.329 | -0.158 | <0.001 | <0.001 | -0.088 | -0.186 | 0.010 | 0.079 | 0.185 |
| XXL-VLDL | -0.051 | -0.134 | 0.032 | 0.227 | 0.266 | -0.006 | -0.096 | 0.085 | 0.904 | 0.970 |
| S-LDL | -0.154 | -0.237 | -0.072 | <0.001 | 0.001 | -0.034 | -0.127 | 0.059 | 0.477 | 0.689 |
| M-LDL | -0.276 | -0.359 | -0.193 | <0.001 | <0.001 | -0.116 | -0.211 | -0.021 | 0.017 | 0.057 |
| L-LDL | -0.361 | -0.444 | -0.277 | <0.001 | <0.001 | -0.160 | -0.257 | -0.063 | 0.001 | 0.009 |
| IDL | -0.258 | -0.342 | -0.174 | <0.001 | <0.001 | -0.046 | -0.145 | 0.053 | 0.361 | 0.583 |
| S-HDL | -0.283 | -0.365 | -0.200 | <0.001 | <0.001 | -0.137 | -0.229 | -0.044 | 0.004 | 0.021 |
| M-HDL | -0.248 | -0.334 | -0.161 | <0.001 | <0.001 | -0.077 | -0.178 | 0.024 | 0.133 | 0.290 |
| L-HDL | -0.426 | -0.513 | -0.338 | <0.001 | <0.001 | -0.200 | -0.306 | -0.094 | <0.001 | 0.004 |
| XL-HDL | 0.042 | -0.046 | 0.130 | 0.355 | 0.396 | 0.005 | -0.094 | 0.103 | 0.927 | 0.974 |
| **Cholesteryl esters to Total lipids** | |  |  |  |  |  |  |  |  |  |
| VS-VLDL | -0.361 | -0.448 | -0.274 | <0.001 | <0.001 | -0.121 | -0.227 | -0.015 | 0.025 | 0.075 |
| S-VLDL | -0.222 | -0.305 | -0.139 | <0.001 | <0.001 | -0.105 | -0.200 | -0.011 | 0.029 | 0.082 |
| M-VLDL | -0.357 | -0.445 | -0.270 | <0.001 | <0.001 | -0.141 | -0.246 | -0.037 | 0.008 | 0.035 |
| L-VLDL | -0.267 | -0.353 | -0.181 | <0.001 | <0.001 | -0.119 | -0.218 | -0.020 | 0.018 | 0.060 |
| XL-VLDL | -0.235 | -0.321 | -0.149 | <0.001 | <0.001 | -0.085 | -0.182 | 0.012 | 0.088 | 0.204 |
| XXL-VLDL | -0.065 | -0.148 | 0.017 | 0.121 | 0.152 | -0.021 | -0.111 | 0.069 | 0.645 | 0.819 |
| S-LDL | 0.132 | 0.048 | 0.215 | 0.002 | 0.004 | 0.085 | -0.009 | 0.179 | 0.078 | 0.184 |
| M-LDL | 0.094 | 0.009 | 0.178 | 0.030 | 0.041 | 0.034 | -0.061 | 0.130 | 0.481 | 0.689 |
| L-LDL | -0.155 | -0.238 | -0.073 | <0.001 | 0.001 | -0.103 | -0.193 | -0.013 | 0.025 | 0.075 |
| IDL | -0.170 | -0.254 | -0.086 | <0.001 | <0.001 | <0.001 | -0.097 | 0.097 | 0.995 | 0.999 |
| S-HDL | -0.225 | -0.308 | -0.142 | <0.001 | <0.001 | -0.121 | -0.212 | -0.029 | 0.010 | 0.041 |
| M-HDL | -0.241 | -0.326 | -0.157 | <0.001 | <0.001 | -0.099 | -0.196 | -0.001 | 0.048 | 0.120 |
| L-HDL | -0.424 | -0.510 | -0.338 | <0.001 | <0.001 | -0.214 | -0.318 | -0.110 | <0.001 | 0.001 |
| XL-HDL | -0.127 | -0.210 | -0.044 | 0.003 | 0.005 | -0.017 | -0.109 | 0.075 | 0.718 | 0.871 |
| **Cholestryl esters** |  |  |  |  |  |  |  |  |  |  |
| VS-VLDL | -0.146 | -0.231 | -0.061 | 0.001 | 0.002 | -0.035 | -0.136 | 0.066 | 0.499 | 0.702 |
| S-VLDL | -0.042 | -0.126 | 0.041 | 0.320 | 0.365 | -0.050 | -0.146 | 0.047 | 0.316 | 0.542 |
| M-VLDL | -0.251 | -0.334 | -0.168 | <0.001 | <0.001 | -0.141 | -0.239 | -0.043 | 0.005 | 0.026 |
| L-VLDL | <0.001 | -0.085 | 0.084 | 0.993 | 0.993 | -0.049 | -0.147 | 0.049 | 0.325 | 0.551 |
| VL-VLDL | 0.033 | -0.052 | 0.118 | 0.447 | 0.486 | -0.025 | -0.125 | 0.074 | 0.616 | 0.816 |
| XL-VLDL | 0.188 | 0.102 | 0.273 | <0.001 | <0.001 | 0.083 | -0.016 | 0.182 | 0.100 | 0.230 |
| VLDL | -0.078 | -0.161 | 0.005 | 0.064 | 0.085 | -0.054 | -0.152 | 0.043 | 0.276 | 0.505 |
| S-LDL | -0.043 | -0.126 | 0.040 | 0.308 | 0.356 | -0.007 | -0.104 | 0.091 | 0.891 | 0.970 |
| M-LDL | -0.012 | -0.095 | 0.072 | 0.782 | 0.808 | 0.015 | -0.082 | 0.112 | 0.766 | 0.899 |
| L-LDL | -0.108 | -0.191 | -0.025 | 0.011 | 0.017 | -0.021 | -0.117 | 0.076 | 0.672 | 0.840 |
| LDL | -0.077 | -0.160 | 0.006 | 0.069 | 0.090 | -0.010 | -0.106 | 0.086 | 0.837 | 0.952 |
| IDL | -0.131 | -0.216 | -0.046 | 0.003 | 0.005 | 0.006 | -0.095 | 0.108 | 0.902 | 0.970 |
| S-HDL | 0.185 | 0.102 | 0.268 | <0.001 | <0.001 | 0.146 | 0.054 | 0.237 | 0.002 | 0.012 |
| M-HDL | 0.106 | 0.016 | 0.197 | 0.020 | 0.029 | 0.174 | 0.073 | 0.276 | 0.001 | 0.007 |
| L-HDL | -0.208 | -0.302 | -0.114 | <0.001 | <0.001 | -0.006 | -0.117 | 0.106 | 0.922 | 0.973 |
| XL-HDL | -0.277 | -0.370 | -0.184 | <0.001 | <0.001 | -0.057 | -0.167 | 0.053 | 0.312 | 0.539 |
| HDL | -0.048 | -0.141 | 0.044 | 0.309 | 0.356 | 0.103 | -0.004 | 0.209 | 0.060 | 0.148 |
| Total | -0.099 | -0.184 | -0.014 | 0.022 | 0.032 | 0.024 | -0.075 | 0.123 | 0.636 | 0.819 |
| **Fatty acids** |  |  |  |  |  |  |  |  |  |  |
| DHA | -0.044 | -0.130 | 0.042 | 0.313 | 0.359 | 0.037 | -0.057 | 0.131 | 0.442 | 0.667 |
| LA | -0.102 | -0.186 | -0.019 | 0.016 | 0.024 | -0.039 | -0.134 | 0.056 | 0.419 | 0.648 |
| MUFA | 0.249 | 0.166 | 0.332 | <0.001 | <0.001 | 0.130 | 0.035 | 0.226 | 0.008 | 0.035 |
| n-3 | 0.042 | -0.043 | 0.128 | 0.329 | 0.373 | 0.056 | -0.037 | 0.148 | 0.236 | 0.453 |
| n-6 | -0.041 | -0.125 | 0.043 | 0.337 | 0.380 | -0.001 | -0.096 | 0.094 | 0.983 | 0.991 |
| PUFA | -0.024 | -0.108 | 0.061 | 0.584 | 0.614 | 0.015 | -0.079 | 0.110 | 0.749 | 0.892 |
| SFA | 0.238 | 0.155 | 0.321 | <0.001 | <0.001 | 0.176 | 0.081 | 0.270 | <0.001 | 0.004 |
| Total | 0.171 | 0.087 | 0.254 | <0.001 | <0.001 | 0.117 | 0.023 | 0.211 | 0.015 | 0.056 |
| Unsaturation | -0.276 | -0.362 | -0.190 | <0.001 | <0.001 | -0.119 | -0.216 | -0.021 | 0.017 | 0.057 |
| DHA/FA | -0.124 | -0.209 | -0.039 | 0.004 | 0.007 | -0.005 | -0.100 | 0.090 | 0.913 | 0.973 |
| LA/FA | -0.467 | -0.551 | -0.382 | <0.001 | <0.001 | -0.291 | -0.392 | -0.190 | <0.001 | <0.001 |
| MUFA/FA | 0.329 | 0.242 | 0.415 | <0.001 | <0.001 | 0.108 | 0.005 | 0.210 | 0.040 | 0.106 |
| n-3/FA | -0.026 | -0.111 | 0.059 | 0.546 | 0.579 | 0.026 | -0.067 | 0.118 | 0.586 | 0.787 |
| n-6/n-3 | -0.026 | -0.110 | 0.058 | 0.545 | 0.579 | -0.001 | -0.094 | 0.091 | 0.978 | 0.991 |
| n-6/FA | -0.423 | -0.508 | -0.338 | <0.001 | <0.001 | -0.267 | -0.366 | -0.167 | <0.001 | <0.001 |
| PUFA/MUFA | -0.354 | -0.440 | -0.268 | <0.001 | <0.001 | -0.147 | -0.249 | -0.045 | 0.005 | 0.026 |
| PUFA/FA | -0.422 | -0.507 | -0.337 | <0.001 | <0.001 | -0.251 | -0.352 | -0.151 | <0.001 | <0.001 |
| SFA/FA | 0.348 | 0.265 | 0.430 | <0.001 | <0.001 | 0.284 | 0.191 | 0.377 | <0.001 | <0.001 |
| **Free cholesterol** |  |  |  |  |  |  |  |  |  |  |
| VS-VLDL | -0.062 | -0.146 | 0.022 | 0.151 | 0.182 | -0.020 | -0.117 | 0.078 | 0.688 | 0.849 |
| S-VLDL | -0.103 | -0.186 | -0.020 | 0.015 | 0.023 | -0.070 | -0.167 | 0.027 | 0.155 | 0.321 |
| M-VLDL | -0.109 | -0.192 | -0.026 | 0.010 | 0.015 | -0.076 | -0.173 | 0.021 | 0.124 | 0.278 |
| L-VLDL | 0.131 | 0.045 | 0.217 | 0.003 | 0.005 | 0.029 | -0.071 | 0.130 | 0.567 | 0.772 |
| VL-VLDL | 0.144 | 0.059 | 0.230 | 0.001 | 0.002 | 0.046 | -0.054 | 0.146 | 0.363 | 0.583 |
| XL-VLDL | 0.230 | 0.145 | 0.316 | <0.001 | <0.001 | 0.111 | 0.013 | 0.209 | 0.027 | 0.079 |
| VLDL | 0.029 | -0.055 | 0.113 | 0.503 | 0.537 | -0.005 | -0.103 | 0.093 | 0.919 | 0.973 |
| S-LDL | -0.182 | -0.265 | -0.100 | <0.001 | <0.001 | -0.075 | -0.171 | 0.021 | 0.128 | 0.284 |
| M-LDL | -0.127 | -0.210 | -0.044 | 0.003 | 0.005 | -0.035 | -0.131 | 0.061 | 0.472 | 0.689 |
| L-LDL | -0.163 | -0.246 | -0.079 | <0.001 | <0.001 | -0.032 | -0.130 | 0.066 | 0.525 | 0.730 |
| LDL | -0.156 | -0.239 | -0.073 | <0.001 | <0.001 | -0.038 | -0.135 | 0.060 | 0.448 | 0.671 |
| IDL | -0.168 | -0.253 | -0.083 | <0.001 | <0.001 | -0.018 | -0.119 | 0.084 | 0.732 | 0.881 |
| S-HDL | 0.214 | 0.130 | 0.298 | <0.001 | <0.001 | 0.182 | 0.089 | 0.275 | <0.001 | 0.002 |
| M-HDL | 0.115 | 0.024 | 0.207 | 0.013 | 0.020 | 0.185 | 0.083 | 0.287 | <0.001 | 0.005 |
| L-HDL | -0.163 | -0.257 | -0.068 | 0.001 | 0.002 | 0.031 | -0.079 | 0.142 | 0.578 | 0.782 |
| XL-HDL | -0.266 | -0.356 | -0.176 | <0.001 | <0.001 | -0.062 | -0.167 | 0.044 | 0.253 | 0.467 |
| HDL | -0.011 | -0.105 | 0.082 | 0.811 | 0.831 | 0.130 | 0.024 | 0.237 | 0.017 | 0.057 |
| Total | -0.107 | -0.191 | -0.023 | 0.013 | 0.019 | -0.006 | -0.105 | 0.092 | 0.899 | 0.970 |
| **Free cholesterol to Total lipids** | |  |  |  |  |  |  |  |  |  |
| VS-VLDL | -0.339 | -0.421 | -0.256 | <0.001 | <0.001 | -0.194 | -0.290 | -0.099 | <0.001 | 0.001 |
| S-VLDL | -0.316 | -0.401 | -0.230 | <0.001 | <0.001 | -0.121 | -0.222 | -0.020 | 0.019 | 0.060 |
| M-VLDL | -0.318 | -0.404 | -0.232 | <0.001 | <0.001 | -0.112 | -0.215 | -0.009 | 0.032 | 0.090 |
| L-VLDL | 0.065 | -0.019 | 0.148 | 0.128 | 0.157 | 0.023 | -0.070 | 0.115 | 0.632 | 0.819 |
| XL-VLDL | -0.205 | -0.289 | -0.120 | <0.001 | <0.001 | -0.069 | -0.165 | 0.027 | 0.158 | 0.324 |
| XXL-VLDL | -0.006 | -0.089 | 0.077 | 0.888 | 0.895 | 0.031 | -0.062 | 0.123 | 0.515 | 0.720 |
| S-LDL | -0.303 | -0.387 | -0.219 | <0.001 | <0.001 | -0.130 | -0.225 | -0.035 | 0.008 | 0.035 |
| M-LDL | -0.335 | -0.421 | -0.250 | <0.001 | <0.001 | -0.140 | -0.240 | -0.041 | 0.006 | 0.029 |
| L-LDL | -0.345 | -0.431 | -0.259 | <0.001 | <0.001 | -0.108 | -0.210 | -0.006 | 0.039 | 0.104 |
| IDL | -0.290 | -0.373 | -0.207 | <0.001 | <0.001 | -0.105 | -0.199 | -0.011 | 0.028 | 0.080 |
| S-HDL | -0.230 | -0.317 | -0.143 | <0.001 | <0.001 | -0.051 | -0.151 | 0.048 | 0.309 | 0.538 |
| M-HDL | -0.143 | -0.236 | -0.050 | 0.003 | 0.005 | 0.071 | -0.038 | 0.179 | 0.201 | 0.395 |
| L-HDL | -0.226 | -0.315 | -0.138 | <0.001 | <0.001 | -0.020 | -0.126 | 0.085 | 0.703 | 0.859 |
| XL-HDL | 0.212 | 0.119 | 0.305 | <0.001 | <0.001 | 0.030 | -0.079 | 0.140 | 0.589 | 0.787 |
| **KB, FB & Inflammation** | |  |  |  |  |  |  |  |  |  |
| Glycoprotein acetyls | 0.309 | 0.225 | 0.392 | <0.001 | <0.001 | 0.169 | 0.072 | 0.266 | 0.001 | 0.006 |
| 3-Hydrocybutyrate | 0.108 | 0.025 | 0.191 | 0.011 | 0.017 | 0.118 | 0.028 | 0.208 | 0.010 | 0.042 |
| Acetate | -0.126 | -0.209 | -0.043 | 0.003 | 0.005 | -0.090 | -0.176 | -0.003 | 0.042 | 0.111 |
| Acetoacetate | 0.159 | 0.076 | 0.242 | <0.001 | <0.001 | 0.159 | 0.068 | 0.249 | 0.001 | 0.006 |
| Acetone | 0.109 | 0.026 | 0.192 | 0.010 | 0.015 | 0.143 | 0.053 | 0.234 | 0.002 | 0.012 |
| Albumin | 0.008 | -0.075 | 0.090 | 0.859 | 0.873 | 0.033 | -0.058 | 0.125 | 0.478 | 0.689 |
| Creatinine | -0.038 | -0.139 | 0.062 | 0.455 | 0.492 | -0.134 | -0.245 | -0.024 | 0.017 | 0.058 |
| **Lipoprotein particles** | |  |  |  |  |  |  |  |  |  |
| VS-VLDL | -0.030 | -0.113 | 0.054 | 0.486 | 0.521 | 0.001 | -0.096 | 0.098 | 0.979 | 0.991 |
| S-VLDL | 0.042 | -0.042 | 0.127 | 0.326 | 0.370 | -0.015 | -0.112 | 0.081 | 0.754 | 0.894 |
| M-VLDL | -0.054 | -0.137 | 0.030 | 0.206 | 0.243 | -0.052 | -0.149 | 0.045 | 0.292 | 0.518 |
| L-VLDL | 0.147 | 0.060 | 0.233 | 0.001 | 0.002 | 0.042 | -0.059 | 0.142 | 0.416 | 0.648 |
| VL-VLDL | 0.194 | 0.107 | 0.280 | <0.001 | <0.001 | 0.075 | -0.025 | 0.176 | 0.142 | 0.306 |
| XL-VLDL | 0.244 | 0.159 | 0.330 | <0.001 | <0.001 | 0.123 | 0.024 | 0.222 | 0.015 | 0.054 |
| VLDL | 0.023 | -0.061 | 0.106 | 0.598 | 0.625 | -0.006 | -0.103 | 0.091 | 0.900 | 0.970 |
| S-LDL | -0.095 | -0.179 | -0.012 | 0.024 | 0.034 | -0.040 | -0.138 | 0.057 | 0.418 | 0.648 |
| M-LDL | -0.060 | -0.143 | 0.023 | 0.155 | 0.187 | -0.011 | -0.108 | 0.087 | 0.830 | 0.952 |
| L-LDL | -0.153 | -0.236 | -0.070 | <0.001 | 0.001 | -0.089 | -0.186 | 0.008 | 0.073 | 0.175 |
| LDL | -0.125 | -0.207 | -0.042 | 0.003 | 0.006 | -0.064 | -0.161 | 0.033 | 0.197 | 0.390 |
| IDL | -0.108 | -0.191 | -0.025 | 0.011 | 0.017 | -0.011 | -0.111 | 0.088 | 0.822 | 0.948 |
| S-HDL | 0.212 | 0.129 | 0.295 | <0.001 | <0.001 | 0.159 | 0.067 | 0.251 | 0.001 | 0.006 |
| M-HDL | 0.143 | 0.053 | 0.233 | 0.002 | 0.004 | 0.190 | 0.090 | 0.291 | <0.001 | 0.004 |
| L-HDL | -0.177 | -0.272 | -0.083 | <0.001 | 0.001 | 0.016 | -0.095 | 0.127 | 0.778 | 0.905 |
| XL-HDL | -0.253 | -0.345 | -0.160 | <0.001 | <0.001 | -0.044 | -0.153 | 0.064 | 0.422 | 0.648 |
| HDL | 0.123 | 0.035 | 0.211 | 0.006 | 0.010 | 0.169 | 0.071 | 0.266 | 0.001 | 0.006 |
| Total | 0.099 | 0.011 | 0.186 | 0.027 | 0.037 | 0.153 | 0.056 | 0.251 | 0.002 | 0.013 |
| **Phospholipids** |  |  |  |  |  |  |  |  |  |  |
| VS-VLDL | -0.006 | -0.090 | 0.078 | 0.885 | 0.895 | -0.004 | -0.100 | 0.093 | 0.943 | 0.982 |
| S-VLDL | -0.040 | -0.123 | 0.044 | 0.349 | 0.392 | -0.044 | -0.141 | 0.052 | 0.370 | 0.591 |
| M-VLDL | -0.061 | -0.144 | 0.022 | 0.151 | 0.182 | -0.053 | -0.150 | 0.044 | 0.286 | 0.518 |
| L-VLDL | 0.153 | 0.067 | 0.239 | 0.001 | 0.001 | 0.040 | -0.061 | 0.140 | 0.439 | 0.666 |
| VL-VLDL | 0.169 | 0.083 | 0.255 | <0.001 | <0.001 | 0.062 | -0.039 | 0.162 | 0.230 | 0.444 |
| XL-VLDL | 0.245 | 0.160 | 0.331 | <0.001 | <0.001 | 0.120 | 0.021 | 0.219 | 0.017 | 0.057 |
| VLDL | 0.071 | -0.013 | 0.156 | 0.096 | 0.123 | 0.014 | -0.084 | 0.113 | 0.772 | 0.903 |
| S-LDL | -0.111 | -0.194 | -0.028 | 0.009 | 0.014 | -0.051 | -0.149 | 0.046 | 0.299 | 0.525 |
| M-LDL | -0.033 | -0.116 | 0.050 | 0.432 | 0.472 | 0.004 | -0.092 | 0.101 | 0.931 | 0.975 |
| L-LDL | -0.100 | -0.183 | -0.017 | 0.018 | 0.026 | -0.007 | -0.104 | 0.091 | 0.896 | 0.970 |
| LDL | -0.083 | -0.166 | <0.001 | 0.049 | 0.066 | -0.009 | -0.106 | 0.088 | 0.851 | 0.954 |
| IDL | -0.144 | -0.229 | -0.059 | 0.001 | 0.002 | -0.025 | -0.126 | 0.076 | 0.627 | 0.819 |
| S-HDL | 0.336 | 0.253 | 0.419 | <0.001 | <0.001 | 0.235 | 0.142 | 0.328 | <0.001 | <0.001 |
| M-HDL | 0.243 | 0.154 | 0.332 | <0.001 | <0.001 | 0.233 | 0.135 | 0.332 | <0.001 | <0.001 |
| L-HDL | -0.089 | -0.184 | 0.005 | 0.064 | 0.085 | 0.074 | -0.036 | 0.184 | 0.187 | 0.375 |
| XL-HDL | -0.235 | -0.328 | -0.142 | <0.001 | <0.001 | -0.038 | -0.147 | 0.071 | 0.491 | 0.699 |
| HDL | 0.127 | 0.035 | 0.219 | 0.007 | 0.011 | 0.191 | 0.087 | 0.294 | <0.001 | 0.004 |
| Total | 0.058 | -0.029 | 0.144 | 0.191 | 0.227 | 0.117 | 0.019 | 0.215 | 0.019 | 0.060 |
| **Phospholipids to Total lipids** | |  |  |  |  |  |  |  |  |  |
| VS-VLDL | 0.064 | -0.020 | 0.148 | 0.138 | 0.168 | -0.109 | -0.203 | -0.014 | 0.024 | 0.072 |
| S-VLDL | -0.307 | -0.392 | -0.222 | <0.001 | <0.001 | -0.119 | -0.219 | -0.019 | 0.019 | 0.060 |
| M-VLDL | -0.256 | -0.341 | -0.171 | <0.001 | <0.001 | -0.072 | -0.174 | 0.030 | 0.166 | 0.339 |
| L-VLDL | 0.144 | 0.058 | 0.229 | 0.001 | 0.002 | 0.016 | -0.080 | 0.112 | 0.748 | 0.892 |
| VL-VLDL | -0.036 | -0.120 | 0.047 | 0.392 | 0.433 | -0.055 | -0.147 | 0.037 | 0.245 | 0.459 |
| XL-VLDL | 0.118 | 0.035 | 0.202 | 0.006 | 0.009 | 0.021 | -0.071 | 0.113 | 0.658 | 0.827 |
| S-LDL | -0.143 | -0.227 | -0.060 | 0.001 | 0.002 | -0.100 | -0.194 | -0.007 | 0.035 | 0.096 |
| M-LDL | -0.069 | -0.152 | 0.014 | 0.103 | 0.131 | -0.055 | -0.146 | 0.036 | 0.238 | 0.453 |
| L-LDL | -0.016 | -0.099 | 0.068 | 0.714 | 0.741 | -0.002 | -0.093 | 0.089 | 0.964 | 0.991 |
| IDL | -0.119 | -0.202 | -0.037 | 0.005 | 0.008 | -0.165 | -0.256 | -0.075 | <0.001 | 0.005 |
| S-HDL | 0.242 | 0.156 | 0.329 | <0.001 | <0.001 | 0.167 | 0.072 | 0.262 | 0.001 | 0.006 |
| M-HDL | 0.298 | 0.211 | 0.384 | <0.001 | <0.001 | 0.104 | 0.002 | 0.206 | 0.046 | 0.118 |
| L-HDL | 0.536 | 0.450 | 0.621 | <0.001 | <0.001 | 0.309 | 0.202 | 0.416 | <0.001 | <0.001 |
| XL-HDL | -0.207 | -0.297 | -0.117 | <0.001 | <0.001 | -0.070 | -0.174 | 0.034 | 0.186 | 0.375 |
| **Total lipids** |  |  |  |  |  |  |  |  |  |  |
| VS-VLDL | -0.016 | -0.100 | 0.068 | 0.714 | 0.741 | 0.008 | -0.089 | 0.104 | 0.879 | 0.970 |
| S-VLDL | 0.039 | -0.045 | 0.124 | 0.359 | 0.399 | -0.012 | -0.108 | 0.085 | 0.814 | 0.943 |
| M-VLDL | -0.010 | -0.094 | 0.074 | 0.819 | 0.835 | -0.035 | -0.132 | 0.062 | 0.481 | 0.689 |
| L-VLDL | 0.135 | 0.049 | 0.221 | 0.002 | 0.004 | 0.031 | -0.069 | 0.131 | 0.544 | 0.753 |
| VL-VLDL | 0.192 | 0.106 | 0.278 | <0.001 | <0.001 | 0.077 | -0.024 | 0.177 | 0.134 | 0.290 |
| XL-VLDL | 0.236 | 0.151 | 0.322 | <0.001 | <0.001 | 0.122 | 0.023 | 0.220 | 0.016 | 0.056 |
| VLDL | 0.112 | 0.027 | 0.196 | 0.010 | 0.015 | 0.036 | -0.063 | 0.134 | 0.475 | 0.689 |
| S-LDL | -0.071 | -0.154 | 0.012 | 0.094 | 0.120 | -0.023 | -0.121 | 0.074 | 0.637 | 0.819 |
| M-LDL | -0.025 | -0.108 | 0.058 | 0.556 | 0.586 | 0.010 | -0.086 | 0.107 | 0.834 | 0.952 |
| L-LDL | -0.097 | -0.181 | -0.014 | 0.022 | 0.031 | -0.009 | -0.106 | 0.088 | 0.861 | 0.961 |
| LDL | -0.075 | -0.158 | 0.007 | 0.074 | 0.097 | -0.005 | -0.102 | 0.092 | 0.918 | 0.973 |
| IDL | -0.116 | -0.201 | -0.031 | 0.007 | 0.012 | 0.008 | -0.093 | 0.108 | 0.884 | 0.970 |
| S-HDL | 0.305 | 0.222 | 0.388 | <0.001 | <0.001 | 0.214 | 0.122 | 0.307 | <0.001 | <0.001 |
| M-HDL | 0.196 | 0.106 | 0.285 | <0.001 | <0.001 | 0.214 | 0.114 | 0.314 | <0.001 | 0.001 |
| L-HDL | -0.141 | -0.236 | -0.046 | 0.003 | 0.006 | 0.041 | -0.069 | 0.152 | 0.463 | 0.688 |
| XL-HDL | -0.250 | -0.343 | -0.157 | <0.001 | <0.001 | -0.044 | -0.154 | 0.065 | 0.425 | 0.650 |
| HDL | 0.066 | -0.027 | 0.158 | 0.164 | 0.196 | 0.163 | 0.058 | 0.268 | 0.002 | 0.014 |
| Total | 0.030 | -0.053 | 0.114 | 0.476 | 0.513 | 0.071 | -0.025 | 0.168 | 0.145 | 0.308 |
| **Triglycerides** |  |  |  |  |  |  |  |  |  |  |
| VS-VLDL | 0.213 | 0.129 | 0.296 | <0.001 | <0.001 | 0.096 | 0.002 | 0.191 | 0.046 | 0.118 |
| S-VLDL | 0.164 | 0.079 | 0.249 | <0.001 | <0.001 | 0.045 | -0.052 | 0.142 | 0.362 | 0.583 |
| M-VLDL | 0.121 | 0.035 | 0.206 | 0.006 | 0.009 | 0.023 | -0.075 | 0.121 | 0.645 | 0.819 |
| L-VLDL | 0.157 | 0.071 | 0.244 | <0.001 | 0.001 | 0.047 | -0.053 | 0.146 | 0.356 | 0.583 |
| XL-VLDL | 0.231 | 0.145 | 0.317 | <0.001 | <0.001 | 0.103 | 0.003 | 0.204 | 0.044 | 0.115 |
| XXL-VLDL | 0.239 | 0.154 | 0.325 | <0.001 | <0.001 | 0.128 | 0.029 | 0.226 | 0.011 | 0.044 |
| VLDL | 0.194 | 0.108 | 0.280 | <0.001 | <0.001 | 0.077 | -0.022 | 0.176 | 0.129 | 0.284 |
| S-LDL | 0.217 | 0.132 | 0.301 | <0.001 | <0.001 | 0.109 | 0.012 | 0.205 | 0.028 | 0.080 |
| M-LDL | 0.242 | 0.158 | 0.326 | <0.001 | <0.001 | 0.135 | 0.039 | 0.230 | 0.006 | 0.029 |
| L-LDL | 0.249 | 0.165 | 0.332 | <0.001 | <0.001 | 0.149 | 0.054 | 0.244 | 0.002 | 0.013 |
| LDL | 0.246 | 0.162 | 0.329 | <0.001 | <0.001 | 0.142 | 0.047 | 0.237 | 0.003 | 0.020 |
| IDL | 0.223 | 0.139 | 0.306 | <0.001 | <0.001 | 0.124 | 0.030 | 0.218 | 0.010 | 0.041 |
| S-HDL | 0.296 | 0.211 | 0.382 | <0.001 | <0.001 | 0.136 | 0.037 | 0.235 | 0.007 | 0.033 |
| M-HDL | 0.279 | 0.196 | 0.363 | <0.001 | <0.001 | 0.152 | 0.059 | 0.245 | 0.001 | 0.010 |
| L-HDL | 0.104 | 0.019 | 0.190 | 0.017 | 0.024 | 0.087 | -0.006 | 0.179 | 0.067 | 0.162 |
| XL-HDL | 0.077 | -0.006 | 0.161 | 0.069 | 0.090 | 0.054 | -0.038 | 0.145 | 0.251 | 0.467 |
| HDL | 0.247 | 0.164 | 0.330 | <0.001 | <0.001 | 0.131 | 0.038 | 0.224 | 0.006 | 0.029 |
| Total | 0.210 | 0.125 | 0.295 | <0.001 | <0.001 | 0.092 | -0.006 | 0.191 | 0.066 | 0.160 |
| **Triglycerides to Total lipids** | |  |  |  |  |  |  |  |  |  |
| VS-VLDL | 0.400 | 0.314 | 0.486 | <0.001 | <0.001 | 0.185 | 0.080 | 0.290 | 0.001 | 0.006 |
| S-VLDL | 0.293 | 0.209 | 0.377 | <0.001 | <0.001 | 0.123 | 0.024 | 0.221 | 0.014 | 0.054 |
| M-VLDL | 0.336 | 0.250 | 0.423 | <0.001 | <0.001 | 0.124 | 0.021 | 0.228 | 0.019 | 0.060 |
| L-VLDL | 0.051 | -0.031 | 0.134 | 0.223 | 0.261 | 0.045 | -0.046 | 0.136 | 0.334 | 0.561 |
| XL-VLDL | 0.246 | 0.162 | 0.331 | <0.001 | <0.001 | 0.105 | 0.009 | 0.202 | 0.032 | 0.089 |
| XXL-VLDL | 0.011 | -0.072 | 0.094 | 0.795 | 0.818 | -0.001 | -0.091 | 0.089 | 0.981 | 0.991 |
| S-LDL | 0.333 | 0.249 | 0.417 | <0.001 | <0.001 | 0.154 | 0.057 | 0.250 | 0.002 | 0.012 |
| M-LDL | 0.335 | 0.252 | 0.418 | <0.001 | <0.001 | 0.159 | 0.063 | 0.254 | 0.001 | 0.009 |
| L-LDL | 0.372 | 0.289 | 0.456 | <0.001 | <0.001 | 0.164 | 0.067 | 0.261 | 0.001 | 0.007 |
| IDL | 0.366 | 0.282 | 0.451 | <0.001 | <0.001 | 0.140 | 0.039 | 0.242 | 0.007 | 0.032 |
| S-HDL | 0.205 | 0.118 | 0.291 | <0.001 | <0.001 | 0.049 | -0.052 | 0.149 | 0.340 | 0.565 |
| M-HDL | 0.195 | 0.109 | 0.280 | <0.001 | <0.001 | 0.053 | -0.045 | 0.152 | 0.291 | 0.518 |
| L-HDL | 0.210 | 0.123 | 0.297 | <0.001 | <0.001 | 0.055 | -0.046 | 0.156 | 0.289 | 0.518 |
| XL-HDL | 0.317 | 0.231 | 0.404 | <0.001 | <0.001 | 0.133 | 0.028 | 0.238 | 0.013 | 0.050 |

† Model adjusted for age, sex, and education.
‡ Model adjusted for age, sex, education, race, socioeconomic status, body mass index, smoking status, alcohol drinking status, physical activity, social connection, hypertension, diabetes, heart disease, beta-blockers, calcium blockers, lipid-lowering, and APOE ε4 status.
Abbreviation: Apo, apolipoprotein; LDL-AD, average diameter for LDL particles; CHOL, cholesterol; CE, cholesteryl esters; TG, triglycerides; S, small; M, medium; L, large; VL, very large; XL, extremely large; VLDL, very low-density lipoprotein; LDL; low-density lipoprotein; IDL, intermediate-density lipoprotein; HDL, high-density lipoprotein; LA, linoleic acid; MUFA, monounsaturated fatty acid; n-6, omega-6 fatty acid; PUFA, polyunsaturated fatty acid; SFA, saturated fatty acid; FA, fatty acid.

**Table S9.** β coefficients and 95% confidence intervals (CIs) for the association between metabolites and brain age among non-APOE ε4 carriers: results from linear regression models

| **Metabolites** | **Basic-model^†^** | |  |  |  | **Multi-model^‡^** | |  |  |  |
| --- | --- | --- | --- | --- | --- | --- | --- | --- | --- | --- |
|  | **β** | **Lower** | **Upper** | ***P*-value** | **FDR-q** | **β** | **Lower** | **Upper** | ***P*-value** | **FDR-q** |
| **Amino acids & Glycolysis** |  |  |  |  |  |  |  |  |  |  |
| Valine | 0.128 | 0.021 | 0.234 | 0.019 | 0.034 | 0.046 | -0.065 | 0.157 | 0.420 | 0.685 |
| Leucine | 0.132 | 0.024 | 0.239 | 0.016 | 0.030 | 0.074 | -0.036 | 0.184 | 0.189 | 0.390 |
| Isoleucine | 0.077 | -0.028 | 0.182 | 0.148 | 0.211 | 0.026 | -0.082 | 0.133 | 0.640 | 0.829 |
| Phenylalanine | 0.119 | 0.014 | 0.224 | 0.026 | 0.045 | 0.078 | -0.028 | 0.185 | 0.150 | 0.333 |
| Tyrosine | 0.229 | 0.126 | 0.332 | <0.001 | <0.001 | 0.194 | 0.087 | 0.300 | <0.001 | 0.004 |
| Alanine | 0.049 | -0.055 | 0.153 | 0.357 | 0.440 | -0.009 | -0.115 | 0.097 | 0.872 | 0.928 |
| Glutamine | -0.315 | -0.419 | -0.211 | <0.001 | <0.001 | -0.223 | -0.330 | -0.116 | <0.001 | 0.001 |
| Glycine | -0.024 | -0.135 | 0.087 | 0.670 | 0.722 | 0.043 | -0.071 | 0.158 | 0.457 | 0.711 |
| Histidine | -0.025 | -0.128 | 0.078 | 0.631 | 0.689 | 0.006 | -0.098 | 0.110 | 0.912 | 0.946 |
| BCAAs | 0.123 | 0.017 | 0.230 | 0.023 | 0.041 | 0.053 | -0.058 | 0.163 | 0.350 | 0.608 |
| Lactate | 0.080 | -0.026 | 0.185 | 0.138 | 0.199 | 0.010 | -0.096 | 0.117 | 0.848 | 0.910 |
| Pyruvate | 0.014 | -0.090 | 0.118 | 0.792 | 0.836 | -0.038 | -0.144 | 0.067 | 0.478 | 0.713 |
| Glucose | 0.310 | 0.208 | 0.412 | <0.001 | <0.001 | 0.136 | 0.026 | 0.246 | 0.015 | 0.072 |
| Citrate | -0.075 | -0.180 | 0.030 | 0.163 | 0.228 | -0.047 | -0.154 | 0.061 | 0.393 | 0.647 |
| **Apo-LP, LP size & Other lipids** | |  |  |  |  |  |  |  |  |  |
| ApoA1 | 0.179 | 0.066 | 0.292 | 0.002 | 0.004 | 0.230 | 0.113 | 0.348 | <0.001 | 0.002 |
| ApoB | -0.087 | -0.194 | 0.019 | 0.107 | 0.160 | -0.075 | -0.189 | 0.040 | 0.201 | 0.405 |
| ApoB/ApoA1 | -0.166 | -0.276 | -0.057 | 0.003 | 0.006 | -0.186 | -0.304 | -0.068 | 0.002 | 0.016 |
| HDL-AD | -0.157 | -0.274 | -0.039 | 0.009 | 0.017 | 0.041 | -0.087 | 0.169 | 0.531 | 0.751 |
| LDL-AD | -0.196 | -0.304 | -0.089 | <0.001 | 0.001 | -0.124 | -0.235 | -0.012 | 0.029 | 0.105 |
| VLDL-AD | 0.183 | 0.071 | 0.296 | 0.001 | 0.003 | 0.028 | -0.092 | 0.149 | 0.646 | 0.829 |
| Phosphatidyl choline | 0.158 | 0.047 | 0.268 | 0.005 | 0.011 | 0.170 | 0.056 | 0.284 | 0.004 | 0.026 |
| Phosphoglycerides | 0.189 | 0.080 | 0.299 | 0.001 | 0.002 | 0.175 | 0.062 | 0.288 | 0.002 | 0.018 |
| Sphingomyeline | -0.008 | -0.119 | 0.103 | 0.888 | 0.910 | 0.063 | -0.054 | 0.180 | 0.292 | 0.535 |
| Total choline | 0.133 | 0.023 | 0.243 | 0.018 | 0.033 | 0.149 | 0.035 | 0.263 | 0.011 | 0.059 |
| TG/Phosphoglycerides | 0.184 | 0.069 | 0.298 | 0.002 | 0.004 | 0.009 | -0.115 | 0.133 | 0.892 | 0.940 |
| **Cholesterol** |  |  |  |  |  |  |  |  |  |  |
| XS-VLDL | -0.103 | -0.212 | 0.005 | 0.063 | 0.099 | -0.054 | -0.171 | 0.063 | 0.364 | 0.616 |
| S-VLDL | -0.057 | -0.163 | 0.049 | 0.295 | 0.373 | -0.089 | -0.202 | 0.024 | 0.122 | 0.296 |
| M-VLDL | -0.181 | -0.287 | -0.075 | 0.001 | 0.002 | -0.143 | -0.257 | -0.028 | 0.015 | 0.070 |
| L-VLDL | 0.065 | -0.043 | 0.174 | 0.238 | 0.315 | -0.043 | -0.159 | 0.073 | 0.467 | 0.713 |
| VL-VLDL | 0.089 | -0.019 | 0.198 | 0.107 | 0.160 | -0.022 | -0.139 | 0.095 | 0.709 | 0.869 |
| XL-VLDL | 0.207 | 0.098 | 0.316 | <0.001 | 0.001 | 0.071 | -0.046 | 0.187 | 0.234 | 0.448 |
| VLDL | -0.025 | -0.131 | 0.081 | 0.649 | 0.706 | -0.066 | -0.180 | 0.048 | 0.256 | 0.482 |
| S-LDL | -0.056 | -0.162 | 0.050 | 0.302 | 0.378 | -0.047 | -0.161 | 0.067 | 0.421 | 0.685 |
| M-LDL | -0.012 | -0.117 | 0.094 | 0.831 | 0.869 | -0.015 | -0.127 | 0.098 | 0.799 | 0.889 |
| L-LDL | -0.083 | -0.190 | 0.024 | 0.127 | 0.186 | -0.029 | -0.143 | 0.085 | 0.616 | 0.808 |
| Clinical-LDL | -0.091 | -0.197 | 0.015 | 0.094 | 0.143 | -0.042 | -0.156 | 0.072 | 0.471 | 0.713 |
| LDL | -0.062 | -0.168 | 0.044 | 0.254 | 0.330 | -0.027 | -0.140 | 0.086 | 0.637 | 0.829 |
| IDL | -0.098 | -0.207 | 0.012 | 0.080 | 0.125 | -0.003 | -0.122 | 0.116 | 0.958 | 0.974 |
| S-HDL | 0.256 | 0.153 | 0.360 | <0.001 | <0.001 | 0.189 | 0.083 | 0.295 | <0.001 | 0.005 |
| M-HDL | 0.176 | 0.064 | 0.289 | 0.002 | 0.005 | 0.237 | 0.118 | 0.355 | <0.001 | 0.001 |
| L-HDL | -0.150 | -0.267 | -0.033 | 0.012 | 0.022 | 0.050 | -0.078 | 0.179 | 0.441 | 0.696 |
| XL-HDL | -0.246 | -0.360 | -0.131 | <0.001 | <0.001 | -0.035 | -0.161 | 0.091 | 0.583 | 0.794 |
| HDL | 0.027 | -0.089 | 0.142 | 0.653 | 0.707 | 0.166 | 0.043 | 0.290 | 0.008 | 0.049 |
| Total | -0.050 | -0.158 | 0.058 | 0.360 | 0.442 | 0.019 | -0.096 | 0.135 | 0.743 | 0.882 |
| Remnant | -0.062 | -0.168 | 0.044 | 0.254 | 0.330 | -0.042 | -0.157 | 0.074 | 0.477 | 0.713 |
| Total-HDLC | -0.063 | -0.169 | 0.043 | 0.245 | 0.321 | -0.035 | -0.149 | 0.080 | 0.549 | 0.761 |
| **Cholesterol to Total lipids** |  |  |  |  |  |  |  |  |  |  |
| VS-VLDL | -0.368 | -0.479 | -0.257 | <0.001 | <0.001 | -0.152 | -0.276 | -0.027 | 0.017 | 0.073 |
| S-VLDL | -0.284 | -0.391 | -0.177 | <0.001 | <0.001 | -0.145 | -0.260 | -0.030 | 0.013 | 0.065 |
| M-VLDL | -0.338 | -0.449 | -0.227 | <0.001 | <0.001 | -0.140 | -0.261 | -0.018 | 0.025 | 0.093 |
| L-VLDL | -0.207 | -0.312 | -0.101 | <0.001 | <0.001 | -0.106 | -0.216 | 0.003 | 0.056 | 0.157 |
| XL-VLDL | -0.237 | -0.345 | -0.130 | <0.001 | <0.001 | -0.079 | -0.193 | 0.034 | 0.172 | 0.360 |
| XXL-VLDL | -0.061 | -0.166 | 0.045 | 0.259 | 0.334 | -0.015 | -0.123 | 0.092 | 0.777 | 0.889 |
| S-LDL | -0.121 | -0.224 | -0.019 | 0.020 | 0.036 | -0.039 | -0.146 | 0.068 | 0.476 | 0.713 |
| M-LDL | -0.256 | -0.358 | -0.153 | <0.001 | <0.001 | -0.120 | -0.229 | -0.011 | 0.031 | 0.109 |
| L-LDL | -0.315 | -0.418 | -0.211 | <0.001 | <0.001 | -0.134 | -0.245 | -0.022 | 0.019 | 0.076 |
| IDL | -0.220 | -0.327 | -0.112 | <0.001 | <0.001 | -0.038 | -0.153 | 0.078 | 0.524 | 0.746 |
| S-HDL | -0.270 | -0.376 | -0.164 | <0.001 | <0.001 | -0.130 | -0.239 | -0.020 | 0.020 | 0.079 |
| M-HDL | -0.222 | -0.332 | -0.113 | <0.001 | <0.001 | -0.043 | -0.162 | 0.075 | 0.472 | 0.713 |
| L-HDL | -0.408 | -0.518 | -0.298 | <0.001 | <0.001 | -0.177 | -0.300 | -0.054 | 0.005 | 0.032 |
| XL-HDL | 0.053 | -0.057 | 0.163 | 0.347 | 0.431 | -0.007 | -0.120 | 0.106 | 0.906 | 0.946 |
| **Cholesteryl esters to Total lipids** | |  |  |  |  |  |  |  |  |  |
| VS-VLDL | -0.354 | -0.465 | -0.242 | <0.001 | <0.001 | -0.128 | -0.254 | -0.003 | 0.045 | 0.136 |
| S-VLDL | -0.236 | -0.341 | -0.131 | <0.001 | <0.001 | -0.140 | -0.250 | -0.029 | 0.013 | 0.065 |
| M-VLDL | -0.343 | -0.454 | -0.231 | <0.001 | <0.001 | -0.141 | -0.263 | -0.018 | 0.024 | 0.092 |
| L-VLDL | -0.291 | -0.400 | -0.182 | <0.001 | <0.001 | -0.139 | -0.254 | -0.024 | 0.018 | 0.073 |
| XL-VLDL | -0.223 | -0.330 | -0.115 | <0.001 | <0.001 | -0.073 | -0.186 | 0.040 | 0.204 | 0.406 |
| XXL-VLDL | -0.074 | -0.180 | 0.032 | 0.169 | 0.235 | -0.028 | -0.135 | 0.080 | 0.612 | 0.806 |
| S-LDL | 0.121 | 0.018 | 0.224 | 0.021 | 0.037 | 0.060 | -0.046 | 0.167 | 0.268 | 0.498 |
| M-LDL | 0.075 | -0.030 | 0.180 | 0.161 | 0.227 | 0.016 | -0.094 | 0.126 | 0.775 | 0.889 |
| L-LDL | -0.130 | -0.231 | -0.030 | 0.011 | 0.021 | -0.077 | -0.179 | 0.026 | 0.143 | 0.328 |
| IDL | -0.122 | -0.229 | -0.016 | 0.024 | 0.042 | 0.023 | -0.090 | 0.136 | 0.688 | 0.862 |
| S-HDL | -0.220 | -0.326 | -0.114 | <0.001 | <0.001 | -0.117 | -0.225 | -0.008 | 0.035 | 0.115 |
| M-HDL | -0.233 | -0.341 | -0.125 | <0.001 | <0.001 | -0.072 | -0.187 | 0.043 | 0.220 | 0.424 |
| L-HDL | -0.417 | -0.527 | -0.307 | <0.001 | <0.001 | -0.195 | -0.316 | -0.074 | 0.002 | 0.013 |
| XL-HDL | -0.066 | -0.171 | 0.039 | 0.216 | 0.290 | 0.007 | -0.100 | 0.114 | 0.895 | 0.940 |
| **Cholestryl esters** |  |  |  |  |  |  |  |  |  |  |
| VS-VLDL | -0.127 | -0.236 | -0.017 | 0.023 | 0.040 | -0.057 | -0.175 | 0.061 | 0.345 | 0.605 |
| S-VLDL | -0.038 | -0.144 | 0.068 | 0.482 | 0.550 | -0.083 | -0.197 | 0.030 | 0.148 | 0.332 |
| M-VLDL | -0.240 | -0.347 | -0.134 | <0.001 | <0.001 | -0.166 | -0.282 | -0.051 | 0.005 | 0.032 |
| L-VLDL | -0.005 | -0.112 | 0.102 | 0.931 | 0.950 | -0.085 | -0.199 | 0.029 | 0.145 | 0.328 |
| VL-VLDL | 0.033 | -0.075 | 0.141 | 0.545 | 0.608 | -0.059 | -0.176 | 0.057 | 0.320 | 0.569 |
| XL-VLDL | 0.186 | 0.077 | 0.296 | 0.001 | 0.002 | 0.056 | -0.061 | 0.172 | 0.351 | 0.608 |
| VLDL | -0.070 | -0.175 | 0.036 | 0.196 | 0.266 | -0.087 | -0.201 | 0.027 | 0.135 | 0.320 |
| S-LDL | -0.018 | -0.124 | 0.089 | 0.743 | 0.794 | -0.030 | -0.144 | 0.084 | 0.611 | 0.806 |
| M-LDL | 0.018 | -0.088 | 0.124 | 0.738 | 0.792 | -0.003 | -0.116 | 0.110 | 0.961 | 0.974 |
| L-LDL | -0.068 | -0.174 | 0.039 | 0.213 | 0.288 | -0.026 | -0.139 | 0.087 | 0.656 | 0.833 |
| LDL | -0.040 | -0.146 | 0.066 | 0.458 | 0.526 | -0.020 | -0.133 | 0.093 | 0.725 | 0.876 |
| IDL | -0.084 | -0.193 | 0.025 | 0.131 | 0.191 | 0.007 | -0.112 | 0.126 | 0.911 | 0.946 |
| S-HDL | 0.239 | 0.135 | 0.342 | <0.001 | <0.001 | 0.173 | 0.067 | 0.279 | 0.001 | 0.012 |
| M-HDL | 0.173 | 0.061 | 0.286 | 0.003 | 0.006 | 0.234 | 0.116 | 0.352 | <0.001 | 0.002 |
| L-HDL | -0.160 | -0.276 | -0.043 | 0.007 | 0.015 | 0.043 | -0.085 | 0.171 | 0.511 | 0.741 |
| XL-HDL | -0.241 | -0.356 | -0.126 | <0.001 | <0.001 | -0.028 | -0.155 | 0.098 | 0.661 | 0.836 |
| HDL | 0.018 | -0.097 | 0.133 | 0.757 | 0.803 | 0.160 | 0.037 | 0.284 | 0.011 | 0.059 |
| Total | -0.044 | -0.153 | 0.064 | 0.422 | 0.493 | 0.031 | -0.084 | 0.147 | 0.595 | 0.801 |
| **Fatty acids** |  |  |  |  |  |  |  |  |  |  |
| DHA | -0.042 | -0.150 | 0.066 | 0.448 | 0.518 | 0.014 | -0.097 | 0.125 | 0.804 | 0.890 |
| LA | -0.070 | -0.175 | 0.036 | 0.194 | 0.266 | -0.037 | -0.147 | 0.074 | 0.512 | 0.741 |
| MUFA | 0.274 | 0.167 | 0.381 | <0.001 | <0.001 | 0.127 | 0.014 | 0.241 | 0.027 | 0.100 |
| n-3 | 0.041 | -0.066 | 0.149 | 0.452 | 0.521 | 0.036 | -0.073 | 0.145 | 0.520 | 0.746 |
| n-6 | <0.001 | -0.106 | 0.105 | 0.993 | 0.993 | 0.003 | -0.108 | 0.113 | 0.962 | 0.974 |
| PUFA | 0.011 | -0.096 | 0.118 | 0.841 | 0.876 | 0.012 | -0.098 | 0.123 | 0.824 | 0.900 |
| SFA | 0.287 | 0.181 | 0.394 | <0.001 | <0.001 | 0.181 | 0.070 | 0.293 | 0.001 | 0.013 |
| Total | 0.208 | 0.102 | 0.314 | <0.001 | <0.001 | 0.116 | 0.005 | 0.227 | 0.040 | 0.125 |
| Unsaturation | -0.282 | -0.391 | -0.173 | <0.001 | <0.001 | -0.139 | -0.253 | -0.024 | 0.018 | 0.073 |
| DHA/FA | -0.144 | -0.252 | -0.037 | 0.008 | 0.016 | -0.038 | -0.151 | 0.074 | 0.502 | 0.735 |
| LA/FA | -0.470 | -0.578 | -0.363 | <0.001 | <0.001 | -0.286 | -0.405 | -0.167 | <0.001 | <0.001 |
| MUFA/FA | 0.320 | 0.209 | 0.430 | <0.001 | <0.001 | 0.106 | -0.015 | 0.227 | 0.085 | 0.221 |
| n-3/FA | -0.047 | -0.154 | 0.059 | 0.383 | 0.461 | -0.001 | -0.110 | 0.108 | 0.984 | 0.988 |
| n-6/n-3 | 0.012 | -0.094 | 0.117 | 0.828 | 0.869 | 0.025 | -0.083 | 0.132 | 0.652 | 0.833 |
| n-6/FA | -0.440 | -0.549 | -0.332 | <0.001 | <0.001 | -0.264 | -0.381 | -0.147 | <0.001 | <0.001 |
| PUFA/MUFA | -0.360 | -0.469 | -0.250 | <0.001 | <0.001 | -0.153 | -0.273 | -0.033 | 0.012 | 0.065 |
| PUFA/FA | -0.448 | -0.557 | -0.340 | <0.001 | <0.001 | -0.262 | -0.380 | -0.143 | <0.001 | <0.001 |
| SFA/FA | 0.397 | 0.292 | 0.502 | <0.001 | <0.001 | 0.298 | 0.189 | 0.407 | <0.001 | <0.001 |
| **Free cholesterol** |  |  |  |  |  |  |  |  |  |  |
| VS-VLDL | -0.046 | -0.153 | 0.061 | 0.401 | 0.477 | -0.046 | -0.161 | 0.068 | 0.427 | 0.686 |
| S-VLDL | -0.088 | -0.194 | 0.018 | 0.103 | 0.156 | -0.097 | -0.210 | 0.016 | 0.092 | 0.233 |
| M-VLDL | -0.097 | -0.202 | 0.009 | 0.073 | 0.115 | -0.105 | -0.219 | 0.008 | 0.070 | 0.190 |
| L-VLDL | 0.133 | 0.023 | 0.243 | 0.018 | 0.032 | <0.001 | -0.118 | 0.118 | 0.999 | 0.999 |
| VL-VLDL | 0.145 | 0.036 | 0.255 | 0.009 | 0.018 | 0.017 | -0.100 | 0.134 | 0.774 | 0.889 |
| XL-VLDL | 0.229 | 0.120 | 0.338 | <0.001 | <0.001 | 0.088 | -0.028 | 0.203 | 0.138 | 0.324 |
| VLDL | 0.037 | -0.069 | 0.144 | 0.492 | 0.557 | -0.036 | -0.151 | 0.078 | 0.534 | 0.751 |
| S-LDL | -0.151 | -0.257 | -0.045 | 0.005 | 0.011 | -0.086 | -0.199 | 0.027 | 0.134 | 0.320 |
| M-LDL | -0.092 | -0.198 | 0.014 | 0.090 | 0.138 | -0.046 | -0.158 | 0.067 | 0.424 | 0.685 |
| L-LDL | -0.124 | -0.231 | -0.016 | 0.024 | 0.042 | -0.038 | -0.153 | 0.078 | 0.522 | 0.746 |
| LDL | -0.119 | -0.226 | -0.012 | 0.029 | 0.049 | -0.046 | -0.160 | 0.069 | 0.434 | 0.693 |
| IDL | -0.133 | -0.242 | -0.024 | 0.017 | 0.032 | -0.031 | -0.150 | 0.088 | 0.607 | 0.806 |
| S-HDL | 0.279 | 0.174 | 0.384 | <0.001 | <0.001 | 0.212 | 0.104 | 0.320 | <0.001 | 0.002 |
| M-HDL | 0.185 | 0.071 | 0.298 | 0.001 | 0.003 | 0.240 | 0.121 | 0.358 | <0.001 | 0.001 |
| L-HDL | -0.115 | -0.232 | 0.003 | 0.055 | 0.088 | 0.075 | -0.053 | 0.202 | 0.251 | 0.478 |
| XL-HDL | -0.252 | -0.363 | -0.140 | <0.001 | <0.001 | -0.057 | -0.178 | 0.064 | 0.354 | 0.608 |
| HDL | 0.055 | -0.061 | 0.171 | 0.354 | 0.439 | 0.179 | 0.056 | 0.302 | 0.004 | 0.030 |
| Total | -0.064 | -0.172 | 0.043 | 0.240 | 0.316 | -0.011 | -0.127 | 0.104 | 0.848 | 0.910 |
| **Free cholesterol to Total lipids** | |  |  |  |  |  |  |  |  |  |
| VS-VLDL | -0.356 | -0.462 | -0.250 | <0.001 | <0.001 | -0.232 | -0.344 | -0.119 | <0.001 | 0.001 |
| S-VLDL | -0.301 | -0.410 | -0.191 | <0.001 | <0.001 | -0.125 | -0.244 | -0.006 | 0.039 | 0.124 |
| M-VLDL | -0.310 | -0.420 | -0.200 | <0.001 | <0.001 | -0.129 | -0.249 | -0.009 | 0.035 | 0.116 |
| L-VLDL | 0.028 | -0.074 | 0.130 | 0.591 | 0.654 | -0.019 | -0.124 | 0.086 | 0.721 | 0.875 |
| XL-VLDL | -0.215 | -0.321 | -0.108 | <0.001 | <0.001 | -0.073 | -0.184 | 0.038 | 0.198 | 0.404 |
| XXL-VLDL | -0.017 | -0.122 | 0.089 | 0.758 | 0.803 | 0.015 | -0.093 | 0.123 | 0.786 | 0.889 |
| S-LDL | -0.273 | -0.380 | -0.167 | <0.001 | <0.001 | -0.114 | -0.225 | -0.002 | 0.046 | 0.137 |
| M-LDL | -0.316 | -0.425 | -0.207 | <0.001 | <0.001 | -0.129 | -0.246 | -0.012 | 0.031 | 0.107 |
| L-LDL | -0.320 | -0.429 | -0.212 | <0.001 | <0.001 | -0.104 | -0.223 | 0.015 | 0.087 | 0.224 |
| IDL | -0.292 | -0.398 | -0.186 | <0.001 | <0.001 | -0.134 | -0.245 | -0.024 | 0.017 | 0.073 |
| S-HDL | -0.187 | -0.296 | -0.079 | 0.001 | 0.002 | -0.039 | -0.154 | 0.075 | 0.501 | 0.735 |
| M-HDL | -0.064 | -0.180 | 0.052 | 0.278 | 0.354 | 0.110 | -0.015 | 0.234 | 0.085 | 0.221 |
| L-HDL | -0.182 | -0.292 | -0.071 | 0.001 | 0.003 | -0.006 | -0.125 | 0.113 | 0.926 | 0.952 |
| XL-HDL | 0.154 | 0.040 | 0.269 | 0.008 | 0.016 | -0.020 | -0.144 | 0.103 | 0.747 | 0.882 |
| **KB, FB & Inflammation** |  |  |  |  |  |  |  |  |  |  |
| Glycoprotein acetyls | 0.308 | 0.203 | 0.413 | <0.001 | <0.001 | 0.137 | 0.024 | 0.251 | 0.017 | 0.073 |
| 3-Hydrocybutyrate | 0.140 | 0.038 | 0.243 | 0.007 | 0.015 | 0.131 | 0.026 | 0.236 | 0.014 | 0.069 |
| Acetate | -0.129 | -0.223 | -0.036 | 0.007 | 0.013 | -0.102 | -0.195 | -0.008 | 0.034 | 0.114 |
| Acetoacetate | 0.202 | 0.098 | 0.305 | <0.001 | <0.001 | 0.157 | 0.052 | 0.262 | 0.003 | 0.026 |
| Acetone | 0.141 | 0.037 | 0.245 | 0.008 | 0.016 | 0.144 | 0.039 | 0.250 | 0.007 | 0.045 |
| Albumin | 0.064 | -0.041 | 0.169 | 0.230 | 0.308 | 0.059 | -0.048 | 0.166 | 0.277 | 0.512 |
| Creatinine | -0.054 | -0.181 | 0.073 | 0.407 | 0.483 | -0.129 | -0.259 | 0.002 | 0.054 | 0.155 |
| **Lipoprotein particles** |  |  |  |  |  |  |  |  |  |  |
| VS-VLDL | -0.011 | -0.118 | 0.096 | 0.847 | 0.878 | -0.022 | -0.136 | 0.092 | 0.708 | 0.869 |
| S-VLDL | 0.048 | -0.059 | 0.156 | 0.381 | 0.461 | -0.044 | -0.157 | 0.070 | 0.450 | 0.705 |
| M-VLDL | -0.044 | -0.151 | 0.062 | 0.414 | 0.488 | -0.082 | -0.196 | 0.031 | 0.156 | 0.338 |
| L-VLDL | 0.152 | 0.042 | 0.262 | 0.007 | 0.014 | 0.017 | -0.101 | 0.134 | 0.781 | 0.889 |
| VL-VLDL | 0.201 | 0.091 | 0.311 | <0.001 | 0.001 | 0.053 | -0.065 | 0.172 | 0.379 | 0.630 |
| XL-VLDL | 0.249 | 0.140 | 0.359 | <0.001 | <0.001 | 0.104 | -0.012 | 0.221 | 0.079 | 0.211 |
| VLDL | 0.034 | -0.073 | 0.140 | 0.535 | 0.600 | -0.035 | -0.148 | 0.079 | 0.550 | 0.761 |
| S-LDL | -0.078 | -0.185 | 0.029 | 0.151 | 0.214 | -0.073 | -0.188 | 0.041 | 0.211 | 0.410 |
| M-LDL | -0.043 | -0.150 | 0.063 | 0.425 | 0.494 | -0.042 | -0.155 | 0.072 | 0.473 | 0.713 |
| L-LDL | -0.135 | -0.241 | -0.028 | 0.013 | 0.024 | -0.111 | -0.225 | 0.003 | 0.056 | 0.157 |
| LDL | -0.106 | -0.212 | <0.001 | 0.050 | 0.081 | -0.091 | -0.205 | 0.024 | 0.120 | 0.292 |
| IDL | -0.074 | -0.181 | 0.033 | 0.176 | 0.243 | -0.023 | -0.140 | 0.094 | 0.703 | 0.869 |
| S-HDL | 0.265 | 0.162 | 0.368 | <0.001 | <0.001 | 0.184 | 0.078 | 0.290 | 0.001 | 0.007 |
| M-HDL | 0.210 | 0.097 | 0.322 | <0.001 | 0.001 | 0.246 | 0.129 | 0.363 | <0.001 | 0.001 |
| L-HDL | -0.126 | -0.243 | -0.009 | 0.035 | 0.058 | 0.065 | -0.063 | 0.193 | 0.317 | 0.567 |
| XL-HDL | -0.218 | -0.332 | -0.103 | <0.001 | 0.001 | -0.021 | -0.145 | 0.104 | 0.745 | 0.882 |
| HDL | 0.195 | 0.086 | 0.305 | <0.001 | 0.001 | 0.218 | 0.105 | 0.331 | <0.001 | 0.002 |
| Total | 0.172 | 0.063 | 0.281 | 0.002 | 0.005 | 0.196 | 0.084 | 0.309 | 0.001 | 0.007 |
| **Phospholipids** |  |  |  |  |  |  |  |  |  |  |
| VS-VLDL | 0.004 | -0.103 | 0.111 | 0.939 | 0.954 | -0.030 | -0.143 | 0.083 | 0.601 | 0.804 |
| S-VLDL | -0.027 | -0.133 | 0.079 | 0.617 | 0.680 | -0.073 | -0.186 | 0.040 | 0.208 | 0.409 |
| M-VLDL | -0.048 | -0.154 | 0.058 | 0.374 | 0.456 | -0.080 | -0.193 | 0.034 | 0.168 | 0.354 |
| L-VLDL | 0.156 | 0.046 | 0.266 | 0.006 | 0.011 | 0.014 | -0.104 | 0.132 | 0.813 | 0.896 |
| VL-VLDL | 0.172 | 0.062 | 0.282 | 0.002 | 0.005 | 0.035 | -0.083 | 0.153 | 0.560 | 0.770 |
| XL-VLDL | 0.244 | 0.135 | 0.354 | <0.001 | <0.001 | 0.097 | -0.019 | 0.214 | 0.100 | 0.253 |
| VLDL | 0.079 | -0.028 | 0.187 | 0.148 | 0.211 | -0.015 | -0.130 | 0.100 | 0.800 | 0.889 |
| S-LDL | -0.090 | -0.197 | 0.017 | 0.101 | 0.153 | -0.073 | -0.187 | 0.041 | 0.209 | 0.409 |
| M-LDL | 0.001 | -0.106 | 0.107 | 0.989 | 0.993 | -0.011 | -0.124 | 0.102 | 0.844 | 0.910 |
| L-LDL | -0.061 | -0.167 | 0.045 | 0.262 | 0.334 | -0.017 | -0.131 | 0.097 | 0.765 | 0.889 |
| LDL | -0.047 | -0.153 | 0.059 | 0.386 | 0.462 | -0.023 | -0.137 | 0.090 | 0.689 | 0.862 |
| IDL | -0.112 | -0.221 | -0.002 | 0.046 | 0.074 | -0.037 | -0.155 | 0.082 | 0.543 | 0.759 |
| S-HDL | 0.386 | 0.282 | 0.491 | <0.001 | <0.001 | 0.265 | 0.157 | 0.373 | <0.001 | <0.001 |
| M-HDL | 0.305 | 0.194 | 0.416 | <0.001 | <0.001 | 0.284 | 0.170 | 0.399 | <0.001 | <0.001 |
| L-HDL | -0.038 | -0.156 | 0.079 | 0.521 | 0.587 | 0.124 | -0.002 | 0.251 | 0.054 | 0.155 |
| XL-HDL | -0.210 | -0.325 | -0.095 | <0.001 | 0.001 | -0.015 | -0.140 | 0.111 | 0.820 | 0.899 |
| HDL | 0.189 | 0.074 | 0.304 | 0.001 | 0.003 | 0.243 | 0.124 | 0.363 | <0.001 | 0.001 |
| Total | 0.118 | 0.009 | 0.227 | 0.034 | 0.055 | 0.134 | 0.020 | 0.249 | 0.021 | 0.080 |
| **Phospholipids to Total lipids** |  |  |  |  |  |  |  |  |  |  |
| VS-VLDL | 0.008 | -0.098 | 0.113 | 0.887 | 0.910 | -0.139 | -0.248 | -0.029 | 0.013 | 0.065 |
| S-VLDL | -0.292 | -0.401 | -0.182 | <0.001 | <0.001 | -0.122 | -0.240 | -0.004 | 0.043 | 0.133 |
| M-VLDL | -0.240 | -0.349 | -0.132 | <0.001 | <0.001 | -0.083 | -0.201 | 0.035 | 0.167 | 0.354 |
| L-VLDL | 0.107 | 0.003 | 0.211 | 0.044 | 0.072 | -0.014 | -0.124 | 0.095 | 0.800 | 0.889 |
| VL-VLDL | -0.057 | -0.157 | 0.043 | 0.262 | 0.334 | -0.075 | -0.178 | 0.028 | 0.154 | 0.335 |
| XL-VLDL | 0.092 | -0.011 | 0.196 | 0.080 | 0.125 | -0.014 | -0.121 | 0.092 | 0.795 | 0.889 |
| S-LDL | -0.148 | -0.251 | -0.046 | 0.005 | 0.010 | -0.086 | -0.192 | 0.020 | 0.111 | 0.273 |
| M-LDL | -0.063 | -0.167 | 0.041 | 0.234 | 0.311 | -0.052 | -0.158 | 0.055 | 0.341 | 0.602 |
| L-LDL | -0.031 | -0.133 | 0.072 | 0.555 | 0.617 | -0.030 | -0.135 | 0.074 | 0.567 | 0.776 |
| IDL | -0.170 | -0.272 | -0.068 | 0.001 | 0.003 | -0.177 | -0.281 | -0.074 | 0.001 | 0.008 |
| S-HDL | 0.244 | 0.134 | 0.354 | <0.001 | <0.001 | 0.190 | 0.078 | 0.302 | 0.001 | 0.009 |
| M-HDL | 0.261 | 0.152 | 0.371 | <0.001 | <0.001 | 0.069 | -0.051 | 0.188 | 0.260 | 0.486 |
| L-HDL | 0.532 | 0.424 | 0.641 | <0.001 | <0.001 | 0.306 | 0.181 | 0.430 | <0.001 | <0.001 |
| XL-HDL | -0.200 | -0.312 | -0.087 | 0.001 | 0.001 | -0.056 | -0.174 | 0.063 | 0.358 | 0.610 |
| **Total lipids** |  |  |  |  |  |  |  |  |  |  |
| VS-VLDL | 0.001 | -0.106 | 0.108 | 0.987 | 0.993 | -0.016 | -0.130 | 0.097 | 0.777 | 0.889 |
| S-VLDL | 0.048 | -0.059 | 0.155 | 0.383 | 0.461 | -0.039 | -0.153 | 0.074 | 0.497 | 0.735 |
| M-VLDL | 0.002 | -0.105 | 0.109 | 0.970 | 0.982 | -0.060 | -0.174 | 0.054 | 0.300 | 0.546 |
| L-VLDL | 0.141 | 0.031 | 0.251 | 0.012 | 0.023 | 0.008 | -0.109 | 0.125 | 0.891 | 0.940 |
| VL-VLDL | 0.199 | 0.089 | 0.310 | <0.001 | 0.001 | 0.054 | -0.064 | 0.173 | 0.367 | 0.618 |
| XL-VLDL | 0.244 | 0.134 | 0.353 | <0.001 | <0.001 | 0.105 | -0.011 | 0.221 | 0.076 | 0.207 |
| VLDL | 0.121 | 0.013 | 0.230 | 0.028 | 0.047 | 0.011 | -0.105 | 0.126 | 0.858 | 0.917 |
| S-LDL | -0.044 | -0.151 | 0.062 | 0.416 | 0.488 | -0.045 | -0.159 | 0.069 | 0.442 | 0.696 |
| M-LDL | 0.008 | -0.098 | 0.114 | 0.881 | 0.910 | -0.006 | -0.119 | 0.107 | 0.922 | 0.952 |
| L-LDL | -0.057 | -0.163 | 0.050 | 0.299 | 0.376 | -0.015 | -0.129 | 0.098 | 0.791 | 0.889 |
| LDL | -0.038 | -0.144 | 0.068 | 0.486 | 0.553 | -0.016 | -0.129 | 0.097 | 0.783 | 0.889 |
| IDL | -0.073 | -0.183 | 0.036 | 0.187 | 0.258 | 0.002 | -0.117 | 0.120 | 0.976 | 0.984 |
| S-HDL | 0.356 | 0.252 | 0.459 | <0.001 | <0.001 | 0.241 | 0.133 | 0.348 | <0.001 | <0.001 |
| M-HDL | 0.260 | 0.148 | 0.372 | <0.001 | <0.001 | 0.268 | 0.152 | 0.383 | <0.001 | <0.001 |
| L-HDL | -0.091 | -0.209 | 0.026 | 0.127 | 0.186 | 0.090 | -0.037 | 0.217 | 0.166 | 0.354 |
| XL-HDL | -0.222 | -0.336 | -0.107 | <0.001 | <0.001 | -0.021 | -0.146 | 0.104 | 0.740 | 0.882 |
| HDL | 0.131 | 0.016 | 0.246 | 0.026 | 0.045 | 0.216 | 0.096 | 0.337 | <0.001 | 0.005 |
| Total | 0.079 | -0.027 | 0.186 | 0.143 | 0.206 | 0.073 | -0.039 | 0.186 | 0.202 | 0.405 |
| **Triglycerides** |  |  |  |  |  |  |  |  |  |  |
| VS-VLDL | 0.229 | 0.121 | 0.336 | <0.001 | <0.001 | 0.085 | -0.028 | 0.197 | 0.140 | 0.326 |
| S-VLDL | 0.171 | 0.062 | 0.280 | 0.002 | 0.005 | 0.027 | -0.087 | 0.142 | 0.643 | 0.829 |
| M-VLDL | 0.130 | 0.021 | 0.239 | 0.020 | 0.036 | 0.004 | -0.112 | 0.119 | 0.947 | 0.970 |
| L-VLDL | 0.169 | 0.059 | 0.279 | 0.003 | 0.006 | 0.032 | -0.085 | 0.149 | 0.593 | 0.801 |
| XL-VLDL | 0.242 | 0.132 | 0.353 | <0.001 | <0.001 | 0.086 | -0.032 | 0.205 | 0.153 | 0.335 |
| XXL-VLDL | 0.251 | 0.142 | 0.360 | <0.001 | <0.001 | 0.116 | <0.001 | 0.232 | 0.050 | 0.146 |
| VLDL | 0.205 | 0.096 | 0.315 | <0.001 | 0.001 | 0.060 | -0.057 | 0.177 | 0.312 | 0.563 |
| S-LDL | 0.238 | 0.128 | 0.347 | <0.001 | <0.001 | 0.095 | -0.020 | 0.211 | 0.105 | 0.262 |
| M-LDL | 0.276 | 0.167 | 0.385 | <0.001 | <0.001 | 0.128 | 0.014 | 0.243 | 0.028 | 0.101 |
| L-LDL | 0.285 | 0.177 | 0.393 | <0.001 | <0.001 | 0.147 | 0.034 | 0.260 | 0.011 | 0.059 |
| LDL | 0.280 | 0.172 | 0.388 | <0.001 | <0.001 | 0.138 | 0.024 | 0.251 | 0.018 | 0.073 |
| IDL | 0.251 | 0.144 | 0.358 | <0.001 | <0.001 | 0.121 | 0.009 | 0.232 | 0.034 | 0.115 |
| S-HDL | 0.304 | 0.195 | 0.413 | <0.001 | <0.001 | 0.121 | 0.004 | 0.238 | 0.042 | 0.130 |
| M-HDL | 0.293 | 0.187 | 0.398 | <0.001 | <0.001 | 0.153 | 0.043 | 0.262 | 0.006 | 0.040 |
| L-HDL | 0.119 | 0.012 | 0.227 | 0.030 | 0.050 | 0.097 | -0.012 | 0.206 | 0.080 | 0.213 |
| XL-HDL | 0.091 | -0.015 | 0.198 | 0.093 | 0.143 | 0.050 | -0.059 | 0.158 | 0.372 | 0.621 |
| HDL | 0.261 | 0.155 | 0.367 | <0.001 | <0.001 | 0.130 | 0.020 | 0.239 | 0.021 | 0.080 |
| Total | 0.226 | 0.116 | 0.335 | <0.001 | <0.001 | 0.078 | -0.038 | 0.194 | 0.187 | 0.389 |
| **Triglycerides to Total lipids** |  |  |  |  |  |  |  |  |  |  |
| VS-VLDL | 0.412 | 0.301 | 0.524 | <0.001 | <0.001 | 0.210 | 0.086 | 0.335 | 0.001 | 0.009 |
| S-VLDL | 0.292 | 0.184 | 0.401 | <0.001 | <0.001 | 0.142 | 0.026 | 0.258 | 0.017 | 0.073 |
| M-VLDL | 0.323 | 0.213 | 0.434 | <0.001 | <0.001 | 0.130 | 0.009 | 0.252 | 0.036 | 0.116 |
| L-VLDL | 0.080 | -0.021 | 0.182 | 0.121 | 0.179 | 0.077 | -0.027 | 0.181 | 0.145 | 0.328 |
| XL-VLDL | 0.253 | 0.146 | 0.360 | <0.001 | <0.001 | 0.108 | -0.004 | 0.220 | 0.058 | 0.162 |
| XXL-VLDL | 0.026 | -0.078 | 0.129 | 0.629 | 0.689 | 0.018 | -0.087 | 0.124 | 0.736 | 0.882 |
| S-LDL | 0.319 | 0.212 | 0.425 | <0.001 | <0.001 | 0.152 | 0.039 | 0.265 | 0.008 | 0.049 |
| M-LDL | 0.321 | 0.216 | 0.426 | <0.001 | <0.001 | 0.165 | 0.054 | 0.276 | 0.004 | 0.026 |
| L-LDL | 0.346 | 0.241 | 0.451 | <0.001 | <0.001 | 0.157 | 0.044 | 0.270 | 0.006 | 0.040 |
| IDL | 0.352 | 0.243 | 0.460 | <0.001 | <0.001 | 0.142 | 0.023 | 0.262 | 0.020 | 0.079 |
| S-HDL | 0.181 | 0.070 | 0.292 | 0.001 | 0.003 | 0.012 | -0.106 | 0.130 | 0.847 | 0.910 |
| M-HDL | 0.178 | 0.069 | 0.287 | 0.001 | 0.003 | 0.023 | -0.093 | 0.139 | 0.698 | 0.869 |
| L-HDL | 0.188 | 0.078 | 0.299 | 0.001 | 0.002 | 0.022 | -0.095 | 0.139 | 0.718 | 0.875 |
| XL-HDL | 0.308 | 0.196 | 0.419 | <0.001 | <0.001 | 0.121 | 0.002 | 0.239 | 0.046 | 0.137 |

† Model adjusted for age, sex, and education.
‡ Model adjusted for age, sex, education, race, socioeconomic status, body mass index, smoking status, alcohol drinking status, physical activity, social connection, hypertension, diabetes, heart disease, beta-blockers, calcium blockers, lipid-lowering, and APOE ε4 status.
Abbreviation: Apo, apolipoprotein; LDL-AD, average diameter for LDL particles; CHOL, cholesterol; CE, cholesteryl esters; TG, triglycerides; S, small; M, medium; L, large; VL, very large; XL, extremely large; VLDL, very low-density lipoprotein; LDL; low-density lipoprotein; IDL, intermediate-density lipoprotein; HDL, high-density lipoprotein; LA, linoleic acid; MUFA, monounsaturated fatty acid; n-6, omega-6 fatty acid; PUFA, polyunsaturated fatty acid; SFA, saturated fatty acid; FA, fatty acid.

**Table S10.** β coefficients and 95% confidence intervals (CIs) for the association between metabolites and brain age gap (BAG) among non-*APOE* ε4 carriers: results from linear regression models

| **Metabolites** | **Basic-model ^†^** | |  |  |  | **Multi-model ^‡^** | |  |  |  |
| --- | --- | --- | --- | --- | --- | --- | --- | --- | --- | --- |
|  | **β** | **Lower** | **Upper** | ***P*-value** | **FDR-q** | **β** | **Lower** | **Upper** | ***P*-value** | **FDR-q** |
| **Amino acids & Glycolysis** |  |  |  |  |  |  |  |  |  |  |
| Valine | 0.100 | 0.002 | 0.197 | 0.045 | 0.071 | -0.003 | -0.105 | 0.098 | 0.952 | 0.976 |
| Leucine | 0.100 | 0.002 | 0.198 | 0.046 | 0.072 | 0.028 | -0.073 | 0.128 | 0.590 | 0.799 |
| Isoleucine | 0.048 | -0.048 | 0.144 | 0.330 | 0.393 | -0.014 | -0.112 | 0.084 | 0.774 | 0.889 |
| Phenylalanine | 0.091 | -0.005 | 0.187 | 0.065 | 0.097 | 0.043 | -0.055 | 0.140 | 0.392 | 0.614 |
| Tyrosine | 0.191 | 0.096 | 0.285 | <0.001 | <0.001 | 0.137 | 0.040 | 0.234 | 0.006 | 0.034 |
| Alanine | 0.071 | -0.025 | 0.166 | 0.146 | 0.194 | 0.011 | -0.086 | 0.108 | 0.818 | 0.922 |
| Glutamine | -0.342 | -0.438 | -0.247 | <0.001 | <0.001 | -0.236 | -0.333 | -0.138 | <0.001 | <0.001 |
| Glycine | -0.069 | -0.171 | 0.033 | 0.185 | 0.243 | 0.019 | -0.085 | 0.124 | 0.721 | 0.857 |
| Histidine | -0.048 | -0.142 | 0.047 | 0.323 | 0.386 | -0.017 | -0.112 | 0.079 | 0.732 | 0.859 |
| BCAAs | 0.093 | -0.005 | 0.190 | 0.063 | 0.095 | 0.005 | -0.096 | 0.105 | 0.929 | 0.972 |
| Lactate | 0.208 | 0.112 | 0.304 | <0.001 | <0.001 | 0.135 | 0.038 | 0.233 | 0.007 | 0.038 |
| Pyruvate | 0.137 | 0.041 | 0.232 | 0.005 | 0.010 | 0.082 | -0.015 | 0.178 | 0.096 | 0.242 |
| Glucose | 0.278 | 0.185 | 0.371 | <0.001 | <0.001 | 0.093 | -0.008 | 0.194 | 0.070 | 0.198 |
| Citrate | -0.044 | -0.141 | 0.053 | 0.374 | 0.435 | -0.005 | -0.103 | 0.093 | 0.917 | 0.968 |
| **Apo-LP, LP size & Other lipids** | |  |  |  |  |  |  |  |  |  |
| ApoA1 | 0.171 | 0.068 | 0.275 | 0.001 | 0.003 | 0.235 | 0.127 | 0.342 | <0.001 | <0.001 |
| ApoB | -0.082 | -0.180 | 0.015 | 0.099 | 0.139 | -0.070 | -0.175 | 0.035 | 0.189 | 0.384 |
| ApoB/ApoA1 | -0.159 | -0.259 | -0.059 | 0.002 | 0.004 | -0.187 | -0.295 | -0.079 | 0.001 | 0.006 |
| HDL-AD | -0.191 | -0.298 | -0.083 | 0.001 | 0.001 | 0.043 | -0.074 | 0.160 | 0.475 | 0.704 |
| LDL-AD | -0.166 | -0.264 | -0.067 | 0.001 | 0.002 | -0.070 | -0.171 | 0.032 | 0.179 | 0.375 |
| VLDL-AD | 0.215 | 0.112 | 0.318 | <0.001 | <0.001 | 0.032 | -0.079 | 0.142 | 0.574 | 0.786 |
| Phosphatidylcholine | 0.137 | 0.036 | 0.238 | 0.008 | 0.014 | 0.157 | 0.053 | 0.261 | 0.003 | 0.021 |
| Phosphoglycerides | 0.181 | 0.081 | 0.281 | <0.001 | 0.001 | 0.170 | 0.067 | 0.273 | 0.001 | 0.010 |
| Sphingomyeline | 0.005 | -0.097 | 0.107 | 0.928 | 0.939 | 0.087 | -0.020 | 0.194 | 0.112 | 0.261 |
| Total choline | 0.127 | 0.026 | 0.228 | 0.014 | 0.024 | 0.149 | 0.045 | 0.254 | 0.005 | 0.033 |
| TG/Phosphoglycerides | 0.198 | 0.093 | 0.303 | <0.001 | 0.001 | -0.008 | -0.121 | 0.106 | 0.896 | 0.968 |
| **Cholesterol** |  |  |  |  |  |  |  |  |  |  |
| XS-VLDL | -0.117 | -0.216 | -0.017 | 0.021 | 0.035 | -0.066 | -0.173 | 0.041 | 0.224 | 0.427 |
| S-VLDL | -0.050 | -0.147 | 0.048 | 0.318 | 0.384 | -0.091 | -0.194 | 0.012 | 0.084 | 0.217 |
| M-VLDL | -0.169 | -0.266 | -0.072 | 0.001 | 0.001 | -0.128 | -0.232 | -0.023 | 0.016 | 0.077 |
| L-VLDL | 0.081 | -0.018 | 0.181 | 0.109 | 0.152 | -0.047 | -0.153 | 0.060 | 0.390 | 0.614 |
| VL-VLDL | 0.110 | 0.010 | 0.209 | 0.031 | 0.051 | -0.022 | -0.129 | 0.085 | 0.682 | 0.844 |
| XL-VLDL | 0.226 | 0.125 | 0.326 | <0.001 | <0.001 | 0.064 | -0.043 | 0.170 | 0.240 | 0.446 |
| VLDL | -0.014 | -0.112 | 0.083 | 0.771 | 0.813 | -0.067 | -0.171 | 0.037 | 0.209 | 0.410 |
| S-LDL | -0.045 | -0.143 | 0.052 | 0.363 | 0.428 | -0.037 | -0.141 | 0.067 | 0.489 | 0.713 |
| M-LDL | <0.001 | -0.097 | 0.097 | 0.995 | 0.995 | -0.007 | -0.110 | 0.096 | 0.889 | 0.968 |
| L-LDL | -0.082 | -0.180 | 0.016 | 0.099 | 0.139 | -0.022 | -0.126 | 0.082 | 0.685 | 0.844 |
| Clinical LDL | -0.086 | -0.183 | 0.012 | 0.085 | 0.123 | -0.033 | -0.137 | 0.072 | 0.538 | 0.756 |
| LDL | -0.057 | -0.155 | 0.040 | 0.250 | 0.309 | -0.019 | -0.123 | 0.084 | 0.713 | 0.857 |
| IDL | -0.107 | -0.208 | -0.007 | 0.036 | 0.057 | -0.004 | -0.112 | 0.105 | 0.947 | 0.976 |
| S-HDL | 0.279 | 0.184 | 0.373 | <0.001 | <0.001 | 0.201 | 0.104 | 0.297 | <0.001 | 0.001 |
| M-HDL | 0.174 | 0.071 | 0.278 | 0.001 | 0.002 | 0.248 | 0.140 | 0.355 | <0.001 | <0.001 |
| L-HDL | -0.175 | -0.282 | -0.068 | 0.001 | 0.003 | 0.062 | -0.055 | 0.179 | 0.298 | 0.512 |
| XL-HDL | -0.279 | -0.384 | -0.174 | <0.001 | <0.001 | -0.029 | -0.144 | 0.086 | 0.617 | 0.809 |
| HDL | 0.015 | -0.091 | 0.121 | 0.785 | 0.825 | 0.180 | 0.067 | 0.293 | 0.002 | 0.013 |
| Total | -0.051 | -0.150 | 0.048 | 0.311 | 0.378 | 0.027 | -0.078 | 0.133 | 0.614 | 0.808 |
| Remnant | -0.061 | -0.158 | 0.037 | 0.224 | 0.284 | -0.042 | -0.148 | 0.063 | 0.430 | 0.661 |
| Total-HDLC | -0.060 | -0.157 | 0.037 | 0.228 | 0.286 | -0.031 | -0.136 | 0.074 | 0.560 | 0.780 |
| **Cholesterol to Total lipids** |  |  |  |  |  |  |  |  |  |  |
| VS-VLDL | -0.380 | -0.482 | -0.278 | <0.001 | <0.001 | -0.138 | -0.251 | -0.024 | 0.017 | 0.079 |
| S-VLDL | -0.288 | -0.387 | -0.190 | <0.001 | <0.001 | -0.134 | -0.239 | -0.030 | 0.012 | 0.063 |
| M-VLDL | -0.357 | -0.459 | -0.255 | <0.001 | <0.001 | -0.131 | -0.242 | -0.019 | 0.021 | 0.090 |
| L-VLDL | -0.232 | -0.329 | -0.135 | <0.001 | <0.001 | -0.122 | -0.222 | -0.022 | 0.016 | 0.077 |
| XL-VLDL | -0.254 | -0.353 | -0.155 | <0.001 | <0.001 | -0.072 | -0.176 | 0.032 | 0.174 | 0.372 |
| XXL-VLDL | -0.073 | -0.170 | 0.023 | 0.138 | 0.184 | -0.020 | -0.118 | 0.079 | 0.696 | 0.850 |
| S-LDL | -0.114 | -0.208 | -0.020 | 0.017 | 0.030 | -0.031 | -0.129 | 0.067 | 0.532 | 0.753 |
| M-LDL | -0.241 | -0.335 | -0.147 | <0.001 | <0.001 | -0.095 | -0.195 | 0.004 | 0.061 | 0.189 |
| L-LDL | -0.315 | -0.410 | -0.221 | <0.001 | <0.001 | -0.106 | -0.208 | -0.004 | 0.041 | 0.146 |
| IDL | -0.223 | -0.321 | -0.124 | <0.001 | <0.001 | -0.023 | -0.129 | 0.083 | 0.669 | 0.843 |
| S-HDL | -0.256 | -0.353 | -0.159 | <0.001 | <0.001 | -0.099 | -0.198 | 0.001 | 0.053 | 0.171 |
| M-HDL | -0.213 | -0.314 | -0.113 | <0.001 | <0.001 | -0.002 | -0.110 | 0.106 | 0.977 | 0.984 |
| L-HDL | -0.399 | -0.500 | -0.298 | <0.001 | <0.001 | -0.128 | -0.240 | -0.015 | 0.026 | 0.105 |
| XL-HDL | 0.091 | -0.010 | 0.192 | 0.078 | 0.114 | 0.019 | -0.085 | 0.123 | 0.719 | 0.857 |
| **Cholesteryl esters to Total lipids** | |  |  |  |  |  |  |  |  |  |
| VS-VLDL | -0.371 | -0.473 | -0.268 | <0.001 | <0.001 | -0.119 | -0.233 | -0.004 | 0.042 | 0.146 |
| S-VLDL | -0.241 | -0.337 | -0.144 | <0.001 | <0.001 | -0.138 | -0.239 | -0.037 | 0.007 | 0.041 |
| M-VLDL | -0.359 | -0.462 | -0.257 | <0.001 | <0.001 | -0.128 | -0.239 | -0.016 | 0.025 | 0.102 |
| L-VLDL | -0.320 | -0.420 | -0.220 | <0.001 | <0.001 | -0.149 | -0.254 | -0.044 | 0.006 | 0.034 |
| XL-VLDL | -0.232 | -0.330 | -0.134 | <0.001 | <0.001 | -0.059 | -0.161 | 0.044 | 0.265 | 0.471 |
| XXL-VLDL | -0.078 | -0.175 | 0.019 | 0.115 | 0.156 | -0.024 | -0.122 | 0.074 | 0.637 | 0.822 |
| S-LDL | 0.128 | 0.034 | 0.223 | 0.008 | 0.014 | 0.049 | -0.049 | 0.147 | 0.327 | 0.557 |
| M-LDL | 0.095 | -0.001 | 0.191 | 0.053 | 0.080 | 0.016 | -0.084 | 0.116 | 0.758 | 0.874 |
| L-LDL | -0.124 | -0.216 | -0.031 | 0.009 | 0.016 | -0.059 | -0.152 | 0.035 | 0.219 | 0.423 |
| IDL | -0.126 | -0.223 | -0.028 | 0.012 | 0.020 | 0.037 | -0.066 | 0.140 | 0.482 | 0.711 |
| S-HDL | -0.193 | -0.290 | -0.096 | <0.001 | <0.001 | -0.081 | -0.180 | 0.018 | 0.108 | 0.261 |
| M-HDL | -0.214 | -0.313 | -0.115 | <0.001 | <0.001 | -0.026 | -0.131 | 0.079 | 0.628 | 0.819 |
| L-HDL | -0.400 | -0.500 | -0.299 | <0.001 | <0.001 | -0.139 | -0.250 | -0.029 | 0.014 | 0.066 |
| XL-HDL | -0.033 | -0.129 | 0.063 | 0.495 | 0.550 | 0.047 | -0.051 | 0.144 | 0.349 | 0.581 |
| **Cholestryl esters** |  |  |  |  |  |  |  |  |  |  |
| VS-VLDL | -0.142 | -0.242 | -0.042 | 0.005 | 0.010 | -0.068 | -0.176 | 0.040 | 0.216 | 0.420 |
| S-VLDL | -0.032 | -0.130 | 0.065 | 0.515 | 0.569 | -0.090 | -0.193 | 0.014 | 0.089 | 0.227 |
| M-VLDL | -0.229 | -0.327 | -0.132 | <0.001 | <0.001 | -0.147 | -0.253 | -0.041 | 0.006 | 0.038 |
| L-VLDL | 0.011 | -0.087 | 0.109 | 0.823 | 0.854 | -0.085 | -0.189 | 0.020 | 0.112 | 0.261 |
| VL-VLDL | 0.060 | -0.039 | 0.159 | 0.236 | 0.295 | -0.049 | -0.156 | 0.057 | 0.364 | 0.597 |
| XL-VLDL | 0.212 | 0.112 | 0.312 | <0.001 | <0.001 | 0.058 | -0.049 | 0.165 | 0.286 | 0.497 |
| VLDL | -0.060 | -0.157 | 0.037 | 0.224 | 0.284 | -0.085 | -0.189 | 0.020 | 0.111 | 0.261 |
| S-LDL | -0.006 | -0.104 | 0.091 | 0.898 | 0.916 | -0.022 | -0.126 | 0.082 | 0.677 | 0.843 |
| M-LDL | 0.031 | -0.066 | 0.128 | 0.533 | 0.588 | 0.003 | -0.100 | 0.107 | 0.951 | 0.976 |
| L-LDL | -0.066 | -0.164 | 0.032 | 0.185 | 0.243 | -0.018 | -0.122 | 0.085 | 0.727 | 0.857 |
| LDL | -0.034 | -0.132 | 0.063 | 0.488 | 0.548 | -0.013 | -0.116 | 0.090 | 0.802 | 0.912 |
| IDL | -0.094 | -0.194 | 0.006 | 0.066 | 0.099 | 0.007 | -0.102 | 0.115 | 0.902 | 0.968 |
| S-HDL | 0.267 | 0.173 | 0.362 | <0.001 | <0.001 | 0.190 | 0.094 | 0.287 | <0.001 | 0.001 |
| M-HDL | 0.173 | 0.070 | 0.277 | 0.001 | 0.002 | 0.247 | 0.140 | 0.355 | <0.001 | <0.001 |
| L-HDL | -0.182 | -0.289 | -0.076 | 0.001 | 0.002 | 0.057 | -0.060 | 0.175 | 0.338 | 0.572 |
| XL-HDL | -0.272 | -0.377 | -0.166 | <0.001 | <0.001 | -0.020 | -0.135 | 0.096 | 0.738 | 0.859 |
| HDL | 0.010 | -0.096 | 0.116 | 0.853 | 0.880 | 0.178 | 0.065 | 0.291 | 0.002 | 0.014 |
| Total | -0.046 | -0.145 | 0.053 | 0.365 | 0.428 | 0.040 | -0.066 | 0.145 | 0.462 | 0.694 |
| **Fatty acids** |  |  |  |  |  |  |  |  |  |  |
| DHA | -0.097 | -0.196 | 0.003 | 0.056 | 0.085 | -0.039 | -0.140 | 0.062 | 0.451 | 0.680 |
| LA | -0.087 | -0.183 | 0.010 | 0.078 | 0.114 | -0.047 | -0.148 | 0.054 | 0.361 | 0.595 |
| MUFA | 0.303 | 0.205 | 0.401 | <0.001 | <0.001 | 0.138 | 0.035 | 0.242 | 0.009 | 0.049 |
| n-3 | 0.008 | -0.090 | 0.106 | 0.873 | 0.894 | -0.006 | -0.105 | 0.094 | 0.910 | 0.968 |
| n-6 | -0.002 | -0.099 | 0.095 | 0.964 | 0.972 | 0.003 | -0.098 | 0.104 | 0.951 | 0.976 |
| PUFA | <0.001 | -0.097 | 0.098 | 0.995 | 0.995 | 0.001 | -0.100 | 0.102 | 0.984 | 0.984 |
| SFA | 0.298 | 0.200 | 0.395 | <0.001 | <0.001 | 0.176 | 0.074 | 0.278 | 0.001 | 0.006 |
| Total | 0.218 | 0.121 | 0.315 | <0.001 | <0.001 | 0.114 | 0.013 | 0.215 | 0.027 | 0.106 |
| Unsaturation | -0.336 | -0.436 | -0.236 | <0.001 | <0.001 | -0.182 | -0.287 | -0.078 | 0.001 | 0.006 |
| DHA/FA | -0.218 | -0.317 | -0.120 | <0.001 | <0.001 | -0.106 | -0.209 | -0.004 | 0.042 | 0.146 |
| LA/FA | -0.520 | -0.618 | -0.421 | <0.001 | <0.001 | -0.302 | -0.411 | -0.194 | <0.001 | <0.001 |
| MUFA/FA | 0.391 | 0.290 | 0.492 | <0.001 | <0.001 | 0.157 | 0.046 | 0.267 | 0.005 | 0.034 |
| n-3/FA | -0.102 | -0.200 | -0.004 | 0.041 | 0.065 | -0.059 | -0.159 | 0.040 | 0.242 | 0.447 |
| n-6/n-3 | 0.042 | -0.055 | 0.138 | 0.398 | 0.458 | 0.067 | -0.031 | 0.165 | 0.178 | 0.375 |
| n-6/FA | -0.469 | -0.568 | -0.369 | <0.001 | <0.001 | -0.261 | -0.368 | -0.154 | <0.001 | <0.001 |
| PUFA/MUFA | -0.433 | -0.533 | -0.333 | <0.001 | <0.001 | -0.205 | -0.314 | -0.095 | <0.001 | 0.003 |
| PUFA/FA | -0.500 | -0.600 | -0.401 | <0.001 | <0.001 | -0.289 | -0.397 | -0.181 | <0.001 | <0.001 |
| SFA/FA | 0.403 | 0.307 | 0.499 | <0.001 | <0.001 | 0.286 | 0.187 | 0.386 | <0.001 | <0.001 |
| **Free cholesterol** |  |  |  |  |  |  |  |  |  |  |
| VS-VLDL | -0.055 | -0.154 | 0.043 | 0.271 | 0.332 | -0.059 | -0.164 | 0.045 | 0.264 | 0.471 |
| S-VLDL | -0.079 | -0.176 | 0.018 | 0.112 | 0.154 | -0.092 | -0.195 | 0.011 | 0.081 | 0.212 |
| M-VLDL | -0.084 | -0.181 | 0.013 | 0.089 | 0.127 | -0.097 | -0.201 | 0.007 | 0.067 | 0.197 |
| L-VLDL | 0.149 | 0.048 | 0.249 | 0.004 | 0.007 | -0.007 | -0.115 | 0.100 | 0.894 | 0.968 |
| VL-VLDL | 0.158 | 0.058 | 0.259 | 0.002 | 0.004 | 0.007 | -0.100 | 0.114 | 0.902 | 0.968 |
| XL-VLDL | 0.238 | 0.138 | 0.338 | <0.001 | <0.001 | 0.069 | -0.036 | 0.175 | 0.199 | 0.392 |
| VLDL | 0.048 | -0.050 | 0.146 | 0.337 | 0.400 | -0.041 | -0.146 | 0.064 | 0.443 | 0.672 |
| S-LDL | -0.143 | -0.241 | -0.046 | 0.004 | 0.007 | -0.070 | -0.174 | 0.033 | 0.181 | 0.375 |
| M-LDL | -0.086 | -0.183 | 0.012 | 0.085 | 0.123 | -0.036 | -0.138 | 0.067 | 0.497 | 0.714 |
| L-LDL | -0.126 | -0.224 | -0.027 | 0.012 | 0.022 | -0.030 | -0.135 | 0.076 | 0.581 | 0.791 |
| LDL | -0.118 | -0.216 | -0.020 | 0.019 | 0.031 | -0.036 | -0.140 | 0.068 | 0.499 | 0.714 |
| IDL | -0.143 | -0.243 | -0.043 | 0.005 | 0.010 | -0.033 | -0.142 | 0.075 | 0.547 | 0.765 |
| S-HDL | 0.280 | 0.183 | 0.376 | <0.001 | <0.001 | 0.207 | 0.108 | 0.305 | <0.001 | 0.001 |
| M-HDL | 0.173 | 0.069 | 0.277 | 0.001 | 0.002 | 0.241 | 0.133 | 0.349 | <0.001 | <0.001 |
| L-HDL | -0.146 | -0.253 | -0.038 | 0.008 | 0.014 | 0.078 | -0.038 | 0.194 | 0.190 | 0.384 |
| XL-HDL | -0.289 | -0.392 | -0.187 | <0.001 | <0.001 | -0.061 | -0.172 | 0.049 | 0.278 | 0.487 |
| HDL | 0.031 | -0.076 | 0.137 | 0.569 | 0.621 | 0.179 | 0.067 | 0.291 | 0.002 | 0.013 |
| Total | -0.063 | -0.162 | 0.035 | 0.208 | 0.269 | -0.004 | -0.110 | 0.101 | 0.935 | 0.974 |
| **Free cholesterol to Total lipids** |  |  |  |  |  |  |  |  |  |  |
| VS-VLDL | -0.334 | -0.431 | -0.237 | <0.001 | <0.001 | -0.200 | -0.302 | -0.097 | <0.001 | 0.001 |
| S-VLDL | -0.305 | -0.405 | -0.205 | <0.001 | <0.001 | -0.103 | -0.211 | 0.006 | 0.063 | 0.194 |
| M-VLDL | -0.333 | -0.434 | -0.232 | <0.001 | <0.001 | -0.132 | -0.242 | -0.023 | 0.018 | 0.081 |
| L-VLDL | 0.017 | -0.077 | 0.111 | 0.719 | 0.768 | -0.044 | -0.140 | 0.052 | 0.371 | 0.604 |
| XL-VLDL | -0.253 | -0.351 | -0.156 | <0.001 | <0.001 | -0.094 | -0.196 | 0.007 | 0.068 | 0.197 |
| XXL-VLDL | -0.044 | -0.140 | 0.053 | 0.374 | 0.435 | -0.006 | -0.104 | 0.093 | 0.913 | 0.968 |
| S-LDL | -0.274 | -0.371 | -0.176 | <0.001 | <0.001 | -0.091 | -0.193 | 0.010 | 0.078 | 0.208 |
| M-LDL | -0.325 | -0.425 | -0.225 | <0.001 | <0.001 | -0.106 | -0.213 | 0.001 | 0.051 | 0.170 |
| L-LDL | -0.330 | -0.429 | -0.230 | <0.001 | <0.001 | -0.085 | -0.194 | 0.024 | 0.125 | 0.286 |
| IDL | -0.292 | -0.389 | -0.195 | <0.001 | <0.001 | -0.128 | -0.229 | -0.027 | 0.013 | 0.064 |
| S-HDL | -0.237 | -0.337 | -0.138 | <0.001 | <0.001 | -0.061 | -0.165 | 0.044 | 0.256 | 0.466 |
| M-HDL | -0.098 | -0.204 | 0.009 | 0.072 | 0.107 | 0.106 | -0.008 | 0.220 | 0.067 | 0.197 |
| L-HDL | -0.211 | -0.312 | -0.110 | <0.001 | <0.001 | -0.010 | -0.119 | 0.098 | 0.851 | 0.945 |
| XL-HDL | 0.176 | 0.070 | 0.281 | 0.001 | 0.002 | -0.026 | -0.139 | 0.087 | 0.652 | 0.832 |
| **KB, FB & Inflammation** |  |  |  |  |  |  |  |  |  |  |
| Glycoprotein acetyls | 0.318 | 0.222 | 0.414 | <0.001 | <0.001 | 0.111 | 0.007 | 0.214 | 0.036 | 0.131 |
| 3-Hydrocybutyrate | 0.153 | 0.059 | 0.247 | 0.001 | 0.003 | 0.150 | 0.054 | 0.246 | 0.002 | 0.015 |
| Acetate | -0.113 | -0.198 | -0.027 | 0.010 | 0.017 | -0.083 | -0.168 | 0.003 | 0.058 | 0.183 |
| Acetoacetate | 0.164 | 0.069 | 0.259 | 0.001 | 0.002 | 0.123 | 0.027 | 0.218 | 0.012 | 0.063 |
| Acetone | 0.099 | 0.004 | 0.195 | 0.041 | 0.065 | 0.111 | 0.014 | 0.207 | 0.024 | 0.100 |
| Albumin | 0.026 | -0.070 | 0.122 | 0.598 | 0.651 | 0.021 | -0.077 | 0.119 | 0.675 | 0.843 |
| Creatinine | -0.030 | -0.146 | 0.087 | 0.617 | 0.666 | -0.118 | -0.237 | 0.002 | 0.053 | 0.171 |
| **Lipoprotein particles** |  |  |  |  |  |  |  |  |  |  |
| VS-VLDL | -0.025 | -0.123 | 0.073 | 0.618 | 0.666 | -0.042 | -0.146 | 0.062 | 0.427 | 0.660 |
| S-VLDL | 0.050 | -0.049 | 0.148 | 0.321 | 0.386 | -0.057 | -0.161 | 0.046 | 0.278 | 0.487 |
| M-VLDL | -0.029 | -0.127 | 0.068 | 0.555 | 0.609 | -0.076 | -0.180 | 0.028 | 0.152 | 0.339 |
| L-VLDL | 0.166 | 0.065 | 0.267 | 0.001 | 0.003 | 0.009 | -0.099 | 0.116 | 0.873 | 0.966 |
| VL-VLDL | 0.213 | 0.112 | 0.314 | <0.001 | <0.001 | 0.039 | -0.069 | 0.148 | 0.475 | 0.704 |
| XL-VLDL | 0.256 | 0.156 | 0.356 | <0.001 | <0.001 | 0.084 | -0.023 | 0.190 | 0.123 | 0.283 |
| VLDL | 0.037 | -0.061 | 0.134 | 0.464 | 0.530 | -0.045 | -0.149 | 0.059 | 0.392 | 0.614 |
| S-LDL | -0.079 | -0.177 | 0.019 | 0.114 | 0.155 | -0.078 | -0.183 | 0.026 | 0.143 | 0.320 |
| M-LDL | -0.041 | -0.138 | 0.057 | 0.411 | 0.472 | -0.046 | -0.150 | 0.058 | 0.389 | 0.614 |
| L-LDL | -0.122 | -0.220 | -0.025 | 0.014 | 0.024 | -0.093 | -0.198 | 0.011 | 0.079 | 0.208 |
| LDL | -0.098 | -0.195 | <0.001 | 0.049 | 0.077 | -0.081 | -0.186 | 0.023 | 0.126 | 0.286 |
| IDL | -0.080 | -0.178 | 0.018 | 0.110 | 0.153 | -0.028 | -0.135 | 0.079 | 0.606 | 0.807 |
| S-HDL | 0.289 | 0.194 | 0.383 | <0.001 | <0.001 | 0.194 | 0.097 | 0.291 | <0.001 | 0.001 |
| M-HDL | 0.204 | 0.101 | 0.308 | <0.001 | <0.001 | 0.251 | 0.144 | 0.358 | <0.001 | <0.001 |
| L-HDL | -0.153 | -0.260 | -0.046 | 0.005 | 0.010 | 0.073 | -0.044 | 0.190 | 0.221 | 0.423 |
| XL-HDL | -0.255 | -0.360 | -0.150 | <0.001 | <0.001 | -0.022 | -0.136 | 0.092 | 0.706 | 0.857 |
| HDL | 0.199 | 0.098 | 0.299 | <0.001 | <0.001 | 0.227 | 0.124 | 0.331 | <0.001 | <0.001 |
| Total | 0.176 | 0.076 | 0.276 | 0.001 | 0.001 | 0.207 | 0.104 | 0.309 | <0.001 | 0.001 |
| **Phospholipids** |  |  |  |  |  |  |  |  |  |  |
| VS-VLDL | -0.005 | -0.103 | 0.093 | 0.924 | 0.939 | -0.045 | -0.148 | 0.058 | 0.390 | 0.614 |
| S-VLDL | -0.019 | -0.117 | 0.078 | 0.695 | 0.746 | -0.074 | -0.177 | 0.029 | 0.159 | 0.346 |
| M-VLDL | -0.036 | -0.133 | 0.062 | 0.473 | 0.538 | -0.074 | -0.178 | 0.029 | 0.160 | 0.346 |
| L-VLDL | 0.171 | 0.070 | 0.271 | 0.001 | 0.002 | 0.006 | -0.102 | 0.114 | 0.913 | 0.968 |
| VL-VLDL | 0.184 | 0.084 | 0.285 | <0.001 | 0.001 | 0.023 | -0.085 | 0.131 | 0.677 | 0.843 |
| XL-VLDL | 0.252 | 0.152 | 0.352 | <0.001 | <0.001 | 0.077 | -0.029 | 0.183 | 0.155 | 0.341 |
| VLDL | 0.089 | -0.010 | 0.187 | 0.077 | 0.114 | -0.023 | -0.128 | 0.082 | 0.672 | 0.843 |
| S-LDL | -0.083 | -0.181 | 0.016 | 0.099 | 0.139 | -0.062 | -0.166 | 0.042 | 0.245 | 0.449 |
| M-LDL | 0.009 | -0.088 | 0.107 | 0.855 | 0.880 | -0.007 | -0.110 | 0.097 | 0.899 | 0.968 |
| L-LDL | -0.059 | -0.156 | 0.039 | 0.239 | 0.298 | -0.013 | -0.117 | 0.092 | 0.813 | 0.920 |
| LDL | -0.042 | -0.140 | 0.055 | 0.396 | 0.458 | -0.017 | -0.121 | 0.086 | 0.741 | 0.859 |
| IDL | -0.121 | -0.221 | -0.020 | 0.018 | 0.031 | -0.037 | -0.145 | 0.071 | 0.504 | 0.717 |
| S-HDL | 0.401 | 0.306 | 0.497 | <0.001 | <0.001 | 0.264 | 0.165 | 0.362 | <0.001 | <0.001 |
| M-HDL | 0.299 | 0.197 | 0.401 | <0.001 | <0.001 | 0.281 | 0.176 | 0.385 | <0.001 | <0.001 |
| L-HDL | -0.067 | -0.174 | 0.041 | 0.225 | 0.284 | 0.126 | 0.011 | 0.242 | 0.032 | 0.119 |
| XL-HDL | -0.247 | -0.353 | -0.142 | <0.001 | <0.001 | -0.016 | -0.130 | 0.099 | 0.787 | 0.899 |
| HDL | 0.174 | 0.069 | 0.280 | 0.001 | 0.003 | 0.242 | 0.133 | 0.351 | <0.001 | <0.001 |
| Total | 0.113 | 0.013 | 0.213 | 0.027 | 0.044 | 0.132 | 0.028 | 0.237 | 0.013 | 0.064 |
| **Phospholipids to Total lipids** |  |  |  |  |  |  |  |  |  |  |
| VS-VLDL | 0.064 | -0.033 | 0.160 | 0.197 | 0.255 | -0.091 | -0.191 | 0.009 | 0.074 | 0.203 |
| S-VLDL | -0.293 | -0.394 | -0.193 | <0.001 | <0.001 | -0.098 | -0.206 | 0.010 | 0.075 | 0.203 |
| M-VLDL | -0.267 | -0.366 | -0.168 | <0.001 | <0.001 | -0.094 | -0.202 | 0.014 | 0.088 | 0.226 |
| L-VLDL | 0.115 | 0.020 | 0.211 | 0.018 | 0.031 | -0.026 | -0.126 | 0.074 | 0.604 | 0.807 |
| VL-VLDL | -0.060 | -0.152 | 0.031 | 0.197 | 0.255 | -0.086 | -0.181 | 0.008 | 0.073 | 0.203 |
| XL-VLDL | 0.103 | 0.008 | 0.198 | 0.033 | 0.054 | -0.020 | -0.117 | 0.078 | 0.693 | 0.850 |
| S-LDL | -0.146 | -0.240 | -0.052 | 0.002 | 0.004 | -0.064 | -0.161 | 0.033 | 0.193 | 0.385 |
| M-LDL | -0.065 | -0.160 | 0.031 | 0.185 | 0.243 | -0.041 | -0.138 | 0.056 | 0.410 | 0.638 |
| L-LDL | -0.011 | -0.105 | 0.083 | 0.815 | 0.849 | -0.025 | -0.121 | 0.070 | 0.600 | 0.807 |
| IDL | -0.151 | -0.244 | -0.057 | 0.002 | 0.003 | -0.153 | -0.248 | -0.058 | 0.002 | 0.012 |
| S-HDL | 0.231 | 0.130 | 0.332 | <0.001 | <0.001 | 0.176 | 0.074 | 0.278 | 0.001 | 0.006 |
| M-HDL | 0.259 | 0.159 | 0.360 | <0.001 | <0.001 | 0.032 | -0.078 | 0.141 | 0.570 | 0.786 |
| L-HDL | 0.539 | 0.440 | 0.639 | <0.001 | <0.001 | 0.273 | 0.159 | 0.386 | <0.001 | <0.001 |
| XL-HDL | -0.230 | -0.333 | -0.127 | <0.001 | <0.001 | -0.065 | -0.173 | 0.043 | 0.237 | 0.446 |
| **Total lipids** |  |  |  |  |  |  |  |  |  |  |
| VS-VLDL | -0.013 | -0.112 | 0.085 | 0.790 | 0.826 | -0.036 | -0.140 | 0.068 | 0.496 | 0.714 |
| S-VLDL | 0.052 | -0.046 | 0.150 | 0.301 | 0.368 | -0.050 | -0.154 | 0.053 | 0.342 | 0.576 |
| M-VLDL | 0.017 | -0.081 | 0.115 | 0.730 | 0.773 | -0.055 | -0.159 | 0.048 | 0.296 | 0.512 |
| L-VLDL | 0.158 | 0.058 | 0.259 | 0.002 | 0.004 | 0.005 | -0.102 | 0.112 | 0.928 | 0.972 |
| VL-VLDL | 0.213 | 0.112 | 0.314 | <0.001 | <0.001 | 0.043 | -0.065 | 0.151 | 0.433 | 0.662 |
| XL-VLDL | 0.254 | 0.154 | 0.354 | <0.001 | <0.001 | 0.089 | -0.017 | 0.195 | 0.100 | 0.247 |
| VLDL | 0.132 | 0.033 | 0.231 | 0.009 | 0.016 | 0.002 | -0.104 | 0.108 | 0.971 | 0.983 |
| S-LDL | -0.035 | -0.133 | 0.063 | 0.479 | 0.543 | -0.037 | -0.141 | 0.068 | 0.490 | 0.713 |
| M-LDL | 0.018 | -0.080 | 0.115 | 0.724 | 0.771 | -0.001 | -0.104 | 0.102 | 0.984 | 0.984 |
| L-LDL | -0.057 | -0.154 | 0.041 | 0.256 | 0.316 | -0.011 | -0.115 | 0.093 | 0.837 | 0.934 |
| LDL | -0.034 | -0.132 | 0.063 | 0.491 | 0.548 | -0.011 | -0.114 | 0.092 | 0.834 | 0.934 |
| IDL | -0.085 | -0.185 | 0.015 | 0.098 | 0.139 | -0.002 | -0.110 | 0.106 | 0.968 | 0.983 |
| S-HDL | 0.373 | 0.278 | 0.468 | <0.001 | <0.001 | 0.242 | 0.144 | 0.340 | <0.001 | <0.001 |
| M-HDL | 0.255 | 0.152 | 0.358 | <0.001 | <0.001 | 0.269 | 0.163 | 0.374 | <0.001 | <0.001 |
| L-HDL | -0.119 | -0.227 | -0.012 | 0.030 | 0.049 | 0.095 | -0.021 | 0.212 | 0.109 | 0.261 |
| XL-HDL | -0.258 | -0.363 | -0.153 | <0.001 | <0.001 | -0.021 | -0.135 | 0.094 | 0.723 | 0.857 |
| HDL | 0.116 | 0.010 | 0.221 | 0.032 | 0.052 | 0.220 | 0.109 | 0.330 | <0.001 | 0.001 |
| Total | 0.079 | -0.018 | 0.177 | 0.111 | 0.153 | 0.071 | -0.032 | 0.174 | 0.175 | 0.372 |
| **Triglycerides** |  |  |  |  |  |  |  |  |  |  |
| VS-VLDL | 0.212 | 0.113 | 0.310 | <0.001 | <0.001 | 0.049 | -0.054 | 0.152 | 0.350 | 0.581 |
| S-VLDL | 0.170 | 0.070 | 0.270 | 0.001 | 0.002 | 0.006 | -0.099 | 0.110 | 0.914 | 0.968 |
| M-VLDL | 0.145 | 0.045 | 0.245 | 0.004 | 0.008 | 0.002 | -0.103 | 0.108 | 0.970 | 0.983 |
| L-VLDL | 0.187 | 0.087 | 0.288 | <0.001 | 0.001 | 0.031 | -0.076 | 0.138 | 0.572 | 0.786 |
| XL-VLDL | 0.253 | 0.152 | 0.355 | <0.001 | <0.001 | 0.072 | -0.036 | 0.180 | 0.193 | 0.385 |
| XXL-VLDL | 0.260 | 0.160 | 0.360 | <0.001 | <0.001 | 0.098 | -0.008 | 0.204 | 0.069 | 0.197 |
| VLDL | 0.216 | 0.116 | 0.317 | <0.001 | <0.001 | 0.048 | -0.059 | 0.155 | 0.379 | 0.612 |
| S-LDL | 0.234 | 0.133 | 0.334 | <0.001 | <0.001 | 0.071 | -0.034 | 0.176 | 0.186 | 0.383 |
| M-LDL | 0.264 | 0.164 | 0.364 | <0.001 | <0.001 | 0.099 | -0.006 | 0.203 | 0.065 | 0.197 |
| L-LDL | 0.268 | 0.169 | 0.366 | <0.001 | <0.001 | 0.114 | 0.011 | 0.217 | 0.030 | 0.113 |
| LDL | 0.266 | 0.166 | 0.365 | <0.001 | <0.001 | 0.106 | 0.002 | 0.210 | 0.045 | 0.153 |
| IDL | 0.231 | 0.133 | 0.330 | <0.001 | <0.001 | 0.086 | -0.016 | 0.188 | 0.097 | 0.242 |
| S-HDL | 0.301 | 0.201 | 0.401 | <0.001 | <0.001 | 0.088 | -0.018 | 0.195 | 0.105 | 0.256 |
| M-HDL | 0.278 | 0.181 | 0.375 | <0.001 | <0.001 | 0.118 | 0.018 | 0.218 | 0.020 | 0.088 |
| L-HDL | 0.079 | -0.020 | 0.178 | 0.116 | 0.157 | 0.060 | -0.040 | 0.159 | 0.240 | 0.446 |
| XL-HDL | 0.062 | -0.036 | 0.160 | 0.217 | 0.279 | 0.019 | -0.081 | 0.118 | 0.711 | 0.857 |
| HDL | 0.242 | 0.145 | 0.339 | <0.001 | <0.001 | 0.093 | -0.007 | 0.193 | 0.068 | 0.197 |
| Total | 0.231 | 0.131 | 0.331 | <0.001 | <0.001 | 0.060 | -0.046 | 0.167 | 0.264 | 0.471 |
| **Triglycerides to Total lipids** |  |  |  |  |  |  |  |  |  |  |
| VS-VLDL | 0.412 | 0.310 | 0.514 | <0.001 | <0.001 | 0.181 | 0.067 | 0.295 | 0.002 | 0.013 |
| S-VLDL | 0.296 | 0.197 | 0.395 | <0.001 | <0.001 | 0.127 | 0.021 | 0.233 | 0.019 | 0.084 |
| M-VLDL | 0.344 | 0.243 | 0.446 | <0.001 | <0.001 | 0.126 | 0.014 | 0.237 | 0.027 | 0.106 |
| L-VLDL | 0.093 | <0.001 | 0.186 | 0.050 | 0.077 | 0.094 | -0.001 | 0.189 | 0.053 | 0.171 |
| XL-VLDL | 0.270 | 0.173 | 0.368 | <0.001 | <0.001 | 0.106 | 0.004 | 0.208 | 0.042 | 0.146 |
| XXL-VLDL | 0.033 | -0.062 | 0.129 | 0.491 | 0.548 | 0.023 | -0.073 | 0.120 | 0.634 | 0.822 |
| S-LDL | 0.307 | 0.209 | 0.405 | <0.001 | <0.001 | 0.116 | 0.013 | 0.219 | 0.028 | 0.106 |
| M-LDL | 0.305 | 0.209 | 0.401 | <0.001 | <0.001 | 0.131 | 0.030 | 0.233 | 0.011 | 0.062 |
| L-LDL | 0.337 | 0.241 | 0.433 | <0.001 | <0.001 | 0.125 | 0.022 | 0.229 | 0.017 | 0.079 |
| IDL | 0.347 | 0.247 | 0.446 | <0.001 | <0.001 | 0.111 | 0.001 | 0.220 | 0.048 | 0.160 |
| S-HDL | 0.172 | 0.071 | 0.274 | 0.001 | 0.002 | -0.025 | -0.133 | 0.083 | 0.647 | 0.830 |
| M-HDL | 0.164 | 0.064 | 0.264 | 0.001 | 0.003 | -0.018 | -0.124 | 0.088 | 0.740 | 0.859 |
| L-HDL | 0.166 | 0.065 | 0.267 | 0.001 | 0.003 | -0.028 | -0.135 | 0.079 | 0.611 | 0.808 |
| XL-HDL | 0.311 | 0.209 | 0.413 | <0.001 | <0.001 | 0.099 | -0.010 | 0.207 | 0.074 | 0.203 |

† Model adjusted for age, sex, and education.
‡ Model adjusted for age, sex, education, race, socioeconomic status, body mass index, smoking status, alcohol drinking status, physical activity, social connection, hypertension, diabetes, heart disease, beta-blockers, calcium blockers, lipid-lowering, and APOE ε4 status.
Abbreviation: Apo, apolipoprotein; LDL-AD, average diameter for LDL particles; CHOL, cholesterol; CE, cholesteryl esters; TG, triglycerides; S, small; M, medium; L, large; VL, very large; XL, extremely large; VLDL, very low-density lipoprotein; LDL; low-density lipoprotein; IDL, intermediate-density lipoprotein; HDL, high-density lipoprotein; LA, linoleic acid; MUFA, monounsaturated fatty acid; n-6, omega-6 fatty acid; PUFA, polyunsaturated fatty acid; SFA, saturated fatty acid; FA, fatty acid.

**Table S11.** β coefficients and 95% confidence intervals (CIs) for the association between metabolites and brain age among *APOE* ε4 carriers: results from linear regression models

| **Metabolites** | **Basic-model ^†^** | |  |  |  | **Multi-model ^‡^** | |  |  |  |
| --- | --- | --- | --- | --- | --- | --- | --- | --- | --- | --- |
|  | **β** | **Lower** | **Upper** | ***P*-value** | **FDR-q** | **β** | **Lower** | **Upper** | ***P*-value** | **FDR-q** |
| **Amino acids & Glycolysis** |  |  |  |  |  |  |  |  |  |  |
| Valine | 0.181 | -0.006 | 0.368 | 0.057 | 0.125 | 0.146 | -0.043 | 0.334 | 0.131 | 0.593 |
| Leucine | 0.084 | -0.105 | 0.272 | 0.384 | 0.541 | 0.041 | -0.146 | 0.228 | 0.668 | 0.914 |
| Isoleucine | 0.054 | -0.131 | 0.238 | 0.567 | 0.695 | 0.022 | -0.160 | 0.205 | 0.810 | 0.982 |
| Phenylalanine | 0.110 | -0.065 | 0.284 | 0.218 | 0.339 | 0.066 | -0.106 | 0.237 | 0.455 | 0.801 |
| Tyrosine | 0.250 | 0.063 | 0.437 | 0.009 | 0.034 | 0.225 | 0.040 | 0.410 | 0.017 | 0.400 |
| Alanine | 0.080 | -0.102 | 0.261 | 0.391 | 0.547 | 0.103 | -0.075 | 0.281 | 0.255 | 0.668 |
| Glutamine | -0.276 | -0.456 | -0.096 | 0.003 | 0.019 | -0.222 | -0.399 | -0.044 | 0.014 | 0.400 |
| Glycine | -0.221 | -0.408 | -0.034 | 0.021 | 0.058 | -0.155 | -0.340 | 0.029 | 0.099 | 0.585 |
| Histidine | -0.171 | -0.356 | 0.014 | 0.070 | 0.148 | -0.164 | -0.343 | 0.016 | 0.074 | 0.585 |
| BCAAs | 0.130 | -0.058 | 0.317 | 0.175 | 0.295 | 0.090 | -0.098 | 0.278 | 0.347 | 0.715 |
| Lactate | -0.030 | -0.207 | 0.146 | 0.736 | 0.833 | -0.086 | -0.259 | 0.086 | 0.327 | 0.703 |
| Pyruvate | -0.036 | -0.213 | 0.141 | 0.690 | 0.803 | -0.054 | -0.226 | 0.119 | 0.542 | 0.838 |
| Glucose | 0.333 | 0.140 | 0.526 | 0.001 | 0.009 | 0.202 | -0.001 | 0.404 | 0.051 | 0.585 |
| Citrate | -0.178 | -0.362 | 0.005 | 0.057 | 0.125 | -0.123 | -0.304 | 0.058 | 0.183 | 0.623 |
| **Apo-LP, LP size & Other lipids** |  |  |  |  |  |  |  |  |  |  |
| ApoA1 | -0.045 | -0.245 | 0.156 | 0.664 | 0.785 | 0.020 | -0.181 | 0.221 | 0.847 | 0.984 |
| ApoB | -0.077 | -0.254 | 0.101 | 0.398 | 0.552 | 0.006 | -0.182 | 0.194 | 0.951 | 0.984 |
| ApoB/ApoA1 | -0.054 | -0.234 | 0.126 | 0.555 | 0.690 | -0.009 | -0.199 | 0.180 | 0.923 | 0.984 |
| HDL-AD | -0.296 | -0.508 | -0.085 | 0.006 | 0.026 | -0.119 | -0.345 | 0.106 | 0.299 | 0.683 |
| LDL-AD | -0.316 | -0.502 | -0.130 | 0.001 | 0.009 | -0.200 | -0.385 | -0.015 | 0.034 | 0.585 |
| VLDL-AD | 0.285 | 0.087 | 0.484 | 0.005 | 0.025 | 0.113 | -0.097 | 0.322 | 0.291 | 0.683 |
| Phosphatidylcholine | -0.014 | -0.206 | 0.178 | 0.884 | 0.921 | 0.051 | -0.140 | 0.243 | 0.600 | 0.878 |
| Phosphoglycerides | 0.031 | -0.158 | 0.220 | 0.748 | 0.835 | 0.078 | -0.110 | 0.267 | 0.416 | 0.767 |
| Sphingomyeline | -0.124 | -0.316 | 0.067 | 0.204 | 0.325 | -0.004 | -0.201 | 0.193 | 0.969 | 0.984 |
| Total choline | -0.028 | -0.220 | 0.163 | 0.771 | 0.853 | 0.044 | -0.148 | 0.237 | 0.651 | 0.903 |
| TG/Phosphoglycerides | 0.276 | 0.086 | 0.466 | 0.004 | 0.025 | 0.129 | -0.072 | 0.329 | 0.208 | 0.627 |
| **Cholesterol** |  |  |  |  |  |  |  |  |  |  |
| XS-VLDL | -0.117 | -0.298 | 0.065 | 0.207 | 0.326 | 0.022 | -0.170 | 0.213 | 0.823 | 0.982 |
| S-VLDL | -0.034 | -0.213 | 0.146 | 0.711 | 0.820 | 0.021 | -0.167 | 0.208 | 0.828 | 0.982 |
| M-VLDL | -0.159 | -0.336 | 0.018 | 0.079 | 0.160 | -0.055 | -0.243 | 0.133 | 0.566 | 0.848 |
| L-VLDL | 0.126 | -0.058 | 0.310 | 0.179 | 0.299 | 0.081 | -0.110 | 0.273 | 0.405 | 0.765 |
| VL-VLDL | 0.143 | -0.041 | 0.326 | 0.127 | 0.231 | 0.098 | -0.095 | 0.292 | 0.319 | 0.703 |
| XL-VLDL | 0.243 | 0.061 | 0.424 | 0.009 | 0.034 | 0.169 | -0.020 | 0.357 | 0.079 | 0.585 |
| VLDL | 0.007 | -0.173 | 0.186 | 0.943 | 0.962 | 0.049 | -0.140 | 0.238 | 0.610 | 0.884 |
| S-LDL | -0.045 | -0.223 | 0.134 | 0.623 | 0.751 | 0.018 | -0.170 | 0.206 | 0.852 | 0.984 |
| M-LDL | -0.013 | -0.192 | 0.167 | 0.890 | 0.924 | 0.037 | -0.151 | 0.224 | 0.702 | 0.940 |
| L-LDL | -0.119 | -0.297 | 0.058 | 0.188 | 0.308 | -0.022 | -0.207 | 0.163 | 0.816 | 0.982 |
| Clinical LDL | -0.111 | -0.288 | 0.067 | 0.222 | 0.344 | -0.009 | -0.196 | 0.178 | 0.921 | 0.984 |
| LDL | -0.085 | -0.263 | 0.093 | 0.349 | 0.503 | -0.003 | -0.188 | 0.183 | 0.976 | 0.984 |
| IDL | -0.163 | -0.345 | 0.018 | 0.078 | 0.160 | -0.007 | -0.202 | 0.187 | 0.941 | 0.984 |
| S-HDL | 0.103 | -0.082 | 0.287 | 0.275 | 0.412 | 0.071 | -0.111 | 0.254 | 0.444 | 0.795 |
| M-HDL | -0.051 | -0.252 | 0.149 | 0.616 | 0.749 | 0.006 | -0.197 | 0.208 | 0.957 | 0.984 |
| L-HDL | -0.301 | -0.512 | -0.090 | 0.005 | 0.025 | -0.145 | -0.371 | 0.081 | 0.209 | 0.627 |
| XL-HDL | -0.324 | -0.533 | -0.115 | 0.002 | 0.018 | -0.136 | -0.360 | 0.089 | 0.236 | 0.657 |
| HDL | -0.181 | -0.388 | 0.026 | 0.087 | 0.167 | -0.060 | -0.275 | 0.154 | 0.581 | 0.866 |
| Total | -0.128 | -0.311 | 0.055 | 0.170 | 0.292 | -0.007 | -0.199 | 0.185 | 0.945 | 0.984 |
| Remnant | -0.076 | -0.255 | 0.102 | 0.401 | 0.552 | 0.026 | -0.164 | 0.216 | 0.789 | 0.982 |
| Total-HDLC | -0.082 | -0.261 | 0.096 | 0.364 | 0.522 | 0.011 | -0.177 | 0.200 | 0.909 | 0.984 |
| **Cholesterol to Total lipids** |  |  |  |  |  |  |  |  |  |  |
| VS-VLDL | -0.318 | -0.501 | -0.135 | 0.001 | 0.009 | -0.120 | -0.318 | 0.078 | 0.235 | 0.657 |
| S-VLDL | -0.252 | -0.432 | -0.073 | 0.006 | 0.026 | -0.070 | -0.256 | 0.116 | 0.462 | 0.804 |
| M-VLDL | -0.364 | -0.550 | -0.177 | <0.001 | 0.004 | -0.140 | -0.340 | 0.060 | 0.170 | 0.623 |
| L-VLDL | -0.169 | -0.356 | 0.018 | 0.076 | 0.159 | -0.008 | -0.197 | 0.181 | 0.936 | 0.984 |
| XL-VLDL | -0.264 | -0.450 | -0.077 | 0.006 | 0.026 | -0.125 | -0.318 | 0.069 | 0.207 | 0.627 |
| XXL-VLDL | -0.064 | -0.235 | 0.106 | 0.458 | 0.598 | 0.005 | -0.164 | 0.174 | 0.954 | 0.984 |
| S-LDL | -0.127 | -0.315 | 0.061 | 0.186 | 0.307 | -0.029 | -0.219 | 0.162 | 0.768 | 0.982 |
| M-LDL | -0.209 | -0.397 | -0.020 | 0.030 | 0.079 | -0.104 | -0.297 | 0.090 | 0.295 | 0.683 |
| L-LDL | -0.369 | -0.559 | -0.180 | <0.001 | 0.004 | -0.249 | -0.447 | -0.050 | 0.014 | 0.400 |
| IDL | -0.250 | -0.433 | -0.067 | 0.007 | 0.030 | -0.084 | -0.275 | 0.107 | 0.390 | 0.754 |
| S-HDL | -0.242 | -0.417 | -0.068 | 0.007 | 0.028 | -0.162 | -0.336 | 0.012 | 0.067 | 0.585 |
| M-HDL | -0.265 | -0.450 | -0.080 | 0.005 | 0.025 | -0.173 | -0.367 | 0.021 | 0.080 | 0.585 |
| L-HDL | -0.438 | -0.631 | -0.245 | <0.001 | 0.001 | -0.270 | -0.480 | -0.061 | 0.011 | 0.400 |
| XL-HDL | 0.129 | -0.071 | 0.329 | 0.206 | 0.326 | 0.026 | -0.172 | 0.223 | 0.798 | 0.982 |
| **Cholesteryl esters to Total lipids** |  |  |  |  |  |  |  |  |  |  |
| VS-VLDL | -0.324 | -0.508 | -0.140 | 0.001 | 0.009 | -0.117 | -0.317 | 0.083 | 0.251 | 0.668 |
| S-VLDL | -0.195 | -0.376 | -0.014 | 0.035 | 0.087 | -0.024 | -0.209 | 0.160 | 0.795 | 0.982 |
| M-VLDL | -0.378 | -0.565 | -0.191 | <0.001 | 0.003 | -0.159 | -0.359 | 0.042 | 0.120 | 0.585 |
| L-VLDL | -0.265 | -0.453 | -0.077 | 0.006 | 0.026 | -0.075 | -0.270 | 0.120 | 0.452 | 0.801 |
| XL-VLDL | -0.259 | -0.446 | -0.071 | 0.007 | 0.029 | -0.131 | -0.325 | 0.063 | 0.187 | 0.623 |
| XXL-VLDL | -0.075 | -0.241 | 0.092 | 0.379 | 0.536 | -0.019 | -0.183 | 0.145 | 0.820 | 0.982 |
| S-LDL | 0.195 | <0.001 | 0.389 | 0.049 | 0.114 | 0.164 | -0.033 | 0.361 | 0.102 | 0.585 |
| M-LDL | 0.175 | -0.015 | 0.364 | 0.071 | 0.150 | 0.102 | -0.091 | 0.295 | 0.300 | 0.683 |
| L-LDL | -0.172 | -0.363 | 0.020 | 0.079 | 0.160 | -0.185 | -0.374 | 0.003 | 0.054 | 0.585 |
| IDL | -0.218 | -0.403 | -0.033 | 0.021 | 0.058 | -0.081 | -0.272 | 0.109 | 0.403 | 0.765 |
| S-HDL | -0.183 | -0.358 | -0.008 | 0.040 | 0.096 | -0.139 | -0.311 | 0.033 | 0.113 | 0.585 |
| M-HDL | -0.235 | -0.414 | -0.055 | 0.010 | 0.038 | -0.172 | -0.357 | 0.014 | 0.070 | 0.585 |
| L-HDL | -0.399 | -0.586 | -0.213 | <0.001 | 0.001 | -0.271 | -0.472 | -0.071 | 0.008 | 0.332 |
| XL-HDL | -0.138 | -0.324 | 0.047 | 0.143 | 0.254 | -0.099 | -0.281 | 0.084 | 0.288 | 0.683 |
| **Cholestryl esters** |  |  |  |  |  |  |  |  |  |  |
| VS-VLDL | -0.142 | -0.325 | 0.040 | 0.125 | 0.229 | 0.012 | -0.182 | 0.205 | 0.906 | 0.984 |
| S-VLDL | -0.011 | -0.191 | 0.169 | 0.907 | 0.937 | 0.035 | -0.152 | 0.223 | 0.711 | 0.942 |
| M-VLDL | -0.223 | -0.400 | -0.046 | 0.013 | 0.047 | -0.091 | -0.278 | 0.097 | 0.342 | 0.715 |
| L-VLDL | 0.063 | -0.120 | 0.246 | 0.500 | 0.632 | 0.048 | -0.142 | 0.239 | 0.618 | 0.890 |
| VL-VLDL | 0.089 | -0.095 | 0.272 | 0.342 | 0.495 | 0.065 | -0.129 | 0.258 | 0.511 | 0.821 |
| XL-VLDL | 0.228 | 0.047 | 0.409 | 0.014 | 0.048 | 0.159 | -0.030 | 0.347 | 0.099 | 0.585 |
| VLDL | -0.042 | -0.221 | 0.137 | 0.645 | 0.772 | 0.027 | -0.162 | 0.216 | 0.781 | 0.982 |
| S-LDL | -0.002 | -0.181 | 0.177 | 0.983 | 0.987 | 0.046 | -0.142 | 0.235 | 0.629 | 0.894 |
| M-LDL | 0.023 | -0.157 | 0.203 | 0.804 | 0.878 | 0.057 | -0.132 | 0.245 | 0.555 | 0.847 |
| L-LDL | -0.104 | -0.282 | 0.073 | 0.248 | 0.377 | -0.019 | -0.203 | 0.166 | 0.842 | 0.984 |
| LDL | -0.062 | -0.240 | 0.116 | 0.495 | 0.629 | 0.007 | -0.178 | 0.193 | 0.937 | 0.984 |
| IDL | -0.162 | -0.344 | 0.020 | 0.080 | 0.161 | -0.011 | -0.205 | 0.183 | 0.912 | 0.984 |
| S-HDL | 0.096 | -0.088 | 0.280 | 0.308 | 0.454 | 0.059 | -0.123 | 0.241 | 0.525 | 0.827 |
| M-HDL | -0.053 | -0.253 | 0.147 | 0.602 | 0.735 | <0.001 | -0.202 | 0.202 | 0.999 | 0.999 |
| L-HDL | -0.309 | -0.520 | -0.099 | 0.004 | 0.024 | -0.156 | -0.382 | 0.070 | 0.175 | 0.623 |
| XL-HDL | -0.334 | -0.543 | -0.124 | 0.002 | 0.014 | -0.150 | -0.376 | 0.076 | 0.194 | 0.623 |
| HDL | -0.188 | -0.394 | 0.018 | 0.074 | 0.154 | -0.072 | -0.286 | 0.142 | 0.510 | 0.821 |
| Total | -0.133 | -0.316 | 0.051 | 0.157 | 0.273 | -0.008 | -0.200 | 0.184 | 0.933 | 0.984 |
| **Fatty acids** |  |  |  |  |  |  |  |  |  |  |
| DHA | 0.018 | -0.162 | 0.198 | 0.842 | 0.896 | 0.094 | -0.083 | 0.270 | 0.298 | 0.683 |
| LA | -0.132 | -0.312 | 0.048 | 0.151 | 0.268 | -0.050 | -0.235 | 0.135 | 0.594 | 0.874 |
| MUFA | 0.218 | 0.043 | 0.393 | 0.014 | 0.048 | 0.141 | -0.040 | 0.321 | 0.127 | 0.585 |
| n-3 | 0.093 | -0.086 | 0.272 | 0.310 | 0.454 | 0.110 | -0.064 | 0.285 | 0.215 | 0.636 |
| n-6 | -0.078 | -0.260 | 0.104 | 0.399 | 0.552 | -0.016 | -0.201 | 0.169 | 0.864 | 0.984 |
| PUFA | -0.040 | -0.221 | 0.142 | 0.670 | 0.787 | 0.021 | -0.163 | 0.204 | 0.825 | 0.982 |
| SFA | 0.192 | 0.017 | 0.368 | 0.032 | 0.081 | 0.163 | -0.017 | 0.343 | 0.076 | 0.585 |
| Total | 0.140 | -0.037 | 0.317 | 0.121 | 0.223 | 0.119 | -0.061 | 0.300 | 0.195 | 0.623 |
| Unsaturation | -0.216 | -0.398 | -0.033 | 0.021 | 0.058 | -0.070 | -0.255 | 0.115 | 0.457 | 0.801 |
| DHA/FA | -0.022 | -0.199 | 0.156 | 0.812 | 0.882 | 0.076 | -0.102 | 0.254 | 0.403 | 0.765 |
| LA/FA | -0.469 | -0.648 | -0.290 | <0.001 | <0.001 | -0.316 | -0.508 | -0.125 | 0.001 | 0.269 |
| MUFA/FA | 0.302 | 0.120 | 0.484 | 0.001 | 0.010 | 0.117 | -0.077 | 0.311 | 0.238 | 0.657 |
| n-3/FA | 0.059 | -0.121 | 0.238 | 0.521 | 0.655 | 0.099 | -0.077 | 0.274 | 0.271 | 0.683 |
| n-6/n-3 | -0.089 | -0.271 | 0.094 | 0.340 | 0.495 | -0.080 | -0.261 | 0.102 | 0.390 | 0.754 |
| n-6/FA | -0.405 | -0.584 | -0.226 | <0.001 | 0.001 | -0.283 | -0.472 | -0.095 | 0.003 | 0.269 |
| PUFA/MUFA | -0.309 | -0.491 | -0.127 | 0.001 | 0.009 | -0.137 | -0.332 | 0.057 | 0.166 | 0.623 |
| PUFA/FA | -0.370 | -0.550 | -0.190 | <0.001 | 0.002 | -0.231 | -0.421 | -0.040 | 0.018 | 0.400 |
| SFA/FA | 0.301 | 0.121 | 0.481 | 0.001 | 0.010 | 0.252 | 0.071 | 0.433 | 0.006 | 0.332 |
| **Free cholesterol** |  |  |  |  |  |  |  |  |  |  |
| VS-VLDL | -0.053 | -0.232 | 0.126 | 0.564 | 0.695 | 0.043 | -0.144 | 0.230 | 0.650 | 0.903 |
| S-VLDL | -0.073 | -0.252 | 0.105 | 0.421 | 0.563 | -0.005 | -0.192 | 0.181 | 0.954 | 0.984 |
| M-VLDL | -0.065 | -0.244 | 0.114 | 0.476 | 0.614 | -0.005 | -0.193 | 0.183 | 0.958 | 0.984 |
| L-VLDL | 0.183 | -0.001 | 0.368 | 0.052 | 0.118 | 0.111 | -0.082 | 0.305 | 0.259 | 0.671 |
| VL-VLDL | 0.193 | 0.010 | 0.377 | 0.039 | 0.094 | 0.128 | -0.064 | 0.320 | 0.191 | 0.623 |
| XL-VLDL | 0.257 | 0.076 | 0.438 | 0.005 | 0.026 | 0.177 | -0.010 | 0.364 | 0.063 | 0.585 |
| VLDL | 0.072 | -0.108 | 0.252 | 0.435 | 0.576 | 0.077 | -0.112 | 0.267 | 0.424 | 0.776 |
| S-LDL | -0.152 | -0.329 | 0.026 | 0.094 | 0.179 | -0.056 | -0.240 | 0.128 | 0.549 | 0.844 |
| M-LDL | -0.105 | -0.283 | 0.072 | 0.244 | 0.373 | -0.019 | -0.203 | 0.165 | 0.839 | 0.984 |
| L-LDL | -0.157 | -0.335 | 0.022 | 0.085 | 0.165 | -0.031 | -0.218 | 0.156 | 0.748 | 0.975 |
| LDL | -0.143 | -0.321 | 0.034 | 0.113 | 0.211 | -0.030 | -0.216 | 0.155 | 0.748 | 0.975 |
| IDL | -0.161 | -0.343 | 0.020 | 0.081 | 0.162 | 0.003 | -0.190 | 0.196 | 0.975 | 0.984 |
| S-HDL | 0.107 | -0.077 | 0.291 | 0.253 | 0.383 | 0.096 | -0.087 | 0.279 | 0.303 | 0.683 |
| M-HDL | -0.042 | -0.244 | 0.161 | 0.687 | 0.803 | 0.027 | -0.176 | 0.230 | 0.796 | 0.982 |
| L-HDL | -0.266 | -0.478 | -0.053 | 0.014 | 0.048 | -0.102 | -0.327 | 0.122 | 0.372 | 0.735 |
| XL-HDL | -0.266 | -0.470 | -0.063 | 0.010 | 0.038 | -0.076 | -0.292 | 0.139 | 0.487 | 0.815 |
| HDL | -0.146 | -0.355 | 0.063 | 0.171 | 0.292 | -0.017 | -0.230 | 0.197 | 0.880 | 0.984 |
| Total | -0.114 | -0.295 | 0.067 | 0.216 | 0.338 | -0.003 | -0.194 | 0.188 | 0.975 | 0.984 |
| **Free cholesterol to Total lipids** |  |  |  |  |  |  |  |  |  |  |
| VS-VLDL | -0.210 | -0.387 | -0.033 | 0.020 | 0.058 | -0.104 | -0.284 | 0.077 | 0.261 | 0.671 |
| S-VLDL | -0.298 | -0.479 | -0.117 | 0.001 | 0.011 | -0.123 | -0.314 | 0.069 | 0.208 | 0.627 |
| M-VLDL | -0.309 | -0.496 | -0.123 | 0.001 | 0.010 | -0.080 | -0.279 | 0.119 | 0.430 | 0.781 |
| L-VLDL | 0.128 | -0.066 | 0.322 | 0.195 | 0.316 | 0.151 | -0.040 | 0.343 | 0.122 | 0.585 |
| XL-VLDL | -0.224 | -0.410 | -0.039 | 0.018 | 0.053 | -0.070 | -0.260 | 0.121 | 0.472 | 0.815 |
| XXL-VLDL | -0.019 | -0.196 | 0.158 | 0.833 | 0.891 | 0.063 | -0.115 | 0.242 | 0.488 | 0.815 |
| S-LDL | -0.303 | -0.484 | -0.122 | 0.001 | 0.010 | -0.182 | -0.367 | 0.002 | 0.052 | 0.585 |
| M-LDL | -0.321 | -0.504 | -0.139 | 0.001 | 0.009 | -0.180 | -0.370 | 0.010 | 0.063 | 0.585 |
| L-LDL | -0.324 | -0.512 | -0.137 | 0.001 | 0.009 | -0.132 | -0.331 | 0.067 | 0.194 | 0.623 |
| IDL | -0.179 | -0.357 | -0.001 | 0.049 | 0.114 | -0.036 | -0.215 | 0.143 | 0.692 | 0.937 |
| S-HDL | -0.257 | -0.450 | -0.064 | 0.009 | 0.035 | -0.090 | -0.288 | 0.108 | 0.372 | 0.735 |
| M-HDL | -0.252 | -0.459 | -0.045 | 0.017 | 0.053 | -0.052 | -0.270 | 0.165 | 0.636 | 0.894 |
| L-HDL | -0.364 | -0.575 | -0.152 | 0.001 | 0.009 | -0.067 | -0.294 | 0.160 | 0.562 | 0.848 |
| XL-HDL | 0.419 | 0.194 | 0.643 | <0.001 | 0.006 | 0.210 | -0.029 | 0.448 | 0.085 | 0.585 |
| **KB, FB & Inflammation** |  |  |  |  |  |  |  |  |  |  |
| Glycoprotein acetyls | 0.308 | 0.130 | 0.487 | 0.001 | 0.009 | 0.260 | 0.071 | 0.448 | 0.007 | 0.332 |
| 3-Hydrocybutyrate | 0.123 | -0.058 | 0.304 | 0.182 | 0.302 | 0.095 | -0.081 | 0.271 | 0.289 | 0.683 |
| Acetate | -0.058 | -0.288 | 0.173 | 0.624 | 0.751 | -0.031 | -0.252 | 0.191 | 0.787 | 0.982 |
| Acetoacetate | 0.227 | 0.044 | 0.410 | 0.015 | 0.049 | 0.175 | -0.002 | 0.353 | 0.053 | 0.585 |
| Acetone | 0.174 | -0.004 | 0.352 | 0.055 | 0.124 | 0.145 | -0.027 | 0.318 | 0.099 | 0.585 |
| Albumin | -0.075 | -0.255 | 0.104 | 0.410 | 0.561 | -0.043 | -0.221 | 0.135 | 0.635 | 0.894 |
| Creatinine | -0.137 | -0.349 | 0.074 | 0.202 | 0.325 | -0.163 | -0.371 | 0.045 | 0.125 | 0.585 |
| **Lipoprotein particles** |  |  |  |  |  |  |  |  |  |  |
| VS-VLDL | -0.030 | -0.208 | 0.148 | 0.742 | 0.833 | 0.055 | -0.131 | 0.240 | 0.563 | 0.848 |
| S-VLDL | 0.071 | -0.109 | 0.251 | 0.438 | 0.577 | 0.058 | -0.127 | 0.243 | 0.540 | 0.838 |
| M-VLDL | -0.003 | -0.183 | 0.177 | 0.973 | 0.981 | 0.024 | -0.164 | 0.212 | 0.801 | 0.982 |
| L-VLDL | 0.193 | 0.010 | 0.376 | 0.039 | 0.094 | 0.112 | -0.079 | 0.302 | 0.252 | 0.668 |
| VL-VLDL | 0.228 | 0.045 | 0.411 | 0.014 | 0.048 | 0.139 | -0.052 | 0.331 | 0.154 | 0.604 |
| XL-VLDL | 0.266 | 0.085 | 0.446 | 0.004 | 0.024 | 0.178 | -0.009 | 0.365 | 0.062 | 0.585 |
| VLDL | 0.053 | -0.125 | 0.232 | 0.558 | 0.691 | 0.067 | -0.120 | 0.253 | 0.484 | 0.815 |
| S-LDL | -0.024 | -0.202 | 0.153 | 0.791 | 0.868 | 0.039 | -0.148 | 0.226 | 0.684 | 0.931 |
| M-LDL | 0.004 | -0.175 | 0.183 | 0.963 | 0.978 | 0.066 | -0.121 | 0.254 | 0.487 | 0.815 |
| L-LDL | -0.125 | -0.302 | 0.051 | 0.164 | 0.284 | -0.041 | -0.227 | 0.144 | 0.662 | 0.911 |
| LDL | -0.081 | -0.258 | 0.096 | 0.371 | 0.528 | -0.003 | -0.190 | 0.184 | 0.974 | 0.984 |
| IDL | -0.123 | -0.300 | 0.055 | 0.175 | 0.295 | 0.002 | -0.188 | 0.193 | 0.980 | 0.984 |
| S-HDL | 0.123 | -0.061 | 0.307 | 0.191 | 0.310 | 0.081 | -0.102 | 0.265 | 0.386 | 0.754 |
| M-HDL | -0.019 | -0.218 | 0.180 | 0.851 | 0.897 | 0.030 | -0.170 | 0.229 | 0.771 | 0.982 |
| L-HDL | -0.289 | -0.501 | -0.077 | 0.008 | 0.030 | -0.136 | -0.361 | 0.089 | 0.237 | 0.657 |
| XL-HDL | -0.303 | -0.512 | -0.094 | 0.004 | 0.025 | -0.120 | -0.342 | 0.101 | 0.288 | 0.683 |
| HDL | -0.021 | -0.216 | 0.174 | 0.834 | 0.891 | 0.024 | -0.170 | 0.218 | 0.807 | 0.982 |
| Total | -0.034 | -0.227 | 0.160 | 0.733 | 0.833 | 0.024 | -0.169 | 0.217 | 0.808 | 0.982 |
| **Phospholipids** |  |  |  |  |  |  |  |  |  |  |
| VS-VLDL | -0.008 | -0.186 | 0.169 | 0.927 | 0.950 | 0.061 | -0.122 | 0.245 | 0.513 | 0.821 |
| S-VLDL | -0.009 | -0.188 | 0.170 | 0.920 | 0.947 | 0.027 | -0.160 | 0.213 | 0.780 | 0.982 |
| M-VLDL | -0.020 | -0.200 | 0.159 | 0.825 | 0.891 | 0.015 | -0.173 | 0.203 | 0.873 | 0.984 |
| L-VLDL | 0.195 | 0.011 | 0.379 | 0.038 | 0.093 | 0.112 | -0.081 | 0.304 | 0.255 | 0.668 |
| VL-VLDL | 0.213 | 0.029 | 0.396 | 0.023 | 0.062 | 0.136 | -0.056 | 0.329 | 0.164 | 0.623 |
| XL-VLDL | 0.271 | 0.090 | 0.452 | 0.003 | 0.022 | 0.185 | -0.003 | 0.372 | 0.053 | 0.585 |
| VLDL | 0.108 | -0.072 | 0.288 | 0.240 | 0.369 | 0.092 | -0.096 | 0.281 | 0.337 | 0.711 |
| S-LDL | -0.071 | -0.248 | 0.106 | 0.433 | 0.576 | -0.004 | -0.189 | 0.182 | 0.969 | 0.984 |
| M-LDL | -0.015 | -0.193 | 0.164 | 0.873 | 0.914 | 0.035 | -0.151 | 0.222 | 0.709 | 0.942 |
| L-LDL | -0.095 | -0.274 | 0.084 | 0.297 | 0.441 | 0.009 | -0.178 | 0.197 | 0.921 | 0.984 |
| LDL | -0.069 | -0.247 | 0.109 | 0.448 | 0.587 | 0.016 | -0.171 | 0.203 | 0.869 | 0.984 |
| IDL | -0.152 | -0.333 | 0.030 | 0.101 | 0.191 | -0.007 | -0.200 | 0.186 | 0.942 | 0.984 |
| S-HDL | 0.217 | 0.034 | 0.401 | 0.020 | 0.058 | 0.150 | -0.033 | 0.333 | 0.108 | 0.585 |
| M-HDL | 0.081 | -0.114 | 0.276 | 0.416 | 0.563 | 0.091 | -0.102 | 0.284 | 0.354 | 0.717 |
| L-HDL | -0.214 | -0.426 | -0.002 | 0.047 | 0.112 | -0.078 | -0.299 | 0.143 | 0.491 | 0.815 |
| XL-HDL | -0.287 | -0.496 | -0.077 | 0.007 | 0.030 | -0.112 | -0.334 | 0.110 | 0.322 | 0.703 |
| HDL | -0.029 | -0.233 | 0.174 | 0.777 | 0.856 | 0.040 | -0.164 | 0.244 | 0.699 | 0.940 |
| Total | -0.018 | -0.205 | 0.170 | 0.854 | 0.897 | 0.064 | -0.126 | 0.255 | 0.507 | 0.821 |
| **Phospholipids to Total lipids** |  |  |  |  |  |  |  |  |  |  |
| VS-VLDL | 0.076 | -0.108 | 0.260 | 0.420 | 0.563 | -0.017 | -0.203 | 0.169 | 0.860 | 0.984 |
| S-VLDL | -0.291 | -0.471 | -0.111 | 0.002 | 0.012 | -0.126 | -0.314 | 0.063 | 0.191 | 0.623 |
| M-VLDL | -0.286 | -0.474 | -0.097 | 0.003 | 0.020 | -0.054 | -0.256 | 0.149 | 0.603 | 0.878 |
| L-VLDL | 0.166 | -0.029 | 0.361 | 0.095 | 0.180 | 0.111 | -0.088 | 0.311 | 0.272 | 0.683 |
| VL-VLDL | -0.033 | -0.236 | 0.170 | 0.751 | 0.835 | 0.020 | -0.185 | 0.224 | 0.849 | 0.984 |
| XL-VLDL | 0.163 | -0.021 | 0.346 | 0.082 | 0.162 | 0.126 | -0.057 | 0.309 | 0.178 | 0.623 |
| S-LDL | -0.187 | -0.384 | 0.009 | 0.061 | 0.133 | -0.148 | -0.345 | 0.049 | 0.142 | 0.604 |
| M-LDL | -0.131 | -0.312 | 0.050 | 0.157 | 0.273 | -0.081 | -0.258 | 0.097 | 0.372 | 0.735 |
| L-LDL | 0.042 | -0.148 | 0.232 | 0.665 | 0.785 | 0.078 | -0.109 | 0.264 | 0.413 | 0.767 |
| IDL | -0.061 | -0.251 | 0.129 | 0.530 | 0.664 | -0.116 | -0.304 | 0.072 | 0.227 | 0.657 |
| S-HDL | 0.142 | -0.042 | 0.327 | 0.130 | 0.235 | 0.116 | -0.064 | 0.297 | 0.207 | 0.627 |
| M-HDL | 0.318 | 0.130 | 0.505 | 0.001 | 0.009 | 0.207 | 0.008 | 0.405 | 0.041 | 0.585 |
| L-HDL | 0.517 | 0.328 | 0.707 | <0.001 | <0.001 | 0.327 | 0.116 | 0.539 | 0.002 | 0.269 |
| XL-HDL | -0.261 | -0.473 | -0.049 | 0.016 | 0.050 | -0.107 | -0.324 | 0.109 | 0.332 | 0.706 |
| **Total lipids** |  |  |  |  |  |  |  |  |  |  |
| VS-VLDL | -0.020 | -0.198 | 0.159 | 0.830 | 0.891 | 0.065 | -0.121 | 0.250 | 0.495 | 0.816 |
| S-VLDL | 0.067 | -0.113 | 0.247 | 0.465 | 0.603 | 0.060 | -0.125 | 0.246 | 0.525 | 0.827 |
| M-VLDL | 0.034 | -0.147 | 0.214 | 0.714 | 0.820 | 0.031 | -0.157 | 0.219 | 0.744 | 0.975 |
| L-VLDL | 0.182 | -0.002 | 0.366 | 0.052 | 0.118 | 0.097 | -0.094 | 0.289 | 0.320 | 0.703 |
| VL-VLDL | 0.232 | 0.049 | 0.415 | 0.013 | 0.047 | 0.142 | -0.049 | 0.334 | 0.146 | 0.604 |
| XL-VLDL | 0.259 | 0.078 | 0.439 | 0.005 | 0.025 | 0.171 | -0.016 | 0.358 | 0.073 | 0.585 |
| VLDL | 0.148 | -0.033 | 0.328 | 0.109 | 0.204 | 0.105 | -0.083 | 0.294 | 0.274 | 0.683 |
| S-LDL | -0.032 | -0.210 | 0.146 | 0.724 | 0.827 | 0.025 | -0.163 | 0.212 | 0.797 | 0.982 |
| M-LDL | 0.001 | -0.178 | 0.181 | 0.989 | 0.989 | 0.046 | -0.142 | 0.233 | 0.633 | 0.894 |
| L-LDL | -0.096 | -0.274 | 0.082 | 0.290 | 0.432 | -0.003 | -0.188 | 0.183 | 0.978 | 0.984 |
| LDL | -0.063 | -0.241 | 0.114 | 0.484 | 0.622 | 0.014 | -0.172 | 0.199 | 0.887 | 0.984 |
| IDL | -0.139 | -0.321 | 0.043 | 0.134 | 0.241 | 0.008 | -0.186 | 0.202 | 0.935 | 0.984 |
| S-HDL | 0.199 | 0.015 | 0.383 | 0.034 | 0.085 | 0.136 | -0.048 | 0.319 | 0.148 | 0.604 |
| M-HDL | 0.033 | -0.164 | 0.231 | 0.743 | 0.833 | 0.061 | -0.135 | 0.257 | 0.542 | 0.838 |
| L-HDL | -0.256 | -0.468 | -0.044 | 0.018 | 0.053 | -0.107 | -0.331 | 0.116 | 0.347 | 0.715 |
| XL-HDL | -0.300 | -0.510 | -0.091 | 0.005 | 0.025 | -0.118 | -0.341 | 0.105 | 0.299 | 0.683 |
| HDL | -0.086 | -0.291 | 0.120 | 0.414 | 0.563 | 0.006 | -0.201 | 0.214 | 0.952 | 0.984 |
| Total | 0.004 | -0.176 | 0.183 | 0.968 | 0.980 | 0.062 | -0.124 | 0.248 | 0.514 | 0.821 |
| **Triglycerides** |  |  |  |  |  |  |  |  |  |  |
| VS-VLDL | 0.177 | 0.004 | 0.350 | 0.045 | 0.108 | 0.128 | -0.048 | 0.303 | 0.154 | 0.604 |
| S-VLDL | 0.173 | -0.005 | 0.351 | 0.056 | 0.125 | 0.095 | -0.086 | 0.276 | 0.305 | 0.683 |
| M-VLDL | 0.160 | -0.022 | 0.342 | 0.085 | 0.165 | 0.078 | -0.109 | 0.266 | 0.412 | 0.767 |
| L-VLDL | 0.199 | 0.015 | 0.383 | 0.034 | 0.085 | 0.095 | -0.095 | 0.286 | 0.326 | 0.703 |
| XL-VLDL | 0.262 | 0.079 | 0.444 | 0.005 | 0.025 | 0.155 | -0.036 | 0.345 | 0.111 | 0.585 |
| XXL-VLDL | 0.257 | 0.077 | 0.438 | 0.005 | 0.025 | 0.166 | -0.021 | 0.352 | 0.082 | 0.585 |
| VLDL | 0.220 | 0.039 | 0.401 | 0.017 | 0.053 | 0.127 | -0.061 | 0.314 | 0.186 | 0.623 |
| S-LDL | 0.208 | 0.034 | 0.381 | 0.019 | 0.056 | 0.144 | -0.034 | 0.323 | 0.113 | 0.585 |
| M-LDL | 0.208 | 0.036 | 0.381 | 0.018 | 0.053 | 0.151 | -0.025 | 0.328 | 0.092 | 0.585 |
| L-LDL | 0.195 | 0.023 | 0.367 | 0.027 | 0.071 | 0.156 | -0.019 | 0.331 | 0.081 | 0.585 |
| LDL | 0.202 | 0.030 | 0.374 | 0.022 | 0.059 | 0.155 | -0.021 | 0.331 | 0.083 | 0.585 |
| IDL | 0.165 | -0.008 | 0.337 | 0.062 | 0.133 | 0.133 | -0.042 | 0.308 | 0.136 | 0.601 |
| S-HDL | 0.274 | 0.094 | 0.454 | 0.003 | 0.020 | 0.178 | -0.009 | 0.365 | 0.062 | 0.585 |
| M-HDL | 0.218 | 0.042 | 0.394 | 0.015 | 0.049 | 0.154 | -0.022 | 0.329 | 0.087 | 0.585 |
| L-HDL | 0.042 | -0.140 | 0.224 | 0.654 | 0.779 | 0.062 | -0.115 | 0.239 | 0.491 | 0.815 |
| XL-HDL | 0.061 | -0.113 | 0.235 | 0.493 | 0.629 | 0.068 | -0.104 | 0.239 | 0.439 | 0.792 |
| HDL | 0.196 | 0.021 | 0.371 | 0.028 | 0.073 | 0.138 | -0.036 | 0.312 | 0.121 | 0.585 |
| Total | 0.220 | 0.041 | 0.398 | 0.016 | 0.050 | 0.135 | -0.050 | 0.319 | 0.153 | 0.604 |
| **Triglycerides to Total lipids** |  |  |  |  |  |  |  |  |  |  |
| VS-VLDL | 0.337 | 0.155 | 0.519 | <0.001 | 0.006 | 0.137 | -0.059 | 0.334 | 0.171 | 0.623 |
| S-VLDL | 0.268 | 0.089 | 0.447 | 0.003 | 0.022 | 0.088 | -0.099 | 0.275 | 0.354 | 0.717 |
| M-VLDL | 0.352 | 0.165 | 0.539 | <0.001 | 0.006 | 0.124 | -0.076 | 0.325 | 0.224 | 0.657 |
| L-VLDL | 0.037 | -0.155 | 0.230 | 0.703 | 0.814 | -0.053 | -0.243 | 0.138 | 0.588 | 0.872 |
| XL-VLDL | 0.263 | 0.079 | 0.448 | 0.005 | 0.025 | 0.112 | -0.079 | 0.302 | 0.250 | 0.668 |
| XXL-VLDL | 0.017 | -0.158 | 0.192 | 0.850 | 0.897 | -0.040 | -0.213 | 0.133 | 0.653 | 0.903 |
| S-LDL | 0.306 | 0.123 | 0.488 | 0.001 | 0.010 | 0.171 | -0.016 | 0.357 | 0.074 | 0.585 |
| M-LDL | 0.281 | 0.099 | 0.462 | 0.002 | 0.018 | 0.153 | -0.033 | 0.339 | 0.108 | 0.585 |
| L-LDL | 0.331 | 0.148 | 0.514 | <0.001 | 0.007 | 0.194 | 0.004 | 0.384 | 0.046 | 0.585 |
| IDL | 0.317 | 0.136 | 0.497 | 0.001 | 0.009 | 0.149 | -0.042 | 0.341 | 0.126 | 0.585 |
| S-HDL | 0.244 | 0.060 | 0.427 | 0.009 | 0.035 | 0.151 | -0.039 | 0.342 | 0.120 | 0.585 |
| M-HDL | 0.211 | 0.028 | 0.393 | 0.024 | 0.064 | 0.137 | -0.052 | 0.325 | 0.155 | 0.604 |
| L-HDL | 0.259 | 0.063 | 0.455 | 0.010 | 0.036 | 0.153 | -0.049 | 0.354 | 0.138 | 0.601 |
| XL-HDL | 0.314 | 0.098 | 0.530 | 0.004 | 0.025 | 0.179 | -0.046 | 0.404 | 0.119 | 0.585 |

† Model adjusted for age, sex, and education.
‡ Model adjusted for age, sex, education, race, socioeconomic status, body mass index, smoking status, alcohol drinking status, physical activity, social connection, hypertension, diabetes, heart disease, beta-blockers, calcium blockers, lipid-lowering, and APOE ε4 status.
Abbreviation: Apo, apolipoprotein; LDL-AD, average diameter for LDL particles; CHOL, cholesterol; CE, cholesteryl esters; TG, triglycerides; S, small; M, medium; L, large; VL, very large; XL, extremely large; VLDL, very low-density lipoprotein; LDL; low-density lipoprotein; IDL, intermediate-density lipoprotein; HDL, high-density lipoprotein; LA, linoleic acid; MUFA, monounsaturated fatty acid; n-6, omega-6 fatty acid; PUFA, polyunsaturated fatty acid; SFA, saturated fatty acid; FA, fatty acid.

**Table S12.** β coefficients and 95% confidence intervals (CIs) for the association between metabolites and brain age gap (BAG) among *APOE* ε4 carriers: results from linear regression models

| **Metabolites** | **Basic-model ^†^** | |  |  |  | **Multi-model ^‡^** | |  |  |  |
| --- | --- | --- | --- | --- | --- | --- | --- | --- | --- | --- |
|  | **β** | **Lower** | **Upper** | ***P*-value** | **FDR-q** | **β** | **Lower** | **Upper** | ***P*-value** | **FDR-q** |
| **Amino acids & Glycolysis** |  |  |  |  |  |  |  |  |  |  |
| Valine | 0.151 | -0.022 | 0.325 | 0.087 | 0.150 | 0.080 | -0.094 | 0.254 | 0.369 | 0.642 |
| Leucine | 0.051 | -0.124 | 0.226 | 0.570 | 0.673 | -0.016 | -0.188 | 0.156 | 0.852 | 0.934 |
| Isoleucine | 0.022 | -0.150 | 0.193 | 0.803 | 0.877 | -0.033 | -0.201 | 0.135 | 0.697 | 0.848 |
| Phenylalanine | 0.090 | -0.072 | 0.252 | 0.278 | 0.375 | 0.039 | -0.119 | 0.197 | 0.628 | 0.836 |
| Tyrosine | 0.170 | -0.003 | 0.344 | 0.055 | 0.102 | 0.130 | -0.041 | 0.300 | 0.136 | 0.427 |
| Alanine | 0.152 | -0.017 | 0.321 | 0.077 | 0.135 | 0.157 | -0.007 | 0.320 | 0.061 | 0.421 |
| Glutamine | -0.266 | -0.433 | -0.098 | 0.002 | 0.007 | -0.210 | -0.374 | -0.047 | 0.012 | 0.294 |
| Glycine | -0.222 | -0.395 | -0.048 | 0.012 | 0.032 | -0.140 | -0.310 | 0.030 | 0.107 | 0.421 |
| Histidine | -0.185 | -0.356 | -0.013 | 0.035 | 0.072 | -0.177 | -0.342 | -0.012 | 0.036 | 0.421 |
| BCAAs | 0.097 | -0.077 | 0.271 | 0.275 | 0.375 | 0.026 | -0.147 | 0.199 | 0.767 | 0.892 |
| Lactate | 0.108 | -0.056 | 0.272 | 0.196 | 0.290 | 0.046 | -0.113 | 0.205 | 0.569 | 0.810 |
| Pyruvate | 0.100 | -0.065 | 0.264 | 0.235 | 0.331 | 0.078 | -0.081 | 0.236 | 0.336 | 0.607 |
| Glucose | 0.346 | 0.166 | 0.525 | <0.001 | 0.001 | 0.176 | -0.010 | 0.362 | 0.064 | 0.421 |
| Citrate | -0.145 | -0.316 | 0.025 | 0.095 | 0.161 | -0.088 | -0.254 | 0.079 | 0.302 | 0.562 |
| **Apo-LP, LP size & Other lipids** |  |  |  |  |  |  |  |  |  |  |
| ApoA1 | -0.014 | -0.201 | 0.172 | 0.882 | 0.926 | 0.067 | -0.118 | 0.252 | 0.475 | 0.754 |
| ApoB | -0.099 | -0.263 | 0.066 | 0.241 | 0.336 | -0.034 | -0.207 | 0.140 | 0.703 | 0.848 |
| ApoB/ApoA1 | -0.093 | -0.260 | 0.075 | 0.278 | 0.375 | -0.073 | -0.248 | 0.101 | 0.409 | 0.683 |
| HDL-AD | -0.344 | -0.540 | -0.148 | 0.001 | 0.003 | -0.136 | -0.343 | 0.071 | 0.199 | 0.486 |
| LDL-AD | -0.326 | -0.499 | -0.154 | <0.001 | 0.001 | -0.189 | -0.360 | -0.019 | 0.029 | 0.421 |
| VLDL-AD | 0.321 | 0.137 | 0.506 | 0.001 | 0.003 | 0.116 | -0.077 | 0.309 | 0.237 | 0.510 |
| Phosphatidylcholine | -0.014 | -0.192 | 0.165 | 0.881 | 0.926 | 0.052 | -0.125 | 0.228 | 0.566 | 0.810 |
| Phosphoglycerides | 0.041 | -0.135 | 0.217 | 0.647 | 0.738 | 0.085 | -0.089 | 0.258 | 0.340 | 0.609 |
| Sphingomyeline | -0.116 | -0.294 | 0.062 | 0.203 | 0.298 | 0.009 | -0.172 | 0.191 | 0.919 | 0.969 |
| Total choline | -0.023 | -0.201 | 0.154 | 0.797 | 0.875 | 0.049 | -0.128 | 0.226 | 0.588 | 0.817 |
| TG/Phosphoglycerides | 0.295 | 0.119 | 0.471 | 0.001 | 0.005 | 0.112 | -0.072 | 0.297 | 0.232 | 0.510 |
| **Cholesterol** |  |  |  |  |  |  |  |  |  |  |
| XS-VLDL | -0.169 | -0.337 | -0.001 | 0.049 | 0.094 | -0.049 | -0.225 | 0.127 | 0.586 | 0.817 |
| S-VLDL | -0.050 | -0.216 | 0.117 | 0.559 | 0.665 | -0.016 | -0.189 | 0.156 | 0.855 | 0.934 |
| M-VLDL | -0.188 | -0.352 | -0.023 | 0.025 | 0.055 | -0.097 | -0.270 | 0.076 | 0.270 | 0.529 |
| L-VLDL | 0.136 | -0.035 | 0.306 | 0.119 | 0.193 | 0.063 | -0.113 | 0.240 | 0.481 | 0.754 |
| VL-VLDL | 0.157 | -0.013 | 0.328 | 0.070 | 0.129 | 0.084 | -0.094 | 0.262 | 0.354 | 0.625 |
| XL-VLDL | 0.261 | 0.093 | 0.429 | 0.002 | 0.008 | 0.157 | -0.016 | 0.330 | 0.075 | 0.421 |
| VLDL | -0.008 | -0.175 | 0.158 | 0.921 | 0.944 | 0.009 | -0.165 | 0.183 | 0.918 | 0.969 |
| S-LDL | -0.049 | -0.215 | 0.117 | 0.561 | 0.665 | -0.001 | -0.173 | 0.172 | 0.995 | 0.999 |
| M-LDL | -0.009 | -0.176 | 0.158 | 0.917 | 0.943 | 0.027 | -0.146 | 0.199 | 0.762 | 0.891 |
| L-LDL | -0.136 | -0.301 | 0.028 | 0.105 | 0.174 | -0.045 | -0.216 | 0.125 | 0.600 | 0.826 |
| Clinical LDL | -0.130 | -0.295 | 0.035 | 0.123 | 0.197 | -0.038 | -0.210 | 0.134 | 0.664 | 0.847 |
| LDL | -0.095 | -0.260 | 0.070 | 0.257 | 0.355 | -0.022 | -0.193 | 0.148 | 0.796 | 0.897 |
| IDL | -0.205 | -0.374 | -0.037 | 0.017 | 0.041 | -0.059 | -0.238 | 0.120 | 0.518 | 0.772 |
| S-HDL | 0.176 | 0.005 | 0.347 | 0.044 | 0.087 | 0.142 | -0.026 | 0.310 | 0.097 | 0.421 |
| M-HDL | -0.013 | -0.199 | 0.174 | 0.894 | 0.935 | 0.067 | -0.119 | 0.254 | 0.480 | 0.754 |
| L-HDL | -0.336 | -0.532 | -0.141 | 0.001 | 0.004 | -0.145 | -0.353 | 0.063 | 0.171 | 0.459 |
| XL-HDL | -0.397 | -0.591 | -0.203 | <0.001 | 0.001 | -0.186 | -0.392 | 0.021 | 0.078 | 0.421 |
| HDL | -0.174 | -0.366 | 0.019 | 0.077 | 0.135 | -0.021 | -0.219 | 0.176 | 0.834 | 0.923 |
| Total | -0.145 | -0.315 | 0.025 | 0.094 | 0.159 | -0.028 | -0.204 | 0.149 | 0.757 | 0.889 |
| Remnant | -0.106 | -0.271 | 0.060 | 0.211 | 0.307 | -0.023 | -0.199 | 0.152 | 0.794 | 0.897 |
| Total-HDLC | -0.102 | -0.268 | 0.063 | 0.225 | 0.323 | -0.023 | -0.197 | 0.150 | 0.791 | 0.897 |
| **Cholesterol to Total lipids** |  |  |  |  |  |  |  |  |  |  |
| VS-VLDL | -0.369 | -0.538 | -0.199 | <0.001 | <0.001 | -0.152 | -0.334 | 0.030 | 0.101 | 0.421 |
| S-VLDL | -0.299 | -0.465 | -0.132 | <0.001 | 0.002 | -0.106 | -0.278 | 0.065 | 0.224 | 0.506 |
| M-VLDL | -0.426 | -0.600 | -0.253 | <0.001 | <0.001 | -0.187 | -0.372 | -0.003 | 0.046 | 0.421 |
| L-VLDL | -0.265 | -0.438 | -0.091 | 0.003 | 0.010 | -0.099 | -0.273 | 0.075 | 0.264 | 0.529 |
| XL-VLDL | -0.304 | -0.478 | -0.131 | 0.001 | 0.003 | -0.143 | -0.321 | 0.035 | 0.116 | 0.421 |
| XXL-VLDL | -0.065 | -0.223 | 0.093 | 0.421 | 0.538 | 0.013 | -0.142 | 0.168 | 0.872 | 0.949 |
| S-LDL | -0.136 | -0.310 | 0.039 | 0.127 | 0.203 | -0.031 | -0.207 | 0.144 | 0.727 | 0.866 |
| M-LDL | -0.206 | -0.381 | -0.031 | 0.021 | 0.048 | -0.085 | -0.263 | 0.094 | 0.352 | 0.625 |
| L-LDL | -0.386 | -0.562 | -0.209 | <0.001 | <0.001 | -0.238 | -0.420 | -0.055 | 0.011 | 0.294 |
| IDL | -0.282 | -0.452 | -0.112 | 0.001 | 0.005 | -0.097 | -0.273 | 0.078 | 0.277 | 0.530 |
| S-HDL | -0.241 | -0.403 | -0.079 | 0.004 | 0.012 | -0.140 | -0.300 | 0.020 | 0.086 | 0.421 |
| M-HDL | -0.258 | -0.430 | -0.086 | 0.003 | 0.011 | -0.128 | -0.307 | 0.050 | 0.158 | 0.458 |
| L-HDL | -0.462 | -0.641 | -0.283 | <0.001 | <0.001 | -0.254 | -0.446 | -0.061 | 0.010 | 0.294 |
| XL-HDL | 0.168 | -0.017 | 0.354 | 0.076 | 0.134 | 0.059 | -0.122 | 0.241 | 0.522 | 0.772 |
| **Cholesteryl esters to Total lipids** |  |  |  |  |  |  |  |  |  |  |
| VS-VLDL | -0.379 | -0.550 | -0.208 | <0.001 | <0.001 | -0.154 | -0.337 | 0.030 | 0.102 | 0.421 |
| S-VLDL | -0.245 | -0.413 | -0.077 | 0.004 | 0.013 | -0.072 | -0.241 | 0.098 | 0.407 | 0.683 |
| M-VLDL | -0.435 | -0.608 | -0.261 | <0.001 | <0.001 | -0.197 | -0.381 | -0.013 | 0.036 | 0.421 |
| L-VLDL | -0.356 | -0.530 | -0.181 | <0.001 | 0.001 | -0.156 | -0.335 | 0.023 | 0.089 | 0.421 |
| XL-VLDL | -0.279 | -0.453 | -0.105 | 0.002 | 0.007 | -0.125 | -0.303 | 0.054 | 0.171 | 0.459 |
| XXL-VLDL | -0.056 | -0.211 | 0.099 | 0.477 | 0.580 | 0.003 | -0.148 | 0.154 | 0.967 | 0.995 |
| S-LDL | 0.198 | 0.018 | 0.379 | 0.031 | 0.065 | 0.147 | -0.034 | 0.329 | 0.111 | 0.421 |
| M-LDL | 0.214 | 0.038 | 0.390 | 0.017 | 0.041 | 0.125 | -0.053 | 0.302 | 0.168 | 0.459 |
| L-LDL | -0.138 | -0.316 | 0.040 | 0.129 | 0.205 | -0.142 | -0.315 | 0.031 | 0.109 | 0.421 |
| IDL | -0.249 | -0.421 | -0.077 | 0.005 | 0.014 | -0.098 | -0.273 | 0.077 | 0.274 | 0.529 |
| S-HDL | -0.167 | -0.329 | -0.004 | 0.044 | 0.087 | -0.106 | -0.264 | 0.052 | 0.189 | 0.481 |
| M-HDL | -0.223 | -0.390 | -0.057 | 0.009 | 0.023 | -0.126 | -0.297 | 0.045 | 0.148 | 0.454 |
| L-HDL | -0.411 | -0.584 | -0.237 | <0.001 | <0.001 | -0.240 | -0.424 | -0.056 | 0.011 | 0.294 |
| XL-HDL | -0.096 | -0.268 | 0.076 | 0.275 | 0.375 | -0.038 | -0.206 | 0.130 | 0.659 | 0.847 |
| **Cholestryl esters** |  |  |  |  |  |  |  |  |  |  |
| VS-VLDL | -0.201 | -0.370 | -0.032 | 0.020 | 0.046 | -0.064 | -0.242 | 0.114 | 0.480 | 0.754 |
| S-VLDL | -0.026 | -0.193 | 0.141 | 0.759 | 0.848 | -0.003 | -0.176 | 0.170 | 0.974 | 0.997 |
| M-VLDL | -0.259 | -0.423 | -0.095 | 0.002 | 0.007 | -0.136 | -0.308 | 0.037 | 0.123 | 0.421 |
| L-VLDL | 0.063 | -0.107 | 0.233 | 0.466 | 0.572 | 0.023 | -0.152 | 0.199 | 0.794 | 0.897 |
| VL-VLDL | 0.107 | -0.063 | 0.277 | 0.218 | 0.316 | 0.058 | -0.120 | 0.236 | 0.522 | 0.772 |
| XL-VLDL | 0.255 | 0.086 | 0.423 | 0.003 | 0.010 | 0.159 | -0.015 | 0.332 | 0.073 | 0.421 |
| VLDL | -0.063 | -0.229 | 0.103 | 0.456 | 0.565 | -0.017 | -0.191 | 0.157 | 0.848 | 0.934 |
| S-LDL | -0.003 | -0.169 | 0.163 | 0.974 | 0.976 | 0.028 | -0.145 | 0.202 | 0.747 | 0.885 |
| M-LDL | 0.032 | -0.135 | 0.199 | 0.705 | 0.795 | 0.050 | -0.123 | 0.224 | 0.569 | 0.810 |
| L-LDL | -0.117 | -0.282 | 0.047 | 0.162 | 0.250 | -0.038 | -0.208 | 0.131 | 0.657 | 0.847 |
| LDL | -0.068 | -0.233 | 0.097 | 0.421 | 0.538 | -0.009 | -0.179 | 0.162 | 0.919 | 0.969 |
| IDL | -0.203 | -0.372 | -0.035 | 0.018 | 0.043 | -0.062 | -0.240 | 0.117 | 0.499 | 0.762 |
| S-HDL | 0.173 | 0.002 | 0.344 | 0.047 | 0.092 | 0.135 | -0.033 | 0.303 | 0.114 | 0.421 |
| M-HDL | -0.012 | -0.197 | 0.174 | 0.900 | 0.938 | 0.066 | -0.120 | 0.252 | 0.489 | 0.756 |
| L-HDL | -0.342 | -0.538 | -0.147 | 0.001 | 0.003 | -0.153 | -0.360 | 0.055 | 0.150 | 0.454 |
| XL-HDL | -0.399 | -0.594 | -0.205 | <0.001 | 0.001 | -0.189 | -0.396 | 0.019 | 0.075 | 0.421 |
| HDL | -0.176 | -0.367 | 0.015 | 0.071 | 0.129 | -0.026 | -0.223 | 0.170 | 0.792 | 0.897 |
| Total | -0.148 | -0.318 | 0.023 | 0.089 | 0.152 | -0.025 | -0.202 | 0.151 | 0.778 | 0.897 |
| **Fatty acids** |  |  |  |  |  |  |  |  |  |  |
| DHA | 0.003 | -0.164 | 0.170 | 0.971 | 0.976 | 0.094 | -0.068 | 0.257 | 0.254 | 0.528 |
| LA | -0.179 | -0.346 | -0.012 | 0.036 | 0.074 | -0.109 | -0.279 | 0.062 | 0.211 | 0.495 |
| MUFA | 0.261 | 0.099 | 0.424 | 0.002 | 0.007 | 0.159 | -0.007 | 0.325 | 0.061 | 0.421 |
| n-3 | 0.092 | -0.074 | 0.258 | 0.279 | 0.375 | 0.114 | -0.046 | 0.274 | 0.164 | 0.459 |
| n-6 | -0.104 | -0.273 | 0.065 | 0.227 | 0.325 | -0.052 | -0.223 | 0.118 | 0.546 | 0.795 |
| PUFA | -0.062 | -0.231 | 0.107 | 0.474 | 0.579 | -0.008 | -0.177 | 0.160 | 0.922 | 0.969 |
| SFA | 0.223 | 0.060 | 0.386 | 0.007 | 0.021 | 0.173 | 0.007 | 0.338 | 0.041 | 0.421 |
| Total | 0.160 | -0.004 | 0.324 | 0.056 | 0.104 | 0.120 | -0.046 | 0.286 | 0.157 | 0.458 |
| Unsaturation | -0.285 | -0.454 | -0.116 | 0.001 | 0.005 | -0.112 | -0.282 | 0.058 | 0.198 | 0.486 |
| DHA/FA | -0.050 | -0.215 | 0.115 | 0.551 | 0.659 | 0.074 | -0.090 | 0.238 | 0.376 | 0.645 |
| LA/FA | -0.582 | -0.748 | -0.417 | <0.001 | <0.001 | -0.423 | -0.600 | -0.247 | <0.001 | 0.001 |
| MUFA/FA | 0.380 | 0.211 | 0.548 | <0.001 | <0.001 | 0.165 | -0.013 | 0.344 | 0.069 | 0.421 |
| n-3/FA | 0.042 | -0.125 | 0.209 | 0.620 | 0.718 | 0.098 | -0.064 | 0.259 | 0.236 | 0.510 |
| n-6/n-3 | -0.103 | -0.273 | 0.066 | 0.232 | 0.329 | -0.095 | -0.262 | 0.072 | 0.264 | 0.529 |
| n-6/FA | -0.484 | -0.650 | -0.318 | <0.001 | <0.001 | -0.340 | -0.513 | -0.167 | <0.001 | 0.015 |
| PUFA/MUFA | -0.397 | -0.566 | -0.228 | <0.001 | <0.001 | -0.199 | -0.378 | -0.020 | 0.030 | 0.421 |
| PUFA/FA | -0.454 | -0.621 | -0.288 | <0.001 | <0.001 | -0.287 | -0.463 | -0.112 | 0.001 | 0.066 |
| SFA/FA | 0.359 | 0.192 | 0.526 | <0.001 | <0.001 | 0.291 | 0.125 | 0.457 | 0.001 | 0.037 |
| **Free cholesterol** |  |  |  |  |  |  |  |  |  |  |
| VS-VLDL | -0.090 | -0.256 | 0.076 | 0.289 | 0.387 | -0.013 | -0.186 | 0.159 | 0.878 | 0.950 |
| S-VLDL | -0.089 | -0.255 | 0.076 | 0.291 | 0.387 | -0.039 | -0.211 | 0.133 | 0.659 | 0.847 |
| M-VLDL | -0.082 | -0.248 | 0.084 | 0.334 | 0.440 | -0.040 | -0.213 | 0.133 | 0.648 | 0.847 |
| L-VLDL | 0.202 | 0.030 | 0.373 | 0.021 | 0.048 | 0.101 | -0.077 | 0.278 | 0.268 | 0.529 |
| VL-VLDL | 0.204 | 0.034 | 0.374 | 0.019 | 0.044 | 0.107 | -0.070 | 0.284 | 0.235 | 0.510 |
| XL-VLDL | 0.264 | 0.096 | 0.432 | 0.002 | 0.008 | 0.152 | -0.020 | 0.325 | 0.083 | 0.421 |
| VLDL | 0.066 | -0.102 | 0.233 | 0.441 | 0.549 | 0.044 | -0.130 | 0.218 | 0.623 | 0.836 |
| S-LDL | -0.165 | -0.329 | <0.001 | 0.050 | 0.095 | -0.074 | -0.243 | 0.095 | 0.392 | 0.668 |
| M-LDL | -0.117 | -0.282 | 0.047 | 0.163 | 0.250 | -0.037 | -0.206 | 0.132 | 0.664 | 0.847 |
| L-LDL | -0.186 | -0.351 | -0.020 | 0.028 | 0.059 | -0.064 | -0.237 | 0.108 | 0.464 | 0.749 |
| LDL | -0.166 | -0.331 | -0.001 | 0.048 | 0.093 | -0.058 | -0.229 | 0.112 | 0.502 | 0.763 |
| IDL | -0.205 | -0.373 | -0.037 | 0.017 | 0.041 | -0.049 | -0.227 | 0.129 | 0.591 | 0.817 |
| S-HDL | 0.157 | -0.014 | 0.328 | 0.071 | 0.129 | 0.140 | -0.028 | 0.308 | 0.102 | 0.421 |
| M-HDL | -0.015 | -0.203 | 0.172 | 0.872 | 0.924 | 0.071 | -0.116 | 0.258 | 0.457 | 0.743 |
| L-HDL | -0.309 | -0.506 | -0.112 | 0.002 | 0.008 | -0.116 | -0.323 | 0.091 | 0.271 | 0.529 |
| XL-HDL | -0.363 | -0.552 | -0.174 | <0.001 | 0.001 | -0.162 | -0.360 | 0.036 | 0.109 | 0.421 |
| HDL | -0.154 | -0.348 | 0.040 | 0.120 | 0.193 | -0.001 | -0.198 | 0.195 | 0.990 | 0.999 |
| Total | -0.135 | -0.303 | 0.033 | 0.114 | 0.187 | -0.033 | -0.209 | 0.142 | 0.709 | 0.849 |
| **Free cholesterol to Total lipids** |  |  |  |  |  |  |  |  |  |  |
| VS-VLDL | -0.224 | -0.388 | -0.059 | 0.008 | 0.021 | -0.102 | -0.268 | 0.064 | 0.228 | 0.508 |
| S-VLDL | -0.334 | -0.503 | -0.166 | <0.001 | 0.001 | -0.140 | -0.316 | 0.036 | 0.119 | 0.421 |
| M-VLDL | -0.388 | -0.560 | -0.215 | <0.001 | <0.001 | -0.152 | -0.335 | 0.031 | 0.104 | 0.421 |
| L-VLDL | 0.049 | -0.130 | 0.229 | 0.590 | 0.693 | 0.055 | -0.122 | 0.231 | 0.543 | 0.795 |
| XL-VLDL | -0.329 | -0.502 | -0.157 | <0.001 | 0.001 | -0.171 | -0.346 | 0.005 | 0.056 | 0.421 |
| XXL-VLDL | -0.065 | -0.229 | 0.099 | 0.438 | 0.549 | 0.032 | -0.132 | 0.197 | 0.700 | 0.848 |
| S-LDL | -0.315 | -0.483 | -0.147 | <0.001 | 0.002 | -0.169 | -0.338 | 0.001 | 0.051 | 0.421 |
| M-LDL | -0.357 | -0.526 | -0.187 | <0.001 | <0.001 | -0.188 | -0.363 | -0.013 | 0.035 | 0.421 |
| L-LDL | -0.378 | -0.552 | -0.204 | <0.001 | <0.001 | -0.165 | -0.349 | 0.018 | 0.077 | 0.421 |
| IDL | -0.195 | -0.361 | -0.030 | 0.021 | 0.048 | -0.036 | -0.201 | 0.128 | 0.667 | 0.847 |
| S-HDL | -0.321 | -0.500 | -0.142 | <0.001 | 0.002 | -0.144 | -0.325 | 0.038 | 0.122 | 0.421 |
| M-HDL | -0.273 | -0.465 | -0.081 | 0.005 | 0.016 | -0.047 | -0.246 | 0.153 | 0.648 | 0.847 |
| L-HDL | -0.438 | -0.634 | -0.241 | <0.001 | <0.001 | -0.138 | -0.347 | 0.071 | 0.195 | 0.486 |
| XL-HDL | 0.430 | 0.222 | 0.639 | <0.001 | 0.001 | 0.182 | -0.038 | 0.401 | 0.105 | 0.421 |
| **KB, FB & Inflammation** |  |  |  |  |  |  |  |  |  |  |
| Glycoprotein acetyls | 0.345 | 0.179 | 0.510 | <0.001 | <0.001 | 0.263 | 0.090 | 0.436 | 0.003 | 0.123 |
| 3-Hydrocybutyrate | 0.125 | -0.043 | 0.293 | 0.145 | 0.226 | 0.104 | -0.058 | 0.265 | 0.207 | 0.491 |
| Acetate | 0.029 | -0.185 | 0.243 | 0.792 | 0.875 | 0.053 | -0.151 | 0.257 | 0.608 | 0.832 |
| Acetoacetate | 0.169 | -0.001 | 0.339 | 0.052 | 0.098 | 0.117 | -0.046 | 0.280 | 0.160 | 0.458 |
| Acetone | 0.133 | -0.032 | 0.299 | 0.114 | 0.187 | 0.110 | -0.049 | 0.269 | 0.175 | 0.465 |
| Albumin | -0.102 | -0.268 | 0.065 | 0.230 | 0.328 | -0.068 | -0.232 | 0.095 | 0.412 | 0.683 |
| Creatinine | -0.077 | -0.273 | 0.119 | 0.441 | 0.549 | -0.113 | -0.304 | 0.079 | 0.250 | 0.522 |
| **Lipoprotein particles** |  |  |  |  |  |  |  |  |  |  |
| VS-VLDL | -0.066 | -0.231 | 0.100 | 0.437 | 0.549 | -0.003 | -0.173 | 0.168 | 0.977 | 0.997 |
| S-VLDL | 0.070 | -0.097 | 0.237 | 0.409 | 0.531 | 0.033 | -0.137 | 0.203 | 0.704 | 0.848 |
| M-VLDL | -0.007 | -0.174 | 0.160 | 0.933 | 0.952 | <0.001 | -0.173 | 0.173 | 1.000 | 1.000 |
| L-VLDL | 0.216 | 0.047 | 0.386 | 0.013 | 0.032 | 0.108 | -0.068 | 0.283 | 0.228 | 0.508 |
| VL-VLDL | 0.251 | 0.081 | 0.420 | 0.004 | 0.012 | 0.131 | -0.045 | 0.307 | 0.144 | 0.450 |
| XL-VLDL | 0.277 | 0.109 | 0.444 | 0.001 | 0.005 | 0.157 | -0.015 | 0.329 | 0.074 | 0.421 |
| VLDL | 0.045 | -0.120 | 0.211 | 0.592 | 0.693 | 0.033 | -0.138 | 0.205 | 0.702 | 0.848 |
| S-LDL | -0.041 | -0.206 | 0.123 | 0.623 | 0.718 | 0.001 | -0.171 | 0.173 | 0.991 | 0.999 |
| M-LDL | -0.009 | -0.175 | 0.157 | 0.916 | 0.943 | 0.035 | -0.137 | 0.207 | 0.690 | 0.848 |
| L-LDL | -0.145 | -0.309 | 0.019 | 0.083 | 0.144 | -0.072 | -0.243 | 0.098 | 0.406 | 0.683 |
| LDL | -0.099 | -0.264 | 0.065 | 0.238 | 0.333 | -0.036 | -0.208 | 0.136 | 0.679 | 0.848 |
| IDL | -0.162 | -0.327 | 0.002 | 0.053 | 0.101 | -0.057 | -0.232 | 0.118 | 0.524 | 0.772 |
| S-HDL | 0.197 | 0.026 | 0.368 | 0.024 | 0.052 | 0.149 | -0.020 | 0.318 | 0.083 | 0.421 |
| M-HDL | 0.018 | -0.168 | 0.203 | 0.852 | 0.911 | 0.083 | -0.100 | 0.266 | 0.374 | 0.645 |
| L-HDL | -0.322 | -0.519 | -0.126 | 0.001 | 0.006 | -0.136 | -0.343 | 0.071 | 0.197 | 0.486 |
| XL-HDL | -0.376 | -0.569 | -0.182 | <0.001 | 0.001 | -0.172 | -0.377 | 0.032 | 0.097 | 0.421 |
| HDL | 0.026 | -0.155 | 0.207 | 0.778 | 0.865 | 0.083 | -0.095 | 0.262 | 0.359 | 0.630 |
| Total | 0.007 | -0.173 | 0.187 | 0.942 | 0.957 | 0.074 | -0.104 | 0.252 | 0.414 | 0.683 |
| **Phospholipids** |  |  |  |  |  |  |  |  |  |  |
| VS-VLDL | -0.038 | -0.203 | 0.127 | 0.649 | 0.738 | 0.010 | -0.159 | 0.179 | 0.907 | 0.969 |
| S-VLDL | -0.017 | -0.183 | 0.150 | 0.845 | 0.911 | -0.002 | -0.173 | 0.170 | 0.986 | 0.999 |
| M-VLDL | -0.028 | -0.194 | 0.139 | 0.745 | 0.835 | -0.013 | -0.186 | 0.161 | 0.887 | 0.956 |
| L-VLDL | 0.217 | 0.047 | 0.388 | 0.012 | 0.032 | 0.105 | -0.072 | 0.282 | 0.243 | 0.518 |
| VL-VLDL | 0.228 | 0.057 | 0.398 | 0.009 | 0.023 | 0.120 | -0.057 | 0.297 | 0.184 | 0.473 |
| XL-VLDL | 0.279 | 0.111 | 0.448 | 0.001 | 0.005 | 0.161 | -0.012 | 0.333 | 0.068 | 0.421 |
| VLDL | 0.108 | -0.059 | 0.275 | 0.204 | 0.298 | 0.064 | -0.109 | 0.238 | 0.466 | 0.749 |
| S-LDL | -0.079 | -0.244 | 0.085 | 0.344 | 0.451 | -0.024 | -0.195 | 0.146 | 0.779 | 0.897 |
| M-LDL | -0.014 | -0.180 | 0.152 | 0.866 | 0.922 | 0.021 | -0.150 | 0.192 | 0.809 | 0.904 |
| L-LDL | -0.115 | -0.281 | 0.051 | 0.174 | 0.265 | -0.021 | -0.194 | 0.151 | 0.809 | 0.904 |
| LDL | -0.082 | -0.247 | 0.084 | 0.333 | 0.440 | -0.009 | -0.181 | 0.163 | 0.917 | 0.969 |
| IDL | -0.195 | -0.364 | -0.027 | 0.023 | 0.051 | -0.063 | -0.241 | 0.115 | 0.487 | 0.756 |
| S-HDL | 0.288 | 0.117 | 0.458 | 0.001 | 0.004 | 0.210 | 0.042 | 0.379 | 0.014 | 0.326 |
| M-HDL | 0.122 | -0.060 | 0.303 | 0.188 | 0.282 | 0.139 | -0.038 | 0.317 | 0.124 | 0.421 |
| L-HDL | -0.239 | -0.436 | -0.042 | 0.017 | 0.041 | -0.071 | -0.275 | 0.132 | 0.492 | 0.756 |
| XL-HDL | -0.361 | -0.555 | -0.166 | <0.001 | 0.002 | -0.163 | -0.367 | 0.041 | 0.118 | 0.421 |
| HDL | -0.011 | -0.200 | 0.178 | 0.906 | 0.940 | 0.075 | -0.112 | 0.262 | 0.433 | 0.709 |
| Total | -0.018 | -0.191 | 0.156 | 0.843 | 0.911 | 0.059 | -0.116 | 0.234 | 0.509 | 0.768 |
| **Phospholipids to Total lipids** |  |  |  |  |  |  |  |  |  |  |
| VS-VLDL | 0.115 | -0.056 | 0.286 | 0.186 | 0.281 | 0.007 | -0.164 | 0.178 | 0.938 | 0.973 |
| S-VLDL | -0.321 | -0.487 | -0.154 | <0.001 | 0.001 | -0.136 | -0.309 | 0.037 | 0.124 | 0.421 |
| M-VLDL | -0.372 | -0.547 | -0.197 | <0.001 | <0.001 | -0.144 | -0.330 | 0.042 | 0.129 | 0.423 |
| L-VLDL | 0.137 | -0.045 | 0.318 | 0.140 | 0.220 | 0.046 | -0.138 | 0.229 | 0.626 | 0.836 |
| VL-VLDL | -0.125 | -0.313 | 0.064 | 0.195 | 0.290 | -0.099 | -0.287 | 0.090 | 0.305 | 0.562 |
| XL-VLDL | 0.149 | -0.022 | 0.319 | 0.087 | 0.150 | 0.100 | -0.069 | 0.268 | 0.247 | 0.520 |
| S-LDL | -0.189 | -0.371 | -0.007 | 0.042 | 0.085 | -0.128 | -0.310 | 0.053 | 0.166 | 0.459 |
| M-LDL | -0.153 | -0.321 | 0.015 | 0.074 | 0.133 | -0.094 | -0.257 | 0.069 | 0.260 | 0.529 |
| L-LDL | 0.020 | -0.157 | 0.196 | 0.829 | 0.901 | 0.052 | -0.120 | 0.223 | 0.554 | 0.802 |
| IDL | -0.056 | -0.232 | 0.121 | 0.537 | 0.646 | -0.108 | -0.281 | 0.065 | 0.219 | 0.503 |
| S-HDL | 0.144 | -0.027 | 0.315 | 0.099 | 0.166 | 0.117 | -0.049 | 0.283 | 0.168 | 0.459 |
| M-HDL | 0.319 | 0.145 | 0.493 | <0.001 | 0.002 | 0.168 | -0.014 | 0.351 | 0.071 | 0.421 |
| L-HDL | 0.572 | 0.396 | 0.747 | <0.001 | <0.001 | 0.353 | 0.159 | 0.548 | <0.001 | 0.031 |
| XL-HDL | -0.305 | -0.501 | -0.108 | 0.002 | 0.008 | -0.129 | -0.329 | 0.070 | 0.204 | 0.491 |
| **Total lipids** |  |  |  |  |  |  |  |  |  |  |
| VS-VLDL | -0.056 | -0.221 | 0.110 | 0.512 | 0.619 | 0.007 | -0.163 | 0.178 | 0.932 | 0.971 |
| S-VLDL | 0.068 | -0.098 | 0.235 | 0.421 | 0.538 | 0.038 | -0.133 | 0.209 | 0.663 | 0.847 |
| M-VLDL | 0.042 | -0.125 | 0.210 | 0.621 | 0.718 | 0.019 | -0.154 | 0.192 | 0.828 | 0.921 |
| L-VLDL | 0.210 | 0.039 | 0.380 | 0.016 | 0.040 | 0.098 | -0.078 | 0.275 | 0.273 | 0.529 |
| VL-VLDL | 0.256 | 0.086 | 0.426 | 0.003 | 0.011 | 0.137 | -0.040 | 0.313 | 0.129 | 0.423 |
| XL-VLDL | 0.270 | 0.102 | 0.438 | 0.002 | 0.007 | 0.151 | -0.022 | 0.323 | 0.086 | 0.421 |
| VLDL | 0.158 | -0.010 | 0.326 | 0.065 | 0.119 | 0.087 | -0.086 | 0.261 | 0.324 | 0.590 |
| S-LDL | -0.036 | -0.201 | 0.129 | 0.668 | 0.756 | 0.005 | -0.167 | 0.177 | 0.956 | 0.988 |
| M-LDL | 0.005 | -0.161 | 0.172 | 0.953 | 0.964 | 0.034 | -0.138 | 0.206 | 0.697 | 0.848 |
| L-LDL | -0.113 | -0.278 | 0.053 | 0.181 | 0.276 | -0.028 | -0.199 | 0.143 | 0.750 | 0.885 |
| LDL | -0.073 | -0.238 | 0.092 | 0.385 | 0.502 | -0.008 | -0.179 | 0.163 | 0.931 | 0.971 |
| IDL | -0.179 | -0.348 | -0.011 | 0.037 | 0.076 | -0.045 | -0.223 | 0.134 | 0.623 | 0.836 |
| S-HDL | 0.272 | 0.101 | 0.442 | 0.002 | 0.007 | 0.199 | 0.030 | 0.367 | 0.021 | 0.421 |
| M-HDL | 0.073 | -0.110 | 0.257 | 0.434 | 0.549 | 0.114 | -0.067 | 0.294 | 0.217 | 0.503 |
| L-HDL | -0.288 | -0.484 | -0.091 | 0.004 | 0.013 | -0.106 | -0.312 | 0.100 | 0.311 | 0.570 |
| XL-HDL | -0.375 | -0.569 | -0.180 | <0.001 | 0.001 | -0.170 | -0.375 | 0.035 | 0.104 | 0.421 |
| HDL | -0.072 | -0.263 | 0.119 | 0.459 | 0.565 | 0.042 | -0.150 | 0.233 | 0.670 | 0.847 |
| Total | 0.003 | -0.164 | 0.170 | 0.976 | 0.976 | 0.048 | -0.123 | 0.219 | 0.585 | 0.817 |
| **Triglycerides** |  |  |  |  |  |  |  |  |  |  |
| VS-VLDL | 0.182 | 0.022 | 0.343 | 0.026 | 0.056 | 0.109 | -0.052 | 0.271 | 0.184 | 0.473 |
| S-VLDL | 0.193 | 0.028 | 0.358 | 0.022 | 0.049 | 0.091 | -0.076 | 0.258 | 0.285 | 0.541 |
| M-VLDL | 0.195 | 0.026 | 0.363 | 0.024 | 0.052 | 0.091 | -0.081 | 0.264 | 0.299 | 0.560 |
| L-VLDL | 0.237 | 0.066 | 0.407 | 0.006 | 0.019 | 0.110 | -0.066 | 0.285 | 0.220 | 0.503 |
| XL-VLDL | 0.292 | 0.122 | 0.461 | 0.001 | 0.004 | 0.156 | -0.020 | 0.331 | 0.082 | 0.421 |
| XXL-VLDL | 0.267 | 0.100 | 0.435 | 0.002 | 0.007 | 0.144 | -0.028 | 0.315 | 0.101 | 0.421 |
| VLDL | 0.246 | 0.078 | 0.414 | 0.004 | 0.013 | 0.125 | -0.047 | 0.298 | 0.155 | 0.458 |
| S-LDL | 0.221 | 0.060 | 0.383 | 0.007 | 0.020 | 0.132 | -0.033 | 0.296 | 0.116 | 0.421 |
| M-LDL | 0.224 | 0.064 | 0.384 | 0.006 | 0.018 | 0.143 | -0.019 | 0.305 | 0.084 | 0.421 |
| L-LDL | 0.207 | 0.047 | 0.366 | 0.011 | 0.030 | 0.145 | -0.016 | 0.306 | 0.078 | 0.421 |
| LDL | 0.215 | 0.055 | 0.374 | 0.008 | 0.023 | 0.144 | -0.017 | 0.306 | 0.080 | 0.421 |
| IDL | 0.170 | 0.009 | 0.330 | 0.038 | 0.077 | 0.116 | -0.045 | 0.277 | 0.158 | 0.458 |
| S-HDL | 0.299 | 0.132 | 0.466 | <0.001 | 0.002 | 0.174 | 0.002 | 0.346 | 0.048 | 0.421 |
| M-HDL | 0.229 | 0.066 | 0.392 | 0.006 | 0.018 | 0.142 | -0.020 | 0.304 | 0.085 | 0.421 |
| L-HDL | 0.016 | -0.153 | 0.185 | 0.849 | 0.911 | 0.031 | -0.131 | 0.194 | 0.705 | 0.848 |
| XL-HDL | 0.039 | -0.122 | 0.201 | 0.634 | 0.728 | 0.032 | -0.126 | 0.190 | 0.690 | 0.848 |
| HDL | 0.202 | 0.040 | 0.364 | 0.015 | 0.037 | 0.122 | -0.038 | 0.283 | 0.136 | 0.427 |
| Total | 0.243 | 0.077 | 0.409 | 0.004 | 0.013 | 0.130 | -0.040 | 0.300 | 0.133 | 0.427 |
| **Triglycerides to Total lipids** |  |  |  |  |  |  |  |  |  |  |
| VS-VLDL | 0.385 | 0.216 | 0.554 | <0.001 | <0.001 | 0.167 | -0.014 | 0.348 | 0.070 | 0.421 |
| S-VLDL | 0.310 | 0.144 | 0.476 | <0.001 | 0.002 | 0.117 | -0.055 | 0.289 | 0.181 | 0.473 |
| M-VLDL | 0.421 | 0.248 | 0.594 | <0.001 | <0.001 | 0.181 | -0.003 | 0.366 | 0.054 | 0.421 |
| L-VLDL | 0.127 | -0.052 | 0.306 | 0.163 | 0.250 | 0.049 | -0.126 | 0.225 | 0.581 | 0.817 |
| XL-VLDL | 0.330 | 0.159 | 0.502 | <0.001 | 0.001 | 0.167 | -0.008 | 0.342 | 0.061 | 0.421 |
| XXL-VLDL | 0.021 | -0.141 | 0.184 | 0.796 | 0.875 | -0.040 | -0.199 | 0.119 | 0.623 | 0.836 |
| S-LDL | 0.317 | 0.147 | 0.486 | <0.001 | 0.002 | 0.155 | -0.017 | 0.327 | 0.077 | 0.421 |
| M-LDL | 0.291 | 0.123 | 0.460 | 0.001 | 0.004 | 0.142 | -0.029 | 0.314 | 0.103 | 0.421 |
| L-LDL | 0.356 | 0.186 | 0.526 | <0.001 | <0.001 | 0.196 | 0.021 | 0.371 | 0.028 | 0.421 |
| IDL | 0.352 | 0.185 | 0.520 | <0.001 | <0.001 | 0.163 | -0.013 | 0.339 | 0.070 | 0.421 |
| S-HDL | 0.240 | 0.069 | 0.410 | 0.006 | 0.017 | 0.113 | -0.062 | 0.288 | 0.207 | 0.491 |
| M-HDL | 0.200 | 0.030 | 0.369 | 0.021 | 0.048 | 0.092 | -0.081 | 0.265 | 0.297 | 0.560 |
| L-HDL | 0.247 | 0.065 | 0.429 | 0.008 | 0.021 | 0.104 | -0.082 | 0.289 | 0.274 | 0.529 |
| XL-HDL | 0.337 | 0.136 | 0.537 | 0.001 | 0.005 | 0.162 | -0.045 | 0.369 | 0.125 | 0.421 |

† Model adjusted for age, sex, and education.
‡ Model adjusted for age, sex, education, race, socioeconomic status, body mass index, smoking status, alcohol drinking status, physical activity, social connection, hypertension, diabetes, heart disease, beta-blockers, calcium blockers, lipid-lowering, and APOE ε4 status.
Abbreviation: Apo, apolipoprotein; LDL-AD, average diameter for LDL particles; CHOL, cholesterol; CE, cholesteryl esters; TG, triglycerides; S, small; M, medium; L, large; VL, very large; XL, extremely large; VLDL, very low-density lipoprotein; LDL; low-density lipoprotein; IDL, intermediate-density lipoprotein; HDL, high-density lipoprotein; LA, linoleic acid; MUFA, monounsaturated fatty acid; n-6, omega-6 fatty acid; PUFA, polyunsaturated fatty acid; SFA, saturated fatty acid; FA, fatty acid.

**Table S13.** The interaction effect of metabolites with *APOE* ε4 status on brain age gap (BAG)

| **Metabolites** | **Estimate** | **SE** | **Statistic** | ***P-value*** | **FDR-q** |
| --- | --- | --- | --- | --- | --- |
| **Amino acids & Glycolysis** | 1.197 | 2.224 | 0.538 | 0.591 | 0.998 |
| Valine | -2.252 | 3.303 | -0.682 | 0.495 | 0.998 |
| Leucine | -2.122 | 5.259 | -0.404 | 0.687 | 0.998 |
| Isoleucine | -0.442 | 8.683 | -0.051 | 0.959 | 0.998 |
| Phenylalanine | 0.713 | 6.877 | 0.104 | 0.917 | 0.998 |
| Tyrosine | 1.779 | 1.239 | 1.436 | 0.151 | 0.998 |
| Alanine | 0.679 | 1.139 | 0.596 | 0.551 | 0.998 |
| Glutamine | -1.540 | 1.381 | -1.115 | 0.265 | 0.998 |
| Glycine | -16.472 | 8.862 | -1.859 | 0.063 | 0.998 |
| Histidine | -0.049 | 1.102 | -0.045 | 0.964 | 0.998 |
| BCAAs | -0.103 | 0.088 | -1.163 | 0.245 | 0.998 |
| Lactate | -0.444 | 3.368 | -0.132 | 0.895 | 0.998 |
| Pyruvate | 0.102 | 0.105 | 0.969 | 0.333 | 0.998 |
| Glucose | -2.786 | 7.416 | -0.376 | 0.707 | 0.998 |
| Citrate |  |  |  |  |  |
| **Apo-LP, LP size & Other lipids** |  |  |  |  |  |
| ApoA1 | -0.280 | 0.406 | -0.689 | 0.491 | 0.998 |
| ApoB | 0.259 | 0.492 | 0.526 | 0.599 | 0.998 |
| ApoB/ApoA1 | 0.432 | 0.582 | 0.743 | 0.457 | 0.998 |
| HDL-AD | -0.166 | 0.471 | -0.353 | 0.724 | 0.998 |
| LDL-AD | -0.966 | 1.100 | -0.879 | 0.380 | 0.998 |
| VLDL-AD | 0.006 | 0.081 | 0.070 | 0.944 | 0.998 |
| Phosphatidylcholine | -0.076 | 0.263 | -0.290 | 0.772 | 0.998 |
| Phosphoglycerides | -0.052 | 0.247 | -0.209 | 0.835 | 0.998 |
| Sphingomyeline | -0.036 | 1.374 | -0.026 | 0.979 | 0.998 |
| Totalcholine | -0.071 | 0.240 | -0.296 | 0.767 | 0.998 |
| TG/Phosphoglycerides | 0.177 | 0.434 | 0.408 | 0.683 | 0.998 |
| **Cholesterol** |  |  |  |  |  |
| XS-VLDL | 1.335 | 1.998 | 0.668 | 0.504 | 0.998 |
| S-VLDL | 1.413 | 1.904 | 0.742 | 0.458 | 0.998 |
| M-VLDL | 0.781 | 1.507 | 0.518 | 0.604 | 0.998 |
| L-VLDL | 1.374 | 2.036 | 0.675 | 0.500 | 0.998 |
| VL-VLDL | 1.970 | 3.422 | 0.576 | 0.565 | 0.998 |
| XL-VLDL | 0.888 | 2.350 | 0.378 | 0.705 | 0.998 |
| VLDL | 0.275 | 0.401 | 0.687 | 0.492 | 0.998 |
| S-LDL | 0.906 | 2.212 | 0.409 | 0.682 | 0.998 |
| M-LDL | 0.313 | 0.828 | 0.379 | 0.705 | 0.998 |
| L-LDL | 0.050 | 0.345 | 0.146 | 0.884 | 0.998 |
| Clinical-LDL | 0.032 | 0.135 | 0.240 | 0.811 | 0.998 |
| LDL | 0.052 | 0.223 | 0.235 | 0.814 | 0.998 |
| IDL | 0.046 | 0.465 | 0.099 | 0.921 | 0.998 |
| S-HDL | -0.819 | 1.609 | -0.509 | 0.611 | 0.998 |
| M-HDL | -0.670 | 0.806 | -0.832 | 0.405 | 0.998 |
| L-HDL | -0.315 | 0.582 | -0.541 | 0.589 | 0.998 |
| XL-HDL | -0.703 | 2.867 | -0.245 | 0.806 | 0.998 |
| HDL | -0.209 | 0.302 | -0.691 | 0.489 | 0.998 |
| Total | 0.004 | 0.106 | 0.036 | 0.971 | 0.998 |
| Remnant | 0.110 | 0.238 | 0.462 | 0.644 | 0.998 |
| Total-HDLC | 0.041 | 0.117 | 0.352 | 0.725 | 0.998 |
| **Cholesterol to Total lipids** |  |  |  |  |  |
| VS-VLDL | 0.006 | 0.022 | 0.297 | 0.767 | 0.998 |
| S-VLDL | 0.010 | 0.021 | 0.476 | 0.634 | 0.998 |
| M-VLDL | 0.000 | 0.015 | 0.012 | 0.990 | 0.998 |
| L-VLDL | 0.014 | 0.024 | 0.567 | 0.571 | 0.998 |
| XL-VLDL | -0.003 | 0.013 | -0.261 | 0.794 | 0.998 |
| XXL-VLDL | 0.004 | 0.007 | 0.537 | 0.591 | 0.998 |
| S-LDL | -0.002 | 0.054 | -0.040 | 0.968 | 0.998 |
| M-LDL | 0.015 | 0.068 | 0.218 | 0.828 | 0.998 |
| L-LDL | -0.047 | 0.060 | -0.776 | 0.438 | 0.998 |
| IDL | -0.013 | 0.036 | -0.364 | 0.716 | 0.998 |
| S-HDL | -0.019 | 0.052 | -0.374 | 0.709 | 0.998 |
| M-HDL | -0.021 | 0.033 | -0.640 | 0.522 | 0.998 |
| L-HDL | -0.010 | 0.019 | -0.503 | 0.615 | 0.998 |
| XL-HDL | -0.004 | 0.020 | -0.198 | 0.843 | 0.998 |
| **Cholesteryl esters to Total lipids** |  |  |  |  |  |
| VS-VLDL | 0.004 | 0.024 | 0.156 | 0.876 | 0.998 |
| S-VLDL | 0.027 | 0.036 | 0.740 | 0.460 | 0.998 |
| M-VLDL | -0.002 | 0.020 | -0.105 | 0.916 | 0.998 |
| L-VLDL | 0.013 | 0.030 | 0.433 | 0.665 | 0.998 |
| XL-VLDL | -0.004 | 0.016 | -0.260 | 0.794 | 0.998 |
| XXL-VLDL | 0.004 | 0.009 | 0.410 | 0.682 | 0.998 |
| S-LDL | 0.030 | 0.050 | 0.599 | 0.549 | 0.998 |
| M-LDL | 0.037 | 0.054 | 0.682 | 0.495 | 0.998 |
| L-LDL | -0.062 | 0.084 | -0.740 | 0.459 | 0.998 |
| IDL | -0.042 | 0.044 | -0.974 | 0.330 | 0.998 |
| S-HDL | -0.022 | 0.052 | -0.433 | 0.665 | 0.998 |
| M-HDL | -0.025 | 0.038 | -0.651 | 0.515 | 0.998 |
| L-HDL | -0.010 | 0.022 | -0.431 | 0.666 | 0.998 |
| XL-HDL | -0.027 | 0.031 | -0.886 | 0.376 | 0.998 |
| **Cholestryl esters** |  |  |  |  |  |
| VS-VLDL | 1.668 | 2.807 | 0.594 | 0.552 | 0.998 |
| S-VLDL | 2.386 | 2.961 | 0.806 | 0.420 | 0.998 |
| M-VLDL | 1.115 | 2.590 | 0.430 | 0.667 | 0.998 |
| L-VLDL | 3.220 | 4.167 | 0.773 | 0.440 | 0.998 |
| VL-VLDL | 4.163 | 6.642 | 0.627 | 0.531 | 0.998 |
| XL-VLDL | 1.781 | 4.231 | 0.421 | 0.674 | 0.998 |
| VLDL | 0.481 | 0.692 | 0.695 | 0.487 | 0.998 |
| S-LDL | 1.421 | 2.952 | 0.481 | 0.630 | 0.998 |
| M-LDL | 0.483 | 1.108 | 0.436 | 0.663 | 0.998 |
| L-LDL | 0.072 | 0.467 | 0.154 | 0.878 | 0.998 |
| LDL | 0.080 | 0.303 | 0.265 | 0.791 | 0.998 |
| IDL | -0.014 | 0.625 | -0.022 | 0.982 | 0.998 |
| S-HDL | -1.185 | 2.078 | -0.570 | 0.569 | 0.998 |
| M-HDL | -0.875 | 1.004 | -0.871 | 0.384 | 0.998 |
| L-HDL | -0.425 | 0.748 | -0.567 | 0.570 | 0.998 |
| XL-HDL | -1.084 | 3.577 | -0.303 | 0.762 | 0.998 |
| HDL | -0.289 | 0.386 | -0.747 | 0.455 | 0.998 |
| Total | -0.004 | 0.147 | -0.027 | 0.978 | 0.998 |
| **Fatty acids** |  |  |  |  |  |
| DHA | 2.446 | 1.133 | 2.159 | 0.031 | 0.998 |
| LA | -0.048 | 0.143 | -0.336 | 0.737 | 0.998 |
| MUFA | 0.005 | 0.119 | 0.042 | 0.966 | 0.998 |
| n-3 | 0.820 | 0.426 | 1.924 | 0.054 | 0.998 |
| n-6 | -0.033 | 0.144 | -0.231 | 0.818 | 0.998 |
| PUFA | 0.043 | 0.121 | 0.357 | 0.721 | 0.998 |
| SFA | 0.005 | 0.102 | 0.052 | 0.959 | 0.998 |
| Total | 0.008 | 0.041 | 0.188 | 0.851 | 0.998 |
| Unsaturation | 1.648 | 1.222 | 1.348 | 0.178 | 0.998 |
| DHA/FA | 0.358 | 0.143 | 2.508 | 0.012 | 0.998 |
| LA/FA | -0.027 | 0.029 | -0.942 | 0.346 | 0.998 |
| MUFA/FA | -0.019 | 0.038 | -0.492 | 0.623 | 0.998 |
| n-3/FA | 0.145 | 0.062 | 2.353 | 0.019 | 0.998 |
| n-6/n-3 | -0.053 | 0.023 | -2.271 | 0.023 | 0.998 |
| n-6/FA | -0.017 | 0.027 | -0.617 | 0.537 | 0.998 |
| PUFA/MUFA | 0.158 | 0.289 | 0.547 | 0.585 | 0.998 |
| PUFA/FA | 0.011 | 0.027 | 0.422 | 0.673 | 0.998 |
| SFA/FA | 0.001 | 0.050 | 0.022 | 0.982 | 0.998 |
| **Free cholesterol** |  |  |  |  |  |
| VS-VLDL | 5.425 | 6.624 | 0.819 | 0.413 | 0.998 |
| S-VLDL | 3.214 | 5.213 | 0.617 | 0.538 | 0.998 |
| M-VLDL | 2.070 | 3.334 | 0.621 | 0.535 | 0.998 |
| L-VLDL | 2.157 | 3.884 | 0.555 | 0.579 | 0.998 |
| VL-VLDL | 3.407 | 6.839 | 0.498 | 0.618 | 0.998 |
| XL-VLDL | 1.672 | 5.205 | 0.321 | 0.748 | 0.998 |
| VLDL | 0.598 | 0.918 | 0.652 | 0.514 | 0.998 |
| S-LDL | 1.475 | 7.828 | 0.188 | 0.851 | 0.998 |
| M-LDL | 0.616 | 3.018 | 0.204 | 0.838 | 0.998 |
| L-LDL | 0.155 | 1.279 | 0.121 | 0.903 | 0.998 |
| LDL | 0.125 | 0.815 | 0.153 | 0.879 | 0.998 |
| IDL | 0.781 | 1.766 | 0.443 | 0.658 | 0.998 |
| S-HDL | -1.922 | 6.038 | -0.318 | 0.750 | 0.998 |
| M-HDL | -2.655 | 3.988 | -0.666 | 0.506 | 0.998 |
| L-HDL | -1.151 | 2.599 | -0.443 | 0.658 | 0.998 |
| XL-HDL | -0.042 | 13.825 | -0.003 | 0.998 | 0.998 |
| HDL | -0.654 | 1.350 | -0.485 | 0.628 | 0.998 |
| Total | 0.073 | 0.369 | 0.197 | 0.844 | 0.998 |
| **Free cholesterol to Total lipids** |  |  |  |  |  |
| VS-VLDL | 0.182 | 0.167 | 1.093 | 0.275 | 0.998 |
| S-VLDL | 0.001 | 0.045 | 0.018 | 0.986 | 0.998 |
| M-VLDL | 0.020 | 0.059 | 0.341 | 0.733 | 0.998 |
| L-VLDL | 0.062 | 0.076 | 0.821 | 0.412 | 0.998 |
| XL-VLDL | -0.016 | 0.058 | -0.277 | 0.782 | 0.998 |
| XXL-VLDL | 0.013 | 0.020 | 0.680 | 0.496 | 0.998 |
| S-LDL | -0.024 | 0.052 | -0.453 | 0.650 | 0.998 |
| M-LDL | -0.016 | 0.053 | -0.302 | 0.762 | 0.998 |
| L-LDL | -0.020 | 0.080 | -0.253 | 0.800 | 0.998 |
| IDL | 0.103 | 0.090 | 1.144 | 0.252 | 0.998 |
| S-HDL | 0.018 | 0.191 | 0.097 | 0.923 | 0.998 |
| M-HDL | -0.027 | 0.143 | -0.188 | 0.851 | 0.998 |
| L-HDL | -0.030 | 0.094 | -0.315 | 0.753 | 0.998 |
| XL-HDL | 0.018 | 0.030 | 0.590 | 0.555 | 0.998 |
| **KB, FB & Inflammation** |  |  |  |  |  |
| Glycoprotein-acetyls | 0.899 | 0.830 | 1.082 | 0.279 | 0.998 |
| 3-Hydrocybutyrate | -0.713 | 1.628 | -0.438 | 0.661 | 0.998 |
| Acetate | 12.756 | 8.890 | 1.435 | 0.151 | 0.998 |
| Acetoacetate | -0.126 | 8.565 | -0.015 | 0.988 | 0.998 |
| Acetone | 2.452 | 18.641 | 0.132 | 0.895 | 0.998 |
| Albumin | -0.034 | 0.029 | -1.162 | 0.245 | 0.998 |
| Creatinine | -1.190 | 7.466 | -0.159 | 0.873 | 0.998 |
| **Lipoprotein particles** |  |  |  |  |  |
| VS-VLDL | 5269.593 | 7147.629 | 0.737 | 0.461 | 0.998 |
| S-VLDL | 5609.674 | 7438.964 | 0.754 | 0.451 | 0.998 |
| M-VLDL | 5258.731 | 7946.294 | 0.662 | 0.508 | 0.998 |
| L-VLDL | 8701.800 | 17361.710 | 0.501 | 0.616 | 0.998 |
| VL-VLDL | 16335.061 | 42723.132 | 0.382 | 0.702 | 0.998 |
| XL-VLDL | 14729.423 | 66063.559 | 0.223 | 0.824 | 0.998 |
| VLDL | 1590.694 | 2194.392 | 0.725 | 0.469 | 0.998 |
| S-LDL | 2165.106 | 2618.551 | 0.827 | 0.408 | 0.998 |
| M-LDL | 1025.032 | 1282.923 | 0.799 | 0.424 | 0.998 |
| L-LDL | 229.213 | 554.558 | 0.413 | 0.679 | 0.998 |
| LDL | 201.103 | 347.114 | 0.579 | 0.562 | 0.998 |
| IDL | 164.343 | 1301.769 | 0.126 | 0.900 | 0.998 |
| S-HDL | -31.083 | 75.271 | -0.413 | 0.680 | 0.998 |
| M-HDL | -80.506 | 106.116 | -0.759 | 0.448 | 0.998 |
| L-HDL | -68.073 | 127.133 | -0.535 | 0.592 | 0.998 |
| XL-HDL | -207.788 | 1049.982 | -0.198 | 0.843 | 0.998 |
| HDL | -28.790 | 40.809 | -0.705 | 0.481 | 0.998 |
| Total | -23.852 | 39.150 | -0.609 | 0.542 | 0.998 |
| **Phospholipids** |  |  |  |  |  |
| VS-VLDL | 3.092 | 3.690 | 0.838 | 0.402 | 0.998 |
| S-VLDL | 2.292 | 3.241 | 0.707 | 0.479 | 0.998 |
| M-VLDL | 1.194 | 2.058 | 0.580 | 0.562 | 0.998 |
| L-VLDL | 1.138 | 2.450 | 0.465 | 0.642 | 0.998 |
| VL-VLDL | 1.640 | 3.769 | 0.435 | 0.663 | 0.998 |
| XL-VLDL | 0.947 | 3.166 | 0.299 | 0.765 | 0.998 |
| VLDL | 0.335 | 0.542 | 0.619 | 0.536 | 0.998 |
| S-LDL | 2.743 | 5.210 | 0.527 | 0.598 | 0.998 |
| M-LDL | 0.730 | 2.300 | 0.317 | 0.751 | 0.998 |
| L-LDL | 0.277 | 1.194 | 0.232 | 0.816 | 0.998 |
| LDL | 0.209 | 0.697 | 0.300 | 0.764 | 0.998 |
| IDL | 0.508 | 1.497 | 0.340 | 0.734 | 0.998 |
| S-HDL | -0.457 | 1.069 | -0.428 | 0.669 | 0.998 |
| M-HDL | -0.692 | 0.982 | -0.704 | 0.481 | 0.998 |
| L-HDL | -0.333 | 0.640 | -0.521 | 0.602 | 0.998 |
| XL-HDL | -0.453 | 2.203 | -0.206 | 0.837 | 0.998 |
| HDL | -0.189 | 0.307 | -0.616 | 0.538 | 0.998 |
| Total | -0.021 | 0.210 | -0.101 | 0.920 | 0.998 |
| **Phospholipids to Total lipids** |  |  |  |  |  |
| VS-VLDL | 0.059 | 0.112 | 0.529 | 0.597 | 0.998 |
| S-VLDL | -0.001 | 0.048 | -0.023 | 0.982 | 0.998 |
| M-VLDL | 0.001 | 0.058 | 0.023 | 0.982 | 0.998 |
| L-VLDL | 0.010 | 0.031 | 0.315 | 0.753 | 0.998 |
| VL-VLDL | -0.006 | 0.038 | -0.156 | 0.876 | 0.998 |
| XL-VLDL | 0.021 | 0.021 | 1.001 | 0.317 | 0.998 |
| S-LDL | -0.017 | 0.059 | -0.293 | 0.769 | 0.998 |
| M-LDL | -0.070 | 0.121 | -0.576 | 0.564 | 0.998 |
| L-LDL | 0.056 | 0.132 | 0.428 | 0.669 | 0.998 |
| IDL | 0.035 | 0.114 | 0.310 | 0.757 | 0.998 |
| S-HDL | -0.021 | 0.077 | -0.269 | 0.788 | 0.998 |
| M-HDL | 0.044 | 0.079 | 0.561 | 0.575 | 0.998 |
| L-HDL | 0.007 | 0.036 | 0.190 | 0.849 | 0.998 |
| XL-HDL | 0.002 | 0.015 | 0.125 | 0.901 | 0.998 |
| **Total lipids** |  |  |  |  |  |
| VS-VLDL | 0.862 | 1.104 | 0.780 | 0.435 | 0.998 |
| S-VLDL | 0.556 | 0.737 | 0.754 | 0.451 | 0.998 |
| M-VLDL | 0.278 | 0.471 | 0.591 | 0.555 | 0.998 |
| L-VLDL | 0.243 | 0.544 | 0.447 | 0.655 | 0.998 |
| VL-VLDL | 0.286 | 0.728 | 0.392 | 0.695 | 0.998 |
| XL-VLDL | 0.050 | 0.491 | 0.101 | 0.920 | 0.998 |
| VLDL | 0.060 | 0.114 | 0.524 | 0.600 | 0.998 |
| S-LDL | 0.698 | 1.494 | 0.467 | 0.640 | 0.998 |
| M-LDL | 0.223 | 0.587 | 0.381 | 0.703 | 0.998 |
| L-LDL | 0.050 | 0.259 | 0.193 | 0.847 | 0.998 |
| LDL | 0.045 | 0.163 | 0.277 | 0.782 | 0.998 |
| IDL | 0.067 | 0.344 | 0.196 | 0.845 | 0.998 |
| S-HDL | -0.232 | 0.627 | -0.370 | 0.711 | 0.998 |
| M-HDL | -0.324 | 0.439 | -0.739 | 0.460 | 0.998 |
| L-HDL | -0.154 | 0.302 | -0.511 | 0.609 | 0.998 |
| XL-HDL | -0.255 | 1.243 | -0.205 | 0.837 | 0.998 |
| HDL | -0.095 | 0.153 | -0.617 | 0.537 | 0.998 |
| Total | 0.008 | 0.060 | 0.127 | 0.899 | 0.998 |
| **Triglycerides** |  |  |  |  |  |
| VS-VLDL | 2.711 | 4.464 | 0.607 | 0.544 | 0.998 |
| S-VLDL | 0.957 | 1.563 | 0.612 | 0.540 | 0.998 |
| M-VLDL | 0.426 | 0.820 | 0.519 | 0.604 | 0.998 |
| L-VLDL | 0.313 | 1.033 | 0.303 | 0.762 | 0.998 |
| XL-VLDL | 0.351 | 1.194 | 0.294 | 0.768 | 0.998 |
| XXL-VLDL | -0.026 | 0.763 | -0.034 | 0.973 | 0.998 |
| VLDL | 0.066 | 0.201 | 0.328 | 0.743 | 0.998 |
| S-LDL | 6.252 | 16.924 | 0.369 | 0.712 | 0.998 |
| M-LDL | 3.131 | 9.188 | 0.341 | 0.733 | 0.998 |
| L-LDL | 1.387 | 3.698 | 0.375 | 0.708 | 0.998 |
| LDL | 0.844 | 2.311 | 0.365 | 0.715 | 0.998 |
| IDL | 1.533 | 3.643 | 0.421 | 0.674 | 0.998 |
| S-HDL | 2.524 | 5.478 | 0.461 | 0.645 | 0.998 |
| M-HDL | 1.328 | 5.079 | 0.262 | 0.794 | 0.998 |
| L-HDL | 1.917 | 7.861 | 0.244 | 0.807 | 0.998 |
| XL-HDL | 16.286 | 35.807 | 0.455 | 0.649 | 0.998 |
| HDL | 0.702 | 2.029 | 0.346 | 0.729 | 0.998 |
| Total | 0.058 | 0.168 | 0.344 | 0.731 | 0.998 |
| **Triglycerides to Total lipids** |  |  |  |  |  |
| VS-VLDL | -0.012 | 0.024 | -0.484 | 0.628 | 0.998 |
| S-VLDL | -0.005 | 0.015 | -0.337 | 0.736 | 0.998 |
| M-VLDL | 0.000 | 0.012 | -0.018 | 0.985 | 0.998 |
| L-VLDL | -0.009 | 0.018 | -0.502 | 0.615 | 0.998 |
| XL-VLDL | 0.003 | 0.013 | 0.201 | 0.841 | 0.998 |
| XXL-VLDL | -0.005 | 0.006 | -0.803 | 0.422 | 0.998 |
| S-LDL | 0.006 | 0.059 | 0.107 | 0.915 | 0.998 |
| M-LDL | -0.001 | 0.071 | -0.007 | 0.994 | 0.998 |
| L-LDL | 0.026 | 0.060 | 0.432 | 0.666 | 0.998 |
| IDL | 0.006 | 0.043 | 0.148 | 0.882 | 0.998 |
| S-HDL | 0.060 | 0.074 | 0.819 | 0.413 | 0.998 |
| M-HDL | 0.035 | 0.053 | 0.661 | 0.509 | 0.998 |
| L-HDL | 0.024 | 0.034 | 0.698 | 0.485 | 0.998 |
| XL-HDL | 0.002 | 0.032 | 0.073 | 0.942 | 0.998 |

Model adjusted for age, sex, education, race, socioeconomic status, body mass index, smoking status, alcohol drinking status, physical activity, social connection, hypertension, diabetes, heart disease, beta-blockers, calcium blockers, and lipid-lowering

**Table S14.** β coefficients and 95% confidence intervals (CIs) for the association between metabolites and brain age after multiple imputation of covariates: results from linear regression models

| **Metabolites** | **Basic-model ^†^** | |  |  |  | **Multi-model ^‡^** | |  |  |  |
| --- | --- | --- | --- | --- | --- | --- | --- | --- | --- | --- |
|  | **β** | **Lower** | **Upper** | ***P*-value** | **FDR-q** | **β** | **Lower** | **Upper** | ***P*-value** | **FDR-q** |
| **Amino acids & Glycolysis** |  |  |  |  |  |  |  |  |  |  |
| Valine | 0.142 | 0.056 | 0.227 | 0.001 | 0.002 | 0.039 | -0.048 | 0.126 | 0.383 | 0.557 |
| Leucine | 0.119 | 0.034 | 0.205 | 0.006 | 0.011 | 0.037 | -0.050 | 0.124 | 0.405 | 0.576 |
| Isoleucine | 0.073 | -0.012 | 0.157 | 0.091 | 0.116 | 0.004 | -0.081 | 0.089 | 0.924 | 0.962 |
| Phenylalanine | 0.111 | 0.029 | 0.194 | 0.008 | 0.013 | 0.059 | -0.024 | 0.141 | 0.163 | 0.311 |
| Tyrosine | 0.245 | 0.162 | 0.328 | <0.001 | <0.001 | 0.190 | 0.106 | 0.274 | <0.001 | <0.001 |
| Alanine | 0.084 | <0.001 | 0.167 | 0.049 | 0.065 | 0.031 | -0.052 | 0.114 | 0.465 | 0.624 |
| Glutamine | -0.294 | -0.376 | -0.211 | <0.001 | <0.001 | -0.225 | -0.309 | -0.142 | <0.001 | <0.001 |
| Glycine | -0.083 | -0.171 | 0.005 | 0.063 | 0.082 | -0.005 | -0.093 | 0.084 | 0.914 | 0.957 |
| Histidine | -0.080 | -0.163 | 0.003 | 0.060 | 0.079 | -0.062 | -0.144 | 0.020 | 0.140 | 0.271 |
| BCAAs | 0.125 | 0.040 | 0.211 | 0.004 | 0.007 | 0.032 | -0.055 | 0.119 | 0.466 | 0.624 |
| Lactate | 0.055 | -0.028 | 0.138 | 0.195 | 0.232 | 0.009 | -0.073 | 0.092 | 0.825 | 0.913 |
| Pyruvate | 0.004 | -0.079 | 0.087 | 0.926 | 0.930 | -0.041 | -0.124 | 0.041 | 0.325 | 0.496 |
| Glucose | 0.311 | 0.229 | 0.394 | <0.001 | <0.001 | 0.161 | 0.072 | 0.249 | <0.001 | 0.002 |
| Citrate | -0.126 | -0.210 | -0.042 | 0.003 | 0.006 | -0.078 | -0.162 | 0.006 | 0.070 | 0.151 |
| **Apo-LP, LP size & Other lipids** |  |  |  |  |  |  |  |  |  |  |
| ApoA1 | 0.109 | 0.019 | 0.200 | 0.018 | 0.026 | 0.208 | 0.115 | 0.300 | <0.001 | <0.001 |
| ApoB | -0.110 | -0.193 | -0.027 | 0.009 | 0.014 | -0.056 | -0.145 | 0.033 | 0.216 | 0.368 |
| ApoB/ApoA1 | -0.145 | -0.231 | -0.060 | 0.001 | 0.002 | -0.154 | -0.245 | -0.063 | 0.001 | 0.004 |
| HDL-AD | -0.193 | -0.287 | -0.098 | <0.001 | <0.001 | 0.002 | -0.099 | 0.104 | 0.964 | 0.986 |
| LDL-AD | -0.206 | -0.291 | -0.121 | <0.001 | <0.001 | -0.106 | -0.193 | -0.020 | 0.016 | 0.041 |
| VLDL-AD | 0.206 | 0.116 | 0.296 | <0.001 | <0.001 | 0.064 | -0.030 | 0.159 | 0.184 | 0.337 |
| Phosphatidylcholine | 0.094 | 0.006 | 0.182 | 0.036 | 0.049 | 0.169 | 0.080 | 0.258 | <0.001 | 0.001 |
| Phosphoglycerides | 0.126 | 0.039 | 0.213 | 0.004 | 0.008 | 0.180 | 0.092 | 0.268 | <0.001 | <0.001 |
| Sphingomyeline | -0.072 | -0.160 | 0.015 | 0.105 | 0.132 | 0.050 | -0.041 | 0.141 | 0.284 | 0.453 |
| Total choline | 0.067 | -0.021 | 0.154 | 0.134 | 0.164 | 0.150 | 0.061 | 0.239 | 0.001 | 0.004 |
| TG/Phosphoglycerides | 0.206 | 0.116 | 0.295 | <0.001 | <0.001 | 0.047 | -0.048 | 0.142 | 0.331 | 0.502 |
| **Cholesterol** |  |  |  |  |  |  |  |  |  |  |
| XS-VLDL | -0.122 | -0.207 | -0.038 | 0.005 | 0.008 | -0.024 | -0.115 | 0.066 | 0.597 | 0.732 |
| S-VLDL | -0.067 | -0.150 | 0.017 | 0.117 | 0.146 | -0.055 | -0.142 | 0.033 | 0.220 | 0.373 |
| M-VLDL | -0.195 | -0.278 | -0.112 | <0.001 | <0.001 | -0.118 | -0.207 | -0.030 | 0.009 | 0.025 |
| L-VLDL | 0.066 | -0.019 | 0.151 | 0.130 | 0.161 | 0.001 | -0.089 | 0.091 | 0.986 | 0.990 |
| VL-VLDL | 0.088 | 0.003 | 0.173 | 0.044 | 0.059 | 0.021 | -0.069 | 0.112 | 0.648 | 0.771 |
| XL-VLDL | 0.208 | 0.123 | 0.294 | <0.001 | <0.001 | 0.113 | 0.023 | 0.202 | 0.014 | 0.036 |
| VLDL | -0.034 | -0.118 | 0.049 | 0.417 | 0.456 | -0.027 | -0.115 | 0.062 | 0.555 | 0.700 |
| S-LDL | -0.086 | -0.169 | -0.003 | 0.042 | 0.058 | -0.040 | -0.128 | 0.049 | 0.379 | 0.555 |
| M-LDL | -0.046 | -0.129 | 0.037 | 0.277 | 0.322 | -0.008 | -0.096 | 0.079 | 0.856 | 0.920 |
| L-LDL | -0.125 | -0.208 | -0.042 | 0.003 | 0.006 | -0.033 | -0.121 | 0.055 | 0.459 | 0.621 |
| Clinical LDL | -0.128 | -0.211 | -0.045 | 0.002 | 0.005 | -0.041 | -0.130 | 0.047 | 0.357 | 0.532 |
| LDL | -0.101 | -0.184 | -0.018 | 0.017 | 0.025 | -0.027 | -0.115 | 0.060 | 0.540 | 0.689 |
| IDL | -0.143 | -0.228 | -0.058 | 0.001 | 0.002 | -0.008 | -0.100 | 0.083 | 0.857 | 0.920 |
| S-HDL | 0.195 | 0.112 | 0.277 | <0.001 | <0.001 | 0.176 | 0.093 | 0.259 | <0.001 | <0.001 |
| M-HDL | 0.108 | 0.017 | 0.198 | 0.019 | 0.028 | 0.207 | 0.114 | 0.300 | <0.001 | <0.001 |
| L-HDL | -0.196 | -0.290 | -0.102 | <0.001 | <0.001 | 0.008 | -0.094 | 0.110 | 0.881 | 0.929 |
| XL-HDL | -0.275 | -0.367 | -0.183 | <0.001 | <0.001 | -0.068 | -0.168 | 0.032 | 0.186 | 0.337 |
| HDL | -0.040 | -0.133 | 0.052 | 0.394 | 0.437 | 0.129 | 0.031 | 0.226 | 0.010 | 0.027 |
| Total | -0.104 | -0.188 | -0.019 | 0.017 | 0.024 | 0.016 | -0.074 | 0.105 | 0.734 | 0.830 |
| Remnant | -0.090 | -0.173 | -0.007 | 0.033 | 0.046 | -0.020 | -0.110 | 0.069 | 0.657 | 0.771 |
| Total-HDLC | -0.098 | -0.181 | -0.015 | 0.021 | 0.030 | -0.025 | -0.113 | 0.064 | 0.586 | 0.730 |
| **Cholesterol to Total lipids** |  |  |  |  |  |  |  |  |  |  |
| VS-VLDL | -0.370 | -0.457 | -0.284 | <0.001 | <0.001 | -0.158 | -0.253 | -0.063 | 0.001 | 0.005 |
| S-VLDL | -0.281 | -0.365 | -0.197 | <0.001 | <0.001 | -0.132 | -0.220 | -0.044 | 0.003 | 0.012 |
| M-VLDL | -0.351 | -0.438 | -0.264 | <0.001 | <0.001 | -0.153 | -0.247 | -0.059 | 0.001 | 0.006 |
| L-VLDL | -0.183 | -0.266 | -0.099 | <0.001 | <0.001 | -0.069 | -0.154 | 0.016 | 0.111 | 0.227 |
| XL-VLDL | -0.244 | -0.330 | -0.158 | <0.001 | <0.001 | -0.091 | -0.181 | -0.002 | 0.045 | 0.104 |
| XXL-VLDL | -0.052 | -0.135 | 0.031 | 0.215 | 0.252 | -0.007 | -0.090 | 0.076 | 0.874 | 0.926 |
| S-LDL | -0.158 | -0.241 | -0.076 | <0.001 | <0.001 | -0.042 | -0.127 | 0.043 | 0.333 | 0.502 |
| M-LDL | -0.277 | -0.360 | -0.194 | <0.001 | <0.001 | -0.122 | -0.209 | -0.036 | 0.006 | 0.017 |
| L-LDL | -0.361 | -0.444 | -0.277 | <0.001 | <0.001 | -0.180 | -0.270 | -0.091 | <0.001 | 0.001 |
| IDL | -0.258 | -0.342 | -0.174 | <0.001 | <0.001 | -0.076 | -0.166 | 0.013 | 0.093 | 0.194 |
| S-HDL | -0.282 | -0.364 | -0.199 | <0.001 | <0.001 | -0.173 | -0.257 | -0.089 | <0.001 | <0.001 |
| M-HDL | -0.246 | -0.333 | -0.160 | <0.001 | <0.001 | -0.093 | -0.184 | -0.001 | 0.048 | 0.111 |
| L-HDL | -0.422 | -0.509 | -0.335 | <0.001 | <0.001 | -0.221 | -0.316 | -0.126 | <0.001 | <0.001 |
| XL-HDL | 0.038 | -0.050 | 0.126 | 0.401 | 0.440 | -0.026 | -0.115 | 0.062 | 0.556 | 0.700 |
| **Cholesteryl esters to Total lipids** |  |  |  |  |  |  |  |  |  |  |
| VS-VLDL | -0.361 | -0.448 | -0.274 | <0.001 | <0.001 | -0.141 | -0.237 | -0.045 | 0.004 | 0.012 |
| S-VLDL | -0.224 | -0.307 | -0.142 | <0.001 | <0.001 | -0.113 | -0.199 | -0.028 | 0.010 | 0.026 |
| M-VLDL | -0.357 | -0.444 | -0.270 | <0.001 | <0.001 | -0.158 | -0.252 | -0.064 | 0.001 | 0.005 |
| L-VLDL | -0.268 | -0.354 | -0.182 | <0.001 | <0.001 | -0.111 | -0.200 | -0.022 | 0.015 | 0.038 |
| XL-VLDL | -0.236 | -0.322 | -0.151 | <0.001 | <0.001 | -0.092 | -0.180 | -0.003 | 0.043 | 0.101 |
| XXL-VLDL | -0.066 | -0.149 | 0.016 | 0.115 | 0.144 | -0.022 | -0.104 | 0.061 | 0.602 | 0.735 |
| S-LDL | 0.126 | 0.043 | 0.210 | 0.003 | 0.005 | 0.113 | 0.028 | 0.198 | 0.009 | 0.026 |
| M-LDL | 0.091 | 0.007 | 0.175 | 0.034 | 0.047 | 0.062 | -0.024 | 0.149 | 0.159 | 0.305 |
| L-LDL | -0.155 | -0.238 | -0.073 | <0.001 | <0.001 | -0.097 | -0.180 | -0.014 | 0.021 | 0.053 |
| IDL | -0.172 | -0.256 | -0.088 | <0.001 | <0.001 | -0.017 | -0.105 | 0.071 | 0.708 | 0.806 |
| S-HDL | -0.225 | -0.308 | -0.142 | <0.001 | <0.001 | -0.149 | -0.232 | -0.065 | <0.001 | 0.002 |
| M-HDL | -0.240 | -0.325 | -0.156 | <0.001 | <0.001 | -0.112 | -0.201 | -0.024 | 0.013 | 0.035 |
| L-HDL | -0.421 | -0.507 | -0.335 | <0.001 | <0.001 | -0.234 | -0.327 | -0.141 | <0.001 | <0.001 |
| XL-HDL | -0.128 | -0.211 | -0.045 | 0.002 | 0.004 | -0.053 | -0.136 | 0.030 | 0.213 | 0.365 |
| **Cholestryl esters** |  |  |  |  |  |  |  |  |  |  |
| VS-VLDL | -0.147 | -0.232 | -0.062 | 0.001 | 0.002 | -0.031 | -0.122 | 0.061 | 0.509 | 0.667 |
| S-VLDL | -0.044 | -0.128 | 0.039 | 0.299 | 0.344 | -0.046 | -0.133 | 0.042 | 0.309 | 0.482 |
| M-VLDL | -0.252 | -0.335 | -0.169 | <0.001 | <0.001 | -0.147 | -0.236 | -0.058 | 0.001 | 0.005 |
| L-VLDL | -0.002 | -0.086 | 0.083 | 0.972 | 0.972 | -0.039 | -0.128 | 0.050 | 0.392 | 0.564 |
| VL-VLDL | 0.031 | -0.054 | 0.117 | 0.468 | 0.507 | -0.015 | -0.105 | 0.075 | 0.747 | 0.842 |
| XL-VLDL | 0.187 | 0.101 | 0.273 | <0.001 | <0.001 | 0.100 | 0.010 | 0.189 | 0.030 | 0.071 |
| VLDL | -0.080 | -0.163 | 0.003 | 0.060 | 0.079 | -0.048 | -0.137 | 0.040 | 0.283 | 0.453 |
| S-LDL | -0.045 | -0.128 | 0.038 | 0.285 | 0.328 | -0.014 | -0.102 | 0.074 | 0.759 | 0.848 |
| M-LDL | -0.014 | -0.097 | 0.069 | 0.740 | 0.764 | 0.009 | -0.079 | 0.097 | 0.841 | 0.919 |
| L-LDL | -0.110 | -0.193 | -0.027 | 0.010 | 0.015 | -0.028 | -0.116 | 0.059 | 0.525 | 0.678 |
| LDL | -0.079 | -0.162 | 0.004 | 0.062 | 0.082 | -0.017 | -0.104 | 0.070 | 0.699 | 0.806 |
| IDL | -0.133 | -0.218 | -0.048 | 0.002 | 0.004 | -0.001 | -0.092 | 0.091 | 0.990 | 0.990 |
| S-HDL | 0.181 | 0.098 | 0.263 | <0.001 | <0.001 | 0.159 | 0.076 | 0.242 | <0.001 | 0.001 |
| M-HDL | 0.105 | 0.015 | 0.195 | 0.022 | 0.031 | 0.203 | 0.111 | 0.296 | <0.001 | <0.001 |
| L-HDL | -0.205 | -0.299 | -0.111 | <0.001 | <0.001 | -0.001 | -0.103 | 0.101 | 0.983 | 0.990 |
| XL-HDL | -0.274 | -0.367 | -0.181 | <0.001 | <0.001 | -0.065 | -0.165 | 0.036 | 0.209 | 0.365 |
| HDL | -0.048 | -0.140 | 0.044 | 0.309 | 0.353 | 0.120 | 0.023 | 0.218 | 0.015 | 0.040 |
| Total | -0.101 | -0.186 | -0.016 | 0.020 | 0.029 | 0.024 | -0.066 | 0.114 | 0.597 | 0.732 |
| **Fatty acids** |  |  |  |  |  |  |  |  |  |  |
| DHA | -0.040 | -0.126 | 0.046 | 0.363 | 0.408 | 0.028 | -0.058 | 0.114 | 0.525 | 0.678 |
| LA | -0.105 | -0.189 | -0.022 | 0.013 | 0.020 | -0.022 | -0.109 | 0.064 | 0.616 | 0.748 |
| MUFA | 0.247 | 0.164 | 0.330 | <0.001 | <0.001 | 0.155 | 0.068 | 0.241 | <0.001 | 0.002 |
| n-3 | 0.047 | -0.038 | 0.132 | 0.278 | 0.322 | 0.056 | -0.029 | 0.140 | 0.199 | 0.355 |
| n-6 | -0.043 | -0.127 | 0.041 | 0.315 | 0.358 | 0.018 | -0.068 | 0.105 | 0.675 | 0.785 |
| PUFA | -0.024 | -0.108 | 0.060 | 0.578 | 0.610 | 0.031 | -0.055 | 0.118 | 0.473 | 0.630 |
| SFA | 0.237 | 0.154 | 0.321 | <0.001 | <0.001 | 0.194 | 0.108 | 0.280 | <0.001 | <0.001 |
| Total | 0.170 | 0.086 | 0.253 | <0.001 | <0.001 | 0.138 | 0.052 | 0.223 | 0.002 | 0.006 |
| Unsaturation | -0.271 | -0.357 | -0.185 | <0.001 | <0.001 | -0.143 | -0.232 | -0.054 | 0.002 | 0.006 |
| DHA/FA | -0.119 | -0.203 | -0.034 | 0.006 | 0.010 | -0.035 | -0.122 | 0.053 | 0.437 | 0.603 |
| LA/FA | -0.469 | -0.554 | -0.385 | <0.001 | <0.001 | -0.301 | -0.392 | -0.209 | <0.001 | <0.001 |
| MUFA/FA | 0.324 | 0.238 | 0.410 | <0.001 | <0.001 | 0.138 | 0.045 | 0.230 | 0.004 | 0.012 |
| n-3/FA | -0.020 | -0.105 | 0.064 | 0.637 | 0.667 | 0.009 | -0.077 | 0.094 | 0.843 | 0.919 |
| n-6/n-3 | -0.029 | -0.113 | 0.056 | 0.504 | 0.539 | -0.007 | -0.092 | 0.078 | 0.871 | 0.926 |
| n-6/FA | -0.423 | -0.508 | -0.339 | <0.001 | <0.001 | -0.279 | -0.369 | -0.189 | <0.001 | <0.001 |
| PUFA/MUFA | -0.351 | -0.436 | -0.265 | <0.001 | <0.001 | -0.178 | -0.271 | -0.086 | <0.001 | 0.001 |
| PUFA/FA | -0.420 | -0.504 | -0.335 | <0.001 | <0.001 | -0.271 | -0.362 | -0.180 | <0.001 | <0.001 |
| SFA/FA | 0.349 | 0.266 | 0.431 | <0.001 | <0.001 | 0.284 | 0.199 | 0.368 | <0.001 | <0.001 |
| **Free cholesterol** |  |  |  |  |  |  |  |  |  |  |
| VS-VLDL | -0.062 | -0.146 | 0.022 | 0.148 | 0.178 | -0.009 | -0.098 | 0.079 | 0.834 | 0.919 |
| S-VLDL | -0.104 | -0.187 | -0.021 | 0.014 | 0.021 | -0.070 | -0.157 | 0.018 | 0.118 | 0.236 |
| M-VLDL | -0.110 | -0.193 | -0.027 | 0.009 | 0.014 | -0.075 | -0.163 | 0.013 | 0.095 | 0.196 |
| L-VLDL | 0.130 | 0.044 | 0.217 | 0.003 | 0.005 | 0.040 | -0.051 | 0.131 | 0.391 | 0.564 |
| VL-VLDL | 0.144 | 0.059 | 0.230 | 0.001 | 0.002 | 0.058 | -0.033 | 0.148 | 0.212 | 0.365 |
| XL-VLDL | 0.231 | 0.145 | 0.316 | <0.001 | <0.001 | 0.127 | 0.038 | 0.216 | 0.005 | 0.016 |
| VLDL | 0.028 | -0.056 | 0.112 | 0.512 | 0.545 | 0.003 | -0.085 | 0.092 | 0.941 | 0.969 |
| S-LDL | -0.183 | -0.266 | -0.101 | <0.001 | <0.001 | -0.101 | -0.189 | -0.014 | 0.023 | 0.055 |
| M-LDL | -0.129 | -0.212 | -0.046 | 0.002 | 0.004 | -0.053 | -0.140 | 0.033 | 0.229 | 0.382 |
| L-LDL | -0.164 | -0.248 | -0.081 | <0.001 | <0.001 | -0.046 | -0.135 | 0.043 | 0.312 | 0.482 |
| LDL | -0.158 | -0.241 | -0.075 | <0.001 | <0.001 | -0.054 | -0.142 | 0.034 | 0.227 | 0.381 |
| IDL | -0.169 | -0.254 | -0.084 | <0.001 | <0.001 | -0.030 | -0.122 | 0.061 | 0.517 | 0.673 |
| S-HDL | 0.211 | 0.127 | 0.295 | <0.001 | <0.001 | 0.201 | 0.117 | 0.286 | <0.001 | <0.001 |
| M-HDL | 0.115 | 0.023 | 0.206 | 0.014 | 0.021 | 0.215 | 0.122 | 0.308 | <0.001 | <0.001 |
| L-HDL | -0.160 | -0.255 | -0.066 | 0.001 | 0.002 | 0.038 | -0.063 | 0.139 | 0.459 | 0.621 |
| XL-HDL | -0.263 | -0.353 | -0.173 | <0.001 | <0.001 | -0.074 | -0.170 | 0.022 | 0.133 | 0.258 |
| HDL | -0.011 | -0.104 | 0.082 | 0.818 | 0.835 | 0.150 | 0.053 | 0.247 | 0.003 | 0.010 |
| Total | -0.108 | -0.192 | -0.024 | 0.011 | 0.017 | -0.006 | -0.096 | 0.083 | 0.890 | 0.935 |
| **Free cholesterol to Total lipids** |  |  |  |  |  |  |  |  |  |  |
| VS-VLDL | -0.336 | -0.419 | -0.254 | <0.001 | <0.001 | -0.203 | -0.289 | -0.117 | <0.001 | <0.001 |
| S-VLDL | -0.317 | -0.402 | -0.232 | <0.001 | <0.001 | -0.137 | -0.228 | -0.045 | 0.003 | 0.012 |
| M-VLDL | -0.318 | -0.404 | -0.232 | <0.001 | <0.001 | -0.131 | -0.223 | -0.038 | 0.006 | 0.017 |
| L-VLDL | 0.063 | -0.021 | 0.146 | 0.141 | 0.172 | 0.035 | -0.050 | 0.119 | 0.421 | 0.590 |
| XL-VLDL | -0.203 | -0.288 | -0.118 | <0.001 | <0.001 | -0.060 | -0.147 | 0.027 | 0.179 | 0.334 |
| XXL-VLDL | -0.008 | -0.091 | 0.076 | 0.859 | 0.870 | 0.029 | -0.056 | 0.113 | 0.505 | 0.665 |
| S-LDL | -0.301 | -0.385 | -0.218 | <0.001 | <0.001 | -0.169 | -0.255 | -0.083 | <0.001 | 0.001 |
| M-LDL | -0.333 | -0.419 | -0.248 | <0.001 | <0.001 | -0.175 | -0.265 | -0.085 | <0.001 | 0.001 |
| L-LDL | -0.345 | -0.430 | -0.259 | <0.001 | <0.001 | -0.141 | -0.234 | -0.049 | 0.003 | 0.010 |
| IDL | -0.287 | -0.369 | -0.204 | <0.001 | <0.001 | -0.143 | -0.228 | -0.058 | 0.001 | 0.004 |
| S-HDL | -0.226 | -0.313 | -0.140 | <0.001 | <0.001 | -0.085 | -0.175 | 0.006 | 0.067 | 0.147 |
| M-HDL | -0.141 | -0.234 | -0.047 | 0.003 | 0.006 | 0.059 | -0.040 | 0.158 | 0.240 | 0.395 |
| L-HDL | -0.222 | -0.310 | -0.134 | <0.001 | <0.001 | -0.037 | -0.132 | 0.057 | 0.438 | 0.603 |
| XL-HDL | 0.208 | 0.115 | 0.300 | <0.001 | <0.001 | 0.022 | -0.075 | 0.120 | 0.655 | 0.771 |
| **KB, FB & Inflammation** |  |  |  |  |  |  |  |  |  |  |
| Glycoprotein acetyls | 0.306 | 0.223 | 0.389 | <0.001 | <0.001 | 0.179 | 0.091 | 0.268 | <0.001 | 0.001 |
| 3-Hydrocybutyrate | 0.114 | 0.031 | 0.196 | 0.007 | 0.011 | 0.121 | 0.039 | 0.203 | 0.004 | 0.012 |
| Acetate | -0.122 | -0.205 | -0.040 | 0.004 | 0.006 | -0.098 | -0.180 | -0.016 | 0.019 | 0.049 |
| Acetoacetate | 0.164 | 0.081 | 0.247 | <0.001 | <0.001 | 0.132 | 0.050 | 0.215 | 0.002 | 0.006 |
| Acetone | 0.115 | 0.032 | 0.197 | 0.007 | 0.011 | 0.120 | 0.037 | 0.202 | 0.004 | 0.014 |
| Albumin | 0.009 | -0.074 | 0.092 | 0.839 | 0.853 | 0.054 | -0.029 | 0.138 | 0.200 | 0.355 |
| Creatinine | -0.037 | -0.137 | 0.063 | 0.466 | 0.506 | -0.086 | -0.187 | 0.014 | 0.093 | 0.194 |
| **Lipoprotein particles** |  |  |  |  |  |  |  |  |  |  |
| VS-VLDL | -0.030 | -0.114 | 0.053 | 0.475 | 0.512 | 0.009 | -0.079 | 0.097 | 0.846 | 0.919 |
| S-VLDL | 0.041 | -0.043 | 0.126 | 0.337 | 0.380 | -0.009 | -0.096 | 0.079 | 0.849 | 0.919 |
| M-VLDL | -0.055 | -0.138 | 0.028 | 0.196 | 0.232 | -0.048 | -0.136 | 0.040 | 0.287 | 0.455 |
| L-VLDL | 0.146 | 0.060 | 0.232 | 0.001 | 0.002 | 0.051 | -0.039 | 0.142 | 0.266 | 0.433 |
| VL-VLDL | 0.193 | 0.107 | 0.280 | <0.001 | <0.001 | 0.085 | -0.006 | 0.176 | 0.066 | 0.147 |
| XL-VLDL | 0.245 | 0.159 | 0.330 | <0.001 | <0.001 | 0.134 | 0.045 | 0.223 | 0.003 | 0.012 |
| VLDL | 0.022 | -0.062 | 0.105 | 0.612 | 0.643 | 0.001 | -0.086 | 0.089 | 0.975 | 0.987 |
| S-LDL | -0.096 | -0.179 | -0.013 | 0.023 | 0.032 | -0.060 | -0.149 | 0.029 | 0.184 | 0.337 |
| M-LDL | -0.061 | -0.144 | 0.022 | 0.148 | 0.178 | -0.021 | -0.109 | 0.067 | 0.636 | 0.765 |
| L-LDL | -0.154 | -0.237 | -0.072 | <0.001 | 0.001 | -0.092 | -0.180 | -0.004 | 0.041 | 0.096 |
| LDL | -0.126 | -0.208 | -0.043 | 0.003 | 0.005 | -0.071 | -0.160 | 0.017 | 0.112 | 0.228 |
| IDL | -0.110 | -0.193 | -0.027 | 0.010 | 0.015 | -0.020 | -0.110 | 0.070 | 0.664 | 0.777 |
| S-HDL | 0.208 | 0.125 | 0.290 | <0.001 | <0.001 | 0.177 | 0.094 | 0.261 | <0.001 | <0.001 |
| M-HDL | 0.141 | 0.051 | 0.231 | 0.002 | 0.004 | 0.224 | 0.132 | 0.316 | <0.001 | <0.001 |
| L-HDL | -0.175 | -0.269 | -0.081 | <0.001 | 0.001 | 0.025 | -0.077 | 0.126 | 0.632 | 0.763 |
| XL-HDL | -0.250 | -0.342 | -0.158 | <0.001 | <0.001 | -0.050 | -0.149 | 0.049 | 0.324 | 0.496 |
| HDL | 0.121 | 0.033 | 0.208 | 0.007 | 0.011 | 0.195 | 0.106 | 0.284 | <0.001 | <0.001 |
| Total | 0.096 | 0.009 | 0.184 | 0.031 | 0.043 | 0.177 | 0.088 | 0.265 | <0.001 | 0.001 |
| **Phospholipids** |  |  |  |  |  |  |  |  |  |  |
| VS-VLDL | -0.007 | -0.090 | 0.077 | 0.877 | 0.884 | 0.013 | -0.074 | 0.100 | 0.768 | 0.854 |
| S-VLDL | -0.041 | -0.124 | 0.042 | 0.335 | 0.379 | -0.041 | -0.128 | 0.047 | 0.360 | 0.532 |
| M-VLDL | -0.062 | -0.145 | 0.021 | 0.143 | 0.173 | -0.048 | -0.136 | 0.040 | 0.281 | 0.453 |
| L-VLDL | 0.152 | 0.066 | 0.238 | 0.001 | 0.001 | 0.052 | -0.039 | 0.143 | 0.263 | 0.431 |
| VL-VLDL | 0.169 | 0.083 | 0.254 | <0.001 | <0.001 | 0.072 | -0.019 | 0.163 | 0.121 | 0.240 |
| XL-VLDL | 0.246 | 0.160 | 0.331 | <0.001 | <0.001 | 0.134 | 0.045 | 0.223 | 0.003 | 0.012 |
| VLDL | 0.071 | -0.013 | 0.155 | 0.099 | 0.126 | 0.025 | -0.064 | 0.114 | 0.578 | 0.723 |
| S-LDL | -0.112 | -0.194 | -0.029 | 0.008 | 0.013 | -0.068 | -0.156 | 0.020 | 0.129 | 0.253 |
| M-LDL | -0.036 | -0.119 | 0.047 | 0.399 | 0.439 | -0.007 | -0.095 | 0.080 | 0.872 | 0.926 |
| L-LDL | -0.102 | -0.185 | -0.019 | 0.016 | 0.023 | -0.017 | -0.105 | 0.071 | 0.709 | 0.806 |
| LDL | -0.085 | -0.168 | -0.002 | 0.044 | 0.059 | -0.021 | -0.109 | 0.067 | 0.640 | 0.766 |
| IDL | -0.145 | -0.230 | -0.060 | 0.001 | 0.002 | -0.025 | -0.116 | 0.066 | 0.594 | 0.732 |
| S-HDL | 0.332 | 0.249 | 0.415 | <0.001 | <0.001 | 0.272 | 0.188 | 0.356 | <0.001 | <0.001 |
| M-HDL | 0.241 | 0.153 | 0.330 | <0.001 | <0.001 | 0.273 | 0.184 | 0.363 | <0.001 | <0.001 |
| L-HDL | -0.087 | -0.182 | 0.007 | 0.070 | 0.091 | 0.090 | -0.011 | 0.190 | 0.079 | 0.169 |
| XL-HDL | -0.232 | -0.324 | -0.139 | <0.001 | <0.001 | -0.039 | -0.138 | 0.060 | 0.442 | 0.605 |
| HDL | 0.126 | 0.034 | 0.218 | 0.007 | 0.012 | 0.226 | 0.132 | 0.320 | <0.001 | <0.001 |
| Total | 0.056 | -0.030 | 0.142 | 0.202 | 0.238 | 0.138 | 0.049 | 0.227 | 0.002 | 0.009 |
| **Phospholipids to Total lipids** |  |  |  |  |  |  |  |  |  |  |
| VS-VLDL | 0.065 | -0.019 | 0.148 | 0.132 | 0.163 | -0.077 | -0.163 | 0.009 | 0.081 | 0.171 |
| S-VLDL | -0.308 | -0.393 | -0.223 | <0.001 | <0.001 | -0.135 | -0.226 | -0.044 | 0.004 | 0.012 |
| M-VLDL | -0.257 | -0.342 | -0.172 | <0.001 | <0.001 | -0.085 | -0.177 | 0.007 | 0.070 | 0.151 |
| L-VLDL | 0.141 | 0.056 | 0.227 | 0.001 | 0.002 | 0.037 | -0.052 | 0.125 | 0.418 | 0.590 |
| VL-VLDL | -0.036 | -0.119 | 0.047 | 0.395 | 0.437 | -0.036 | -0.121 | 0.049 | 0.405 | 0.576 |
| XL-VLDL | 0.117 | 0.034 | 0.201 | 0.006 | 0.010 | 0.027 | -0.058 | 0.111 | 0.537 | 0.689 |
| S-LDL | -0.139 | -0.223 | -0.055 | 0.001 | 0.002 | -0.113 | -0.198 | -0.028 | 0.009 | 0.025 |
| M-LDL | -0.069 | -0.152 | 0.014 | 0.102 | 0.129 | -0.077 | -0.159 | 0.006 | 0.070 | 0.151 |
| L-LDL | -0.015 | -0.098 | 0.068 | 0.715 | 0.742 | -0.016 | -0.099 | 0.067 | 0.703 | 0.806 |
| IDL | -0.117 | -0.200 | -0.035 | 0.005 | 0.009 | -0.142 | -0.224 | -0.059 | 0.001 | 0.004 |
| S-HDL | 0.241 | 0.155 | 0.328 | <0.001 | <0.001 | 0.218 | 0.131 | 0.305 | <0.001 | <0.001 |
| M-HDL | 0.297 | 0.210 | 0.383 | <0.001 | <0.001 | 0.126 | 0.033 | 0.219 | 0.008 | 0.024 |
| L-HDL | 0.532 | 0.447 | 0.617 | <0.001 | <0.001 | 0.340 | 0.245 | 0.436 | <0.001 | <0.001 |
| XL-HDL | -0.203 | -0.293 | -0.113 | <0.001 | <0.001 | -0.063 | -0.156 | 0.030 | 0.185 | 0.337 |
| **Total lipids** |  |  |  |  |  |  |  |  |  |  |
| VS-VLDL | -0.016 | -0.100 | 0.068 | 0.704 | 0.734 | 0.020 | -0.067 | 0.108 | 0.650 | 0.771 |
| S-VLDL | 0.038 | -0.046 | 0.122 | 0.370 | 0.414 | -0.004 | -0.091 | 0.084 | 0.936 | 0.969 |
| M-VLDL | -0.011 | -0.095 | 0.073 | 0.801 | 0.821 | -0.027 | -0.115 | 0.061 | 0.542 | 0.689 |
| L-VLDL | 0.134 | 0.048 | 0.220 | 0.002 | 0.004 | 0.042 | -0.048 | 0.132 | 0.361 | 0.532 |
| VL-VLDL | 0.192 | 0.106 | 0.278 | <0.001 | <0.001 | 0.086 | -0.004 | 0.177 | 0.062 | 0.140 |
| XL-VLDL | 0.237 | 0.152 | 0.322 | <0.001 | <0.001 | 0.130 | 0.041 | 0.220 | 0.004 | 0.013 |
| VLDL | 0.111 | 0.027 | 0.196 | 0.010 | 0.015 | 0.046 | -0.043 | 0.135 | 0.310 | 0.482 |
| S-LDL | -0.072 | -0.155 | 0.011 | 0.087 | 0.112 | -0.036 | -0.124 | 0.052 | 0.422 | 0.590 |
| M-LDL | -0.027 | -0.110 | 0.056 | 0.520 | 0.551 | 0.001 | -0.086 | 0.089 | 0.974 | 0.987 |
| L-LDL | -0.099 | -0.182 | -0.016 | 0.019 | 0.028 | -0.017 | -0.104 | 0.071 | 0.709 | 0.806 |
| LDL | -0.077 | -0.160 | 0.005 | 0.067 | 0.087 | -0.014 | -0.101 | 0.074 | 0.755 | 0.847 |
| IDL | -0.118 | -0.203 | -0.033 | 0.007 | 0.011 | 0.003 | -0.088 | 0.095 | 0.942 | 0.969 |
| S-HDL | 0.300 | 0.217 | 0.383 | <0.001 | <0.001 | 0.243 | 0.160 | 0.327 | <0.001 | <0.001 |
| M-HDL | 0.194 | 0.105 | 0.283 | <0.001 | <0.001 | 0.251 | 0.160 | 0.341 | <0.001 | <0.001 |
| L-HDL | -0.139 | -0.233 | -0.044 | 0.004 | 0.007 | 0.053 | -0.048 | 0.154 | 0.305 | 0.481 |
| XL-HDL | -0.247 | -0.340 | -0.154 | <0.001 | <0.001 | -0.048 | -0.148 | 0.052 | 0.345 | 0.517 |
| HDL | 0.065 | -0.027 | 0.158 | 0.166 | 0.199 | 0.193 | 0.097 | 0.288 | <0.001 | 0.001 |
| Total | 0.029 | -0.054 | 0.113 | 0.495 | 0.531 | 0.082 | -0.005 | 0.169 | 0.065 | 0.147 |
| **Triglycerides** |  |  |  |  |  |  |  |  |  |  |
| VS-VLDL | 0.212 | 0.129 | 0.296 | <0.001 | <0.001 | 0.114 | 0.029 | 0.200 | 0.009 | 0.025 |
| S-VLDL | 0.163 | 0.078 | 0.248 | <0.001 | <0.001 | 0.058 | -0.029 | 0.146 | 0.192 | 0.346 |
| M-VLDL | 0.120 | 0.035 | 0.205 | 0.006 | 0.010 | 0.036 | -0.053 | 0.125 | 0.427 | 0.594 |
| L-VLDL | 0.158 | 0.072 | 0.244 | <0.001 | 0.001 | 0.057 | -0.033 | 0.147 | 0.212 | 0.365 |
| XL-VLDL | 0.231 | 0.145 | 0.317 | <0.001 | <0.001 | 0.111 | 0.021 | 0.202 | 0.016 | 0.041 |
| XXL-VLDL | 0.240 | 0.155 | 0.326 | <0.001 | <0.001 | 0.133 | 0.044 | 0.222 | 0.003 | 0.012 |
| VLDL | 0.194 | 0.108 | 0.280 | <0.001 | <0.001 | 0.087 | -0.002 | 0.177 | 0.056 | 0.128 |
| S-LDL | 0.216 | 0.132 | 0.301 | <0.001 | <0.001 | 0.118 | 0.030 | 0.206 | 0.008 | 0.024 |
| M-LDL | 0.241 | 0.157 | 0.325 | <0.001 | <0.001 | 0.145 | 0.058 | 0.232 | 0.001 | 0.005 |
| L-LDL | 0.247 | 0.164 | 0.331 | <0.001 | <0.001 | 0.165 | 0.079 | 0.251 | <0.001 | 0.001 |
| LDL | 0.245 | 0.161 | 0.328 | <0.001 | <0.001 | 0.156 | 0.070 | 0.242 | <0.001 | 0.002 |
| IDL | 0.222 | 0.139 | 0.305 | <0.001 | <0.001 | 0.144 | 0.059 | 0.230 | 0.001 | 0.004 |
| S-HDL | 0.294 | 0.209 | 0.379 | <0.001 | <0.001 | 0.157 | 0.068 | 0.247 | 0.001 | 0.003 |
| M-HDL | 0.277 | 0.194 | 0.360 | <0.001 | <0.001 | 0.185 | 0.101 | 0.270 | <0.001 | <0.001 |
| L-HDL | 0.104 | 0.018 | 0.189 | 0.017 | 0.025 | 0.113 | 0.028 | 0.197 | 0.009 | 0.026 |
| XL-HDL | 0.078 | -0.006 | 0.161 | 0.067 | 0.087 | 0.066 | -0.018 | 0.150 | 0.122 | 0.240 |
| HDL | 0.245 | 0.162 | 0.328 | <0.001 | <0.001 | 0.159 | 0.074 | 0.243 | <0.001 | 0.001 |
| Total | 0.210 | 0.125 | 0.295 | <0.001 | <0.001 | 0.105 | 0.017 | 0.194 | 0.020 | 0.050 |
| **Triglycerides to Total lipids** |  |  |  |  |  |  |  |  |  |  |
| VS-VLDL | 0.400 | 0.314 | 0.486 | <0.001 | <0.001 | 0.198 | 0.103 | 0.293 | <0.001 | <0.001 |
| S-VLDL | 0.295 | 0.210 | 0.379 | <0.001 | <0.001 | 0.136 | 0.047 | 0.225 | 0.003 | 0.011 |
| M-VLDL | 0.337 | 0.250 | 0.423 | <0.001 | <0.001 | 0.141 | 0.047 | 0.235 | 0.003 | 0.012 |
| L-VLDL | 0.054 | -0.029 | 0.137 | 0.200 | 0.236 | 0.029 | -0.054 | 0.113 | 0.488 | 0.647 |
| XL-VLDL | 0.247 | 0.163 | 0.332 | <0.001 | <0.001 | 0.101 | 0.013 | 0.188 | 0.024 | 0.058 |
| XXL-VLDL | 0.012 | -0.070 | 0.095 | 0.769 | 0.791 | -0.002 | -0.084 | 0.081 | 0.966 | 0.986 |
| S-LDL | 0.333 | 0.249 | 0.417 | <0.001 | <0.001 | 0.177 | 0.089 | 0.264 | <0.001 | 0.001 |
| M-LDL | 0.336 | 0.253 | 0.419 | <0.001 | <0.001 | 0.179 | 0.092 | 0.266 | <0.001 | <0.001 |
| L-LDL | 0.372 | 0.289 | 0.456 | <0.001 | <0.001 | 0.190 | 0.102 | 0.279 | <0.001 | <0.001 |
| IDL | 0.366 | 0.282 | 0.451 | <0.001 | <0.001 | 0.166 | 0.075 | 0.258 | <0.001 | 0.002 |
| S-HDL | 0.204 | 0.117 | 0.291 | <0.001 | <0.001 | 0.056 | -0.036 | 0.147 | 0.233 | 0.387 |
| M-HDL | 0.193 | 0.107 | 0.279 | <0.001 | <0.001 | 0.063 | -0.027 | 0.153 | 0.169 | 0.319 |
| L-HDL | 0.207 | 0.120 | 0.294 | <0.001 | <0.001 | 0.058 | -0.033 | 0.149 | 0.211 | 0.365 |
| XL-HDL | 0.315 | 0.229 | 0.401 | <0.001 | <0.001 | 0.157 | 0.067 | 0.247 | 0.001 | 0.003 |

† Model adjusted for age, sex, and education.
‡ Model adjusted for age, sex, education, race, socioeconomic status, body mass index, smoking status, alcohol drinking status, physical activity, social connection, hypertension, diabetes, heart disease, beta-blockers, calcium blockers, lipid-lowering, and APOE ε4 status.
Abbreviation: Apo, apolipoprotein; LDL-AD, average diameter for LDL particles; CHOL, cholesterol; CE, cholesteryl esters; TG, triglycerides; S, small; M, medium; L, large; VL, very large; XL, extremely large; VLDL, very low-density lipoprotein; LDL; low-density lipoprotein; IDL, intermediate-density lipoprotein; HDL, high-density lipoprotein; LA, linoleic acid; MUFA, monounsaturated fatty acid; n-6, omega-6 fatty acid; PUFA, polyunsaturated fatty acid; SFA, saturated fatty acid; FA, fatty acid.

**Table S15.** β coefficients and 95% confidence intervals (CIs) for the association between metabolites and brain age gap (BAG) after multiple imputation of covariates: results from linear regression models

| **Metabolites** | **Basic-model ^†^** | |  |  |  | **Multi-model ^‡^** | |  |  |  |
| --- | --- | --- | --- | --- | --- | --- | --- | --- | --- | --- |
|  | **β** | **Lower** | **Upper** | ***P*-value** | **FDR-q** | **β** | **Lower** | **Upper** | ***P*-value** | **FDR-q** |
| **Amino acids & Glycolysis** |  |  |  |  |  |  |  |  |  |  |
| Valine | 0.112 | 0.034 | 0.190 | 0.005 | 0.008 | -0.012 | -0.092 | 0.069 | 0.778 | 0.885 |
| Leucine | 0.086 | 0.007 | 0.165 | 0.032 | 0.042 | -0.011 | -0.090 | 0.069 | 0.790 | 0.891 |
| Isoleucine | 0.039 | -0.039 | 0.116 | 0.328 | 0.360 | -0.043 | -0.120 | 0.035 | 0.281 | 0.438 |
| Phenylalanine | 0.078 | 0.002 | 0.154 | 0.045 | 0.058 | 0.016 | -0.059 | 0.092 | 0.673 | 0.802 |
| Tyrosine | 0.190 | 0.114 | 0.267 | 0.000 | 0.000 | 0.121 | 0.044 | 0.197 | 0.002 | 0.007 |
| Alanine | 0.107 | 0.031 | 0.183 | 0.006 | 0.009 | 0.048 | -0.028 | 0.124 | 0.215 | 0.359 |
| Glutamine | -0.311 | -0.387 | -0.235 | 0.000 | 0.000 | -0.229 | -0.306 | -0.153 | 0.000 | 0.000 |
| Glycine | -0.113 | -0.193 | -0.032 | 0.006 | 0.010 | -0.021 | -0.102 | 0.061 | 0.618 | 0.774 |
| Histidine | -0.097 | -0.174 | -0.021 | 0.012 | 0.018 | -0.078 | -0.153 | -0.002 | 0.044 | 0.096 |
| BCAAs | 0.092 | 0.014 | 0.171 | 0.021 | 0.029 | -0.019 | -0.098 | 0.061 | 0.647 | 0.785 |
| Lactate | 0.183 | 0.107 | 0.259 | 0.000 | 0.000 | 0.131 | 0.055 | 0.206 | 0.001 | 0.003 |
| Pyruvate | 0.130 | 0.054 | 0.206 | 0.001 | 0.001 | 0.080 | 0.004 | 0.155 | 0.038 | 0.086 |
| Glucose | 0.296 | 0.220 | 0.372 | 0.000 | 0.000 | 0.134 | 0.053 | 0.215 | 0.001 | 0.004 |
| Citrate | -0.094 | -0.171 | -0.017 | 0.017 | 0.024 | -0.037 | -0.115 | 0.040 | 0.341 | 0.512 |
| **Apo-LP, LP size & Other lipids** | |  |  |  |  |  |  |  |  |  |
| ApoA1 | 0.115 | 0.032 | 0.198 | 0.007 | 0.010 | 0.225 | 0.140 | 0.309 | 0.000 | 0.000 |
| ApoB | -0.101 | -0.177 | -0.025 | 0.009 | 0.014 | -0.050 | -0.131 | 0.031 | 0.230 | 0.379 |
| ApoB/ApoA1 | -0.144 | -0.222 | -0.065 | 0.000 | 0.001 | -0.161 | -0.244 | -0.077 | 0.000 | 0.001 |
| HDL-AD | -0.232 | -0.318 | -0.145 | 0.000 | 0.000 | -0.010 | -0.103 | 0.083 | 0.835 | 0.925 |
| LDL-AD | -0.193 | -0.272 | -0.115 | 0.000 | 0.000 | -0.079 | -0.158 | 0.000 | 0.050 | 0.107 |
| VLDL-AD | 0.242 | 0.159 | 0.324 | 0.000 | 0.000 | 0.077 | -0.010 | 0.163 | 0.084 | 0.164 |
| Phosphatidylcholine | 0.086 | 0.006 | 0.167 | 0.036 | 0.047 | 0.165 | 0.083 | 0.247 | 0.000 | 0.000 |
| Phosphoglycerides | 0.131 | 0.051 | 0.211 | 0.001 | 0.002 | 0.185 | 0.104 | 0.265 | 0.000 | 0.000 |
| Sphingomyeline | -0.050 | -0.131 | 0.030 | 0.220 | 0.251 | 0.081 | -0.003 | 0.164 | 0.058 | 0.119 |
| Total choline | 0.073 | -0.008 | 0.153 | 0.077 | 0.097 | 0.159 | 0.078 | 0.241 | 0.000 | 0.001 |
| TG/Phosphoglycerides | 0.223 | 0.141 | 0.305 | 0.000 | 0.000 | 0.039 | -0.048 | 0.126 | 0.382 | 0.550 |
| **Cholesterol** |  |  |  |  |  |  |  |  |  |  |
| XS-VLDL | -0.136 | -0.214 | -0.059 | 0.001 | 0.001 | -0.039 | -0.121 | 0.044 | 0.361 | 0.535 |
| S-VLDL | -0.054 | -0.130 | 0.023 | 0.168 | 0.197 | -0.050 | -0.130 | 0.031 | 0.225 | 0.373 |
| M-VLDL | -0.183 | -0.259 | -0.107 | 0.000 | 0.000 | -0.105 | -0.186 | -0.024 | 0.011 | 0.029 |
| L-VLDL | 0.089 | 0.010 | 0.167 | 0.027 | 0.036 | 0.007 | -0.075 | 0.089 | 0.869 | 0.943 |
| VL-VLDL | 0.115 | 0.036 | 0.193 | 0.004 | 0.007 | 0.031 | -0.052 | 0.114 | 0.466 | 0.642 |
| XL-VLDL | 0.234 | 0.156 | 0.313 | 0.000 | 0.000 | 0.119 | 0.037 | 0.201 | 0.004 | 0.012 |
| VLDL | -0.020 | -0.096 | 0.056 | 0.608 | 0.633 | -0.021 | -0.102 | 0.060 | 0.605 | 0.760 |
| S-LDL | -0.068 | -0.144 | 0.008 | 0.079 | 0.098 | -0.025 | -0.105 | 0.056 | 0.550 | 0.713 |
| M-LDL | -0.026 | -0.102 | 0.051 | 0.510 | 0.538 | 0.008 | -0.072 | 0.088 | 0.848 | 0.930 |
| L-LDL | -0.118 | -0.194 | -0.042 | 0.002 | 0.004 | -0.023 | -0.103 | 0.057 | 0.573 | 0.731 |
| Clinical LDL | -0.118 | -0.194 | -0.042 | 0.002 | 0.004 | -0.030 | -0.111 | 0.051 | 0.467 | 0.642 |
| LDL | -0.089 | -0.165 | -0.013 | 0.021 | 0.029 | -0.015 | -0.095 | 0.065 | 0.711 | 0.835 |
| IDL | -0.153 | -0.231 | -0.075 | 0.000 | 0.000 | -0.013 | -0.097 | 0.071 | 0.760 | 0.873 |
| S-HDL | 0.236 | 0.160 | 0.312 | 0.000 | 0.000 | 0.209 | 0.133 | 0.285 | 0.000 | 0.000 |
| M-HDL | 0.117 | 0.034 | 0.200 | 0.006 | 0.009 | 0.230 | 0.145 | 0.315 | 0.000 | 0.000 |
| L-HDL | -0.222 | -0.309 | -0.136 | 0.000 | 0.000 | 0.011 | -0.083 | 0.104 | 0.822 | 0.921 |
| XL-HDL | -0.316 | -0.401 | -0.231 | 0.000 | 0.000 | -0.081 | -0.173 | 0.011 | 0.084 | 0.164 |
| HDL | -0.045 | -0.130 | 0.041 | 0.305 | 0.339 | 0.147 | 0.057 | 0.236 | 0.001 | 0.005 |
| Total | -0.097 | -0.175 | -0.019 | 0.014 | 0.021 | 0.028 | -0.055 | 0.110 | 0.511 | 0.686 |
| Remnant | -0.086 | -0.163 | -0.010 | 0.026 | 0.036 | -0.019 | -0.101 | 0.062 | 0.643 | 0.785 |
| Total-HDLC | -0.090 | -0.166 | -0.013 | 0.021 | 0.029 | -0.018 | -0.099 | 0.064 | 0.672 | 0.802 |
| **Cholesterol to Total lipids** |  |  |  |  |  |  |  |  |  |  |
| VS-VLDL | -0.389 | -0.469 | -0.310 | 0.000 | 0.000 | -0.155 | -0.242 | -0.068 | 0.000 | 0.002 |
| S-VLDL | -0.290 | -0.367 | -0.213 | 0.000 | 0.000 | -0.127 | -0.208 | -0.046 | 0.002 | 0.007 |
| M-VLDL | -0.376 | -0.456 | -0.296 | 0.000 | 0.000 | -0.156 | -0.242 | -0.070 | 0.000 | 0.002 |
| L-VLDL | -0.216 | -0.293 | -0.139 | 0.000 | 0.000 | -0.092 | -0.170 | -0.014 | 0.021 | 0.051 |
| XL-VLDL | -0.264 | -0.342 | -0.185 | 0.000 | 0.000 | -0.090 | -0.172 | -0.008 | 0.031 | 0.073 |
| XXL-VLDL | -0.055 | -0.131 | 0.021 | 0.158 | 0.189 | 0.000 | -0.076 | 0.076 | 0.997 | 1.000 |
| S-LDL | -0.138 | -0.214 | -0.062 | 0.000 | 0.001 | -0.017 | -0.095 | 0.061 | 0.672 | 0.802 |
| M-LDL | -0.257 | -0.332 | -0.181 | 0.000 | 0.000 | -0.091 | -0.170 | -0.012 | 0.024 | 0.059 |
| L-LDL | -0.359 | -0.436 | -0.282 | 0.000 | 0.000 | -0.157 | -0.239 | -0.076 | 0.000 | 0.001 |
| IDL | -0.267 | -0.344 | -0.190 | 0.000 | 0.000 | -0.070 | -0.152 | 0.012 | 0.094 | 0.184 |
| S-HDL | -0.267 | -0.343 | -0.191 | 0.000 | 0.000 | -0.143 | -0.220 | -0.066 | 0.000 | 0.001 |
| M-HDL | -0.238 | -0.317 | -0.158 | 0.000 | 0.000 | -0.056 | -0.141 | 0.028 | 0.188 | 0.324 |
| L-HDL | -0.421 | -0.501 | -0.341 | 0.000 | 0.000 | -0.186 | -0.273 | -0.098 | 0.000 | 0.000 |
| XL-HDL | 0.078 | -0.003 | 0.159 | 0.058 | 0.074 | 0.002 | -0.078 | 0.083 | 0.952 | 0.972 |
| **Cholesteryl esters to Total lipids** | |  |  |  |  |  |  |  |  |  |
| VS-VLDL | -0.385 | -0.465 | -0.306 | 0.000 | 0.000 | -0.144 | -0.232 | -0.056 | 0.001 | 0.005 |
| S-VLDL | -0.233 | -0.309 | -0.157 | 0.000 | 0.000 | -0.114 | -0.193 | -0.036 | 0.004 | 0.012 |
| M-VLDL | -0.381 | -0.461 | -0.301 | 0.000 | 0.000 | -0.158 | -0.244 | -0.072 | 0.000 | 0.002 |
| L-VLDL | -0.306 | -0.385 | -0.227 | 0.000 | 0.000 | -0.132 | -0.214 | -0.051 | 0.002 | 0.005 |
| XL-VLDL | -0.246 | -0.324 | -0.167 | 0.000 | 0.000 | -0.080 | -0.161 | 0.001 | 0.053 | 0.111 |
| XXL-VLDL | -0.054 | -0.130 | 0.022 | 0.163 | 0.193 | -0.002 | -0.078 | 0.073 | 0.956 | 0.972 |
| S-LDL | 0.147 | 0.071 | 0.224 | 0.000 | 0.000 | 0.122 | 0.044 | 0.200 | 0.002 | 0.007 |
| M-LDL | 0.122 | 0.045 | 0.199 | 0.002 | 0.003 | 0.079 | -0.001 | 0.158 | 0.052 | 0.109 |
| L-LDL | -0.138 | -0.214 | -0.062 | 0.000 | 0.001 | -0.076 | -0.152 | 0.000 | 0.049 | 0.105 |
| IDL | -0.186 | -0.263 | -0.109 | 0.000 | 0.000 | -0.020 | -0.100 | 0.061 | 0.630 | 0.781 |
| S-HDL | -0.199 | -0.275 | -0.122 | 0.000 | 0.000 | -0.113 | -0.189 | -0.036 | 0.004 | 0.012 |
| M-HDL | -0.225 | -0.303 | -0.147 | 0.000 | 0.000 | -0.073 | -0.154 | 0.009 | 0.080 | 0.162 |
| L-HDL | -0.410 | -0.489 | -0.331 | 0.000 | 0.000 | -0.189 | -0.275 | -0.104 | 0.000 | 0.000 |
| XL-HDL | -0.090 | -0.166 | -0.014 | 0.020 | 0.028 | -0.008 | -0.084 | 0.068 | 0.828 | 0.925 |
| **Cholestryl esters** |  |  |  |  |  |  |  |  |  |  |
| VS-VLDL | -0.164 | -0.242 | -0.086 | 0.000 | 0.000 | -0.046 | -0.130 | 0.037 | 0.278 | 0.438 |
| S-VLDL | -0.032 | -0.109 | 0.044 | 0.410 | 0.446 | -0.043 | -0.123 | 0.037 | 0.293 | 0.451 |
| M-VLDL | -0.244 | -0.320 | -0.168 | 0.000 | 0.000 | -0.132 | -0.214 | -0.051 | 0.001 | 0.005 |
| L-VLDL | 0.020 | -0.058 | 0.097 | 0.618 | 0.641 | -0.031 | -0.112 | 0.051 | 0.460 | 0.642 |
| VL-VLDL | 0.065 | -0.013 | 0.143 | 0.104 | 0.127 | 0.005 | -0.078 | 0.088 | 0.909 | 0.957 |
| XL-VLDL | 0.222 | 0.143 | 0.301 | 0.000 | 0.000 | 0.117 | 0.035 | 0.199 | 0.005 | 0.015 |
| VLDL | -0.067 | -0.143 | 0.009 | 0.086 | 0.106 | -0.042 | -0.123 | 0.039 | 0.313 | 0.476 |
| S-LDL | -0.026 | -0.102 | 0.050 | 0.504 | 0.534 | 0.001 | -0.080 | 0.081 | 0.990 | 0.998 |
| M-LDL | 0.009 | -0.068 | 0.085 | 0.821 | 0.828 | 0.025 | -0.055 | 0.106 | 0.536 | 0.705 |
| L-LDL | -0.101 | -0.177 | -0.025 | 0.009 | 0.014 | -0.018 | -0.098 | 0.062 | 0.663 | 0.801 |
| LDL | -0.065 | -0.141 | 0.011 | 0.094 | 0.115 | -0.005 | -0.084 | 0.075 | 0.911 | 0.957 |
| IDL | -0.143 | -0.221 | -0.065 | 0.000 | 0.001 | -0.007 | -0.091 | 0.077 | 0.876 | 0.943 |
| S-HDL | 0.227 | 0.151 | 0.303 | 0.000 | 0.000 | 0.198 | 0.122 | 0.274 | 0.000 | 0.000 |
| M-HDL | 0.117 | 0.034 | 0.199 | 0.006 | 0.009 | 0.229 | 0.144 | 0.313 | 0.000 | 0.000 |
| L-HDL | -0.230 | -0.316 | -0.143 | 0.000 | 0.000 | 0.005 | -0.089 | 0.098 | 0.921 | 0.957 |
| XL-HDL | -0.312 | -0.397 | -0.226 | 0.000 | 0.000 | -0.073 | -0.165 | 0.019 | 0.120 | 0.228 |
| HDL | -0.049 | -0.134 | 0.036 | 0.260 | 0.294 | 0.143 | 0.054 | 0.232 | 0.002 | 0.006 |
| Total | -0.095 | -0.173 | -0.017 | 0.017 | 0.024 | 0.037 | -0.045 | 0.119 | 0.379 | 0.549 |
| **Fatty acids** |  |  |  |  |  |  |  |  |  |  |
| DHA | -0.079 | -0.157 | 0.000 | 0.051 | 0.065 | -0.007 | -0.086 | 0.072 | 0.859 | 0.939 |
| LA | -0.122 | -0.199 | -0.046 | 0.002 | 0.003 | -0.037 | -0.116 | 0.043 | 0.366 | 0.538 |
| MUFA | 0.285 | 0.209 | 0.362 | 0.000 | 0.000 | 0.176 | 0.097 | 0.256 | 0.000 | 0.000 |
| n-3 | 0.028 | -0.051 | 0.106 | 0.488 | 0.524 | 0.031 | -0.047 | 0.109 | 0.433 | 0.612 |
| n-6 | -0.042 | -0.119 | 0.035 | 0.284 | 0.319 | 0.019 | -0.060 | 0.098 | 0.640 | 0.785 |
| PUFA | -0.028 | -0.106 | 0.049 | 0.471 | 0.508 | 0.025 | -0.054 | 0.104 | 0.538 | 0.705 |
| SFA | 0.261 | 0.185 | 0.338 | 0.000 | 0.000 | 0.204 | 0.125 | 0.282 | 0.000 | 0.000 |
| Total | 0.190 | 0.114 | 0.267 | 0.000 | 0.000 | 0.147 | 0.068 | 0.225 | 0.000 | 0.001 |
| Unsaturation | -0.327 | -0.406 | -0.248 | 0.000 | 0.000 | -0.187 | -0.268 | -0.105 | 0.000 | 0.000 |
| DHA/FA | -0.180 | -0.258 | -0.102 | 0.000 | 0.000 | -0.087 | -0.167 | -0.007 | 0.032 | 0.075 |
| LA/FA | -0.535 | -0.612 | -0.458 | 0.000 | 0.000 | -0.346 | -0.429 | -0.262 | 0.000 | 0.000 |
| MUFA/FA | 0.395 | 0.317 | 0.474 | 0.000 | 0.000 | 0.191 | 0.106 | 0.276 | 0.000 | 0.000 |
| n-3/FA | -0.065 | -0.143 | 0.013 | 0.103 | 0.126 | -0.035 | -0.113 | 0.043 | 0.375 | 0.546 |
| n-6/n-3 | -0.006 | -0.084 | 0.071 | 0.873 | 0.873 | 0.023 | -0.055 | 0.100 | 0.562 | 0.725 |
| n-6/FA | -0.467 | -0.545 | -0.390 | 0.000 | 0.000 | -0.299 | -0.381 | -0.217 | 0.000 | 0.000 |
| PUFA/MUFA | -0.428 | -0.506 | -0.349 | 0.000 | 0.000 | -0.237 | -0.322 | -0.152 | 0.000 | 0.000 |
| PUFA/FA | -0.481 | -0.559 | -0.404 | 0.000 | 0.000 | -0.313 | -0.396 | -0.229 | 0.000 | 0.000 |
| SFA/FA | 0.372 | 0.296 | 0.447 | 0.000 | 0.000 | 0.293 | 0.216 | 0.370 | 0.000 | 0.000 |
| **Free cholesterol** |  |  |  |  |  |  |  |  |  |  |
| VS-VLDL | -0.068 | -0.145 | 0.009 | 0.083 | 0.104 | -0.020 | -0.101 | 0.061 | 0.626 | 0.779 |
| S-VLDL | -0.090 | -0.166 | -0.014 | 0.020 | 0.028 | -0.060 | -0.141 | 0.020 | 0.140 | 0.257 |
| M-VLDL | -0.095 | -0.171 | -0.018 | 0.015 | 0.021 | -0.064 | -0.144 | 0.017 | 0.121 | 0.229 |
| L-VLDL | 0.154 | 0.075 | 0.233 | 0.000 | 0.000 | 0.044 | -0.039 | 0.127 | 0.302 | 0.462 |
| VL-VLDL | 0.163 | 0.084 | 0.242 | 0.000 | 0.000 | 0.057 | -0.026 | 0.140 | 0.180 | 0.314 |
| XL-VLDL | 0.245 | 0.166 | 0.323 | 0.000 | 0.000 | 0.120 | 0.038 | 0.201 | 0.004 | 0.012 |
| VLDL | 0.044 | -0.033 | 0.121 | 0.261 | 0.294 | 0.006 | -0.075 | 0.088 | 0.878 | 0.943 |
| S-LDL | -0.172 | -0.248 | -0.096 | 0.000 | 0.000 | -0.087 | -0.167 | -0.007 | 0.033 | 0.077 |
| M-LDL | -0.117 | -0.193 | -0.041 | 0.003 | 0.004 | -0.040 | -0.119 | 0.040 | 0.329 | 0.497 |
| L-LDL | -0.162 | -0.239 | -0.086 | 0.000 | 0.000 | -0.037 | -0.119 | 0.044 | 0.367 | 0.538 |
| LDL | -0.152 | -0.228 | -0.076 | 0.000 | 0.000 | -0.044 | -0.124 | 0.037 | 0.289 | 0.446 |
| IDL | -0.175 | -0.253 | -0.097 | 0.000 | 0.000 | -0.031 | -0.115 | 0.053 | 0.472 | 0.646 |
| S-HDL | 0.232 | 0.155 | 0.309 | 0.000 | 0.000 | 0.217 | 0.139 | 0.294 | 0.000 | 0.000 |
| M-HDL | 0.115 | 0.031 | 0.198 | 0.007 | 0.011 | 0.227 | 0.142 | 0.312 | 0.000 | 0.000 |
| L-HDL | -0.194 | -0.280 | -0.107 | 0.000 | 0.000 | 0.031 | -0.061 | 0.124 | 0.509 | 0.686 |
| XL-HDL | -0.314 | -0.397 | -0.231 | 0.000 | 0.000 | -0.103 | -0.191 | -0.014 | 0.023 | 0.055 |
| HDL | -0.028 | -0.113 | 0.058 | 0.529 | 0.556 | 0.153 | 0.063 | 0.242 | 0.001 | 0.003 |
| Total | -0.102 | -0.179 | -0.024 | 0.010 | 0.014 | 0.004 | -0.078 | 0.085 | 0.930 | 0.957 |
| **Free cholesterol to Total lipids** | |  |  |  |  |  |  |  |  |  |
| VS-VLDL | -0.317 | -0.393 | -0.241 | 0.000 | 0.000 | -0.171 | -0.250 | -0.092 | 0.000 | 0.000 |
| S-VLDL | -0.324 | -0.403 | -0.246 | 0.000 | 0.000 | -0.123 | -0.207 | -0.039 | 0.004 | 0.012 |
| M-VLDL | -0.346 | -0.425 | -0.267 | 0.000 | 0.000 | -0.142 | -0.227 | -0.057 | 0.001 | 0.004 |
| L-VLDL | 0.049 | -0.028 | 0.125 | 0.215 | 0.246 | 0.012 | -0.065 | 0.089 | 0.761 | 0.873 |
| XL-VLDL | -0.253 | -0.331 | -0.175 | 0.000 | 0.000 | -0.096 | -0.176 | -0.016 | 0.019 | 0.048 |
| XXL-VLDL | -0.041 | -0.118 | 0.036 | 0.295 | 0.330 | 0.004 | -0.073 | 0.081 | 0.915 | 0.957 |
| S-LDL | -0.303 | -0.380 | -0.226 | 0.000 | 0.000 | -0.152 | -0.231 | -0.073 | 0.000 | 0.001 |
| M-LDL | -0.348 | -0.427 | -0.270 | 0.000 | 0.000 | -0.166 | -0.249 | -0.083 | 0.000 | 0.001 |
| L-LDL | -0.361 | -0.440 | -0.283 | 0.000 | 0.000 | -0.134 | -0.219 | -0.049 | 0.002 | 0.007 |
| IDL | -0.278 | -0.354 | -0.202 | 0.000 | 0.000 | -0.122 | -0.200 | -0.044 | 0.002 | 0.007 |
| S-HDL | -0.272 | -0.351 | -0.192 | 0.000 | 0.000 | -0.113 | -0.196 | -0.031 | 0.007 | 0.020 |
| M-HDL | -0.167 | -0.252 | -0.081 | 0.000 | 0.000 | 0.054 | -0.036 | 0.145 | 0.239 | 0.391 |
| L-HDL | -0.262 | -0.343 | -0.181 | 0.000 | 0.000 | -0.062 | -0.148 | 0.025 | 0.164 | 0.292 |
| XL-HDL | 0.226 | 0.141 | 0.311 | 0.000 | 0.000 | 0.015 | -0.074 | 0.105 | 0.741 | 0.858 |
| **KB, FB & Inflammation** |  |  |  |  |  |  |  |  |  |  |
| Glycoprotein acetyls | 0.327 | 0.251 | 0.404 | 0.000 | 0.000 | 0.175 | 0.093 | 0.256 | 0.000 | 0.000 |
| 3-Hydrocybutyrate | 0.134 | 0.058 | 0.210 | 0.001 | 0.001 | 0.145 | 0.069 | 0.220 | 0.000 | 0.001 |
| Acetate | -0.096 | -0.172 | -0.020 | 0.013 | 0.019 | -0.067 | -0.142 | 0.008 | 0.082 | 0.163 |
| Acetoacetate | 0.135 | 0.059 | 0.211 | 0.000 | 0.001 | 0.104 | 0.028 | 0.179 | 0.007 | 0.019 |
| Acetone | 0.086 | 0.010 | 0.162 | 0.027 | 0.036 | 0.095 | 0.020 | 0.171 | 0.013 | 0.034 |
| Albumin | -0.022 | -0.098 | 0.054 | 0.572 | 0.598 | 0.029 | -0.048 | 0.105 | 0.463 | 0.642 |
| Creatinine | -0.012 | -0.104 | 0.080 | 0.802 | 0.815 | -0.074 | -0.166 | 0.019 | 0.118 | 0.226 |
| **Lipoprotein particles** |  |  |  |  |  |  |  |  |  |  |
| VS-VLDL | -0.039 | -0.116 | 0.037 | 0.314 | 0.347 | -0.007 | -0.087 | 0.074 | 0.872 | 0.943 |
| S-VLDL | 0.053 | -0.025 | 0.130 | 0.181 | 0.212 | -0.011 | -0.091 | 0.069 | 0.786 | 0.890 |
| M-VLDL | -0.035 | -0.111 | 0.042 | 0.376 | 0.410 | -0.035 | -0.116 | 0.045 | 0.391 | 0.559 |
| L-VLDL | 0.169 | 0.090 | 0.248 | 0.000 | 0.000 | 0.055 | -0.028 | 0.138 | 0.195 | 0.334 |
| VL-VLDL | 0.213 | 0.134 | 0.293 | 0.000 | 0.000 | 0.084 | 0.000 | 0.167 | 0.049 | 0.105 |
| XL-VLDL | 0.257 | 0.178 | 0.335 | 0.000 | 0.000 | 0.125 | 0.043 | 0.206 | 0.003 | 0.009 |
| VLDL | 0.032 | -0.045 | 0.109 | 0.414 | 0.448 | -0.001 | -0.081 | 0.080 | 0.989 | 0.998 |
| S-LDL | -0.089 | -0.165 | -0.012 | 0.023 | 0.031 | -0.057 | -0.138 | 0.024 | 0.168 | 0.297 |
| M-LDL | -0.050 | -0.127 | 0.026 | 0.198 | 0.228 | -0.016 | -0.096 | 0.065 | 0.699 | 0.829 |
| L-LDL | -0.141 | -0.217 | -0.065 | 0.000 | 0.001 | -0.078 | -0.159 | 0.003 | 0.058 | 0.119 |
| LDL | -0.114 | -0.190 | -0.038 | 0.003 | 0.006 | -0.061 | -0.142 | 0.020 | 0.140 | 0.257 |
| IDL | -0.115 | -0.192 | -0.039 | 0.003 | 0.005 | -0.027 | -0.109 | 0.056 | 0.522 | 0.695 |
| S-HDL | 0.250 | 0.174 | 0.326 | 0.000 | 0.000 | 0.210 | 0.134 | 0.287 | 0.000 | 0.000 |
| M-HDL | 0.149 | 0.066 | 0.231 | 0.000 | 0.001 | 0.241 | 0.157 | 0.326 | 0.000 | 0.000 |
| L-HDL | -0.202 | -0.288 | -0.115 | 0.000 | 0.000 | 0.026 | -0.067 | 0.119 | 0.584 | 0.738 |
| XL-HDL | -0.292 | -0.377 | -0.208 | 0.000 | 0.000 | -0.067 | -0.157 | 0.024 | 0.150 | 0.271 |
| HDL | 0.141 | 0.060 | 0.221 | 0.001 | 0.001 | 0.221 | 0.140 | 0.303 | 0.000 | 0.000 |
| Total | 0.117 | 0.036 | 0.197 | 0.004 | 0.007 | 0.203 | 0.122 | 0.284 | 0.000 | 0.000 |
| **Phospholipids** |  |  |  |  |  |  |  |  |  |  |
| VS-VLDL | -0.013 | -0.089 | 0.064 | 0.748 | 0.766 | 0.000 | -0.080 | 0.080 | 1.000 | 1.000 |
| S-VLDL | -0.026 | -0.103 | 0.050 | 0.502 | 0.534 | -0.035 | -0.115 | 0.045 | 0.393 | 0.559 |
| M-VLDL | -0.045 | -0.121 | 0.032 | 0.252 | 0.286 | -0.038 | -0.119 | 0.042 | 0.353 | 0.527 |
| L-VLDL | 0.175 | 0.096 | 0.254 | 0.000 | 0.000 | 0.055 | -0.029 | 0.138 | 0.199 | 0.339 |
| VL-VLDL | 0.187 | 0.108 | 0.266 | 0.000 | 0.000 | 0.070 | -0.013 | 0.153 | 0.099 | 0.191 |
| XL-VLDL | 0.258 | 0.180 | 0.337 | 0.000 | 0.000 | 0.125 | 0.043 | 0.207 | 0.003 | 0.009 |
| VLDL | 0.087 | 0.009 | 0.164 | 0.028 | 0.037 | 0.026 | -0.056 | 0.107 | 0.534 | 0.705 |
| S-LDL | -0.102 | -0.178 | -0.026 | 0.008 | 0.013 | -0.060 | -0.141 | 0.020 | 0.142 | 0.258 |
| M-LDL | -0.019 | -0.095 | 0.057 | 0.624 | 0.645 | 0.004 | -0.076 | 0.084 | 0.916 | 0.957 |
| L-LDL | -0.095 | -0.171 | -0.019 | 0.015 | 0.021 | -0.008 | -0.089 | 0.072 | 0.839 | 0.925 |
| LDL | -0.074 | -0.150 | 0.002 | 0.055 | 0.070 | -0.012 | -0.092 | 0.069 | 0.779 | 0.885 |
| IDL | -0.153 | -0.231 | -0.075 | 0.000 | 0.000 | -0.029 | -0.112 | 0.055 | 0.502 | 0.683 |
| S-HDL | 0.364 | 0.288 | 0.440 | 0.000 | 0.000 | 0.292 | 0.215 | 0.369 | 0.000 | 0.000 |
| M-HDL | 0.248 | 0.167 | 0.330 | 0.000 | 0.000 | 0.283 | 0.201 | 0.365 | 0.000 | 0.000 |
| L-HDL | -0.115 | -0.202 | -0.028 | 0.009 | 0.014 | 0.086 | -0.006 | 0.178 | 0.066 | 0.135 |
| XL-HDL | -0.278 | -0.363 | -0.193 | 0.000 | 0.000 | -0.060 | -0.151 | 0.032 | 0.200 | 0.339 |
| HDL | 0.121 | 0.036 | 0.205 | 0.005 | 0.008 | 0.232 | 0.146 | 0.319 | 0.000 | 0.000 |
| Total | 0.062 | -0.018 | 0.141 | 0.128 | 0.154 | 0.145 | 0.063 | 0.226 | 0.000 | 0.002 |
| **Phospholipids to Total lipids** |  |  |  |  |  |  |  |  |  |  |
| VS-VLDL | 0.107 | 0.030 | 0.184 | 0.006 | 0.010 | -0.043 | -0.122 | 0.035 | 0.280 | 0.438 |
| S-VLDL | -0.312 | -0.390 | -0.234 | 0.000 | 0.000 | -0.119 | -0.202 | -0.036 | 0.005 | 0.015 |
| M-VLDL | -0.288 | -0.366 | -0.210 | 0.000 | 0.000 | -0.103 | -0.188 | -0.019 | 0.016 | 0.040 |
| L-VLDL | 0.146 | 0.068 | 0.225 | 0.000 | 0.000 | 0.023 | -0.058 | 0.104 | 0.576 | 0.731 |
| VL-VLDL | -0.052 | -0.128 | 0.025 | 0.185 | 0.216 | -0.060 | -0.137 | 0.018 | 0.132 | 0.245 |
| XL-VLDL | 0.120 | 0.043 | 0.197 | 0.002 | 0.004 | 0.018 | -0.059 | 0.096 | 0.642 | 0.785 |
| S-LDL | -0.156 | -0.232 | -0.079 | 0.000 | 0.000 | -0.117 | -0.195 | -0.039 | 0.003 | 0.010 |
| M-LDL | -0.083 | -0.159 | -0.007 | 0.033 | 0.043 | -0.083 | -0.159 | -0.008 | 0.031 | 0.073 |
| L-LDL | -0.009 | -0.085 | 0.067 | 0.821 | 0.828 | -0.014 | -0.090 | 0.062 | 0.724 | 0.842 |
| IDL | -0.101 | -0.177 | -0.026 | 0.009 | 0.013 | -0.122 | -0.198 | -0.046 | 0.002 | 0.006 |
| S-HDL | 0.222 | 0.142 | 0.301 | 0.000 | 0.000 | 0.197 | 0.118 | 0.277 | 0.000 | 0.000 |
| M-HDL | 0.289 | 0.210 | 0.369 | 0.000 | 0.000 | 0.088 | 0.002 | 0.173 | 0.044 | 0.096 |
| L-HDL | 0.542 | 0.464 | 0.620 | 0.000 | 0.000 | 0.315 | 0.227 | 0.403 | 0.000 | 0.000 |
| XL-HDL | -0.240 | -0.322 | -0.157 | 0.000 | 0.000 | -0.080 | -0.165 | 0.005 | 0.066 | 0.135 |
| **Total lipids** |  |  |  |  |  |  |  |  |  |  |
| VS-VLDL | -0.026 | -0.104 | 0.051 | 0.500 | 0.534 | 0.004 | -0.077 | 0.084 | 0.928 | 0.957 |
| S-VLDL | 0.052 | -0.025 | 0.129 | 0.186 | 0.216 | -0.004 | -0.084 | 0.077 | 0.930 | 0.957 |
| M-VLDL | 0.012 | -0.065 | 0.089 | 0.769 | 0.784 | -0.016 | -0.096 | 0.065 | 0.703 | 0.829 |
| L-VLDL | 0.160 | 0.081 | 0.239 | 0.000 | 0.000 | 0.048 | -0.034 | 0.131 | 0.252 | 0.411 |
| VL-VLDL | 0.213 | 0.134 | 0.292 | 0.000 | 0.000 | 0.087 | 0.003 | 0.170 | 0.042 | 0.092 |
| XL-VLDL | 0.252 | 0.173 | 0.330 | 0.000 | 0.000 | 0.124 | 0.043 | 0.206 | 0.003 | 0.009 |
| VLDL | 0.130 | 0.052 | 0.207 | 0.001 | 0.002 | 0.047 | -0.034 | 0.129 | 0.257 | 0.415 |
| S-LDL | -0.057 | -0.133 | 0.019 | 0.141 | 0.169 | -0.025 | -0.106 | 0.056 | 0.549 | 0.713 |
| M-LDL | -0.008 | -0.085 | 0.068 | 0.833 | 0.836 | 0.015 | -0.065 | 0.095 | 0.714 | 0.835 |
| L-LDL | -0.092 | -0.169 | -0.016 | 0.018 | 0.025 | -0.008 | -0.089 | 0.072 | 0.837 | 0.925 |
| LDL | -0.066 | -0.142 | 0.010 | 0.088 | 0.109 | -0.004 | -0.084 | 0.076 | 0.927 | 0.957 |
| IDL | -0.127 | -0.205 | -0.049 | 0.001 | 0.002 | -0.003 | -0.087 | 0.081 | 0.944 | 0.967 |
| S-HDL | 0.337 | 0.260 | 0.413 | 0.000 | 0.000 | 0.267 | 0.191 | 0.344 | 0.000 | 0.000 |
| M-HDL | 0.202 | 0.120 | 0.284 | 0.000 | 0.000 | 0.265 | 0.182 | 0.348 | 0.000 | 0.000 |
| L-HDL | -0.167 | -0.254 | -0.080 | 0.000 | 0.000 | 0.051 | -0.041 | 0.144 | 0.279 | 0.438 |
| XL-HDL | -0.292 | -0.377 | -0.207 | 0.000 | 0.000 | -0.066 | -0.158 | 0.025 | 0.153 | 0.275 |
| HDL | 0.060 | -0.025 | 0.145 | 0.167 | 0.197 | 0.203 | 0.116 | 0.290 | 0.000 | 0.000 |
| Total | 0.039 | -0.037 | 0.116 | 0.315 | 0.348 | 0.088 | 0.009 | 0.168 | 0.030 | 0.071 |
| **Triglycerides** |  |  |  |  |  |  |  |  |  |  |
| VS-VLDL | 0.209 | 0.132 | 0.286 | 0.000 | 0.000 | 0.094 | 0.015 | 0.172 | 0.019 | 0.048 |
| S-VLDL | 0.175 | 0.097 | 0.253 | 0.000 | 0.000 | 0.051 | -0.029 | 0.132 | 0.211 | 0.355 |
| M-VLDL | 0.147 | 0.068 | 0.225 | 0.000 | 0.000 | 0.045 | -0.036 | 0.127 | 0.275 | 0.438 |
| L-VLDL | 0.184 | 0.105 | 0.263 | 0.000 | 0.000 | 0.065 | -0.017 | 0.147 | 0.123 | 0.230 |
| XL-VLDL | 0.250 | 0.171 | 0.330 | 0.000 | 0.000 | 0.109 | 0.026 | 0.192 | 0.010 | 0.027 |
| XXL-VLDL | 0.252 | 0.174 | 0.330 | 0.000 | 0.000 | 0.124 | 0.042 | 0.205 | 0.003 | 0.009 |
| VLDL | 0.213 | 0.135 | 0.292 | 0.000 | 0.000 | 0.086 | 0.004 | 0.168 | 0.039 | 0.088 |
| S-LDL | 0.224 | 0.147 | 0.302 | 0.000 | 0.000 | 0.108 | 0.027 | 0.188 | 0.009 | 0.023 |
| M-LDL | 0.246 | 0.169 | 0.324 | 0.000 | 0.000 | 0.133 | 0.053 | 0.212 | 0.001 | 0.004 |
| L-LDL | 0.248 | 0.171 | 0.324 | 0.000 | 0.000 | 0.150 | 0.071 | 0.228 | 0.000 | 0.001 |
| LDL | 0.247 | 0.170 | 0.324 | 0.000 | 0.000 | 0.142 | 0.063 | 0.221 | 0.000 | 0.002 |
| IDL | 0.217 | 0.141 | 0.294 | 0.000 | 0.000 | 0.125 | 0.047 | 0.203 | 0.002 | 0.006 |
| S-HDL | 0.305 | 0.226 | 0.383 | 0.000 | 0.000 | 0.144 | 0.061 | 0.226 | 0.001 | 0.003 |
| M-HDL | 0.274 | 0.198 | 0.350 | 0.000 | 0.000 | 0.166 | 0.089 | 0.243 | 0.000 | 0.000 |
| L-HDL | 0.073 | -0.005 | 0.151 | 0.068 | 0.086 | 0.083 | 0.006 | 0.161 | 0.036 | 0.082 |
| XL-HDL | 0.058 | -0.019 | 0.135 | 0.138 | 0.167 | 0.042 | -0.034 | 0.119 | 0.280 | 0.438 |
| HDL | 0.239 | 0.163 | 0.315 | 0.000 | 0.000 | 0.138 | 0.060 | 0.215 | 0.000 | 0.002 |
| Total | 0.226 | 0.147 | 0.304 | 0.000 | 0.000 | 0.101 | 0.019 | 0.182 | 0.015 | 0.039 |
| **Triglycerides to Total lipids** |  |  |  |  |  |  |  |  |  |  |
| VS-VLDL | 0.411 | 0.332 | 0.490 | 0.000 | 0.000 | 0.185 | 0.098 | 0.272 | 0.000 | 0.000 |
| S-VLDL | 0.302 | 0.225 | 0.379 | 0.000 | 0.000 | 0.127 | 0.046 | 0.209 | 0.002 | 0.007 |
| M-VLDL | 0.363 | 0.284 | 0.443 | 0.000 | 0.000 | 0.148 | 0.062 | 0.234 | 0.001 | 0.003 |
| L-VLDL | 0.075 | -0.001 | 0.151 | 0.053 | 0.069 | 0.052 | -0.024 | 0.128 | 0.180 | 0.314 |
| XL-VLDL | 0.272 | 0.194 | 0.349 | 0.000 | 0.000 | 0.109 | 0.029 | 0.189 | 0.008 | 0.021 |
| XXL-VLDL | 0.014 | -0.062 | 0.090 | 0.720 | 0.741 | -0.005 | -0.081 | 0.070 | 0.891 | 0.952 |
| S-LDL | 0.326 | 0.249 | 0.404 | 0.000 | 0.000 | 0.151 | 0.070 | 0.231 | 0.000 | 0.001 |
| M-LDL | 0.322 | 0.246 | 0.398 | 0.000 | 0.000 | 0.150 | 0.070 | 0.229 | 0.000 | 0.001 |
| L-LDL | 0.368 | 0.291 | 0.444 | 0.000 | 0.000 | 0.166 | 0.085 | 0.247 | 0.000 | 0.000 |
| IDL | 0.370 | 0.292 | 0.448 | 0.000 | 0.000 | 0.148 | 0.064 | 0.232 | 0.001 | 0.002 |
| S-HDL | 0.200 | 0.121 | 0.280 | 0.000 | 0.000 | 0.028 | -0.056 | 0.111 | 0.513 | 0.686 |
| M-HDL | 0.184 | 0.105 | 0.263 | 0.000 | 0.000 | 0.031 | -0.051 | 0.114 | 0.452 | 0.637 |
| L-HDL | 0.196 | 0.116 | 0.276 | 0.000 | 0.000 | 0.024 | -0.059 | 0.107 | 0.570 | 0.731 |
| XL-HDL | 0.325 | 0.246 | 0.404 | 0.000 | 0.000 | 0.144 | 0.062 | 0.227 | 0.001 | 0.003 |

† Model adjusted for age, sex, and education.
‡ Model adjusted for age, sex, education, race, socioeconomic status, body mass index, smoking status, alcohol drinking status, physical activity, social connection, hypertension, diabetes, heart disease, beta-blockers, calcium blockers, lipid-lowering, and APOE ε4 status.
Abbreviation: Apo, apolipoprotein; LDL-AD, average diameter for LDL particles; CHOL, cholesterol; CE, cholesteryl esters; TG, triglycerides; S, small; M, medium; L, large; VL, very large; XL, extremely large; VLDL, very low-density lipoprotein; LDL; low-density lipoprotein; IDL, intermediate-density lipoprotein; HDL, high-density lipoprotein; LA, linoleic acid; MUFA, monounsaturated fatty acid; n-6, omega-6 fatty acid; PUFA, polyunsaturated fatty acid; SFA, saturated fatty acid; FA, fatty acid.

**Table S16.** β coefficients and 95% confidence intervals (CIs) for the association between metabolites and brain age gap (BAG) within eight other candidate machine learning models: results from linear regression models

| **Metabolites** | **1** |  | **2** |  | **3** |  | **4** |  | **5** |  | **6** |  | **7** |  | **8** |  |
| --- | --- | --- | --- | --- | --- | --- | --- | --- | --- | --- | --- | --- | --- | --- | --- | --- |
|  | **β** | **FDR-q** | **β** | **FDR-q** | **β** | **FDR-q** | **β** | **FDR-q** | **β** | **FDR-q** | **β** | **FDR-q** | **β** | **FDR-q** | **β** | **FDR-q** |
| **Amino acids & Glycolysis** | |  |  |  |  |  |  |  |  |  |  |  |  |  |  |  |
| Valine | 0.009 | 0.939 | 0.006 | 0.866 | 0.016 | 0.886 | 0.005 | 0.934 | 0.023 | 0.575 | 0.033 | 0.681 | 0.009 | 0.925 | 0.003 | 0.963 |
| Leucine | -0.002 | 0.988 | -0.004 | 0.913 | 0.017 | 0.886 | -0.009 | 0.863 | 0.012 | 0.772 | 0.025 | 0.733 | -0.009 | 0.925 | -0.006 | 0.925 |
| Isoleucine | -0.029 | 0.689 | -0.026 | 0.522 | -0.018 | 0.876 | -0.025 | 0.633 | -0.007 | 0.848 | -0.014 | 0.839 | -0.038 | 0.611 | -0.041 | 0.487 |
| Phenylalanine | 0.032 | 0.663 | 0.039 | 0.324 | 0.041 | 0.578 | 0.014 | 0.795 | 0.046 | 0.245 | 0.045 | 0.506 | 0.013 | 0.894 | 0.040 | 0.487 |
| Tyrosine | 0.080 | 0.071 | 0.046 | 0.260 | 0.130 | 0.014 | 0.082 | 0.083 | 0.057 | 0.130 | 0.152 | 0.007 | 0.109 | 0.028 | 0.087 | 0.059 |
| Alanine | 0.054 | 0.290 | 0.054 | 0.181 | 0.059 | 0.339 | 0.052 | 0.300 | 0.053 | 0.166 | 0.062 | 0.326 | 0.058 | 0.376 | 0.037 | 0.530 |
| Glutamine | -0.143 | 0.000 | -0.106 | 0.001 | -0.226 | 0.000 | -0.168 | 0.000 | -0.095 | 0.004 | -0.247 | 0.000 | -0.193 | 0.000 | -0.177 | 0.000 |
| Glycine | -0.019 | 0.827 | -0.023 | 0.594 | -0.012 | 0.924 | -0.052 | 0.320 | -0.016 | 0.704 | -0.004 | 0.963 | -0.010 | 0.925 | 0.008 | 0.899 |
| Histidine | -0.044 | 0.443 | -0.015 | 0.685 | -0.065 | 0.265 | -0.044 | 0.360 | -0.006 | 0.862 | -0.059 | 0.334 | -0.056 | 0.386 | -0.041 | 0.472 |
| BCAAs | -0.003 | 0.988 | -0.004 | 0.902 | 0.010 | 0.946 | -0.006 | 0.916 | 0.014 | 0.737 | 0.021 | 0.783 | -0.007 | 0.951 | -0.010 | 0.888 |
| Lactate | 0.080 | 0.070 | 0.108 | 0.001 | 0.117 | 0.025 | 0.073 | 0.132 | 0.103 | 0.001 | 0.132 | 0.017 | 0.097 | 0.053 | 0.118 | 0.003 |
| Pyruvate | 0.069 | 0.140 | 0.056 | 0.149 | 0.097 | 0.064 | 0.061 | 0.217 | 0.060 | 0.097 | 0.102 | 0.065 | 0.068 | 0.247 | 0.071 | 0.142 |
| Glucose | 0.084 | 0.071 | 0.024 | 0.581 | 0.124 | 0.025 | 0.089 | 0.069 | 0.039 | 0.389 | 0.102 | 0.093 | 0.069 | 0.285 | 0.026 | 0.722 |
| Citrate | 0.003 | 0.988 | -0.001 | 0.967 | 0.000 | 0.995 | -0.025 | 0.627 | 0.004 | 0.910 | -0.010 | 0.888 | -0.016 | 0.873 | -0.012 | 0.861 |
| **Apo-LP, LP size & Other lipids** | | |  |  |  |  |  |  |  |  |  |  |  |  |  |  |
| ApoA1 | 0.135 | 0.002 | 0.129 | 0.000 | 0.189 | 0.001 | 0.154 | 0.001 | 0.119 | 0.001 | 0.208 | 0.000 | 0.138 | 0.008 | 0.175 | 0.000 |
| ApoB | -0.005 | 0.977 | 0.018 | 0.673 | -0.041 | 0.596 | 0.030 | 0.578 | 0.011 | 0.785 | -0.067 | 0.325 | -0.002 | 0.981 | -0.003 | 0.963 |
| ApoB/ApoA1 | -0.072 | 0.176 | -0.050 | 0.260 | -0.138 | 0.017 | -0.052 | 0.333 | -0.052 | 0.230 | -0.167 | 0.006 | -0.069 | 0.314 | -0.090 | 0.073 |
| HDL-AD | 0.019 | 0.865 | -0.007 | 0.859 | -0.001 | 0.995 | 0.003 | 0.964 | -0.010 | 0.821 | 0.008 | 0.936 | -0.013 | 0.922 | 0.019 | 0.816 |
| LDL-AD | -0.005 | 0.968 | -0.008 | 0.827 | -0.086 | 0.136 | -0.007 | 0.909 | -0.010 | 0.794 | -0.098 | 0.098 | -0.024 | 0.784 | -0.027 | 0.702 |
| VLDL-AD | 0.009 | 0.939 | 0.021 | 0.640 | 0.051 | 0.522 | 0.025 | 0.686 | 0.029 | 0.530 | 0.060 | 0.425 | 0.018 | 0.873 | 0.023 | 0.780 |
| Phosphatidylcholine | 0.102 | 0.024 | 0.108 | 0.002 | 0.141 | 0.013 | 0.141 | 0.002 | 0.103 | 0.004 | 0.139 | 0.019 | 0.107 | 0.048 | 0.137 | 0.001 |
| Phosphoglycerides | 0.115 | 0.007 | 0.120 | 0.000 | 0.160 | 0.004 | 0.158 | 0.000 | 0.116 | 0.001 | 0.159 | 0.007 | 0.122 | 0.017 | 0.148 | 0.000 |
| Sphingomyeline | 0.095 | 0.048 | 0.096 | 0.010 | 0.081 | 0.205 | 0.115 | 0.019 | 0.084 | 0.030 | 0.068 | 0.326 | 0.091 | 0.130 | 0.096 | 0.057 |
| Total choline | 0.104 | 0.023 | 0.110 | 0.002 | 0.135 | 0.017 | 0.143 | 0.002 | 0.103 | 0.004 | 0.132 | 0.026 | 0.107 | 0.048 | 0.134 | 0.002 |
| TG/Phosphoglycerides | -0.001 | 0.994 | 0.014 | 0.737 | 0.032 | 0.731 | 0.015 | 0.818 | 0.023 | 0.607 | 0.026 | 0.763 | -0.002 | 0.980 | 0.001 | 0.991 |
| **Cholesterol** |  |  |  |  |  |  |  |  |  |  |  |  |  |  |  |  |
| XS-VLDL | 0.003 | 0.988 | 0.022 | 0.618 | -0.026 | 0.787 | 0.056 | 0.302 | 0.021 | 0.624 | -0.065 | 0.334 | 0.013 | 0.908 | 0.022 | 0.780 |
| S-VLDL | -0.022 | 0.799 | 0.010 | 0.808 | -0.045 | 0.563 | 0.019 | 0.739 | 0.009 | 0.832 | -0.081 | 0.208 | -0.016 | 0.873 | -0.012 | 0.861 |
| M-VLDL | -0.046 | 0.458 | -0.015 | 0.712 | -0.100 | 0.082 | -0.013 | 0.821 | -0.021 | 0.608 | -0.130 | 0.027 | -0.042 | 0.577 | -0.038 | 0.551 |
| L-VLDL | -0.013 | 0.901 | 0.014 | 0.735 | -0.002 | 0.995 | 0.018 | 0.765 | 0.017 | 0.704 | -0.022 | 0.786 | -0.009 | 0.925 | -0.005 | 0.948 |
| VL-VLDL | 0.002 | 0.988 | 0.025 | 0.581 | 0.019 | 0.876 | 0.034 | 0.530 | 0.027 | 0.534 | 0.003 | 0.969 | 0.007 | 0.951 | 0.010 | 0.888 |
| XL-VLDL | 0.037 | 0.623 | 0.048 | 0.271 | 0.096 | 0.103 | 0.073 | 0.170 | 0.054 | 0.202 | 0.094 | 0.131 | 0.045 | 0.554 | 0.051 | 0.402 |
| VLDL | -0.012 | 0.901 | 0.016 | 0.701 | -0.024 | 0.818 | 0.031 | 0.576 | 0.015 | 0.719 | -0.053 | 0.462 | -0.006 | 0.951 | 0.000 | 0.997 |
| S-LDL | 0.012 | 0.901 | 0.030 | 0.497 | -0.019 | 0.876 | 0.038 | 0.484 | 0.020 | 0.627 | -0.032 | 0.681 | 0.015 | 0.891 | 0.009 | 0.899 |
| M-LDL | 0.027 | 0.729 | 0.044 | 0.307 | 0.010 | 0.941 | 0.056 | 0.296 | 0.034 | 0.431 | -0.003 | 0.969 | 0.033 | 0.679 | 0.029 | 0.689 |
| L-LDL | 0.024 | 0.751 | 0.038 | 0.387 | -0.015 | 0.889 | 0.048 | 0.352 | 0.027 | 0.530 | -0.033 | 0.681 | 0.025 | 0.780 | 0.025 | 0.722 |
| Clinical LDL | 0.015 | 0.896 | 0.034 | 0.452 | -0.022 | 0.839 | 0.045 | 0.398 | 0.022 | 0.593 | -0.038 | 0.629 | 0.023 | 0.808 | 0.020 | 0.780 |
| LDL | 0.024 | 0.751 | 0.039 | 0.363 | -0.009 | 0.953 | 0.050 | 0.334 | 0.028 | 0.513 | -0.025 | 0.740 | 0.027 | 0.780 | 0.025 | 0.722 |
| IDL | 0.029 | 0.724 | 0.048 | 0.281 | -0.002 | 0.995 | 0.072 | 0.186 | 0.037 | 0.418 | -0.015 | 0.846 | 0.046 | 0.554 | 0.051 | 0.413 |
| S-HDL | 0.120 | 0.002 | 0.132 | 0.000 | 0.180 | 0.001 | 0.145 | 0.001 | 0.121 | 0.000 | 0.193 | 0.000 | 0.145 | 0.002 | 0.155 | 0.000 |
| M-HDL | 0.137 | 0.002 | 0.127 | 0.000 | 0.194 | 0.001 | 0.150 | 0.002 | 0.116 | 0.001 | 0.217 | 0.000 | 0.144 | 0.006 | 0.176 | 0.000 |
| L-HDL | 0.035 | 0.686 | 0.013 | 0.779 | 0.006 | 0.995 | 0.019 | 0.786 | 0.002 | 0.964 | 0.019 | 0.829 | 0.006 | 0.951 | 0.037 | 0.645 |
| XL-HDL | 0.000 | 0.998 | -0.027 | 0.581 | -0.066 | 0.380 | -0.019 | 0.779 | -0.036 | 0.467 | -0.064 | 0.410 | -0.039 | 0.675 | -0.018 | 0.820 |
| HDL | 0.104 | 0.037 | 0.090 | 0.028 | 0.122 | 0.050 | 0.106 | 0.049 | 0.077 | 0.067 | 0.142 | 0.028 | 0.095 | 0.138 | 0.129 | 0.009 |
| Total | 0.046 | 0.458 | 0.061 | 0.149 | 0.026 | 0.792 | 0.081 | 0.123 | 0.049 | 0.252 | 0.013 | 0.858 | 0.050 | 0.533 | 0.062 | 0.278 |
| Remnant | 0.006 | 0.950 | 0.033 | 0.469 | -0.015 | 0.889 | 0.054 | 0.320 | 0.027 | 0.530 | -0.039 | 0.629 | 0.019 | 0.869 | 0.024 | 0.742 |
| Total-HDLC | 0.016 | 0.871 | 0.037 | 0.404 | -0.012 | 0.924 | 0.053 | 0.320 | 0.028 | 0.513 | -0.032 | 0.681 | 0.023 | 0.808 | 0.025 | 0.722 |
| **Cholesterol to Total lipids** | |  |  |  |  |  |  |  |  |  |  |  |  |  |  |  |
| VS-VLDL | -0.060 | 0.314 | -0.057 | 0.230 | -0.145 | 0.017 | -0.063 | 0.281 | -0.075 | 0.067 | -0.142 | 0.026 | -0.052 | 0.534 | -0.050 | 0.451 |
| S-VLDL | -0.045 | 0.465 | -0.030 | 0.497 | -0.122 | 0.027 | -0.035 | 0.516 | -0.042 | 0.346 | -0.139 | 0.019 | -0.048 | 0.534 | -0.051 | 0.399 |
| M-VLDL | -0.064 | 0.273 | -0.051 | 0.272 | -0.149 | 0.013 | -0.063 | 0.276 | -0.065 | 0.130 | -0.157 | 0.014 | -0.076 | 0.281 | -0.069 | 0.251 |
| L-VLDL | -0.061 | 0.235 | -0.038 | 0.363 | -0.102 | 0.064 | -0.029 | 0.578 | -0.035 | 0.416 | -0.136 | 0.018 | -0.073 | 0.241 | -0.062 | 0.255 |
| XL-VLDL | -0.033 | 0.674 | -0.019 | 0.648 | -0.096 | 0.098 | -0.044 | 0.412 | -0.025 | 0.550 | -0.109 | 0.072 | -0.061 | 0.386 | -0.043 | 0.489 |
| XXL-VLDL | 0.010 | 0.925 | 0.023 | 0.565 | -0.008 | 0.964 | 0.002 | 0.975 | 0.023 | 0.561 | -0.025 | 0.733 | -0.006 | 0.951 | -0.017 | 0.790 |
| S-LDL | -0.032 | 0.674 | -0.007 | 0.834 | -0.036 | 0.639 | -0.007 | 0.909 | -0.021 | 0.603 | -0.031 | 0.681 | -0.009 | 0.925 | 0.001 | 0.991 |
| M-LDL | -0.068 | 0.181 | -0.056 | 0.182 | -0.102 | 0.066 | -0.069 | 0.179 | -0.072 | 0.050 | -0.094 | 0.115 | -0.049 | 0.527 | -0.047 | 0.413 |
| L-LDL | -0.067 | 0.210 | -0.066 | 0.108 | -0.148 | 0.008 | -0.098 | 0.044 | -0.088 | 0.015 | -0.142 | 0.016 | -0.081 | 0.176 | -0.072 | 0.175 |
| IDL | -0.030 | 0.690 | -0.013 | 0.748 | -0.060 | 0.371 | -0.032 | 0.559 | -0.032 | 0.467 | -0.027 | 0.733 | 0.006 | 0.951 | -0.001 | 0.991 |
| S-HDL | -0.058 | 0.253 | -0.056 | 0.157 | -0.131 | 0.014 | -0.091 | 0.054 | -0.073 | 0.041 | -0.124 | 0.026 | -0.068 | 0.270 | -0.086 | 0.062 |
| M-HDL | -0.008 | 0.939 | -0.021 | 0.625 | -0.059 | 0.405 | -0.047 | 0.397 | -0.040 | 0.397 | -0.042 | 0.610 | -0.026 | 0.782 | -0.021 | 0.780 |
| L-HDL | -0.077 | 0.169 | -0.097 | 0.014 | -0.172 | 0.005 | -0.111 | 0.036 | -0.111 | 0.004 | -0.173 | 0.007 | -0.097 | 0.122 | -0.109 | 0.035 |
| XL-HDL | 0.027 | 0.729 | 0.040 | 0.363 | 0.027 | 0.770 | 0.024 | 0.680 | 0.037 | 0.408 | 0.026 | 0.733 | 0.041 | 0.609 | 0.020 | 0.780 |
| **Cholesteryl esters to Total lipids** | | |  |  |  |  |  |  |  |  |  |  |  |  |  |  |
| VS-VLDL | -0.055 | 0.376 | -0.054 | 0.260 | -0.134 | 0.026 | -0.055 | 0.333 | -0.071 | 0.091 | -0.128 | 0.049 | -0.046 | 0.577 | -0.044 | 0.530 |
| S-VLDL | -0.047 | 0.417 | -0.029 | 0.497 | -0.108 | 0.050 | -0.022 | 0.688 | -0.035 | 0.418 | -0.134 | 0.020 | -0.044 | 0.554 | -0.048 | 0.403 |
| M-VLDL | -0.066 | 0.253 | -0.053 | 0.260 | -0.153 | 0.011 | -0.070 | 0.226 | -0.067 | 0.111 | -0.157 | 0.014 | -0.079 | 0.247 | -0.073 | 0.208 |
| L-VLDL | -0.076 | 0.143 | -0.052 | 0.246 | -0.140 | 0.014 | -0.056 | 0.302 | -0.053 | 0.205 | -0.170 | 0.004 | -0.098 | 0.084 | -0.077 | 0.147 |
| XL-VLDL | -0.025 | 0.743 | -0.009 | 0.821 | -0.085 | 0.154 | -0.042 | 0.433 | -0.016 | 0.704 | -0.093 | 0.131 | -0.054 | 0.501 | -0.034 | 0.632 |
| XXL-VLDL | 0.016 | 0.851 | 0.027 | 0.497 | -0.013 | 0.908 | 0.003 | 0.956 | 0.027 | 0.511 | -0.032 | 0.666 | -0.001 | 0.981 | -0.014 | 0.830 |
| S-LDL | 0.022 | 0.793 | 0.041 | 0.325 | 0.081 | 0.159 | 0.080 | 0.103 | 0.043 | 0.319 | 0.078 | 0.208 | 0.035 | 0.650 | 0.065 | 0.222 |
| M-LDL | 0.003 | 0.988 | 0.011 | 0.778 | 0.048 | 0.506 | 0.040 | 0.445 | 0.014 | 0.726 | 0.046 | 0.519 | 0.019 | 0.861 | 0.032 | 0.650 |
| L-LDL | -0.030 | 0.674 | -0.036 | 0.377 | -0.079 | 0.151 | -0.055 | 0.275 | -0.046 | 0.233 | -0.086 | 0.131 | -0.056 | 0.386 | -0.050 | 0.364 |
| IDL | -0.009 | 0.939 | 0.017 | 0.683 | -0.021 | 0.846 | -0.008 | 0.886 | 0.000 | 0.990 | 0.020 | 0.801 | 0.029 | 0.733 | 0.022 | 0.769 |
| S-HDL | -0.058 | 0.252 | -0.053 | 0.189 | -0.111 | 0.033 | -0.086 | 0.065 | -0.068 | 0.057 | -0.102 | 0.070 | -0.057 | 0.390 | -0.077 | 0.103 |
| M-HDL | -0.026 | 0.734 | -0.036 | 0.420 | -0.078 | 0.207 | -0.065 | 0.228 | -0.053 | 0.205 | -0.063 | 0.340 | -0.041 | 0.609 | -0.040 | 0.530 |
| L-HDL | -0.090 | 0.071 | -0.101 | 0.008 | -0.180 | 0.002 | -0.122 | 0.015 | -0.115 | 0.002 | -0.182 | 0.003 | -0.109 | 0.057 | -0.115 | 0.018 |
| XL-HDL | 0.029 | 0.686 | 0.032 | 0.456 | 0.018 | 0.876 | 0.015 | 0.791 | 0.024 | 0.555 | 0.019 | 0.801 | 0.032 | 0.679 | 0.012 | 0.861 |
| **Cholestryl esters** |  |  |  |  |  |  |  |  |  |  |  |  |  |  |  |  |
| VS-VLDL | 0.000 | 0.998 | 0.018 | 0.666 | -0.033 | 0.698 | 0.054 | 0.320 | 0.016 | 0.712 | -0.071 | 0.308 | 0.012 | 0.922 | 0.020 | 0.780 |
| S-VLDL | -0.021 | 0.812 | 0.011 | 0.779 | -0.039 | 0.626 | 0.023 | 0.688 | 0.011 | 0.785 | -0.076 | 0.246 | -0.015 | 0.888 | -0.010 | 0.888 |
| M-VLDL | -0.057 | 0.314 | -0.026 | 0.561 | -0.125 | 0.026 | -0.026 | 0.641 | -0.034 | 0.445 | -0.156 | 0.009 | -0.054 | 0.506 | -0.050 | 0.403 |
| L-VLDL | -0.034 | 0.669 | -0.002 | 0.959 | -0.037 | 0.639 | 0.000 | 0.999 | 0.000 | 0.990 | -0.064 | 0.334 | -0.031 | 0.701 | -0.023 | 0.754 |
| VL-VLDL | -0.009 | 0.939 | 0.020 | 0.645 | -0.007 | 0.983 | 0.023 | 0.690 | 0.020 | 0.627 | -0.029 | 0.715 | -0.004 | 0.952 | 0.000 | 0.997 |
| XL-VLDL | 0.037 | 0.623 | 0.052 | 0.246 | 0.092 | 0.126 | 0.071 | 0.182 | 0.056 | 0.185 | 0.086 | 0.184 | 0.045 | 0.560 | 0.050 | 0.405 |
| VLDL | -0.021 | 0.812 | 0.010 | 0.808 | -0.042 | 0.592 | 0.024 | 0.668 | 0.008 | 0.832 | -0.075 | 0.257 | -0.014 | 0.904 | -0.007 | 0.916 |
| S-LDL | 0.020 | 0.824 | 0.037 | 0.397 | 0.001 | 0.995 | 0.051 | 0.332 | 0.029 | 0.509 | -0.013 | 0.858 | 0.021 | 0.840 | 0.019 | 0.780 |
| M-LDL | 0.032 | 0.674 | 0.049 | 0.260 | 0.025 | 0.797 | 0.064 | 0.228 | 0.040 | 0.364 | 0.012 | 0.863 | 0.039 | 0.616 | 0.036 | 0.587 |
| L-LDL | 0.026 | 0.732 | 0.039 | 0.363 | -0.010 | 0.941 | 0.050 | 0.333 | 0.029 | 0.511 | -0.029 | 0.709 | 0.026 | 0.780 | 0.026 | 0.722 |
| LDL | 0.028 | 0.724 | 0.042 | 0.324 | 0.000 | 0.998 | 0.055 | 0.301 | 0.032 | 0.462 | -0.017 | 0.829 | 0.030 | 0.726 | 0.028 | 0.697 |
| IDL | 0.032 | 0.686 | 0.053 | 0.246 | 0.003 | 0.995 | 0.075 | 0.167 | 0.042 | 0.364 | -0.007 | 0.932 | 0.050 | 0.534 | 0.055 | 0.384 |
| S-HDL | 0.109 | 0.007 | 0.122 | 0.000 | 0.167 | 0.001 | 0.132 | 0.002 | 0.111 | 0.001 | 0.182 | 0.001 | 0.137 | 0.003 | 0.143 | 0.000 |
| M-HDL | 0.135 | 0.002 | 0.125 | 0.000 | 0.192 | 0.001 | 0.147 | 0.002 | 0.114 | 0.002 | 0.216 | 0.000 | 0.142 | 0.006 | 0.174 | 0.000 |
| L-HDL | 0.030 | 0.734 | 0.009 | 0.834 | -0.001 | 0.995 | 0.012 | 0.854 | -0.002 | 0.973 | 0.012 | 0.883 | 0.000 | 0.996 | 0.032 | 0.697 |
| XL-HDL | 0.002 | 0.988 | -0.023 | 0.625 | -0.061 | 0.439 | -0.018 | 0.790 | -0.034 | 0.502 | -0.058 | 0.483 | -0.037 | 0.685 | -0.014 | 0.861 |
| HDL | 0.100 | 0.049 | 0.086 | 0.037 | 0.117 | 0.061 | 0.100 | 0.066 | 0.072 | 0.090 | 0.138 | 0.033 | 0.092 | 0.165 | 0.124 | 0.012 |
| Total | 0.053 | 0.367 | 0.066 | 0.111 | 0.034 | 0.689 | 0.086 | 0.094 | 0.054 | 0.205 | 0.024 | 0.764 | 0.057 | 0.463 | 0.069 | 0.211 |
| **Fatty acids** |  |  |  |  |  |  |  |  |  |  |  |  |  |  |  |  |
| DHA | -0.001 | 0.994 | 0.005 | 0.868 | 0.031 | 0.698 | 0.019 | 0.732 | 0.010 | 0.794 | 0.000 | 0.997 | 0.002 | 0.980 | 0.000 | 0.997 |
| LA | -0.039 | 0.548 | -0.019 | 0.648 | -0.059 | 0.357 | -0.002 | 0.975 | -0.025 | 0.550 | -0.063 | 0.334 | -0.052 | 0.506 | -0.027 | 0.701 |
| MUFA | 0.100 | 0.024 | 0.107 | 0.002 | 0.152 | 0.006 | 0.134 | 0.003 | 0.112 | 0.001 | 0.149 | 0.011 | 0.094 | 0.089 | 0.105 | 0.020 |
| n-3 | 0.015 | 0.881 | 0.023 | 0.581 | 0.060 | 0.331 | 0.047 | 0.346 | 0.032 | 0.452 | 0.034 | 0.662 | 0.020 | 0.840 | 0.022 | 0.747 |
| n-6 | 0.003 | 0.988 | 0.019 | 0.640 | -0.003 | 0.995 | 0.046 | 0.365 | 0.015 | 0.707 | -0.006 | 0.940 | -0.001 | 0.984 | 0.021 | 0.777 |
| PUFA | 0.007 | 0.946 | 0.023 | 0.589 | 0.015 | 0.889 | 0.052 | 0.308 | 0.022 | 0.587 | 0.005 | 0.953 | 0.005 | 0.951 | 0.024 | 0.722 |
| SFA | 0.130 | 0.002 | 0.131 | 0.000 | 0.187 | 0.001 | 0.171 | 0.000 | 0.131 | 0.000 | 0.187 | 0.001 | 0.141 | 0.003 | 0.154 | 0.000 |
| Total | 0.086 | 0.055 | 0.094 | 0.006 | 0.128 | 0.018 | 0.129 | 0.003 | 0.096 | 0.005 | 0.124 | 0.030 | 0.088 | 0.115 | 0.103 | 0.020 |
| Unsaturation | -0.111 | 0.012 | -0.109 | 0.002 | -0.144 | 0.010 | -0.094 | 0.055 | -0.107 | 0.002 | -0.155 | 0.009 | -0.108 | 0.047 | -0.108 | 0.019 |
| DHA/FA | -0.045 | 0.458 | -0.048 | 0.260 | -0.031 | 0.700 | -0.046 | 0.375 | -0.045 | 0.291 | -0.056 | 0.410 | -0.041 | 0.577 | -0.057 | 0.310 |
| LA/FA | -0.232 | 0.000 | -0.209 | 0.000 | -0.358 | 0.000 | -0.254 | 0.000 | -0.226 | 0.000 | -0.351 | 0.000 | -0.271 | 0.000 | -0.248 | 0.000 |
| MUFA/FA | 0.102 | 0.033 | 0.111 | 0.002 | 0.163 | 0.006 | 0.115 | 0.022 | 0.124 | 0.001 | 0.156 | 0.014 | 0.086 | 0.176 | 0.091 | 0.080 |
| n-3/FA | -0.020 | 0.812 | -0.019 | 0.634 | 0.015 | 0.886 | 0.001 | 0.991 | -0.010 | 0.793 | -0.009 | 0.896 | -0.010 | 0.923 | -0.018 | 0.785 |
| n-6/n-3 | 0.037 | 0.557 | 0.025 | 0.541 | -0.001 | 0.995 | 0.004 | 0.953 | 0.016 | 0.693 | 0.022 | 0.764 | 0.038 | 0.611 | 0.031 | 0.650 |
| n-6/FA | -0.192 | 0.000 | -0.186 | 0.000 | -0.305 | 0.000 | -0.222 | 0.000 | -0.197 | 0.000 | -0.295 | 0.000 | -0.212 | 0.000 | -0.209 | 0.000 |
| PUFA/MUFA | -0.138 | 0.002 | -0.147 | 0.000 | -0.211 | 0.000 | -0.154 | 0.001 | -0.159 | 0.000 | -0.204 | 0.001 | -0.138 | 0.008 | -0.147 | 0.001 |
| PUFA/FA | -0.200 | 0.000 | -0.194 | 0.000 | -0.294 | 0.000 | -0.220 | 0.000 | -0.200 | 0.000 | -0.296 | 0.000 | -0.215 | 0.000 | -0.216 | 0.000 |
| SFA/FA | 0.209 | 0.000 | 0.188 | 0.000 | 0.292 | 0.000 | 0.227 | 0.000 | 0.185 | 0.000 | 0.304 | 0.000 | 0.249 | 0.000 | 0.245 | 0.000 |
| **Free cholesterol** |  |  |  |  |  |  |  |  |  |  |  |  |  |  |  |  |
| VS-VLDL | 0.009 | 0.937 | 0.029 | 0.498 | -0.010 | 0.941 | 0.059 | 0.276 | 0.031 | 0.484 | -0.050 | 0.485 | 0.016 | 0.873 | 0.025 | 0.722 |
| S-VLDL | -0.022 | 0.793 | 0.008 | 0.836 | -0.055 | 0.415 | 0.012 | 0.837 | 0.003 | 0.933 | -0.088 | 0.158 | -0.019 | 0.869 | -0.015 | 0.830 |
| M-VLDL | -0.029 | 0.690 | 0.000 | 0.995 | -0.062 | 0.347 | 0.004 | 0.953 | -0.004 | 0.915 | -0.090 | 0.143 | -0.026 | 0.780 | -0.021 | 0.780 |
| L-VLDL | 0.008 | 0.939 | 0.029 | 0.518 | 0.033 | 0.698 | 0.036 | 0.516 | 0.032 | 0.480 | 0.020 | 0.801 | 0.012 | 0.921 | 0.014 | 0.851 |
| VL-VLDL | 0.013 | 0.901 | 0.029 | 0.518 | 0.045 | 0.578 | 0.045 | 0.412 | 0.033 | 0.463 | 0.036 | 0.666 | 0.019 | 0.873 | 0.021 | 0.780 |
| XL-VLDL | 0.037 | 0.623 | 0.043 | 0.324 | 0.099 | 0.086 | 0.073 | 0.167 | 0.051 | 0.230 | 0.102 | 0.094 | 0.045 | 0.554 | 0.051 | 0.399 |
| VLDL | -0.001 | 0.994 | 0.023 | 0.600 | 0.002 | 0.995 | 0.038 | 0.484 | 0.024 | 0.572 | -0.021 | 0.786 | 0.005 | 0.951 | 0.010 | 0.890 |
| S-LDL | -0.008 | 0.939 | 0.006 | 0.856 | -0.068 | 0.281 | 0.000 | 0.999 | -0.007 | 0.862 | -0.080 | 0.211 | -0.005 | 0.951 | -0.019 | 0.780 |
| M-LDL | 0.012 | 0.910 | 0.029 | 0.499 | -0.029 | 0.731 | 0.031 | 0.569 | 0.016 | 0.704 | -0.042 | 0.581 | 0.017 | 0.873 | 0.010 | 0.888 |
| L-LDL | 0.018 | 0.847 | 0.033 | 0.464 | -0.029 | 0.749 | 0.042 | 0.433 | 0.020 | 0.627 | -0.043 | 0.581 | 0.021 | 0.840 | 0.023 | 0.763 |
| LDL | 0.014 | 0.896 | 0.029 | 0.497 | -0.033 | 0.690 | 0.035 | 0.515 | 0.016 | 0.704 | -0.047 | 0.523 | 0.017 | 0.873 | 0.015 | 0.830 |
| IDL | 0.019 | 0.833 | 0.033 | 0.476 | -0.017 | 0.886 | 0.061 | 0.276 | 0.023 | 0.596 | -0.036 | 0.666 | 0.033 | 0.700 | 0.037 | 0.600 |
| S-HDL | 0.136 | 0.001 | 0.144 | 0.000 | 0.194 | 0.000 | 0.167 | 0.000 | 0.136 | 0.000 | 0.199 | 0.000 | 0.148 | 0.002 | 0.168 | 0.000 |
| M-HDL | 0.141 | 0.002 | 0.130 | 0.000 | 0.195 | 0.001 | 0.158 | 0.001 | 0.121 | 0.001 | 0.214 | 0.000 | 0.145 | 0.006 | 0.177 | 0.000 |
| L-HDL | 0.051 | 0.464 | 0.025 | 0.612 | 0.029 | 0.787 | 0.041 | 0.506 | 0.017 | 0.726 | 0.041 | 0.666 | 0.023 | 0.840 | 0.054 | 0.428 |
| XL-HDL | -0.008 | 0.945 | -0.037 | 0.445 | -0.079 | 0.246 | -0.022 | 0.728 | -0.042 | 0.389 | -0.082 | 0.252 | -0.044 | 0.609 | -0.030 | 0.700 |
| HDL | 0.115 | 0.019 | 0.098 | 0.012 | 0.134 | 0.027 | 0.124 | 0.016 | 0.088 | 0.031 | 0.148 | 0.022 | 0.103 | 0.092 | 0.137 | 0.004 |
| Total | 0.029 | 0.697 | 0.048 | 0.275 | 0.005 | 0.995 | 0.067 | 0.217 | 0.038 | 0.401 | -0.014 | 0.855 | 0.032 | 0.700 | 0.044 | 0.487 |
| **Free cholesterol to Total lipids** | |  |  |  |  |  |  |  |  |  |  |  |  |  |  |  |
| VS-VLDL | -0.070 | 0.166 | -0.059 | 0.149 | -0.161 | 0.003 | -0.085 | 0.084 | -0.073 | 0.048 | -0.177 | 0.002 | -0.067 | 0.291 | -0.068 | 0.195 |
| S-VLDL | -0.033 | 0.674 | -0.027 | 0.561 | -0.122 | 0.034 | -0.046 | 0.397 | -0.045 | 0.322 | -0.122 | 0.049 | -0.046 | 0.554 | -0.046 | 0.472 |
| M-VLDL | -0.055 | 0.367 | -0.042 | 0.363 | -0.131 | 0.026 | -0.040 | 0.484 | -0.054 | 0.224 | -0.147 | 0.019 | -0.062 | 0.426 | -0.053 | 0.403 |
| L-VLDL | -0.019 | 0.812 | -0.002 | 0.953 | -0.001 | 0.995 | 0.032 | 0.523 | 0.010 | 0.794 | -0.036 | 0.629 | -0.006 | 0.951 | -0.018 | 0.780 |
| XL-VLDL | -0.050 | 0.371 | -0.048 | 0.260 | -0.105 | 0.061 | -0.036 | 0.506 | -0.050 | 0.224 | -0.128 | 0.027 | -0.068 | 0.291 | -0.063 | 0.256 |
| XXL-VLDL | -0.007 | 0.939 | 0.008 | 0.834 | 0.006 | 0.983 | -0.002 | 0.969 | 0.008 | 0.832 | -0.002 | 0.969 | -0.013 | 0.896 | -0.019 | 0.780 |
| S-LDL | -0.057 | 0.289 | -0.053 | 0.211 | -0.128 | 0.020 | -0.096 | 0.045 | -0.069 | 0.062 | -0.120 | 0.038 | -0.049 | 0.533 | -0.071 | 0.175 |
| M-LDL | -0.063 | 0.253 | -0.062 | 0.149 | -0.143 | 0.013 | -0.105 | 0.034 | -0.079 | 0.040 | -0.135 | 0.026 | -0.065 | 0.369 | -0.077 | 0.151 |
| L-LDL | -0.061 | 0.290 | -0.053 | 0.257 | -0.121 | 0.040 | -0.077 | 0.165 | -0.072 | 0.071 | -0.103 | 0.106 | -0.049 | 0.537 | -0.043 | 0.514 |
| IDL | -0.052 | 0.351 | -0.063 | 0.111 | -0.097 | 0.079 | -0.058 | 0.274 | -0.074 | 0.042 | -0.101 | 0.085 | -0.044 | 0.554 | -0.046 | 0.426 |
| S-HDL | 0.005 | 0.979 | -0.009 | 0.821 | -0.067 | 0.305 | -0.010 | 0.856 | -0.015 | 0.719 | -0.080 | 0.224 | -0.040 | 0.616 | -0.029 | 0.697 |
| M-HDL | 0.079 | 0.169 | 0.060 | 0.211 | 0.067 | 0.360 | 0.065 | 0.278 | 0.046 | 0.346 | 0.081 | 0.272 | 0.058 | 0.508 | 0.079 | 0.188 |
| L-HDL | 0.021 | 0.827 | -0.020 | 0.648 | -0.038 | 0.672 | 0.003 | 0.964 | -0.028 | 0.547 | -0.034 | 0.686 | 0.010 | 0.925 | -0.015 | 0.851 |
| XL-HDL | 0.009 | 0.939 | 0.028 | 0.565 | 0.025 | 0.846 | 0.023 | 0.728 | 0.035 | 0.484 | 0.021 | 0.802 | 0.029 | 0.780 | 0.020 | 0.790 |
| **KB, FB & Inflammation** | |  |  |  |  |  |  |  |  |  |  |  |  |  |  |  |
| Glycoprotein acetyls | 0.102 | 0.024 | 0.090 | 0.012 | 0.164 | 0.003 | 0.134 | 0.003 | 0.090 | 0.012 | 0.161 | 0.006 | 0.101 | 0.059 | 0.090 | 0.062 |
| 3-Hydrocybutyrate | 0.090 | 0.033 | 0.061 | 0.108 | 0.137 | 0.008 | 0.087 | 0.055 | 0.058 | 0.111 | 0.134 | 0.015 | 0.089 | 0.084 | 0.057 | 0.274 |
| Acetate | -0.034 | 0.569 | -0.026 | 0.497 | -0.055 | 0.352 | -0.037 | 0.442 | -0.022 | 0.565 | -0.041 | 0.531 | -0.033 | 0.650 | -0.038 | 0.489 |
| Acetoacetate | 0.068 | 0.152 | 0.038 | 0.350 | 0.124 | 0.017 | 0.077 | 0.101 | 0.041 | 0.319 | 0.123 | 0.026 | 0.069 | 0.246 | 0.043 | 0.451 |
| Acetone | 0.059 | 0.235 | 0.024 | 0.561 | 0.102 | 0.050 | 0.033 | 0.511 | 0.028 | 0.500 | 0.114 | 0.038 | 0.048 | 0.508 | 0.031 | 0.637 |
| Albumin | 0.004 | 0.988 | 0.014 | 0.721 | 0.002 | 0.995 | 0.014 | 0.802 | 0.010 | 0.794 | 0.015 | 0.837 | 0.005 | 0.951 | 0.005 | 0.942 |
| Creatinine | -0.083 | 0.156 | -0.034 | 0.497 | -0.088 | 0.209 | -0.107 | 0.055 | -0.013 | 0.785 | -0.075 | 0.326 | -0.132 | 0.026 | -0.069 | 0.279 |
| **Lipoprotein particles** | |  |  |  |  |  |  |  |  |  |  |  |  |  |  |  |
| VS-VLDL | 0.014 | 0.896 | 0.036 | 0.422 | 0.002 | 0.995 | 0.064 | 0.228 | 0.037 | 0.401 | -0.035 | 0.666 | 0.021 | 0.840 | 0.030 | 0.679 |
| S-VLDL | -0.013 | 0.896 | 0.016 | 0.701 | -0.011 | 0.933 | 0.019 | 0.732 | 0.019 | 0.649 | -0.040 | 0.611 | -0.015 | 0.888 | -0.006 | 0.925 |
| M-VLDL | -0.022 | 0.799 | 0.008 | 0.834 | -0.039 | 0.619 | 0.010 | 0.856 | 0.005 | 0.908 | -0.064 | 0.334 | -0.019 | 0.869 | -0.013 | 0.854 |
| L-VLDL | 0.014 | 0.896 | 0.032 | 0.476 | 0.044 | 0.586 | 0.037 | 0.506 | 0.036 | 0.429 | 0.036 | 0.666 | 0.017 | 0.873 | 0.021 | 0.780 |
| VL-VLDL | 0.031 | 0.690 | 0.044 | 0.324 | 0.072 | 0.276 | 0.057 | 0.302 | 0.048 | 0.282 | 0.068 | 0.326 | 0.035 | 0.679 | 0.039 | 0.551 |
| XL-VLDL | 0.045 | 0.464 | 0.049 | 0.263 | 0.108 | 0.061 | 0.077 | 0.147 | 0.057 | 0.182 | 0.115 | 0.058 | 0.052 | 0.526 | 0.060 | 0.301 |
| VLDL | -0.001 | 0.994 | 0.025 | 0.565 | -0.001 | 0.995 | 0.038 | 0.484 | 0.027 | 0.530 | -0.029 | 0.709 | 0.002 | 0.980 | 0.010 | 0.888 |
| S-LDL | -0.011 | 0.916 | 0.008 | 0.834 | -0.042 | 0.592 | 0.016 | 0.790 | 0.003 | 0.955 | -0.065 | 0.334 | -0.013 | 0.914 | -0.012 | 0.861 |
| M-LDL | 0.001 | 0.994 | 0.021 | 0.618 | -0.013 | 0.912 | 0.032 | 0.552 | 0.013 | 0.755 | -0.033 | 0.681 | 0.009 | 0.927 | 0.005 | 0.942 |
| L-LDL | -0.011 | 0.917 | 0.007 | 0.840 | -0.068 | 0.281 | 0.016 | 0.790 | 0.001 | 0.980 | -0.096 | 0.116 | -0.017 | 0.873 | -0.019 | 0.780 |
| LDL | -0.008 | 0.939 | 0.011 | 0.778 | -0.052 | 0.464 | 0.021 | 0.721 | 0.005 | 0.910 | -0.078 | 0.233 | -0.010 | 0.925 | -0.012 | 0.861 |
| IDL | 0.006 | 0.950 | 0.033 | 0.469 | -0.016 | 0.886 | 0.055 | 0.308 | 0.026 | 0.550 | -0.036 | 0.666 | 0.029 | 0.757 | 0.024 | 0.746 |
| S-HDL | 0.117 | 0.003 | 0.132 | 0.000 | 0.179 | 0.001 | 0.145 | 0.001 | 0.122 | 0.000 | 0.191 | 0.000 | 0.143 | 0.002 | 0.155 | 0.000 |
| M-HDL | 0.139 | 0.002 | 0.132 | 0.000 | 0.204 | 0.000 | 0.159 | 0.001 | 0.124 | 0.001 | 0.225 | 0.000 | 0.147 | 0.004 | 0.182 | 0.000 |
| L-HDL | 0.040 | 0.639 | 0.021 | 0.648 | 0.019 | 0.886 | 0.029 | 0.646 | 0.012 | 0.794 | 0.031 | 0.733 | 0.010 | 0.925 | 0.049 | 0.489 |
| XL-HDL | 0.004 | 0.988 | -0.017 | 0.701 | -0.054 | 0.515 | -0.009 | 0.886 | -0.025 | 0.591 | -0.054 | 0.511 | -0.039 | 0.675 | -0.009 | 0.904 |
| HDL | 0.132 | 0.002 | 0.133 | 0.000 | 0.186 | 0.001 | 0.154 | 0.001 | 0.122 | 0.001 | 0.204 | 0.000 | 0.142 | 0.004 | 0.173 | 0.000 |
| Total | 0.125 | 0.003 | 0.130 | 0.000 | 0.171 | 0.002 | 0.151 | 0.001 | 0.118 | 0.001 | 0.185 | 0.001 | 0.135 | 0.006 | 0.164 | 0.000 |
| **Phospholipids** |  |  |  |  |  |  |  |  |  |  |  |  |  |  |  |  |
| VS-VLDL | 0.018 | 0.836 | 0.036 | 0.420 | 0.007 | 0.973 | 0.066 | 0.213 | 0.040 | 0.357 | -0.032 | 0.681 | 0.021 | 0.840 | 0.031 | 0.672 |
| S-VLDL | -0.015 | 0.881 | 0.014 | 0.725 | -0.033 | 0.693 | 0.021 | 0.711 | 0.013 | 0.758 | -0.063 | 0.334 | -0.013 | 0.908 | -0.008 | 0.899 |
| M-VLDL | -0.019 | 0.824 | 0.009 | 0.819 | -0.040 | 0.605 | 0.014 | 0.818 | 0.006 | 0.871 | -0.065 | 0.326 | -0.017 | 0.873 | -0.011 | 0.881 |
| L-VLDL | 0.014 | 0.896 | 0.033 | 0.476 | 0.043 | 0.596 | 0.039 | 0.484 | 0.037 | 0.418 | 0.032 | 0.688 | 0.017 | 0.873 | 0.021 | 0.780 |
| VL-VLDL | 0.021 | 0.812 | 0.035 | 0.446 | 0.058 | 0.405 | 0.051 | 0.346 | 0.040 | 0.395 | 0.052 | 0.485 | 0.027 | 0.780 | 0.030 | 0.689 |
| XL-VLDL | 0.041 | 0.544 | 0.045 | 0.305 | 0.106 | 0.066 | 0.076 | 0.157 | 0.054 | 0.204 | 0.111 | 0.068 | 0.048 | 0.537 | 0.055 | 0.363 |
| VLDL | 0.008 | 0.939 | 0.030 | 0.497 | 0.019 | 0.876 | 0.045 | 0.397 | 0.032 | 0.467 | -0.001 | 0.990 | 0.012 | 0.922 | 0.018 | 0.790 |
| S-LDL | 0.010 | 0.937 | 0.021 | 0.625 | -0.043 | 0.583 | 0.021 | 0.711 | 0.012 | 0.785 | -0.056 | 0.414 | 0.005 | 0.951 | -0.009 | 0.894 |
| M-LDL | 0.029 | 0.690 | 0.048 | 0.260 | 0.009 | 0.949 | 0.056 | 0.293 | 0.039 | 0.383 | -0.003 | 0.969 | 0.036 | 0.661 | 0.029 | 0.689 |
| L-LDL | 0.027 | 0.729 | 0.046 | 0.282 | -0.002 | 0.995 | 0.061 | 0.259 | 0.035 | 0.427 | -0.018 | 0.819 | 0.037 | 0.645 | 0.036 | 0.587 |
| LDL | 0.026 | 0.734 | 0.044 | 0.307 | -0.004 | 0.995 | 0.056 | 0.300 | 0.034 | 0.443 | -0.019 | 0.806 | 0.033 | 0.681 | 0.029 | 0.697 |
| IDL | 0.022 | 0.799 | 0.036 | 0.441 | -0.018 | 0.886 | 0.065 | 0.242 | 0.029 | 0.513 | -0.046 | 0.553 | 0.028 | 0.780 | 0.032 | 0.676 |
| S-HDL | 0.156 | 0.000 | 0.164 | 0.000 | 0.251 | 0.000 | 0.195 | 0.000 | 0.160 | 0.000 | 0.265 | 0.000 | 0.186 | 0.000 | 0.206 | 0.000 |
| M-HDL | 0.158 | 0.000 | 0.149 | 0.000 | 0.243 | 0.000 | 0.187 | 0.000 | 0.145 | 0.000 | 0.263 | 0.000 | 0.173 | 0.000 | 0.205 | 0.000 |
| L-HDL | 0.073 | 0.217 | 0.049 | 0.321 | 0.075 | 0.299 | 0.067 | 0.275 | 0.041 | 0.412 | 0.091 | 0.211 | 0.053 | 0.537 | 0.086 | 0.146 |
| XL-HDL | 0.003 | 0.988 | -0.025 | 0.609 | -0.048 | 0.587 | -0.012 | 0.851 | -0.030 | 0.530 | -0.046 | 0.602 | -0.035 | 0.701 | -0.009 | 0.899 |
| HDL | 0.142 | 0.002 | 0.127 | 0.000 | 0.199 | 0.001 | 0.161 | 0.001 | 0.120 | 0.001 | 0.217 | 0.000 | 0.144 | 0.006 | 0.179 | 0.000 |
| Total | 0.101 | 0.025 | 0.108 | 0.002 | 0.127 | 0.024 | 0.142 | 0.002 | 0.101 | 0.004 | 0.122 | 0.040 | 0.107 | 0.048 | 0.131 | 0.003 |
| **Phospholipids to Total lipids** | |  |  |  |  |  |  |  |  |  |  |  |  |  |  |  |
| VS-VLDL | -0.014 | 0.896 | -0.013 | 0.735 | -0.039 | 0.605 | -0.028 | 0.582 | 0.001 | 0.978 | -0.070 | 0.275 | -0.030 | 0.701 | -0.039 | 0.530 |
| S-VLDL | -0.033 | 0.674 | -0.029 | 0.518 | -0.120 | 0.037 | -0.044 | 0.420 | -0.046 | 0.315 | -0.118 | 0.055 | -0.046 | 0.554 | -0.051 | 0.405 |
| M-VLDL | -0.037 | 0.633 | -0.021 | 0.625 | -0.098 | 0.107 | -0.010 | 0.863 | -0.030 | 0.513 | -0.115 | 0.066 | -0.048 | 0.554 | -0.031 | 0.689 |
| L-VLDL | -0.013 | 0.896 | 0.007 | 0.840 | 0.014 | 0.895 | 0.029 | 0.580 | 0.026 | 0.536 | -0.013 | 0.858 | 0.004 | 0.952 | 0.003 | 0.963 |
| VL-VLDL | -0.050 | 0.367 | -0.039 | 0.333 | -0.065 | 0.280 | -0.011 | 0.848 | -0.025 | 0.530 | -0.096 | 0.094 | -0.046 | 0.534 | -0.052 | 0.354 |
| XL-VLDL | 0.000 | 0.998 | 0.014 | 0.707 | 0.030 | 0.698 | 0.036 | 0.484 | 0.020 | 0.618 | 0.017 | 0.817 | 0.010 | 0.923 | 0.003 | 0.963 |
| S-LDL | -0.024 | 0.744 | -0.044 | 0.281 | -0.086 | 0.129 | -0.076 | 0.123 | -0.045 | 0.279 | -0.084 | 0.158 | -0.040 | 0.577 | -0.069 | 0.175 |
| M-LDL | -0.024 | 0.734 | -0.015 | 0.685 | -0.067 | 0.257 | -0.049 | 0.320 | -0.018 | 0.649 | -0.059 | 0.336 | -0.023 | 0.789 | -0.044 | 0.430 |
| L-LDL | -0.044 | 0.447 | -0.020 | 0.618 | -0.014 | 0.889 | -0.016 | 0.778 | -0.019 | 0.625 | -0.002 | 0.969 | 0.007 | 0.944 | -0.008 | 0.899 |
| IDL | -0.061 | 0.211 | -0.087 | 0.010 | -0.119 | 0.023 | -0.082 | 0.078 | -0.076 | 0.028 | -0.170 | 0.002 | -0.108 | 0.027 | -0.108 | 0.010 |
| S-HDL | 0.093 | 0.037 | 0.074 | 0.044 | 0.170 | 0.002 | 0.112 | 0.015 | 0.085 | 0.016 | 0.179 | 0.001 | 0.106 | 0.044 | 0.130 | 0.002 |
| M-HDL | 0.027 | 0.737 | 0.023 | 0.617 | 0.084 | 0.194 | 0.060 | 0.293 | 0.044 | 0.345 | 0.075 | 0.282 | 0.057 | 0.491 | 0.029 | 0.702 |
| L-HDL | 0.173 | 0.000 | 0.160 | 0.000 | 0.297 | 0.000 | 0.195 | 0.000 | 0.178 | 0.000 | 0.320 | 0.000 | 0.211 | 0.000 | 0.200 | 0.000 |
| XL-HDL | -0.046 | 0.489 | -0.069 | 0.113 | -0.078 | 0.247 | -0.055 | 0.323 | -0.070 | 0.091 | -0.076 | 0.282 | -0.054 | 0.527 | -0.054 | 0.399 |
| **Total lipids** |  |  |  |  |  |  |  |  |  |  |  |  |  |  |  |  |
| VS-VLDL | 0.020 | 0.812 | 0.038 | 0.377 | 0.012 | 0.924 | 0.072 | 0.167 | 0.042 | 0.346 | -0.026 | 0.733 | 0.025 | 0.780 | 0.036 | 0.587 |
| S-VLDL | -0.007 | 0.946 | 0.021 | 0.618 | -0.005 | 0.995 | 0.028 | 0.593 | 0.024 | 0.572 | -0.032 | 0.682 | -0.006 | 0.951 | 0.002 | 0.971 |
| M-VLDL | -0.011 | 0.917 | 0.015 | 0.705 | -0.021 | 0.846 | 0.016 | 0.790 | 0.014 | 0.748 | -0.041 | 0.602 | -0.011 | 0.923 | -0.005 | 0.942 |
| L-VLDL | 0.013 | 0.901 | 0.030 | 0.497 | 0.037 | 0.654 | 0.033 | 0.559 | 0.032 | 0.477 | 0.030 | 0.707 | 0.015 | 0.888 | 0.018 | 0.790 |
| VL-VLDL | 0.033 | 0.674 | 0.044 | 0.324 | 0.074 | 0.254 | 0.058 | 0.300 | 0.048 | 0.280 | 0.072 | 0.290 | 0.037 | 0.661 | 0.042 | 0.528 |
| XL-VLDL | 0.046 | 0.464 | 0.049 | 0.260 | 0.108 | 0.061 | 0.075 | 0.165 | 0.056 | 0.185 | 0.116 | 0.056 | 0.053 | 0.508 | 0.062 | 0.278 |
| VLDL | 0.016 | 0.871 | 0.035 | 0.438 | 0.039 | 0.634 | 0.048 | 0.360 | 0.038 | 0.401 | 0.025 | 0.740 | 0.020 | 0.859 | 0.027 | 0.711 |
| S-LDL | 0.016 | 0.871 | 0.032 | 0.476 | -0.017 | 0.886 | 0.040 | 0.466 | 0.023 | 0.586 | -0.030 | 0.703 | 0.016 | 0.880 | 0.008 | 0.899 |
| M-LDL | 0.032 | 0.674 | 0.049 | 0.260 | 0.018 | 0.886 | 0.061 | 0.252 | 0.040 | 0.362 | 0.005 | 0.958 | 0.038 | 0.634 | 0.034 | 0.628 |
| L-LDL | 0.032 | 0.674 | 0.046 | 0.281 | -0.001 | 0.995 | 0.060 | 0.274 | 0.036 | 0.418 | -0.019 | 0.802 | 0.034 | 0.679 | 0.034 | 0.622 |
| LDL | 0.030 | 0.686 | 0.046 | 0.281 | 0.002 | 0.995 | 0.059 | 0.276 | 0.036 | 0.418 | -0.014 | 0.850 | 0.033 | 0.679 | 0.032 | 0.654 |
| IDL | 0.035 | 0.669 | 0.053 | 0.246 | 0.007 | 0.973 | 0.081 | 0.132 | 0.044 | 0.334 | -0.011 | 0.883 | 0.049 | 0.537 | 0.054 | 0.394 |
| S-HDL | 0.145 | 0.000 | 0.156 | 0.000 | 0.230 | 0.000 | 0.182 | 0.000 | 0.150 | 0.000 | 0.243 | 0.000 | 0.173 | 0.000 | 0.190 | 0.000 |
| M-HDL | 0.151 | 0.000 | 0.143 | 0.000 | 0.226 | 0.000 | 0.175 | 0.000 | 0.136 | 0.000 | 0.247 | 0.000 | 0.162 | 0.001 | 0.196 | 0.000 |
| L-HDL | 0.055 | 0.417 | 0.033 | 0.507 | 0.043 | 0.639 | 0.045 | 0.461 | 0.024 | 0.610 | 0.056 | 0.497 | 0.030 | 0.780 | 0.064 | 0.338 |
| XL-HDL | 0.003 | 0.988 | -0.024 | 0.618 | -0.053 | 0.522 | -0.013 | 0.848 | -0.031 | 0.530 | -0.052 | 0.529 | -0.036 | 0.691 | -0.011 | 0.888 |
| HDL | 0.129 | 0.005 | 0.116 | 0.002 | 0.172 | 0.004 | 0.143 | 0.003 | 0.107 | 0.005 | 0.190 | 0.002 | 0.126 | 0.026 | 0.163 | 0.000 |
| Total | 0.068 | 0.191 | 0.081 | 0.027 | 0.079 | 0.194 | 0.106 | 0.024 | 0.075 | 0.044 | 0.069 | 0.290 | 0.072 | 0.262 | 0.088 | 0.071 |
| **Triglycerides** |  |  |  |  |  |  |  |  |  |  |  |  |  |  |  |  |
| VS-VLDL | 0.052 | 0.355 | 0.063 | 0.111 | 0.092 | 0.102 | 0.089 | 0.063 | 0.073 | 0.046 | 0.068 | 0.290 | 0.048 | 0.534 | 0.060 | 0.278 |
| S-VLDL | 0.011 | 0.917 | 0.030 | 0.497 | 0.044 | 0.577 | 0.035 | 0.515 | 0.037 | 0.401 | 0.030 | 0.701 | 0.006 | 0.951 | 0.019 | 0.780 |
| M-VLDL | 0.014 | 0.896 | 0.032 | 0.476 | 0.034 | 0.689 | 0.030 | 0.578 | 0.033 | 0.452 | 0.026 | 0.740 | 0.011 | 0.922 | 0.017 | 0.816 |
| L-VLDL | 0.025 | 0.744 | 0.036 | 0.428 | 0.053 | 0.464 | 0.036 | 0.515 | 0.037 | 0.418 | 0.055 | 0.444 | 0.027 | 0.780 | 0.028 | 0.701 |
| XL-VLDL | 0.046 | 0.464 | 0.053 | 0.246 | 0.096 | 0.107 | 0.066 | 0.228 | 0.057 | 0.185 | 0.101 | 0.106 | 0.050 | 0.534 | 0.055 | 0.376 |
| XXL-VLDL | 0.049 | 0.417 | 0.049 | 0.260 | 0.110 | 0.055 | 0.073 | 0.167 | 0.055 | 0.185 | 0.121 | 0.043 | 0.056 | 0.476 | 0.066 | 0.247 |
| VLDL | 0.033 | 0.674 | 0.044 | 0.321 | 0.075 | 0.245 | 0.054 | 0.318 | 0.048 | 0.272 | 0.074 | 0.275 | 0.035 | 0.679 | 0.042 | 0.514 |
| S-LDL | 0.057 | 0.305 | 0.064 | 0.111 | 0.099 | 0.081 | 0.086 | 0.083 | 0.070 | 0.062 | 0.094 | 0.124 | 0.050 | 0.527 | 0.060 | 0.286 |
| M-LDL | 0.086 | 0.060 | 0.091 | 0.010 | 0.127 | 0.022 | 0.116 | 0.012 | 0.096 | 0.005 | 0.116 | 0.048 | 0.078 | 0.194 | 0.084 | 0.084 |
| L-LDL | 0.104 | 0.017 | 0.107 | 0.001 | 0.145 | 0.008 | 0.140 | 0.001 | 0.114 | 0.001 | 0.128 | 0.026 | 0.100 | 0.057 | 0.105 | 0.019 |
| LDL | 0.095 | 0.033 | 0.099 | 0.004 | 0.136 | 0.013 | 0.129 | 0.003 | 0.105 | 0.002 | 0.122 | 0.035 | 0.090 | 0.110 | 0.095 | 0.041 |
| IDL | 0.082 | 0.071 | 0.088 | 0.011 | 0.121 | 0.025 | 0.122 | 0.006 | 0.097 | 0.004 | 0.098 | 0.094 | 0.080 | 0.170 | 0.087 | 0.065 |
| S-HDL | 0.062 | 0.259 | 0.080 | 0.036 | 0.129 | 0.023 | 0.103 | 0.037 | 0.089 | 0.015 | 0.118 | 0.050 | 0.071 | 0.285 | 0.086 | 0.089 |
| M-HDL | 0.072 | 0.140 | 0.087 | 0.011 | 0.145 | 0.006 | 0.119 | 0.006 | 0.096 | 0.004 | 0.136 | 0.016 | 0.083 | 0.138 | 0.111 | 0.009 |
| L-HDL | 0.039 | 0.528 | 0.050 | 0.237 | 0.073 | 0.215 | 0.071 | 0.155 | 0.057 | 0.140 | 0.062 | 0.326 | 0.026 | 0.775 | 0.070 | 0.162 |
| XL-HDL | 0.026 | 0.729 | 0.035 | 0.401 | 0.040 | 0.587 | 0.052 | 0.302 | 0.041 | 0.322 | 0.028 | 0.709 | 0.005 | 0.951 | 0.037 | 0.541 |
| HDL | 0.061 | 0.235 | 0.076 | 0.034 | 0.122 | 0.023 | 0.103 | 0.024 | 0.085 | 0.014 | 0.111 | 0.050 | 0.063 | 0.314 | 0.093 | 0.040 |
| Total | 0.044 | 0.479 | 0.055 | 0.210 | 0.089 | 0.135 | 0.070 | 0.187 | 0.060 | 0.136 | 0.085 | 0.184 | 0.045 | 0.554 | 0.055 | 0.364 |
| **Triglycerides to Total lipids** | |  |  |  |  |  |  |  |  |  |  |  |  |  |  |  |
| VS-VLDL | 0.072 | 0.210 | 0.068 | 0.129 | 0.174 | 0.004 | 0.078 | 0.167 | 0.084 | 0.040 | 0.179 | 0.005 | 0.066 | 0.386 | 0.066 | 0.278 |
| S-VLDL | 0.042 | 0.513 | 0.030 | 0.497 | 0.125 | 0.027 | 0.038 | 0.484 | 0.044 | 0.322 | 0.136 | 0.024 | 0.049 | 0.534 | 0.052 | 0.394 |
| M-VLDL | 0.059 | 0.320 | 0.045 | 0.324 | 0.141 | 0.018 | 0.052 | 0.346 | 0.058 | 0.185 | 0.151 | 0.017 | 0.071 | 0.314 | 0.062 | 0.310 |
| L-VLDL | 0.048 | 0.367 | 0.022 | 0.581 | 0.061 | 0.306 | 0.005 | 0.936 | 0.011 | 0.785 | 0.098 | 0.083 | 0.047 | 0.527 | 0.040 | 0.487 |
| XL-VLDL | 0.052 | 0.367 | 0.034 | 0.445 | 0.118 | 0.033 | 0.046 | 0.376 | 0.034 | 0.429 | 0.142 | 0.016 | 0.077 | 0.207 | 0.062 | 0.267 |
| XXL-VLDL | -0.009 | 0.937 | -0.026 | 0.522 | -0.002 | 0.995 | -0.012 | 0.819 | -0.027 | 0.511 | 0.018 | 0.802 | 0.002 | 0.980 | 0.015 | 0.825 |
| S-LDL | 0.065 | 0.211 | 0.059 | 0.158 | 0.139 | 0.013 | 0.094 | 0.055 | 0.076 | 0.042 | 0.133 | 0.024 | 0.056 | 0.463 | 0.076 | 0.142 |
| M-LDL | 0.088 | 0.053 | 0.069 | 0.073 | 0.151 | 0.006 | 0.105 | 0.025 | 0.088 | 0.013 | 0.138 | 0.017 | 0.067 | 0.291 | 0.079 | 0.116 |
| L-LDL | 0.091 | 0.048 | 0.078 | 0.037 | 0.159 | 0.004 | 0.109 | 0.022 | 0.100 | 0.004 | 0.146 | 0.014 | 0.079 | 0.197 | 0.077 | 0.141 |
| IDL | 0.069 | 0.210 | 0.059 | 0.182 | 0.136 | 0.020 | 0.081 | 0.132 | 0.079 | 0.044 | 0.118 | 0.058 | 0.046 | 0.560 | 0.054 | 0.389 |
| S-HDL | -0.002 | 0.988 | 0.014 | 0.725 | 0.036 | 0.672 | 0.031 | 0.578 | 0.031 | 0.501 | 0.016 | 0.839 | 0.000 | 0.996 | 0.005 | 0.942 |
| M-HDL | -0.004 | 0.986 | 0.018 | 0.665 | 0.038 | 0.639 | 0.034 | 0.527 | 0.033 | 0.463 | 0.017 | 0.829 | 0.003 | 0.965 | 0.014 | 0.851 |
| L-HDL | -0.024 | 0.793 | 0.017 | 0.684 | 0.022 | 0.846 | 0.011 | 0.854 | 0.026 | 0.555 | 0.005 | 0.958 | -0.024 | 0.813 | 0.003 | 0.963 |
| XL-HDL | 0.046 | 0.487 | 0.072 | 0.105 | 0.110 | 0.072 | 0.071 | 0.217 | 0.078 | 0.054 | 0.109 | 0.094 | 0.042 | 0.616 | 0.075 | 0.195 |

Model adjusted for age, sex, education, race, socioeconomic status, body mass index, smoking status, alcohol drinking status, physical activity, social connection, hypertension, diabetes, heart disease, beta-blockers, calcium blockers, lipid-lowering, and APOE ε4 status.
Abbreviation: Apo, apolipoprotein; LDL-AD, average diameter for LDL particles; CHOL, cholesterol; CE, cholesteryl esters; TG, triglycerides; S, small; M, medium; L, large; VL, very large; XL, extremely large; VLDL, very low-density lipoprotein; LDL; low-density lipoprotein; IDL, intermediate-density lipoprotein; HDL, high-density lipoprotein; LA, linoleic acid; MUFA, monounsaturated fatty acid; n-6, omega-6 fatty acid; PUFA, polyunsaturated fatty acid; SFA, saturated fatty acid; FA, fatty acid.

Model Abbreviations:

1. XGBoost

2. Support Vector Machines

3. Lasso_FeatureWiz feature selector

4. XGBoost_FeatureWiz feature selector

5. Support Vector Machines_FeatureWiz feature selector

6. Lasso_Recursive feature elimination cross-validation feature selector

7. XGBoost_Recursive feature elimination cross-validation feature selector

8. Support Vector Machines_Recursive feature elimination cross-validation feature selector

**
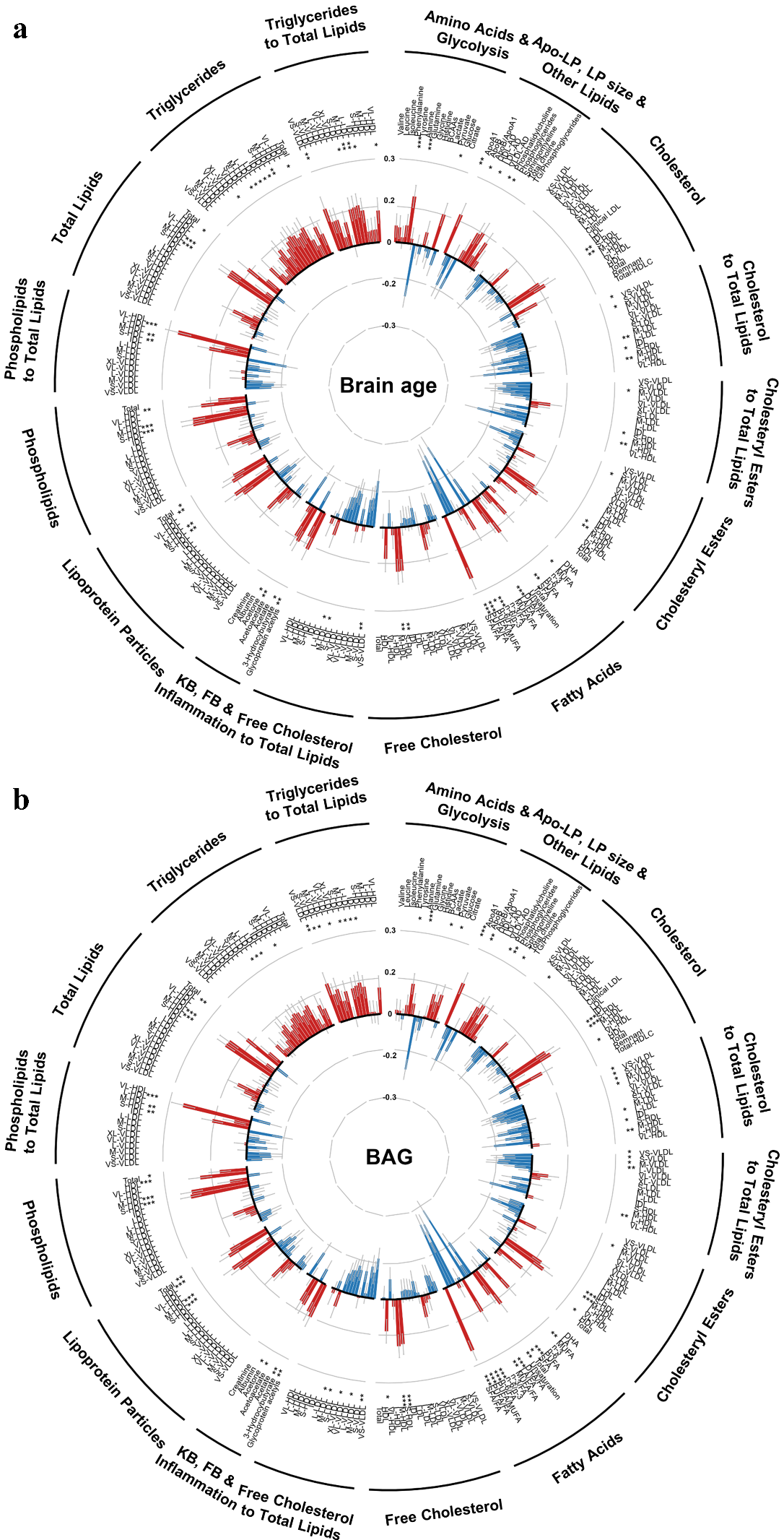
**

**Figure S1.** 249 individual metabolites in relation to (a) brain age and (b) brain age gap.

Bar colors illustrate the directionality of association: positive (red), and negative (blue).

*, **, and *** indicate Benjamini-Hochberg for false discovery rate-corrected *P*<0.05, *P*<0.01, and *P*<0.001, respectively.

All models adjusted for age, sex, education, race, socioeconomic status, body mass index, smoking status, alcohol drinking status, physical activity, social connection, hypertension, diabetes, heart disease, beta-blockers, calcium blockers, lipid-lowering, and *APOE* ε4 status.

Abbreviation: BCAAs, branched-chain amino acids; Apo, apolipoprotein; HDL-AD, average diameter for HDL particles; LDL-AD, average diameter for LDL particles; VLDL-AD, average diameter for VLDL particles; TG, triglycerides; VS, very small; S, small; M, medium; L, large; VL, very large; XL, extremely large; VLDL, very low-density lipoprotein; LDL, low-density lipoprotein; IDL, intermediate-density lipoprotein; HDL, high-density lipoprotein; Remnant, remnant cholesterol (non-HDL, non-LDL-cholesterol); DHA, docosahexaenoic acid; LA, linoleic acid; MUFA, monounsaturated fatty acid; n-3, omega-3 fatty acid; n-6, omega-6 fatty acid; PUFA, polyunsaturated fatty acid; SFA, saturated fatty acid; FA, fatty acid; Unsaturation, degree of unsaturation.
